# The curse and blessing of abundance—the evolution of drug interaction databases and their impact on drug network analysis

**DOI:** 10.1093/gigascience/giad011

**Published:** 2023-03-09

**Authors:** Mihai Udrescu, Sebastian Mihai Ardelean, Lucreţia Udrescu

**Affiliations:** Department of Computer and Information Technology, Politehnica University of Timişoara, Timişoara 300223, Romania; Department of Computer and Information Technology, Politehnica University of Timişoara, Timişoara 300223, Romania; Department I—Drug Analysis, “Victor Babeş” University of Medicine and Pharmacy Timişoara, Timişoara 300041, Romania

**Keywords:** drug–drug interaction networks, drug–target interaction networks, drug database integrity, analysis robustness of drug networks

## Abstract

**Background:**

Widespread bioinformatics applications such as drug repositioning or drug–drug interaction prediction rely on the recent advances in machine learning, complex network science, and comprehensive drug datasets comprising the latest research results in molecular biology, biochemistry, or pharmacology. The problem is that there is much uncertainty in these drug datasets—we know the drug–drug or drug–target interactions reported in the research papers, but we cannot know if the not reported interactions are absent or yet to be discovered. This uncertainty hampers the accuracy of such bioinformatics applications.

**Results:**

We use complex network statistics tools and simulations of randomly inserted previously unaccounted interactions in drug–drug and drug–target interaction networks—built with data from DrugBank versions released over the plast decade—to investigate whether the abundance of new research data (included in the latest dataset versions) mitigates the uncertainty issue. Our results show that the drug–drug interaction networks built with the latest dataset versions become very dense and, therefore, almost impossible to analyze with conventional complex network methods. On the other hand, for the latest drug database versions, drug–target networks still include much uncertainty; however, the robustness of complex network analysis methods slightly improves.

**Conclusions:**

Our big data analysis results pinpoint future research directions to improve the quality and practicality of drug databases for bioinformatics applications: benchmarking for drug–target interaction prediction and drug–drug interaction severity standardization.

Key points

- Many notable and helpful bioinformatics applications, such as drug repositioning and drug–drug interaction prediction, employ machine learning methods and statistical analysis on drug–drug and drug–target interaction network features (i.e., parameters/metrics and centralities).
- The main problem with the approach based on the drug interaction network analysis is that there is much uncertainty in drug databases: the reported drug–drug and drug–target interactions are certain, but it is uncertain if the nonreported interactions do not exist or are yet to be uncovered.
- Versioned drug databases—including DrugBank—record research results accumulated over the past decade, thus enabling network analysis that exposes the weak points and provides hints to improve the drug databases’ practicality and the drug network analysis’s accuracy.
- Our complex network analysis on the evolution of drug–drug and drug–target interaction networks shows that the drug–drug interaction networks made with the latest dataset versions have become too dense to analyze with established complex network methods.
- We do not notice the same density increase in drug–target networks; despite containing much uncertainty, the robustness of drug–target network analysis methods scarcely improves with the evolution of drug database versions.
- Our investigation provides guidance for future studies to improve the usefulness of drug databases and the accuracy of bioinformatics applications: standardization of drug–drug interaction labeling and delivering a comprehensive benchmark dataset for drug–target interaction prediction.

## Background

Spurred by the fast development of efficient complex network analysis tools based on machine learning and the ever-growing drug/medicine databases we witnessed over the past decade, processing drug interaction networks became an appealing drug design method [1, 2]. Such drug interaction networks can represent various relationships or interactions involving active substances (e.g., drug–drug interactions, drug–target interactions, drug–gene interactions, drug–disease relationships, drug–adverse reaction relationships). As such, harnessing big amounts of data describing the intricate drug interactions (or relationships) with other entities can lead to uncovering new drug properties: previously unknown drug–drug [3–6] or drug–target interactions [7–11] and drug repositioning [12–14].

Computational drug–drug interaction (DDI) prediction is critical in designing effective and safe drug combinations and developing drugs [5, 6]. Drug–target interaction (DTI) prediction is a key-step strategy in drug discovery that leads to uncovering new drugs for biological targets and new targets for known, approved drugs—representing a crucial step toward drug repositioning and identifying possible side effects [8, 11].

Drug repositioning (or repurposing) means finding new applications for drugs already in use [15]; the drug propensity for multiple functions underpins this undertaking. Before the upswing of big data and machine learning, pharmacologists and medical doctors mainly relied on serendipity to uncover drug repositionings [16]. The most illustrative example is aspirin, introduced as an antipyretic but—over time—revealed as having painkiller and antiplatelet effects. The prediction of drug–drug interactions and drug–drug interaction severity is another drug–interaction network analysis application with substantial benefits in therapeutic practice [17–19]. It is clear that predicting either adverse or synergistic interactions in drug–drug interaction networks helps to tailor effective therapies for patients with multiple comorbidities.

In comparison with traditional drug design, drug repositioning entails simpler testing and validation procedures (because many adverse events and effects, as well as interactions with food and other drugs, are already known and tested), which translates into reduced costs and approval times [20, 21]. (In the Food and Drug Administration [FDA] New Drug Therapy Approvals 2021, 50 drugs are are *new molecular entities* or *new therapeutic biologics*, and 17 are *drug repositionings* [22].) All these arguments make drug repositioning tempting for exceptional therapeutical cases, such as orphan diseases—for which there is insufficient research funding [23]—and new-pathogen epidemic diseases [24]—for which timing is essential. Particularly relevant is the case of the COVID-19 pandemic, where drug repositioning proved to be a valuable therapeutical method for quick public health care response, given that the conventional drug design requires a substantial amount of time. Indeed, many COVID-19 repositioning predictions uncovered with big data exploration and complex drug networks were confirmed by *in vitro* and *in vivo* experiments [25].

Despite the advances in machine learning, big data mining, complex network analysis, and the undeniable benefits of computational drug repositioning, the field still has significant problems. The most critical issue that affects the robustness of computer-based drug network analysis is that the drug interactions/relationships (e.g., with other drugs, targets, adverse reactions, or diseases) in drug databases primarily reflect what we know as positive information from *in vitro* and *in vivo* experiments. We have little negative information (i.e., interactions we know for sure that do not exist). If we do not have information about a specific drug interacting with a particular target, this does not mean that there is no way they interact; after all, “absence of evidence is not evidence of absence” [26].

Consequently, in computational drug repositioning, we do not have a robust ground truth to operate with; this can seriously affect the analysis accuracy of complex drug interaction networks. Mestres et al. [27] have articulated this idea very eloquently by showing how the network analysis based on degree centrality hierarchization of drugs in a network built with information from one database is affected by adding the information from another database. (In complex networks, a centrality expresses the importance of a network node/vertex; most network-based analysis approaches use either centrality hierarchization or community detection methods.) Nonetheless, the enormous benefits of confirming (with *in vitro* and *in vivo* methods) even a few drug repositionings—uncovered by computational big data approaches—offset such accuracy problems.

As long as we do not have comprehensive negative information, the issue of uncertainty in drug datasets remains. However, the fact that—over the years—the comprehensiveness of drug datasets (such as DrugBank [28]) has constantly grown, and experts curated the data according to the latest literature results, may suggest that the integrity of the data improved. (The integrity characterizes the “absence of improper data alterations” [29].) Indeed, the constant growth of the most comprehensive drug database (reflected in the evolution of the various complex drug network parameters and centralities) may have mitigated the concerns formulated by Mestres et al. [27]. Our article investigates how the evolution of knowledge on drug interactions mirrored by the DrugBank database impacts the robustness of drug interaction network analysis based on centrality hierarchization methods; future research can exploit the insight we get to advance drug repositioning and drug–drug interaction prediction methods.

We build the DDI and DTI networks with the data from DrugBank versions 3.0 to 5.1.9. Over the years, many research papers used DDI [30–35] and DTI networks [36–40] for computational drug repositioning and drug–drug interaction prediction. A drug–drug interaction occurs when one drug influences the action/effect of another drug in a biological environment. Such a drug–drug relationship signifies that one drug augments or, conversely, mitigates the effect of the other one; either way, this generally translates into a clinically potential harmful situation. A drug–target interaction exists if the drug exerts a specific action upon a biological target (generally, a protein or enzyme), thus producing a pharmacologic effect [40].

**Figure 1:**
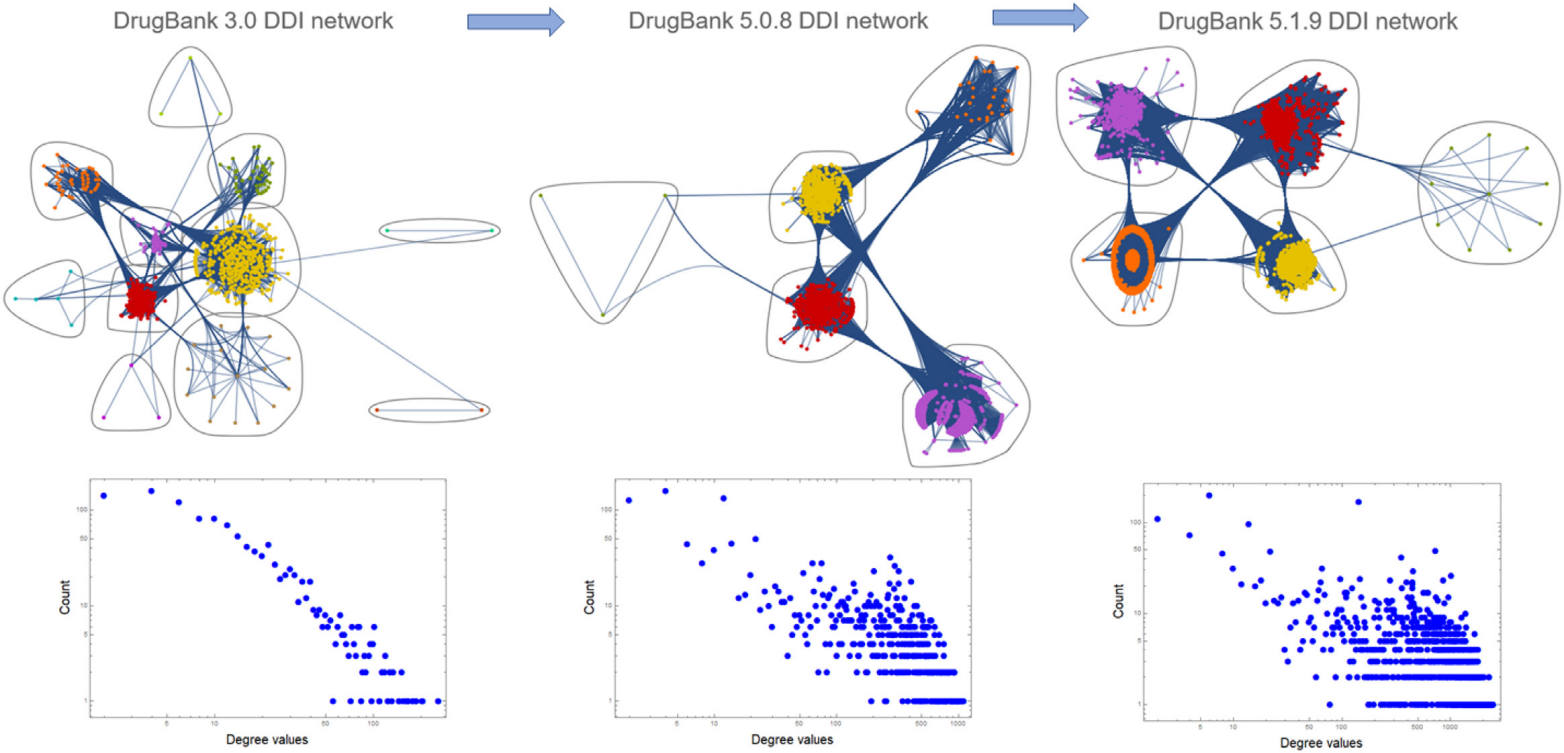
Drug–drug interaction (DDI) network evolution with the DrugBank data versions, showing the massive increase in density from version 3.0 to 5.1.9. Consequently, the number of network clusters/communities generated with hierarchical clustering in Mathematica 13.0 substantially decreases (nodes represent drugs, links represent drug–drug interactions, and node colors represent the distinct clusters/communities of network nodes). Also, the panels below show how the increase in density alters the power-law degree distribution in the DDI networks corresponding to the latest DrugBank versions.

**Figure 2:**
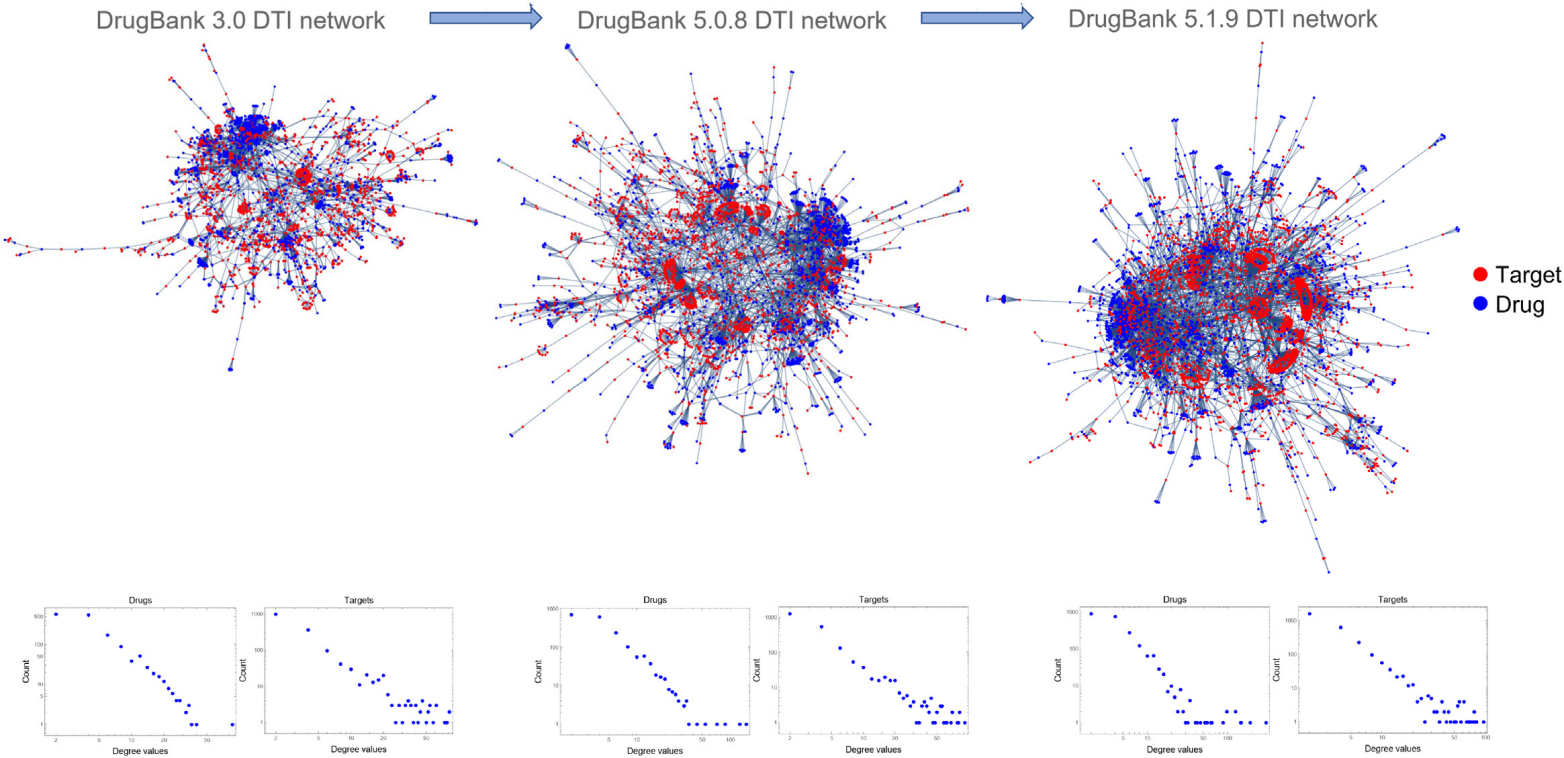
The evolution of drug–target interaction (DTI) bipartite networks with the successive DrugBank data versions, where the red nodes represent biological targets, the blue nodes represent drugs, and the links represent drug–target interactions. Although the DTI network sizes increase with the successive DrugBank data, the network structure and the degree distributions in both drugs and targets (see the panels below) are not altered substantially.

We found the motivation of our research in the visual inspection of the evolution of DDI and DTI networks over the years and across the successive DrugBank versions. Fig. 1 shows how the DDI network density increased from version 3.0 to 5.1.9, via 5.0.8, such that the number of network clusters/communities substantially decreased; moreover, as shown in the panels below, the initial power-law degree distribution in DrugBank 3.0 DDI is altered in the subsequent versions. In contrast with the DDI network evolution presented in Fig. 1, the equivalent evolution for DTI networks in Fig. 2 does not increase the density substantially, and the structure does not change too much despite the increasing size of the networks—from version 3.0 to 5.0.8 and 5.1.9. Also, the panels below the DTI networks in Fig. 2 show that the DTI degree distributions in both drugs and targets are power-law across all DrugBank versions. Indeed, this discrepancy between DDI and DTI network evolution over time (as reflected by the successive DrugBank data versions) inspires the study we present in this article; the results obtained provide valuable insight for researchers developing big data techniques in systems and networks pharmacology, aiming at applications such as computational drug repositioning or drug–drug interaction prediction.

The contributions of our DDI and DTI network analysis across the DrugBank database versions are as follows:

- We present for the first time the evolution of various complex network parameters and centralities [41], in drug–drug and drug–target interaction networks as the knowledge on drug–drug and drug–target interactions grew over more than a decade; we comment on the far-reaching consequences of data evolution on the computational analysis tools used in drug repositioning.
- We estimate the integrity of the processed data using Benford’s law on the most prominent network centralities (i.e., degree and betweenness).
- We test the robustness of centrality hierarchization analysis methods in drug interaction networks using our algorithm that automatically adds unknown interactions in ascending ratios to notice how this process affects the drugs’ order of importance.

Accordingly, one main finding is that the DDI network parameters and centralities distributions were close to those of the typical complex networks in the earlier DrugBank versions but deviated markedly in the latest versions. Instead, the DTI parameters and network centralities distributions oscillate but remain close to the typical complex network ranges across all DrugBank versions. (We explain the typical characteristics of real-world complex networks in the “Typical complex network characteristics” section.)

Such an evolution of the DDI networks owes to their enormous increase in density (i.e., many recent experimental results report new drug–drug interactions); however, typical complex networks are patently sparse. The overarching conclusion is that complex network analysis became irrelevant in the DDI networks built with the latest database versions data. Also, the integrity analysis of the DTI data finds that even the latest DrugBank versions still miss many unaccounted drug–target interactions; therefore, the robustness of the centrality hierarchization analysis (i.e., degree, as suggested in [27]) in DTI networks improved only marginally with the new data in the latest versions. Such a situation calls for an intensified effort to uncover new drug–target interactions; indeed, in 2021, only 17 new drug–target interactions were approved [42].

## Data Description

We chose the DrugBank dataset [28, 43, 44] because it is the most comprehensive and robust drug database, being curated manually by experts and scientists, according to the latest scientific discoveries reported in the literature; it is also versioned, which allows for analyzing the evolution of knowledge over time [28].

We downloaded all DrugBank versions recorded as XML files over a decade, from version 3.0 (January 2011) to version 5.1.9 (January 2022). Access to download the database versions is free with a validated account; an account can be created with an institutional email. The DrugBank versions 5.1.9 to 4.5.0 can be downloaded from https://go.drugbank.com/releases and 4.3 to 3.0 from https://go.drugbank.com/downloads/archived.

For each medicine, DrugBank lists many parameters, properties, and extensive information, such as the generic name, brand names, indications, type, drug categories, ATC codes, chemical structure and formula, chemical identifiers, associated conditions, pharmacodynamics, mechanism of action, metabolism, toxicity, pathways, drug interactions, food interactions, clinical trials, patents, targets, enzymes, carriers, transporters, and so forth. Nonetheless, our present study analyzes the evolution of drug–drug and drug–target interaction networks (DDI and DTI), such that the only information we need from each drug in all DrugBank versions is *drug interactions* and *targets*. Also, we included information concerning *approved* human drugs only (consequently, we excluded the investigational, experimental, withdrawn, or vet-approved drugs).

For every drug in each database version, DrugBank lists the drugs with which it interacts; for example, in DrugBank 5.1.9, ibuprofen has a list of 1,236 approved interacting drugs. Each interaction has a text description; for the ibuprofen entry, the first interacting drug is abacavir, and the corresponding description reads, “Ibuprofen may decrease the excretion rate of Abacavir which could result in a higher serum level.” At the same time, all DrugBank versions provide, for each drug entry, a list of interacting targets. For each interacting target, we find the following information: kind, organism (humans, in our case), pharmacologic action, actions, general function, specific function, gene name, Uniprot ID, Uniprot name, molecular weight, and a list of references (i.e., scientific papers) to support the provided details experimentally. To continue with our example, DrugBank 5.1.9—the latest database version—lists 10 targets for ibuprofen (all proteins), from prostaglandin G/H synthase 2 to protein S100-A7.

## Problem Formulation

### Complex networks

A *complex network* is a graph *G* consisting of a node (or vertex) set *V* and a link (or edge) set *E, G* = (*V, E*), where any edge *e_ij_* ∈ *E* connects nodes *v_i_, v_j_* ∈ *V*. Additionally, compared to normal graphs, real-world complex networks have a large number of nodes |*V*| (i.e., hundreds to millions) and intricate, nontrivial topologies (i.e., links do not form regular structures but are not completely random either) [41, 45–47]. If the nodes *v_i_* ∈ *V* are of the same type, *G* is a *monopartite* network; if any node *v_i_* belongs to one of the *m* disjoint node subsets (*V*_1_ ∪*V*_2_… ∪*V_m_* = *V*), then the network is *multipartite* or *m*-partite.

The network is *unweighted* if the weights of its edges/links *e_ij_* ∈ *E* are *w_ij_* = 1. (In other words, if *w_ij_* = 1, we have a link between nodes *v_i_* and *v_j_*, and if *w_ij_* = 0, there is no link between *v_i_* and *v_j_*.) The network is *weighted* if *w_ij_* are not binary values (i.e., $w_{ij}\in \mathbb {R}$); values *w_ij_* = 0 also correspond to nonedges between *v_i_* and *v_j_*. We say that the network is *directed* if ∃*i, j* (*i* ≠ *j*) such that  *w_ij_* ≠ *w_ji_*. Unweighted networks are directed if ∃*e_ij_* ∈ *E* with *w_ij_* = 0 and *w_ji_* = 1.

Such networks *G* can also be expressed as adjacency matrices *W* containing elements *w_ij_*, with *w_ij_* = 0 when there is no link/edge between nodes *v_i_* and *v_j_*. In unweighted networks, *w_ij_* ∈ {0, 1}, and in weighted networks, $w_{\textit{ij}}\in \mathbb {R}$; furthermore, in undirected networks, elements in the adjacency matrix are symmetric relative to the first diagonal (i.e., *w_ij_* = *w_ji_*).

The *multilayered* networks generalize the network concept by considering multiple types of edges/links so that the network can be described by several adjacency matrices *W*^*q*^ (*q* ∈ {1, 2, …*Q*}), with elements $w^{q}_{ij}$ for each of the *Q* types of links. Moreover, when acknowledging that the link structure (i.e., the network topology) changes over time *t*, the network is expressed as a function of time, $w^{q}_{ij}\left( t\right)$.

### Drug networks

To mine the complexity of drug interactions in the biological environment, researchers use database information to build drug networks such as drug–drug, drug–target, and drug–disease interaction networks, as well as drug–disease and drug–adverse effects association networks. This article considers drug–drug interaction networks (complex drug networks where nodes represent drugs) and drug–target interaction networks (bipartite networks where one type of node represents drugs and the other represents biological targets). Links represent drug–drug and drug–target interactions in the DDI and DTI networks. We do not consider drug–drug interaction type and strength. Consequently, the DDI networks are undirected, unweighted, and monopartite. Also, the drug–target interactions have no information regarding their strength, making DTI networks directed (from drug to target), unweighted, and bipartite.

Using the complex network formalism, in DDI networks, ∀*i, j, i* ≠ *j*, $v_i\in V=\mathcal {D}$ and $e_{ij}\in E=\mathcal {I}$ (*e_ij_* ≠ 0), where $\mathcal {D}$ is the set of drugs and $\mathcal {I}$ the set of drug–drug interactions. In DTI networks, we have $v_i\in V = V_{\mathcal {D}}\cup V_{\mathcal {T}}$ (where $V_{\mathcal {D}}$ is the set of nodes representing drugs and $V_{\mathcal {T}}$ is the set of nodes representing targets) and $e_{ij}\in E=\mathcal {A}$ (where $\mathcal {A} =\left\lbrace e_{ij}\vert v_i\in V_{\mathcal {D}}, v_j\in V_{\mathcal {T}}, i\ne j, e_{ij}\ne 0\right\rbrace$ represents the set of drug–target interactions). Additionally, the set of nonedges in the DDI networks is $\bar{\mathcal {I}} = \left\lbrace e_{ij}\vert v_i, v_j\in \mathcal {D}, i\ne j, e_{ij}=0\right\rbrace$, and in the DTI, it is $\bar{\mathcal {A}} = \left\lbrace e_{ij}\vert v_i\in \mathcal {D}, v_j\in \mathcal {T}, i\ne j, e_{ij}=0\right\rbrace$.

A nonedge in a DDI network ($\bar{e}_{ij}\in \bar{\mathcal {I}}$) or a DTI network ($\in \bar{\mathcal {A}}$) represents a drug–drug or a drug–target interaction that has a probability *p* ∈ (0, 1) of being discovered in the future (i.e., missing information generating uncertainty). If *p* = 0, we are sure that interaction between *v_i_* and *v_j_* is impossible (i.e., the negative information on drug interactions).

The DDI and DTI networks are analyzed and processed with computational tools to acquire new insight and knowledge in areas such as drug repurposing, drug–drug interaction prediction, drug–target interaction prediction, designing synergistic drug combinations, and optimizing drug efficacy.

As this article considers the main types of drug networks—namely, drug–drug and drug–target interaction networks (or interactomes)—we acknowledge 2 main issues:

1. The networks are built according to what we know (in terms of drug–drug or drug–target interactions) at the time when the database is recorded. Over time, some new information may be added, and some may be filtered according to the experimental biological findings.
2. The uncertainty in building the drug networks, mainly represented by missing links between nodes—drug–drug or drug–target interactions that exist but are still uncovered—may affect the conclusions of drug network analysis methods. Previous research also expresses this important concern [27].

Accordingly, our investigation is set to answer 2 fundamental questions.

1. We already know the main topological metrics statistics in complex networks (biological, social, or technological) where the degree of uncertainty is smaller than in drug networks [46]. Thus, how do the drug network parameters evolve with the drug database versions compared to the typical complex network topological characteristics, such that they foster an effective and efficient computational complex network analysis?
2. Newer drug databases contain more information on both drug–drug and drug–target interactions. How did the robustness of the drug networks evolve after adding more knowledge?

To address question 1, we analyze the evolution over time of drug–drug and drug–target networks *w_ij_*(*t*) (where *t* are discrete-time moments when the drug database version was released) for parameters such as average degree, diameter, average path length, and average clustering coefficient. We analyze the same type of evolution for the distributions of centrality metrics such as degree, betweenness, closeness, eccentricity, and page rank.

To answer question 2, we implement a robustness test algorithm that adds/injects links representing potential unknown interactions to the known network, *w_ij_*(*t*) + *u_ij_* (where *u_ij_* represents the unknown links), and then represent the evolution of the ordinal correlation (Kendall τ) between the node rankings in *w_ij_*(*t*) and *w_ij_*(*t*) + *u_ij_* with the rate of unknown links (expressed as the number of nodes in *u_ij_* divided by the number of nodes in *w_ij_*(*t*)). The rationale for our approach is that the analysis of node centrality rankings underpins most network-based bioinformatics methods and applications.

## Methods

In this section, we describe the statistical and computational methods employed in this article: the “Network analysis” subsection introduces the main metrics and centralities required for the computational and statistical analysis of complex drug networks, the “Database quality” subsection presents the statistical methods we use to test the drug database integrity, the “Network analysis robustness” subsection explains the computer simulation method we propose to assess the robustness of the centrality-based drug network analysis strategies, and the “Computational methods” subsection describes the tools we use in this article to implement the required computational and statistical techniques.

### Network analysis

#### Network parameters and metrics

The *degree* of a node *v_i_* ∈ *V* in an undirected network *G* is defined as *d*(*v_i_*) = ∑*w_ij_*. In directed networks, we can compute the in-degree and out-degree of *v_i_* as *d*^*I*^(*v_i_*) = ∑*w_ij_* and *d*^*O*^(*v_i_*) = ∑*w_ji_*, respectively. Then, the average degree of network *G* = (*V, E*) is


(1) \begin{eqnarray*}
\langle d\rangle =\frac{1}{\vert V\vert }\sum _{v_i\in V} d\left( v_i\right) \end{eqnarray*}

where |*V*| is the number of elements in *V*—namely, the number of nodes in network *G*. If *G* is directed, we can compute the average in-degree and out-degree as $\langle d^I\rangle =\frac{1}{\vert V\vert }\sum _{v_i\in V} d^I\left( v_i\right)$ and $\langle d^O\rangle =\frac{1}{\vert V\vert }\sum _{v_i\in V} d^O\left( v_i\right)$. When the network *G* is multipartite, we can also compute the average degree for each node type *V_j_* (*V_j_* ⊂*V*) as $\langle d_{V_j}\rangle =\frac{1}{\vert V_j\vert }\sum _{v_i\in V_j} d\left( v_i\right)$.

The *clustering coefficient* of a node *v_i_* is the number of existing links between nodes directly connected to *v_i_* divided by the total number of possible links,


(2) \begin{eqnarray*}
c\left( v_i\right) = \frac{2\left|\left\lbrace e_{jk}\vert j,k\in L_i\right\rbrace \right|}{\vert L_i\vert \left(\vert L_i\vert -1\right)}, \end{eqnarray*}

with *L_i_* representing the set of nodes directly linked to *v_i_*. The average clustering coefficient of network *G* is


(3) \begin{eqnarray*}
\langle c\rangle = \frac{1}{\vert V\vert } \sum _{v_i\in V} c\left( v_i\right). \end{eqnarray*}

The *network density* is the ratio between the number of links/edges in *G* (i.e., |*E*|) and the maximum number of possible links,


(4) \begin{eqnarray*}
r = \frac{2\vert E\vert }{\vert V\vert \left(\vert V\vert -1\right)}. \end{eqnarray*}

The network *G* is a *connected graph* if there is a path between any 2 nodes *v_i_, v_j_* ∈ *V*; otherwise, *G* has multiple components (a component is a connected subgraph). In general, there can be many paths between 2 nodes *v_i_* and *v_j_* in a connected network or component; we denote the length of the shortest one *s*(*v_i_, v_j_*). Then, the average path length in *G* is


(5) \begin{eqnarray*}
\langle s\rangle = \frac{2}{\vert V\vert \left(\vert V\vert -1\right)}\sum _{v_i,v_j\in V}s( v_i,v_j). \end{eqnarray*}

The *diameter* of a network *G* is the biggest shortest path between any 2 nodes *v_i_, v_j_* ∈ *V*,


(6) \begin{eqnarray*}
\phi = \max _{v_i, v_j\in V}\left\lbrace s(v_i,v_j)\right\rbrace . \end{eqnarray*}

#### Network centralities

A *node centrality*  $\mathcal {C}$ is an attribute or metric that characterizes the importance of a node in the network; many studies in biological networks use centralities to rank nodes. The simplest centrality of node *v_i_* is the *degree d*(*v_i_*), as the number of links (or the weight) associated with the node indicates its importance [48–50].

The *betweenness* centrality characterizes the node’s role in connecting communities or clusters of nodes. (Such node clusters are often associated with specific functionality in biological networks, particularly drug networks [25, 35, 51, 52].) The betweenness of *v_i_* is the number of paths (shortest or random walks) between all node pairs in *G* that cross *v_i_* (normalized by the total number of node pairs in *G*),


(7) \begin{eqnarray*}
b\left( v_i\right) = \sum _{v_j, v_k \in V; \: i, j \ne k} \frac{2 \sigma _{j,k}\left( v_i\right)}{\vert V\vert \left( \vert V\vert -1\right)}
\end{eqnarray*}

where


(8) \begin{eqnarray*}
\sigma _{j,k}\left( v_i\right) = \left\lbrace \begin{array}{@{}l@{\quad }l@{}}1 & \text{if $\exists \: s(v_j, v_k)$ that crosses $v_i$}\\ 0 & \text{otherwise .} \end{array}\right. \end{eqnarray*}

The *closeness* centrality measures how close the node *v_i_* is to the other nodes; it is the inverse of the sum of shortest paths to all other nodes in *V*,


(9) \begin{eqnarray*}
\gamma \left( v_i\right) ={\left( \sum _{v_j\in V\setminus \left\lbrace v_i\right\rbrace } s(v_i, v_j)\right)}^{-1}. \end{eqnarray*}

The *eigenvector* centrality assumes that the degree of a node *v_i_* does not particularly determine its importance; instead, the importance of nodes directly connected to *v_i_* is key. For node *v_i_*, the eigenvector centrality value η(*v_i_*) is defined as


(10) \begin{eqnarray*}
{ \eta \left( v_i\right) = \frac{1}{\lambda }\sum _{v_j\in L_i}\eta (v_j) = \frac{1}{\lambda }\sum _{v_j\in V}\eta (v_j) w_{ij}}
\end{eqnarray*}

where λ is a constant, *w_ij_* is an element in the adjacency matrix *W*, and η(*v_i_*) is the eigenvector centrality value of nodes *v_j_* directly connected to *v_i_*. Because the definition in equation 10 is recursive, finding the eigenvector centrality values for all nodes in *V* requires solving the equation $W\boldsymbol {\eta } =\lambda \boldsymbol {\eta }$ using linear algebra [53]. (*W* is the adjacency matrix, and $\boldsymbol {\eta }$ is the vector of eigenvector centrality values for all nodes in *V*.)

#### Typical complex network characteristics

Generally, in real-world complex networks, the density of links is low (i.e., $\sim \frac{1}{n}$ because the number of links is of the order *n*, where *n* is the number of nodes and far from the order *n*^2^ corresponding to a completely connected network); the total number of links also determines the average degree, and the average degree is significantly lower than *n*; the degree follows either a power-law (exponent is between 1 and 3) or normal distribution; the average path length is between 3 and 10; and the clustering coefficient is larger than $\frac{1}{n}$ but considerably lower than 1 [46, 54].

### Database quality

The drug–drug and drug–target data quality in databases such as DrugBank are degraded by uncertainty, as they contain curated information from published studies, papers, and clinical trials. The ever-growing volume of empirical results that underpin drug databases can be affected by errors generated by improper data handling, academic misconduct, or imbalanced research focus (e.g., one may expect an abundance of new data on SARS-CoV-2 target data but less so for rare diseases). These problems related to data quality in drug databases are adequately acknowledged in the literature, along with the systematic countermeasures [27, 55]. One such analytical approach to validate data quality is checking various parameter distributions against Benford’s law [55]. The law of the first digit—or Benford’s law—states that the natural distribution of the first digit *f* of a real-world variable (covering a wide range of values) is


(11) \begin{eqnarray*}
P\left( f\right) = \log _{10} \left( 1+ \frac{1}{f}\right), \end{eqnarray*}

where *f* ∈ {1, 2, …9}. This means that the probability of first digit being 1 is the highest, with the probabilities of the following digit probabilities decreasing logarithmically.

In this article, we check Benford’s law for the network centralities to verify data quality, meaning that we consider *f* the first digit of network centralities $\mathcal {C}$. For complex network centralities, a similar approach is described in [56], in the case of social networks. To the best of our knowledge, no similar study was performed in biological networks, although we find proven applications of Benford’s law in systems biology [57, 58].

### Network analysis robustness

The drug–drug and drug–target interactions we take from DrugBank are well documented and proven by experiment, but we can assume some unknown interactions are yet to be investigated or tested. To analyze the robustness of network analysis tools, we draw inspiration from the paper [27], where the authors added to the drug–target network generated with DrugBank new drug–target interactions (i.e., unaccounted by DrugBank) reported in another database; this way, they analyze how the centrality distributions change with the added information. In this article, we adopt a systematic approach to study the robustness of both the network structure and the specific centralities by randomly adding unknown links (i.e., drug–drug or drug–target interactions) with a rate *q* and then analyze the structural changes that manifest through modifications in the node rankings. We measure the structural changes incurred by adding the previously unknown links in 2 ways. First, we target the centralities with a power-law distribution (e.g., degree, betweenness) to compute the difference between the log–log distribution slopes α. Second, we compute the Kendall τ correlation between the node rankings according to centrality $\mathcal {C}$ before and after adding the unknown links (τ = 1 indicates a perfect monotonous correlation, τ = 0 indicates no monotonous correlation).

By representing τ as a function of *q* (with *q* going from a very small value to 0.1), we can analyze the robustness of the network structure: rapid decay of τ as *q* increases indicates that the node ranking according to $\mathcal {C}$ is fragile to the uncertainty of new links being present; conversely, slow decay of τ suggests a robust node ranking. We present the algorithmic description of our robustness test in algorithm 1, where *E_t_* represents the set of all possible edges in *G*, and *E_u_* is the set of previously unknown edges (accordingly, *E_t_*∖*E* is the set of nonexisting edges in *G*). As this is a random simulation, we repeat it for each *G* and *q* (*R* times in algorithm 1) to compute the average and variance for α and Kendall τ. (For the simulations presented in this article, we used *R* = 100.)

**Algorithm 1:**
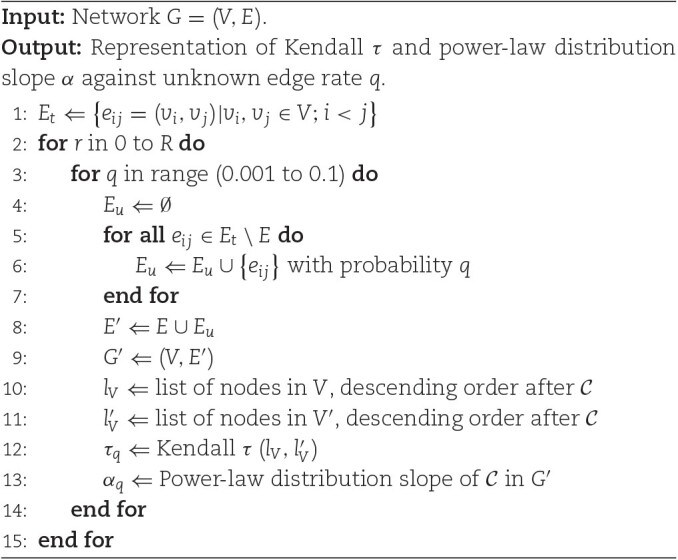
Analyze the variation of Kendall τ between node rankings and power-law distribution slope α (for node centrality $\mathcal{C}$) in network *G* and network with unknown edges *G*′.

### Computational methods

To foster the reproducibility of our analysis, we provide all the necessary tools—Jupyter Notebook, Python, and WolframScript—as buildable Docker containers in the https://github.com/research-hyperion/Drug_database_statistics repository. We automatically install all the tools needed for reproducing our methods in a Linux container and provide a shell script for automatically running the analysis. The activation of WolframScript for running the Mathematica notebooks requires a Wolfram account. DrugBank versions recorded as XML files must be downloaded, as mentioned in the “Data Description” section, in the root of the cloned repository in the DrugBank directory. Each XML file must be saved following a naming convention to avoid file overwriting; therefore, each DrugBank XML file is saved as *drugbank_version.xml* in the DrugBank directory.

To create the Docker image, run the command **docker build –build-arg wolframId=<wolfram account email> –build-arg wolframPass=<wolfram account password> -t hyperion**. The image will take several GB of storage. The *wolframId* and the the *wolframPass* are the Wolfram account credentials needed to activate the WolframScript. After creating the container, run the command **docker run -t -i hyperion /bin/bash** in the terminal window to start an interactive shell in the Docker container.

We created a shell script that allows the execution of the Jupyter notebooks that build the DDI and the DTI networks and the network robustness analysis for each DrugBank version. As presented in the “DTI network robustness” subsection, due to the high complexity of the robustness analysis and huge computational burden, the script must provide the options to build and analyze the DDI and DTI networks or to perform the network robustness analysis. Invoking the script with the “-i” parameter will build and analyze DDI and DTI networks for each DrugBank version; invoking with the “-s” parameter will build the networks and run the robustness analysis.

The DDI and DTI network analyses are performed by 3 Jupyter Notebooks—namely, *parse_DrugBank, parse_DrugBank-DDI*, and *parse_DrugBank-DTI*; if run in the mentioned order, we parse the DrugBank XML files and build and analyze the DDI and DTI networks. The robustness analysis depends on the files created by the mentioned notebooks to perform the algorithmic analysis presented in the “Network analysis robustness” subsection.

## Results

This section analyzes 2 types of networks built from DrugBank data: DDI networks (where nodes *v_i_* represent drugs and links represent drug–drug interactions) and DTI networks (nodes represent both drugs and targets and links represent drug–target interactions).

Many studies that employ complex network science for drug repurposing, drug interaction prediction, or adverse effect prognosis use other, more sophisticated drug networks [1, 12, 13, 33, 59] (multipartite networks, drug similarity networks, etc.) However, the straightforward DDI and DTI topologies are representative of the complexity issues at hand, and most of the more elaborated networks (such as the weighted similarity networks) can be derived from DDI and DTI structures through multipartite network projection [60].

### Network metrics and centrality analysis

Our analysis follows the evolution of metrics and centralities in DDI and DTI networks built with DrugBank information—from version 3.0 to version 5.1.8 (January 2011–January 2021).

**Figure 3:**
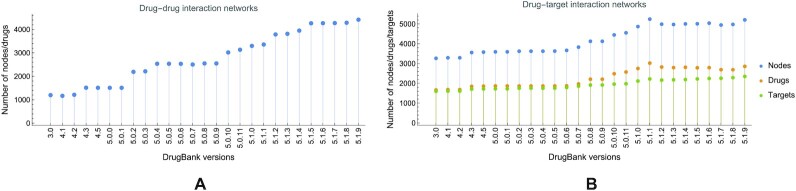
The evolution of nodes |*V*| in drug–drug (representing drugs $\vert V_{\mathcal {D}}\vert$, A) and drug–target interaction networks (representing drugs $\vert V_{\mathcal {D}}\vert$ and targets $\vert V_{\mathcal {T}}\vert$, B) built with information from DrugBank versions 3.0 to 5.1.9. The number of drugs in the DDI evolves from 1,198 for version 3.0 to 4,417 for version 5.1.9; the increase is steady, although some DrugBank versions abruptly put on more drugs so that several ensuing versions filter the added information. The number of drugs and targets in the DTI evolves respectively from 1,166 and 1,599 in version 3.0 to 2,857 and 2,350 in version 5.1.9; because the DTI network is bipartite, the number of DTI nodes is the sum of drugs and targets.

In Fig. 3A, we present the evolution of the number of nodes (i.e., drugs) in DDI networks; in Fig. 3B, we show the evolution of the number of nodes, drugs, and targets in DTI networks. As shown, from versions 3.0 to 5.1.9, the number of medicines/drugs roughly quadrupled; in contrast, the number of targets increased at a much lower rate. Such an evolution suggests that, over the years, the complexity of processing and analyzing drug interaction networks grew considerably. In both DDI and DTI networks, the number of drugs and targets is not the same as the total number of drugs and targets in the respective DrugBank versions because some drugs and targets have no known interactions.

**Figure 4:**
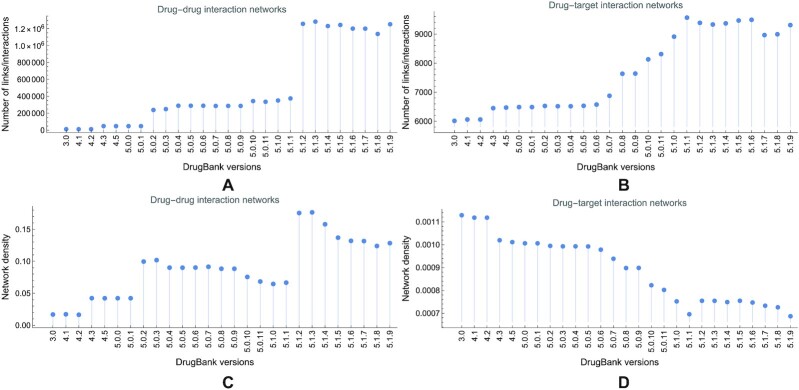
The number of links |*E*| evolution in drug–drug and drug–target interaction networks, built with information from DrugBank versions 3.0 to 5.1.9 (A and B, respectively). The number of links in the DDI evolves from 12,089 in version 3.0 to 1,252,028 in version 5.1.9; in the DTI, it increases from 6,015 to 9,310. Panels C and D respectively present the evolution of density *r* in drug–drug and drug–target interaction networks, built with information from DrugBank versions 3.0 to 5.1.9. The density in the DDI networks evolves from 0.0186 in version 3.0 to 0.128377 in version 5.1.9; some versions abruptly increase the density (by adding many interactions), while the following versions filter the interactions and decrease the density (e.g., the density evolution from 5.1.1 to 5.1.9). In the DTI networks, the density evolves from 0.0011 in version 3.0 to 0.000687 in version 5.1.9; as shown, the density in DTI networks decreases with the newer database versions.

We also notice the significant discrepancy between the number of links in DDI and DTI networks (Fig. 4A and B, respectively), which determine the evolution of DDI and DTI density evolution in Fig. 4C and D.

**Figure 5:**
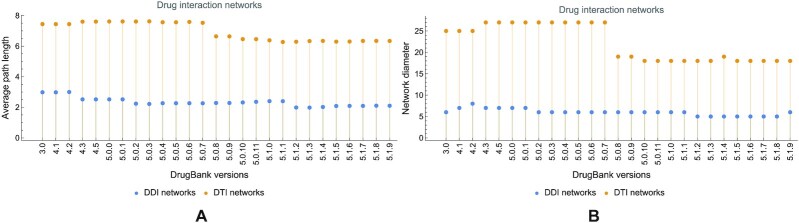
The evolution of average path length 〈*s*〉 (A) and diameter ϕ (B) in drug–drug and drug–target interaction networks, built with information from DrugBank versions 3.0 to 5.1.9. The value of 〈*s*〉 evolves from 2.98 in version 3.0 to 2.1 in version 5.1.9. for DDI and from 7.44 to 6.338 in DTI. The value of ϕ evolves from 6 in version 3.0 to 6 in version 5.1.9. for DDI and from 25 to 18 in DTI.

**Figure 6:**
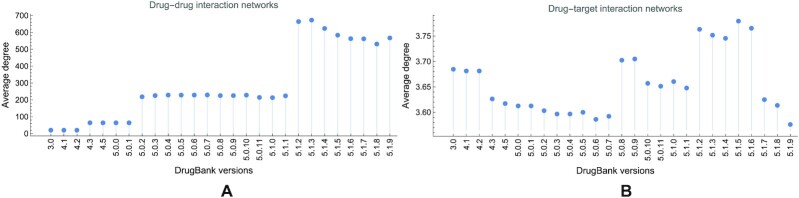
The evolution of average degree 〈*d*〉 in (A) drug–drug and (B) drug–target interaction networks, built with information from DrugBank versions 3.0 to 5.1.9. For DDI networks, the value of 〈*s*〉 increases from 20.181 in version 3.0 to 566.913 in version 5.1.9; for DTI networks, it oscillates from 3.684 to 3.576.

Comparing the average path length and diameter in the DDI and DTI networks further emphasizes the observed discrepancies (see Fig. 5A, B); the same remark holds for the average degree in Fig. 6 (panels A and B for DDI and DTI, respectively).

**Figure 7:**
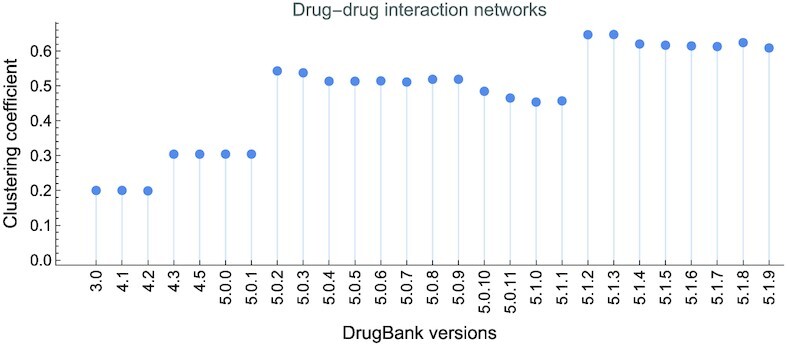
The evolution of average clustering coefficient 〈*c*〉 in drug–drug interaction networks, built with information from DrugBank versions 3.0 to 5.1.9. The value of 〈*c*〉 increases from 0.199 in version 3.0 to 0.609 in version 5.1.9.

The DTI networks are bipartite; therefore, any link/edge connects 1 drug with 1 target. Consequently, DTI networks have a clustering coefficient of 0. Fig. 7 presents the evolution of the average clustering coefficient 〈*c*〉 in DDI networks.

**Figure 8:**
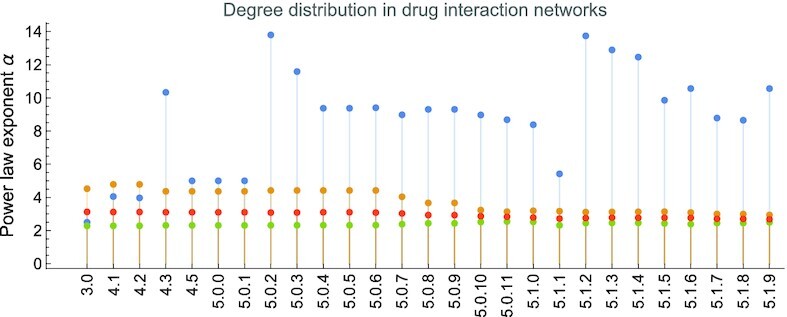
The evolution of the α exponent of the power-law degree distribution in drug–drug and drug–target interaction networks, built with information from DrugBank versions 3.0 to 5.1.9. In DDI networks, the α values are very high—specific for dense networks. In DTI networks, we present the overall degree distribution of the nodes and the in-degree and out-degree distributions, as the DTI network is bipartite and directed. (The connected nodes representing drugs have nonzero out-degrees and zero in-degrees, whereas nodes representing targets have zero out-degrees and nonzero in-degrees.)

Like many other natural complex networks [46,61], the DDI and DTI networks are scale-free, meaning that their node degree distribution is a power-law *P*(*d*)∝*d*^−α^. Fig. 8 presents the evolution of node degree’s power-law distribution exponent α in DDI and DTI networks. (DTI is a bipartite directed network, and we also present the in-degree and out-degree distributions for targets and drugs, respectively.)

The analytical results presented in this section indicate that the DDI networks have become highly dense (*r* = 0.1238 with a huge 〈*d*〉 = 530.8455 for DrugBank 5.1.8), with small 〈*s*〉 and ϕ and an unusually large α. Although the clustering coefficient 〈*c*〉 is high, detecting node communities or ranking nodes with centralities—standard network analysis techniques in network pharmacology [12, 14, 25]—becomes irrelevant because of the high link density. Conversely, the metrics and centrality statistics of the DTI networks are typical for biological scale-free complex networks [46, 54]: small 〈*s*〉 (but not smaller than 6) and an exponent α around 3. Therefore, the DTI network topologies foster the employment of community detection and other specific network analysis techniques.

**Figure 9:**
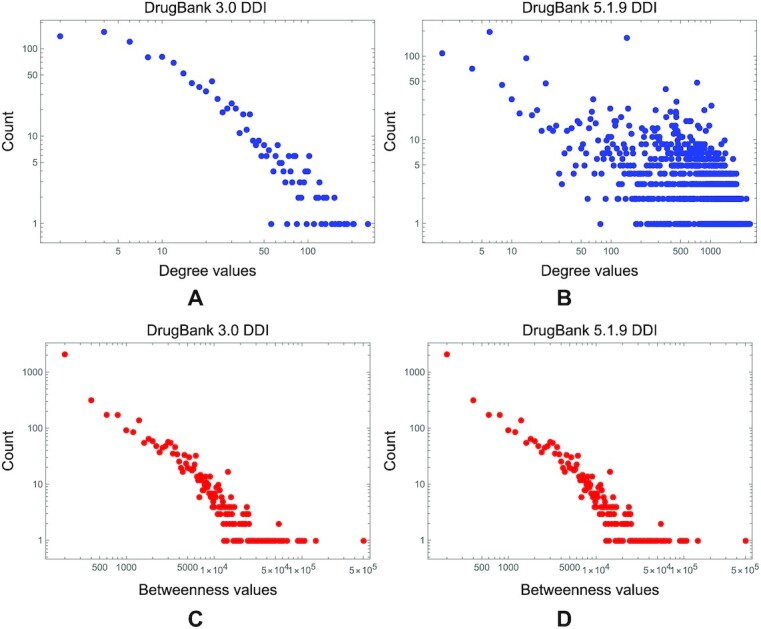
The degree and betweenness distributions in the DDI networks, built with information from the first and latest DrugBank versions. (A, C) The degree and betweenness distributions in the DrugBank 3.0 DDI network. (B, D) The DrugBank 5.1.9 counterparts.

Moreover, upon visual inspection, Fig. 8 indicates that as the DDI networks become denser across the DrugBank versions, their degree exponent α becomes >4 (i.e., too big to correspond to real-world power-law distributions) [46]. Indeed, the visual representation of the degree and betweenness distributions in the DrugBank 3.0 DDI network shows typical power-laws (Fig. 9A, C), whereas the DrugBank 5.1.9 counterparts indicate that only the betweenness distribution is not substantially altered (Fig. 9B, D). We analyzed and calculated these distributions with the Powerlaw Python package [62].

**Figure 10:**
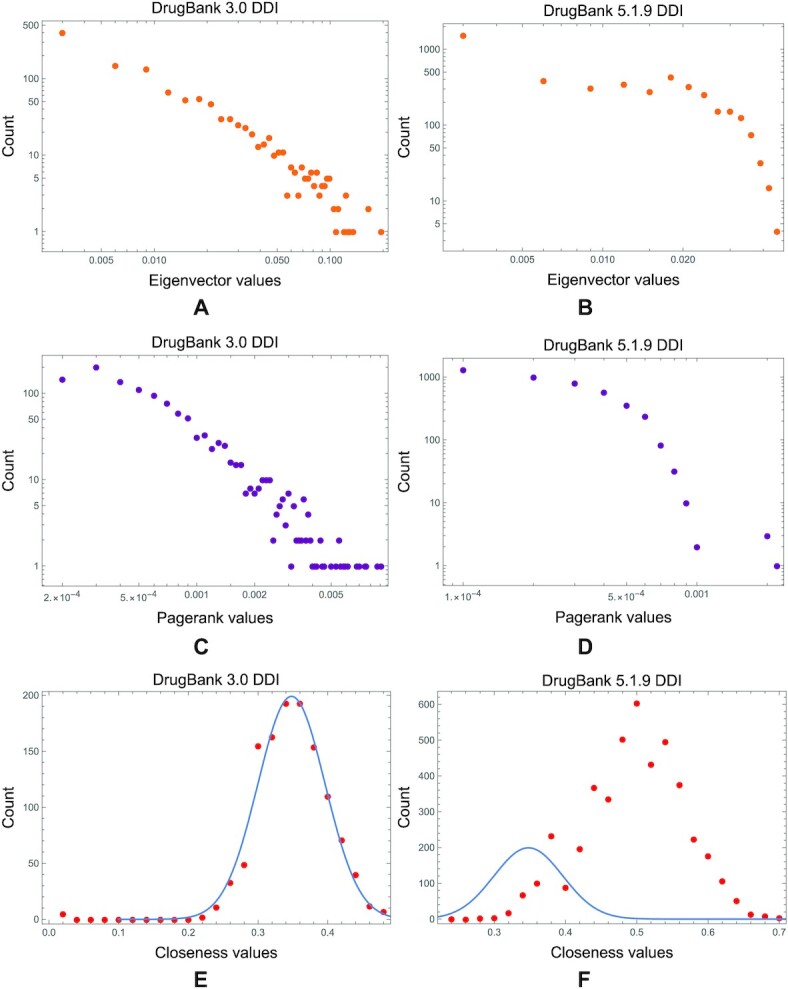
The comparison between eigenvector, PageRank, and closeness distributions in DDI networks built with information from DrugBank 3.0 and 5.1.9. (A, B) The power-law distribution of eigenvector centrality from the DrugBank 3.0 DDI degrades in the latest versions. (C, D) The same can be observed for the PageRank distributions. We also notice that the normal distribution of closeness (a common feature of real-world complex networks, particularly drug interaction networks [35]) in the DrugBank 3.0 DDI network degrades for the DrugBank 5.1.9 DDI network, as shown with the distribution fitting blue line in panels E and F. (We performed the distribution fitting in Mathematica 13.).

We observe the same degradation for the other centrality distributions in DDI networks from the earlier versions of the drug database to the latest ones. In Fig. 10A–C, we present the eigenvector, PageRank, and closeness distributions in the DrugBank 3.0 DDI network; in Fig. 10D–F, we visualize these distributions in the DrugBank 5.1.9 DDI network.

**Figure 11:**
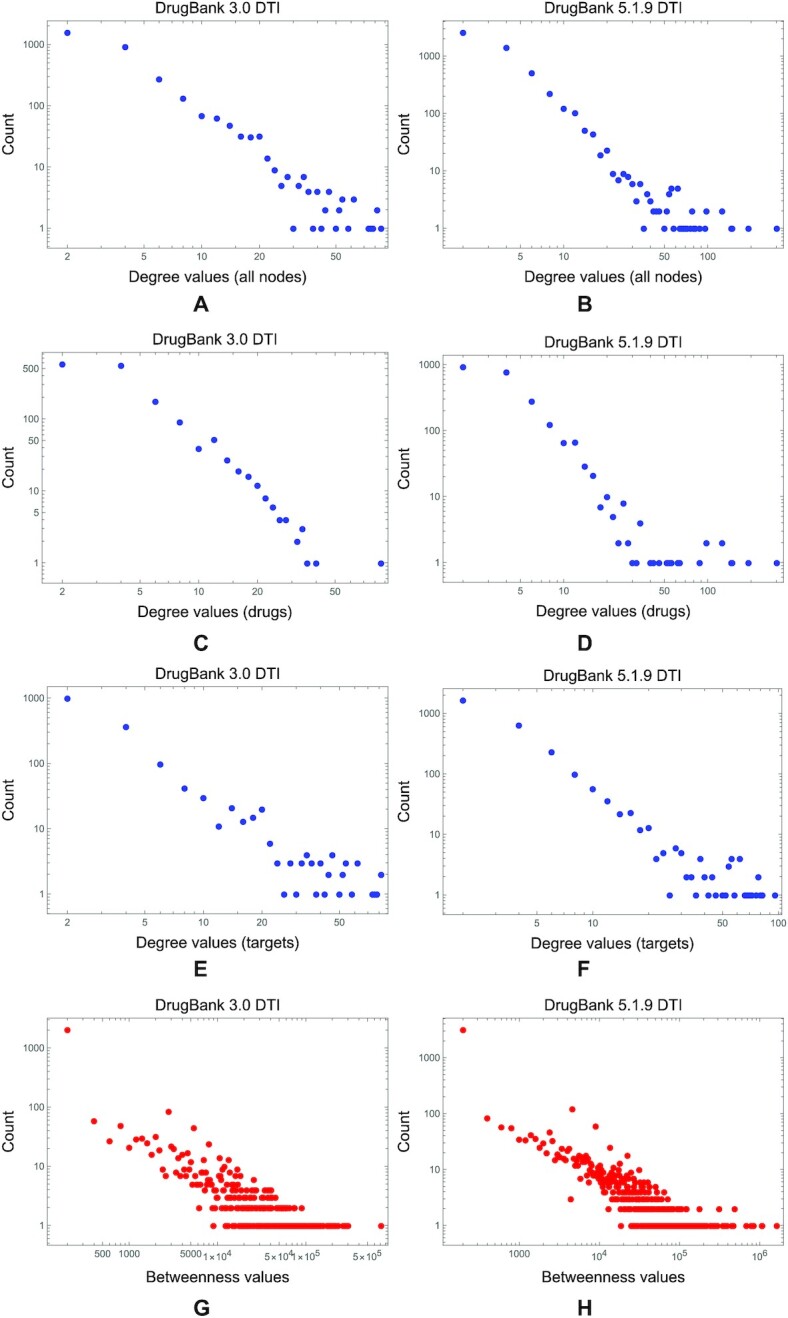
The degree and betweenness distributions in the DTI networks, built with information from the first and latest DrugBank versions. (A, C, E, G) The degree and betweenness distributions in the DrugBank 3.0 DTI network (we separately display the drug and target, as well as all nodes’ degree distributions). (B, D, F, H) DrugBank 5.1.9 counterparts.

**Figure 12:**
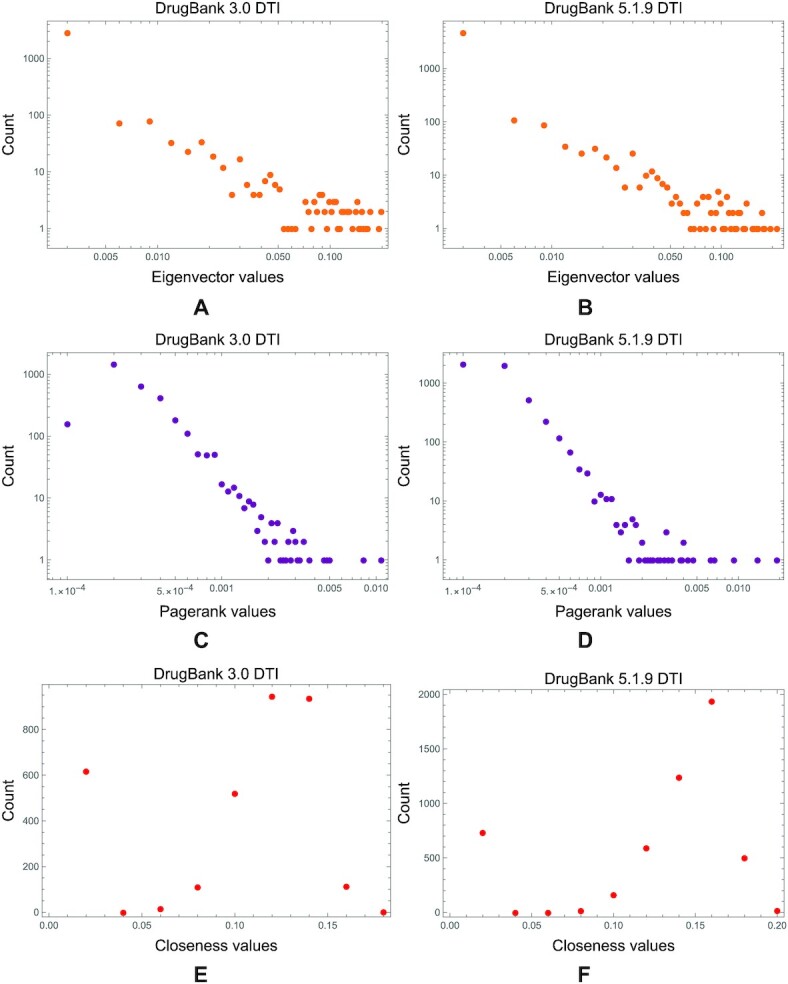
The comparison between eigenvector, PageRank, and closeness distributions in DTI networks with information from DrugBank 3.0 and 5.1.9. (A, B) The eigenvector distribution from the DrugBank 3.0 DTI does not degrade in the latest versions. (C, D) The same can be observed for the PageRank distributions. We also notice the nonregular distribution of closeness in both DrugBank 3.0 DTI and DrugBank 5.1.9 DTI networks in panels E and F. (We performed the distribution fitting in Mathematica 13.)

Conversely, in the DTI networks, we do not notice the same degradation of power-law degree and betweenness distributions from DrugBank 3.0 to 5.1.9 (see Fig. 11, where we analyzed and calculated these distributions with the Powerlaw Python package [62]). We see the same tendency not to alter the distributions in DTI networks across all DrugBank versions for eigenvector, PageRank, and closeness centralities (as presented in Fig. 12).

### Network centralities and Benford’s law

We check if the distributions of centralities abide by Benford’s law in drug–drug and drug–target networks. When data distribution in naturally occurring datasets closely resembles the Benford distribution, we assume a high drug interaction data quality, generating robust analysis results.

We use Pearson’s chi-squared (χ^2^) test to measure the distance between degree and betweenness centrality distributions and the theoretical Benford distribution in DDI networks across all DrugBank versions, as indicated in [56]. However, as argued in [63], Pearson’s χ^2^ is often misused in such analysis cases; therefore, as suggested in this reference, we also use the Wasserstein distance and the sum of squared deviations between distributions. For these 3 metrics, a smaller distance to Benford’s distribution means that the empirical centrality distribution in the drug network is more compliant with Benford’s law of the first digit. Apart from the numerical analysis, we also used graphical representations of the distributions, including Q–Q plots, to check if the degree and betweenness distributions in DDI networks abide by Benford’s law.

#### DDI networks

**Figure 13:**
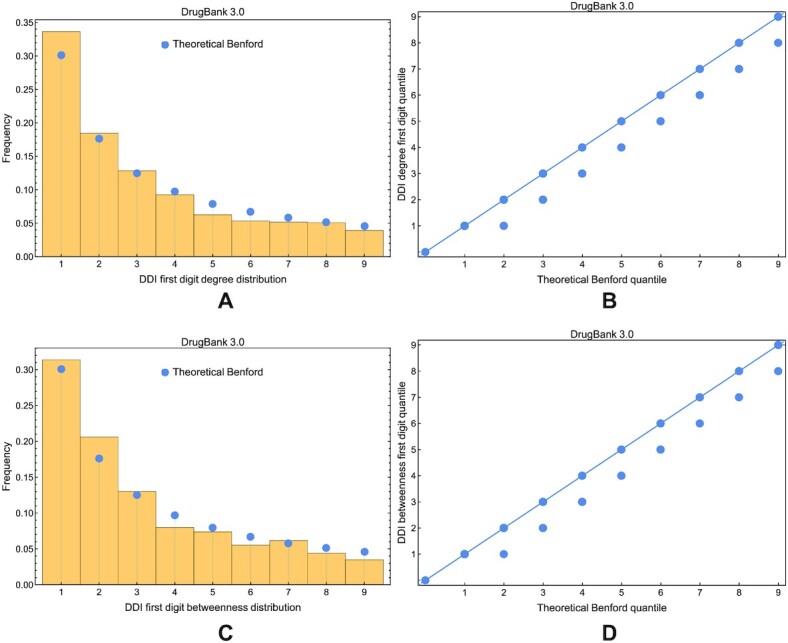
The compliance of degree and betweenness distributions in DDI networks built with DrugBank 3.0 data. (A) The comparison between the empirical distribution represented in the histogram and the theoretical Benford distribution represented with blue disks. (B) The Q–Q plot where the dashed trendline following the diagonal indicates a small distance to a theoretical Benford distribution. (C) The comparison between the empirical distribution and the theoretical Benford distribution. (D) The Q–Q plot where the dashed trendline also indicates a small distance to a theoretical Benford distribution.

**Figure 14:**
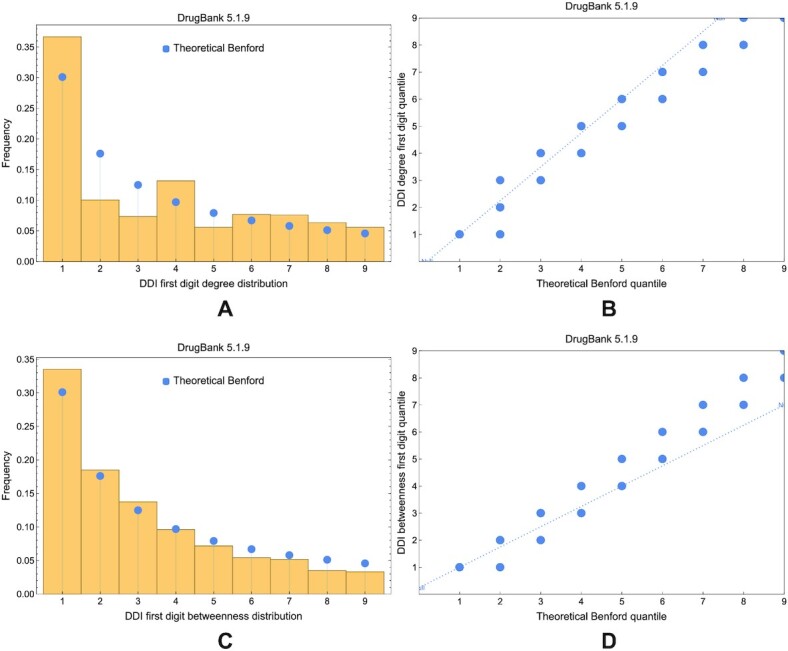
The compliance of degree distribution in DDI networks built with DrugBank 5.1.9 data. (A) The comparison between the empirical distribution represented in the histogram and the theoretical Benford distribution represented with blue disks. (B) The Q–Q plot where the dashed trendline does not follow the diagonal but indicates a significant distance to a theoretical Benford distribution. (C) The comparison between the empirical distribution and the theoretical Benford distribution. (D) The Q–Q plot indicating a close distance to a theoretical Benford distribution.

**Figure 15:**
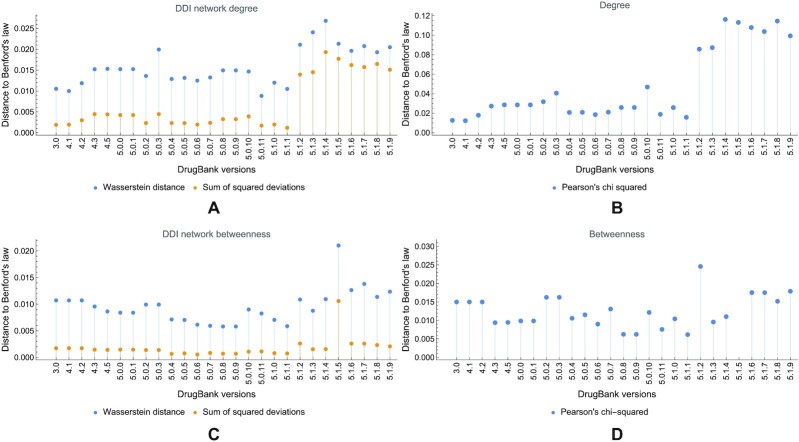
The evolution of distance between the Benford distribution and the empirical degree and betweenness distributions in DDI networks across DrugBank versions. (A) Wasserstein distance and sum of squared deviations for the degree centrality. (B) Pearson’s χ^2^ for the degree centrality. (C) Wasserstein distance and sum of squared deviations for the betweenness centrality. (D) Pearson’s χ^2^ for the betweenness centrality. Smaller values indicate a stronger compliance with Benford’s law of the first digit.

We notice by the visual inspection of Figs. 13 and 14 that the compliance with Benford’s law has degraded over the years (i.e., with the evolution of DrugBank versions) in DDI networks, especially for the degree distribution. Using the Wasserstein distance, the sum of squared deviations, and Pearson’s χ^2^, we show the evolution of distance between the theoretical Benford distribution and the empirical distribution of degree (Fig. 15A, B) and betweenness centrality (Fig. 15C, D) distributions in DDI networks across all DrugBank versions. (We represented Pearson’s χ^2^ separately because its range of values is larger than the other distance metrics.)

#### DTI networks

**Figure 16:**
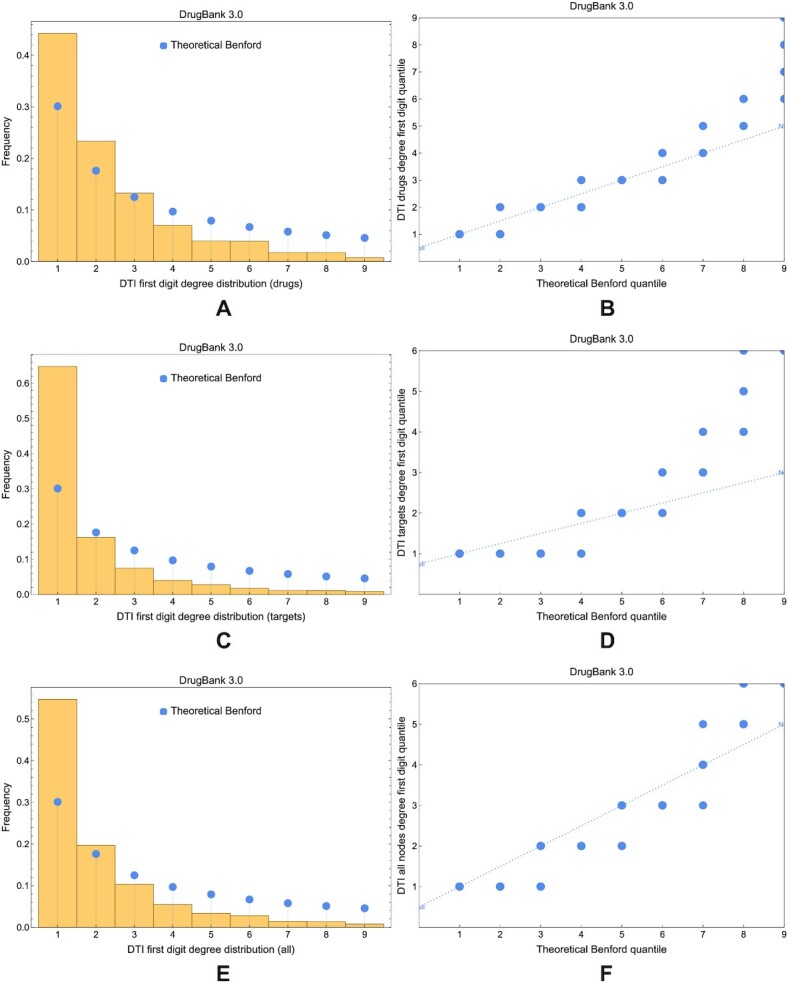
The compliance of degree distribution in DTI networks built with DrugBank 3.0 data. (A, C, E) Comparison of the theoretical Benford distribution with blue disks and the degree distribution in DTI network nodes representing drugs, targets, and all DTI network nodes. (B, D, F) The Q–Q plots corresponding to panels A, C, and E. All the results in this figure indicate noncompliance with the theoretical Benford distribution.

**Figure 17:**
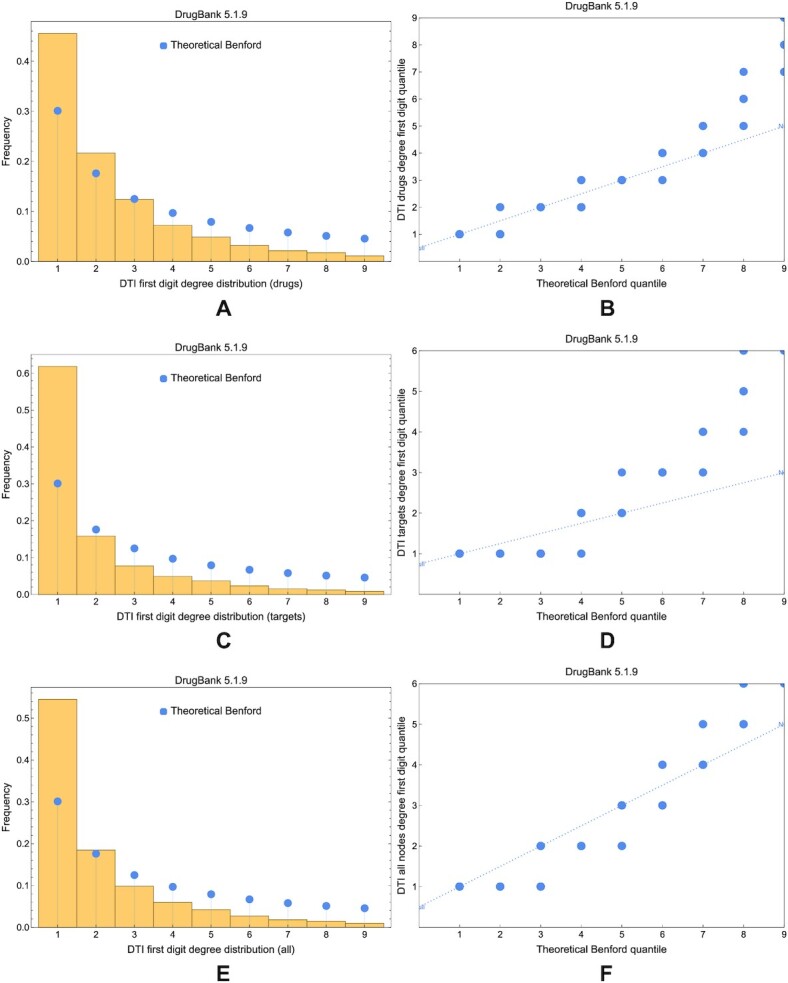
The compliance of degree distribution in DTI networks built with DrugBank 5.1.9 data (i.e., latest version). (A, C, E) Comparison of the theoretical Benford distribution with blue disks and the degree distribution in DTI network nodes representing drugs, targets, and all DTI network nodes. (B, D, F) The Q–Q plots corresponding to panels A, C, and E. All the results in this figure indicate noncompliance with the theoretical Benford distribution.

**Figure 18:**
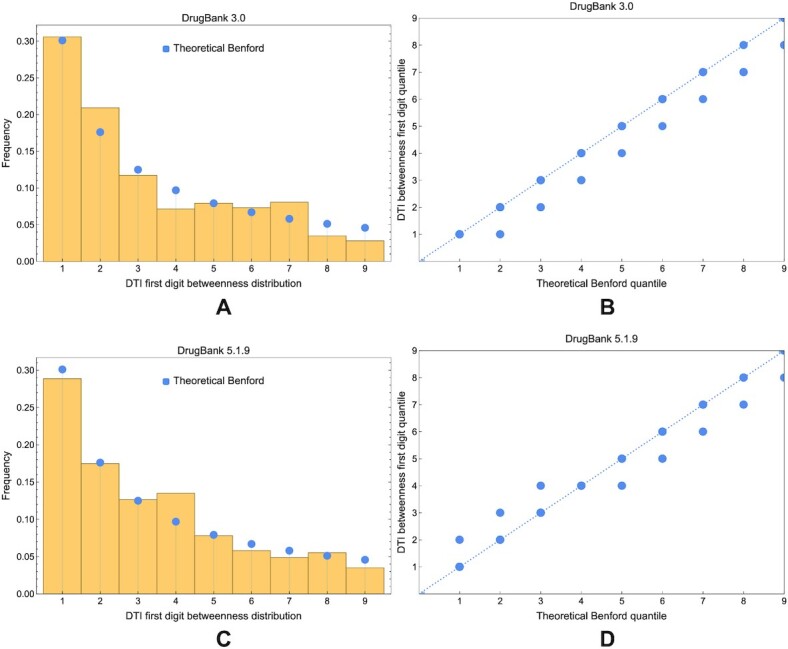
The compliance of betweenness distribution in DTI networks with Benford’s law. (A, C) Comparison of the theoretical Benford distribution (blue disks) with the empirical betweenness distribution in DTI network nodes built with DrugBank 3.0 and 5.1.9 data, respectively. (B, D) The Q–Q plots corresponding to panels A and C. All the results in this figure indicate a relatively good compliance with the theoretical Benford distribution.

For the degree, Figs. 16 and 17 offer a visual comparison between the first and the latest DrugBank versions; they show that the empirical degree is far from Benford’s theoretical distribution in all database versions. For the betweenness, Fig. 18 presents the comparison between the theoretical Benford first digit distribution and the betweenness in DrugBank 3.0 and 5.1.9 DTI networks.

The overarching conclusion in DTI networks is that the degree is not compliant with Benford’s law, but the betweenness is relatively compliant; these properties do not change across the DrugBank versions.

### DTI network robustness

The algorithmic evaluation of network robustness, described in the “Network analysis robustness” subsection, entails running *R* = 100 times the following steps sequentially: adding random edges to the drug network according to a rate of unknown interactions *q*, determining the degree and betweenness centralities of all nodes in networks *G* and *G*′ from algorithm 1, sorting vertices/nodes in *G* and *G*′ after their centralities, and calculating the Kendall τ between the node hierarchies in *G* and *G*′ after sorting the nodes in the descending order of their centrality values. The algorithmic complexity of adding random edges is $\mathcal {O}( n^2)$, where *n* represents the number of nodes of the network (the number of added edges is proportional to *n*^2^, and the complexity of generating random numbers is $\mathcal {O}\left( 1\right)$). Determining the degree centrality for all network nodes has a complexity of $\mathcal {O}( n^2)$; calculating the betweenness of a node with the Brandes algorithm has a complexity of $\mathcal {O}(nN + n^{2}\log n)$ (where *N* is the number of edges |*E*| in *G* = (*V, E*)) [64]; therefore, as many drug networks are dense and *N* ∼ *n*^2^, computing the betweenness of all nodes in *G* entails between $\mathcal {O}( n^4)$ and $\mathcal {O}( n^5)$ complexity. The complexity of sorting the nodes according to their centralities as well as calculating the Kendall τ is between $\mathcal {O}\left( n\log n\right)$ and $\mathcal {O}( n^2)$ [65]. In conclusion, performing the algorithmic evaluation of network robustness from the “Network analysis robustness” subsection entails a huge computational burden, especially when processing the high-density DDI networks and considering the betweenness centrality.

**Figure 19:**
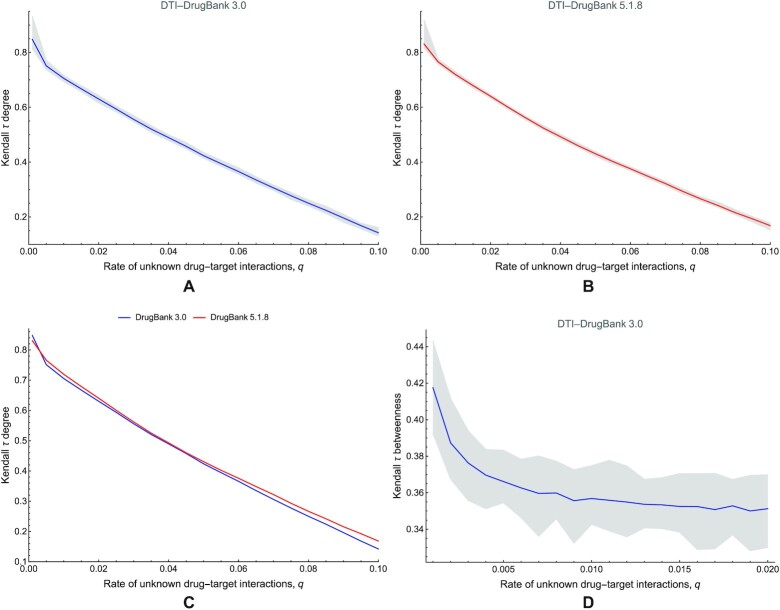
The evolution of Kendall τ (measuring the centrality-based ordinal correlation between the known DTI *G* and the DTI including unknown edges *G*′) with the rate (i.e., fraction) of unknown edges *q*. (A) The results for the DrugBank 3.0 DTI and the degree centrality, with the blue line following the average τ after 100 simulations and the gray area showing the variance. (B) The counterpart of panel A for DrugBank 5.1.8. (C) The comparison between DrugBank 3.0 and 5.1.8 DTI networks. (D) A robustness analysis similar to panel A but for the betweenness centrality.

Assuming the conclusion of the complexity considerations for algorithm 1, we will focus our robustness study on DTI networks and perform it for the betweenness centrality in the less dense DrugBank 3.0 DTI network. As presented in the simulation results (see Fig. 19A, B) of node hierarchy robustness according to the degree centrality, we notice in both DrugBank 3.0 and 5.1.8 DTI networks that the Kendall τ decreases linearly with the unknown edge rate *q* and only a slight increase for DrugBank 5.1.8 DTI in comparison with DrugBank 3.0 (Fig. 19C). After repeating all simulations 100 times for each *q*, we noticed a low variability in Fig. 19A and B.

As explained, due to algorithmic complexity reasons, we perform the betweenness centrality robustness test in algorithm 1 for the DrugBank 3.0 DTI; the corresponding simulation results in Fig. 19D reveal a logarithmic decrease with *q* but a high variability of τ.

**Figure 20:**
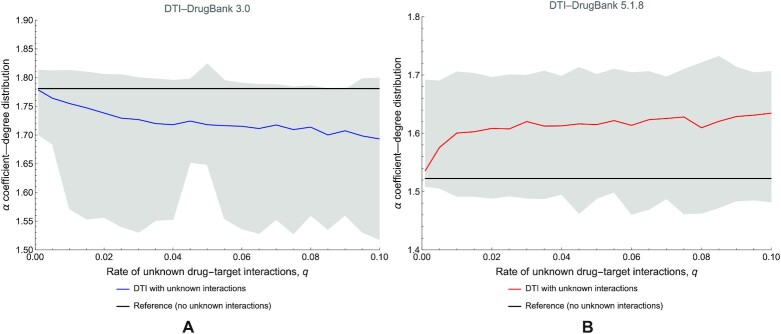
The evolution of the power-law distribution exponent α with the rate of unknown interactions *q*. (A) The case of the first DrugBank version 3.0 DTI network. (B) The case of the DrugBank version 5.1.8 DTI network. In the figure, the black lines represent the reference α values (i.e., corresponding to DTI networks with no unknown interactions, *q* = 0). We performed simulations 100 times for each *q* value, showing the average values with blue and red lines, while the gray area represents the interval between minimum and maximum values.

Because the analysis of the degree distribution under different rates of unknown interactions *q* is not computationally prohibitive in terms of complexity, according to algorithm 1, we analyze the power-law distribution parameters in DTI networks for DrugBank 3.0 and 5.1.8. We choose to focus our analysis on DTI—instead of DDI—because the DDI networks are very dense already, and adding unknown interactions does not have a significant impact. Fig. 20 comparatively presents the evolution of the power-law degree distribution exponent α with the rate of unknown interactions *q* in DrugBank 3.0 (Fig. 20A) and DrugBank 5.1.8 (Fig. 20B) DTI networks.

## Discussion

Our discussions mainly stress the drug datasets’ vulnerable aspects, as revealed by the analysis results, specifically those related to the DDI and DTI networks.

### Discussions on DDI networks

The first finding of our analysis is that the DDI networks built with data from the latest DrugBank versions deviate significantly from the typical complex network parameters and centrality distributions. Such a situation is generated by the high network density, owing to the colossal number of reported interactions. (The latest DDI network has a massive average degree of 650 and a very small average path length of around 2.) The problematic situation of the very dense DDI networks calls for addressing the following issues.

#### Some reliable methods for filtering out the irrelevant DDIs

Tilson et al. [66] highlighted that a continuous process is required to select DDIs for the relevant clinical alert and support the clinical decision; physicians and pharmacists need to filter the DDIs and find a way to develop and agree on a standard set of evidence-based DDIs. Indeed, medicines have a proven propensity toward drug–drug interactions in biological environments; therefore, there is a significant probability of interaction between any 2 drugs. However, only some of these interactions are relevant in specific circumstances.

#### Specifying rules/norms for recording the DDI severity

Experts promote the development of guidelines for drug–disease and drug–drug interactions in patients with multimorbidity. They suggest populational (i.e., big data) evidence to detect circumstances for relevant interactions [67]. Indeed, the different drug datasets often disagree on the severity level for the same DDI [68].

#### Establishing the clinical context and interactions’ relevance for each drug

The DDI severity depends on various clinical factors (e.g., a DDI may be major for some specific comorbidity or patient age and moderate/minor otherwise). Circumstances are paramount to analyzing the complexity of clinical practice and identifying potential DDIs and their attributes (i.e., type, severity, and frequency).

Clinicians’ perspectives on the potential DDI clinical relevance are essential to avoid alert fatigue—a severe problem affecting the electronic DDI alert systems that support modern health care procedures [69]. Consequently, Pirnejad et al. [70] recommend flexibility integrated into DDI clinical decision support systems to personalize potential DDI alerts.

Another approach proposes criteria for evaluating/scoring high-priority DDIs that provide clinical decision support alerts in electronic health records [71]. Kontsioti and collaborators [72] started from the assumption that the literature could be better in creating DDI reference sets or open resources that simultaneously aim at DDIs’ clinical relevance and interacting drugs’ behavior. They automatically extracted and ensembled data from multiple resources and provided a pipeline for generating a reference set for DDIs that help postmarketing drug surveillance.

Nonetheless, the problem is that the drug datasets do not record the clinical context associated with the DDI severity; this may explain why different drug databases report different severity levels for the same DDI—they may assume dissimilar clinical situations.

#### Building a negative dataset of DDIs with drug pairs known as noninteracting in specific clinical circumstances

To simplify clinical work, Assiri and colleagues [73] developed an anti-DDI resource for 200 drugs—a set of drug combinations with negative reported interactions (i.e., no risk of DDI).

However, negative DDI information is scarce, and looking for such data means navigating a vast search space; therefore, developing computer-based tools for predicting noninteracting drug pairs would be highly beneficial [74].

### Discussions on DTI networks

Our analysis results for DTI networks suggest that the data available across all drug database versions miss much information on drug–target interactions. Also, our simulations indicate that the robustness of centrality-based network analysis methods improves (slightly) with the newer database versions (see Fig. 19); however, these latest versions’ degree distribution is much more stable (Fig. 20). The DTIs do not have the high-density problem of DDI networks and the entailed consequences. The DTI network results indicate the need to address the following issues.

#### Accurate methods for DTI predictions

We need more accurate drug–target interaction prediction tools, particularly for the DTIs involving new targets (e.g., introduced each new year by the FDA [42]). Such computational methods will help biologists prune the enormous drug–target interaction search space and focus on the most promising and potentially impactful experiments.

Computational DTI prediction methods evolved as a convenient alternative (or complement) to the conventional methods for discovering new drugs, repositioning drugs, or uncovering potential drug side effects. The DTI prediction methods are diverse: multimolecular networks based on deep walk embedding model [75]; knowledge graph embedding model–neural factorization machine unified framework [76]; convolutional neural networks using only data on drug structure and protein sequence [77]; heterogeneous network-based methods that integrate various drug data [8]; combined computational techniques such as graph embeddings, graph mining, and machine learning [10]; or a convolutional neural network method extracting local residue patterns of proteins participating in DTIs [78]. All these computational methods rely on existing (i.e., positive) drug–target interactions data; in many cases, they also use additional relevant data such as drug–drug or target–target structural similarity [79].

Nevertheless, measuring the accuracy in drug–target interaction prediction (i.e., comparing and ranking the prediction methods correctly) is problematic in the absence of some robust ground truth. Accordingly, recent research proposes a comprehensive benchmark for assessing drug–target interaction prediction methods [80], which will allow for their standardized and fair comparison.

#### Building negative drug–target interaction datasets

Robust DTI prediction requires a better ground truth, including the collections of interactions proven as nonexistent. However, it is impossible to test experimentally all potential DTIs because the search space is too extended.

The efficient training of DTI prediction machine learning models requires positive and negative examples—drug–target pairs experimentally demonstrated as interacting and drug–target couples proven as noninteracting. The DTIs in most drug datasets are robust positive examples because researchers demonstrated them experimentally; however, recording negative drug–target interaction examples is hard to approach [81, 82]. Most drug databases—including DrugBank—contain data supported by experimental research results reported in scientific papers. Such experiments are too expensive to aim at confirming the absence of drug–target interactions. Indeed, the economically reasonable approach is to spend valuable resources to demonstrate existing drug–target interactions because they will lead to new therapies. In this context, a viable approach is prioritizing the potential drug–target interactions by developing computational tools for predicting nonexisting DTIs.

Many approaches interpret nonconfirmed drug–target interactions as mere noninteractions because the negative DTI information is still scarce. Although frequent, such a practice is misleading since the lack of evidence that something exists does not necessarily mean it does not exist [26].

## Potential Implications

This article investigates whether the evolution of drug databases over the past decade has brought—besides the increasing abundance of data—a more accurate and robust analysis of drug interaction networks. We found that the data abundance in the latest DrugBank versions has rendered DDI networks almost impossible to analyze because of their huge density; we also concluded that the DTI networks built with data from the latest database versions only slightly improve the analysis robustness. Fortunately, our investigation also uncovered some database issues that need adjustments (see “Discussion” section). Fixing the reported problems will have far-reaching research implications.

First, in the case of drug–drug interaction data (and DDI networks), in our opinion, the field requires a research effort to define standardized labels for interaction severity. The drug–drug interaction severity labels currently used by drug databases—such as Drugs.com and DrugBank—are character strings: “major,” “moderate,” “minor,” and “no interactions found.” While such labels convey a valuable message to pharmacologists, they are not helpful for statistical analysis or machine learning approaches. Clearly, any statistical or machine learning method must properly quantify the differences between severity labels. By this logic, it is difficult to quantify the difference between character strings “major” and “moderate” in comparison with, say, the difference between “major” and “no interactions found”; yet, from a pharmacological standpoint, the difference is substantial. Consequently, we consider that the field needs rigorous research to define numerical labels for the drug–drug interaction severity labels. Such an undertaking would not be trivial because merely respectively encoding “major,” “moderate,” “minor,” and “no interactions were found” as “3,” “2,” “1,” and “0” does not solve all issues. For instance, Drugs.com considers “major” interactions that are either “contraindicated” in any circumstance or tolerated under medical supervision; there is an evident difference between the 2 types of “major” drug–drug interactions.

Second, owing to the lack of negative information (i.e., drug–drug and drug–target interactions proven experimentally as nonexistent; see “Discussion” section), there is still much uncertainty even in the latest drug database versions. Consequently, it remains hard to assess the performance of drug–drug and drug–target interaction prediction methods (as well as the effectiveness of computational drug repositioning pipelines) in the absence of standardized ground truth. In our opinion, to have a fair and reliable comparison of drug–drug and drug–target prediction methods, we need comprehensive benchmark datasets. In fairness, recent research efforts acknowledge this need for comprehensive benchmarking [80]; however, we think that a lot more research should be spent to standardize such benchmarks and benchmark suites, which the bioinformatics community should adopt. To this end, we believe we can draw inspiration from the way the computer architecture community managed to standardize the measurement of computer performance with the SPEC benchmark suites [83].

Third, any comprehensive benchmark dataset must contain negative information—namely, drug–drug and drug–target interactions proven impossible (or noexistent). The effort of collecting a dataset of negative drug–target interaction examples is not necessarily daunting. To this end, we suggest that computational methods—such as molecular docking or molecular fingerprints [40, 84]—can be employed to precisely identify the most likely noninteractions. Thus, the resources entailed by the confirming experiments will be mitigated substantially.

## Availability of Source Code and Requirements

Our GitHub [85] hosts the software implementing the data analysis methods described in this article—entitled *Drug Database Statistics*. The implementation uses Python and Wolfram Language; it is platform independent and requires Docker Desktop on Microsoft Windows or Docker Engine on Linux distros. The software can be used under the GNU GPL v3.0 license.

## Supplementary Material

giad011_GIGA-D-22-00237_Original_Submission

giad011_GIGA-D-22-00237_Revision_1

giad011_GIGA-D-22-00237_Revision_2

giad011_Response_to_Reviewer_Comments_Original_Submission

giad011_Response_to_Reviewer_Comments_Revision_1

giad011_Reviewer_1_Report_Original_Submission Wen Zhang -- 10/7/2022

giad011_Reviewer_1_Report_Revision_1 Wen Zhang -- 12/24/2022

giad011_Reviewer_2_Report_Original_Submission Maha A. Thafar, Ph.D. -- 10/22/2022

## Data Availability

In this paper, we use data from the DrugBank databases, which can be downloaded from https://go.drugbank.com/releases (versions 5.1.9 to 4.5.0) and https://go.drugbank.com/downloads/archived (versions 4.3 to 3.0). We also provide an archival copy of our GitHub repository via the *GigaScience* database GigaDB [86].

## Additional Files

We provide all the analyses and simulations numerical results in file https://github.com/research-hyperion/Drug_database_statistics/blob/master/Drug-networks-results-synthesis.xlsx.

## Abbreviations

ATC: anatomical therapeutic chemical classification system; COVID-19: coronavirus disease 2019; COX: cyclooxygenase; DDI: drug–drug interaction; DTI: drug–target interaction; FDA: US Food and Drug Administration; SPEC: standard performance evaluation corporation.
