# Supplementary material for: The curse and blessing of abundance—the evolution of drug interaction databases and their impact on drug network analysis
Source: Gigascience. 2023 Mar 9;12:giad011. doi: 10.1093/gigascience/giad011 (PMC10023830; doi:10.1093/gigascience/giad011)
Supplement: giad011_GIGA-D-22-00237_Revision_1 [file giad011_giga-d-22-00237_revision_1.pdf]

GigaScience

The curse and blessing of abundance---the evolution of drug interaction databases and their impact on drug network analysis

--Manuscript Draft--

**Manuscript Number:**	GIGA-D-22-00237R1
**Full Title:**	The curse and blessing of abundance---the evolution of drug interaction databases and their impact on drug network analysis
**Article Type:**	Research
**Funding Information:**	Colegiul Consultativ pentru Cercetare-Dezvoltare și Inovare (PN- III-P2-2.1-PED-2019-2842) — dr. Lucretia Udrescu
**Abstract:**	Background<br>Widespread bioinformatics applications such as drug repositioning or drug-drug interaction prediction rely on the recent advances in machine learning, complex network science, and comprehensive drug datasets comprising the latest research results in molecular biology, biochemistry, or pharmacology. The problem is that there is much uncertainty in these drug datasets---we know the drug-drug or drug-target interactions reported in the research papers, but we cannot know if the not reported interactions are absent or yet to be discovered. This uncertainty hampers the accuracy of such bioinformatics applications.<br>Results<br>We use complex network statistics tools and simulations of randomly-inserted previously unaccounted interactions in drug-drug and drug-target interaction networks---built with data from DrugBank versions released over the last decade---to investigate whether the abundance of new research data (included in the latest dataset versions) mitigate the uncertainty issue. Our results show that the drug-drug interaction networks built with the latest dataset versions become very dense and, therefore, almost impossible to analyze with conventional complex network methods. On the other hand, for the latest drug database versions, drug-target networks still include much uncertainty; however, the robustness of complex network analysis methods slightly improves.<br>Conclusions<br>Our big data analysis results pinpoint future research directions to improve the quality and practicality of drug databases for bioinformatics applications: benchmarking for drug-target interaction prediction and drug-drug interaction severity standardization.
**Corresponding Author:**	Mihai Udrescu<br>Politehnica University of Timisoara: Universitatea Politehnica din Timisoara<br>Timisoara, ROMANIA
**Corresponding Author Secondary Information:**	
**Corresponding Author's Institution:**	Politehnica University of Timisoara: Universitatea Politehnica din Timisoara
**Corresponding Author's Secondary Institution:**	
**First Author:**	Mihai Udrescu
**First Author Secondary Information:**	
**Order of Authors:**	Mihai Udrescu
	Sebastian Mihai Ardelean
	Lucretia Udrescu
**Order of Authors Secondary Information:**	
**Response to Reviewers:**	---Please also see the attached file "Response to reviewers and marked changes.pdf". the response contains mathematical formulas and special characters, which we cannot include in this box. ---

Powered by Editorial Manager® and ProduXion Manager® from Aries Systems Corporation

Response to Reviewers

November 18, 2022

We thank the Academic Editor for allowing us to upgrade our manuscript and to address all comments. We would also like to thank the reviewers for their constructive feedback, which we believe has improved the quality of our paper. The revised version of the manuscript highlights our changes in blue.
Since all three reviewers raise multiple points, we have taken the liberty of responding point by point. All text written in bolded italics corresponds to Reviewers' comments. Our responses are written with regular fonts, while the references we made to changes incorporated in the revised manuscript are underlined.
We hope to have answered all of the reviewers' comments satisfactorily.

EDITOR COMMENTS

In particular, both reviewers feel the problem addressed in the paper and related concepts need to be explained and defined more clearly.

Response: We carefully addressed the concerns formulated by the reviewers—particularly those concerning the concepts we used—and hope they are explained more clearly in the revised version of the manuscript.

REVIEWER COMMENTS

Reviewer #1 (Remarks to the Author):

In recent years, more and more comprehensive drug datasets have been proposed, however, there is much uncertainty in these drug datasets. Researchers cannot know if the not reported interactions are absent or yet to be discovered, which hampers the accuracy of bioinformatics applications on these datasets. The authors used complex network statistics tools and simulations of randomly-inserted previously unaccounted interactions in drug-drug and drug-target interaction networks, and found that the drug-drug interaction networks built with the latest dataset versions become very dense and, therefore, almost impossible to analyze with conventional complex network methods. On the other hand, for the latest drug database versions, drug-target networks still include much uncertainty; however, the robustness of complex network analysis methods slightly improves. The authors pinpoint future research directions to improve the quality and practicality of drug databases for bioinformatics applications: benchmarking for drug-target interaction prediction and drug-drug interaction severity standardization.

Response: We very much appreciate the thorough, insightful, and fair assessment of our work. The observations and comments will undoubtedly contribute to a more robust, readable, and wide-reaching paper.

Comments and suggestions:
The author should explain more clearly the relationship among 'uncertain issue of datasets', 'the evolution of various complex network parameters and centralities', 'the integrity of the processed data' and 'the robustness of centrality-hierarchization analysis methods'.

Response:
We thank the reviewer for showing the need for clarity in the Background section—the part dedicated to analyzing the current issues in drug dataset analysis and the summary of our contributions. We used the terms cited by the reviewer in the enumeration (with bullets) of "our DDI and DTI network analysis across the DrugBank database versions."

In the initially submitted manuscript, the paragraph that tried to explain the connections between the "uncertainty issue of datasets," "the evolution of various complex network parameters and centralities," "the integrity of the processed data," and "the robustness

of centrality-hierarchization analysis methods" read:
"Over the years, the comprehensiveness of drug datasets (such as DrugBank [28]) has constantly grown, and the data accuracy improved by manual curation, according to the latest literature results. This constant evolution of the most comprehensive drug database may have mitigated the concerns formulated by Mestres et al. Our paper investigates how the evolution of knowledge on drug interactions, mirrored by the DrugBank database, impacts the robustness of drug interaction network analysis; future research can exploit the insight we get to advance drug repositioning and drug-drug interaction prediction methods. "

Nonetheless, the reviewer is right. In the above paragraph, we used different terms than those used in the enumeration of the contributions, causing a lack of clarity and confusion. Following the reviewer's suggestion, we revised the paragraph that was intended to explain the mentioned terms. We hope that this part is much clearer in the revised form, which reads:

"As long as we do not have comprehensive negative information, the issue of uncertainty in drug datasets remains. However, the fact that—over the years—the comprehensiveness of drug datasets (such as DrugBank [28]) has constantly grown, and experts curated the data according to the latest literature results, may suggest that the integrity of the data improved. (The integrity characterizes the "absence of improper data alterations" [29].) Indeed, the constant growth of the most comprehensive drug database (reflected in the evolution of the various complex drug network parameters and centralities) may have mitigated the concerns formulated by Mestres et al. Our paper investigates how the evolution of knowledge on drug interactions mirrored by the DrugBank database, impacts the robustness of drug interaction network analysis based on centrality hierarchization methods; future research can exploit the insight we get to advance drug repositioning and drug-drug interaction prediction methods." (Section Background, paragraph 7 on page 2 in the revised manuscript.)

We introduced a new reference that acknowledges the rigorous definition of system integrity:
[29] Avizienis, A., Laprie, J.C., Randell, B. and Landwehr, C., 2004. Basic concepts and taxonomy of dependable and secure computing. IEEE Transactions on Dependable and Secure Computing, 1(1), pp.11-33.

Comment: The author should clearly define the 'typical complex network', for example, the characteristics of a typical complex network and the differences between a typical complex network and a normal network. In addition, the first sentence of the first paragraph in the section 'Complex networks' seems to be a general definition of the 'network', not the 'complex network'.

Response: We thank the reviewer for the observation. First, we added the necessary details to the definition of complex networks, which now reads, "A complex network is a graph G consisting of a node (or vertex) set V and a link (or edge) set E, G = (V, E), where any edge e_{ij}\in E connects nodes v_{i\ }{,v}_j\in V. Additionally, compared to normal graphs, real-world complex networks have a large number of nodes \left|V\right| (i.e., hundreds to millions) and intricate, nontrivial topologies (i.e., links do not form regular structures but are not completely random either)." We highlighted the addition in blue text, in section Problem formulation, first paragraph in subsection Complex networks, page 3.

Second, we defined the typical characteristics of real-world complex networks in the manuscript in a new subsection titled Typical complex network characteristics (page 6 in the revised manuscript). We also point to this new subsection when using the term "typical complex networks" for the first time: "We explain the typical characteristics of real-world complex networks in subsection Typical complex network characteristics" (see the blue text on page 3, 4th paragraph). In the revised form of our manuscript, this new subsection reads, "Generally, in real-world complex networks, the density of links is low (i.e., \sim\frac{1}{n} because the number of links is of the order n, where n is the number of nodes, and far from the order n^2 corresponding to a completely connected network); the total number of links also determines the average degree, and the average degree is significantly lower than n; the degree follows either a power-law

Powered by Editorial Manager® and ProduXion Manager® from Aries Systems Corporation

(exponent is between 1 and 3) or normal distribution; the average path length is between 3 and 10; the clustering coefficient is larger than \frac{1}{n} but considerably lower than 1 [46, 54]." We highlighted these clarifications in blue text, section Methods, subsection Typical complex network characteristics, on page 6.

[46] Wang, X.F. and Chen, G., 2003. Complex networks: small-world, scale-free and beyond. IEEE circuits and systems magazine, 3(1), pp.6-20.
[54] Topirceanu, A., Udrescu, M. and Vladutiu, M., 2014. Genetically optimized realistic social network topology inspired by facebook. In Online Social Media Analysis and Visualization (pp. 163-179). Springer, Cham.

Comment: In the last sentence of the third paragraph in the section 'Complex networks', 'wij̸ = wji' should be 'wij=wji'.

Response: The observation is correct, and we thank the reviewer for pointing out this typo; we corrected it in the revision (see the first paragraph on page 4).

Comment: In Equation (10), the function η() doesn't seem to be defined.

Response: The reviewer is right; we did not explain the eigenvector with clarity (while maintaining conciseness). We denoted the eccentricity of a node v_i as \eta(v_i). Hence, equation (10) shows that eigenvector centrality for v_i is defined recursively: it adds the centralities of the nodes directly connected to v_i. To find the eigenvector centrality values for all nodes in the node set V of the network G = (V, E), we solve the equation Wη = λη; W is the adjacency matrix, λ is a constant, and η is the vector of eigenvector centrality values for all nodes in G. We added these clarifying details for equation 10 in the revised manuscript (please see the blue text on page 6, subsubsection Network centralities).

Comment: In the section 'Discussion', the authors should include more analysis of the results. In addition, I suggest that the authors should elaborate analysis and conclusions with relevant findings from the experimental results, instead of talking about them separately.

Response: The reviewer is correct: our Discussion section needs to be organized better. According to the suggestions, we performed the following steps:
We renamed the subsections of the Discussion as Discussions on DDI networks and Discussions on DTI networks.
We extended the discussions to new issues, such as:
The benefits of building prediction tools for identifying negative information (i.e., drug-drug and drug-target interactions that do not exist);
The need for recording the relevant clinical context for drug-drug interaction severity;
The fact that different drug datasets often disagree on the drug-drug interaction severity.
We reorganized the subsections such that we directly corroborate our analysis conclusions with the experimental results; these two parts are no longer separated.
We rewrite the entire Discussion section to eliminate redundant wording, thus bringing more conciseness and clarity to the manuscript.
Please find the revised form of the Discussion section in blue, starting on page 10.

Comment: Many important literature about the drug interactions have not been reviewed in the paper, such as DOI:10.1109/TPAMI.2021.3135841, DOI:10.1109/TKDE.2022.3154792, doi: 10.1186/s12859-016-1415-9, DOI:10.1109/TCBB.2021.3081268, etc.

Response: We thank the reviewer for pointing out that we did not include proper citations for papers on drug-drug interaction predictions reached from drug-drug and drug-target interactions. Indeed, drug-drug interaction prediction is highly relevant to the central theme of our paper. We added a paragraph in the Background section that explains the application of drug-drug and drug-target interaction prediction (besides drug repositioning, which we already presented in detail), where we correctly cited the outstanding work on drug-drug interaction prediction suggested by the reviewer. (Please, see the blue-text in section Background, first two paragraphs on page 2.) The

Powered by Editorial Manager® and ProduXion Manager® from Aries Systems Corporation

new paragraphs read, "As such, harnessing big amounts of data describing the intricate drug interactions (or relationships) with other entities can lead to uncovering new drug properties: previously unknown drug-drug [3, 4, 5, 6] or drug-target interactions [7, 8, 9, 10, 11], drug repositioning [12, 13, 14]. // Computational drug-drug interaction (DDI) prediction is critical in designing effective and safe drug combinations and developing drugs [5, 6]. Drug-target interaction (DTI) prediction is a key-step strategy in drug discovery that leads to uncovering new drugs for biological targets and new targets for known, approved drugs—representing a crucial step towards drug repositioning and identifying possible side effects [8, 11]."

[3] Zhang, W., Chen, Y., Liu, F., Luo, F., Tian, G. and Li, X., 2017. Predicting potential drug-drug interactions by integrating chemical, biological, phenotypic and network data. BMC bioinformatics, 18(1), pp.1-12.
[4] Zhu, J., Liu, Y., Zhang, Y., Chen, Z. and Wu, X., 2021. Multi-Attribute Discriminative Representation Learning for Prediction of Adverse Drug-Drug Interaction. IEEE Transactions on Pattern Analysis and Machine Intelligence.
[5] Qiu, Y., Zhang, Y., Deng, Y., Liu, S. and Zhang, W., 2021. A comprehensive review of computational methods for drug-drug interaction detection. IEEE/ACM Transactions on Computational Biology and Bioinformatics.
[6] Su, X., You, Z.H., Huang, D.S., Wang, L., Wong, L., Ji, B. and Zhao, B., 2022. Biomedical Knowledge Graph Embedding with Capsule Network for Multi-label Drug-Drug Interaction Prediction. IEEE Transactions on Knowledge and Data Engineering.
[7] Lu, Y., Guo, Y. and Korhonen, A., 2017. Link prediction in drug-target interactions network using similarity indices. BMC bioinformatics, 18(1), pp.1-9.
[8] Luo, Y., Zhao, X., Zhou, J., Yang, J., Zhang, Y., Kuang, W., Peng, J., Chen, L. and Zeng, J., 2017. A network integration approach for drug-target interaction prediction and computational drug repositioning from heterogeneous information. Nature communications, 8(1), pp.1-13.
[9] Wu, Z., Li, W., Liu, G. and Tang, Y., 2018. Network-based methods for prediction of drug-target interactions. Frontiers in pharmacology, 9, p.1134.
[10] Thafar, M.A., Olayan, R.S., Ashoor, H., Albaradei, S., Bajic, V.B., Gao, X., Gojobori, T. and Essack, M., 2020. DTiGEMS+: drug–target interaction prediction using graph embedding, graph mining, and similarity-based techniques. Journal of Cheminformatics, 12(1), pp.1-17.
[11] Thafar, M.A., Olayan, R.S., Albaradei, S., Bajic, V.B., Gojobori, T., Essack, M. and Gao, X., 2021. DTi2Vec: Drug–target interaction prediction using network embedding and ensemble learning. Journal of cheminformatics, 13(1), pp.1-18.
[12] Lotfi Shahreza, M., Ghadiri, N., Mousavi, S.R., Varshosaz, J. and Green, J.R., 2018. A review of network-based approaches to drug repositioning. Briefings in bioinformatics, 19(5), pp.878-892.
[13] Sadeghi, S.S. and Keyvanpour, M.R., 2019. An analytical review of computational drug repurposing. IEEE/ACM transactions on computational biology and bioinformatics, 18(2), pp.472-488.
[14] Badkas, A., De Landtsheer, S. and Sauter, T., 2021. Topological network measures for drug repositioning. Briefings in bioinformatics, 22(4), p.bbaa357.

Reviewer #2 (Remarks to the Author):

The authors proposed an interesting analysis study for several drug-related networks using different versions of the DrugBank database. Therefore, the work is network analysis and machine learning-based methods to analyze the future of drug-drug interactions and drug-target interactions. Hence, this study highlights the future research that can improve the drug databases quality and for bioinformatics applications.

This work tackles a genuine topic to improve bioinformatics applications using complex networks of drug-related data. The abstract and the introduction were well written and presented good motivation, description. However, I have some concerns that have to be addressed. Therefore, I do recommend this work for publications in the GigaScience journal after addressing the following comments.

Response: We thank the reviewer for delivering a sharp, fair, and comprehensive summary of our work. The provided comments will substantially contribute to a much more robust, readable, and wide-reaching manuscript.

Comments

Comment 1. The problem formulation is not very clear. More details are needed for the negative or missing information but focused on the problem itself.

Response: We thank the reviewer for pointing out this issue. Indeed, we misplaced the explanation of the drug-drug and drug-target interaction networks using the formal framework from the Problem formulation section in the Results. We corrected this mistake by moving this part in the Problem formulation. Also, following the reviewer's suggestion, we used network formalism to explain better what missing and negative information means in the context of our problem. These added details and corrections are on page 4, section Problem formulation, subsection Drug networks (first three paragraphs). In the revised manuscript, these paragraphs read:

“To mine the complexity of drug interactions in the biological environment, researchers use database information to build drug networks such as drug-drug, drug-target, and drug-disease interaction networks, as well as drug-disease and drug--adverse effects association networks. This paper considers drug-drug interaction networks (complex drug networks where nodes represent drugs) and drug-target interaction networks (bipartite networks where one type of node represents drugs, and the other represents biological targets). Links represent drug-drug and drug-target interactions in the DDI and DTI networks. We do not consider drug-drug interaction type and strength. Consequently, the DDI networks are undirected, unweighted, and monopartite. Also, the drug-target interactions have no information regarding their strength, making DTI networks directed (from drug to target), unweighted, and bipartite.

Using the complex network formalism, in DDI networks, $\forall i,j,\ i\neq j,v_i\in V=\mathcal{D}\ $ and $e_{ij}\in E=\mathcal{I}$ ($e_{ij}\neq0$), where $\mathcal{D}$ is the set of drugs and $\ \mathcal{I}$ the set of drug-drug interactions. In DTI networks, we have $v_i\in V=V_\mathcal{D}\cup V_\mathcal{T}$ (where $V_\mathcal{D}$ is the set of nodes representing drugs and $V_\mathcal{T}$ is the set of nodes representing targets) and $e_{ij}\in E=\mathcal{A}$, where $\mathcal{A}=\left\{e_{ij}\middle| v_i\in V_\mathcal{D},v_j\in V_\mathcal{T},i\neq j,e_{ij}\neq0\right\}$ represents the set of drug-target interactions). Additionally, the set of non-edges in the DDI networks is $\bar{\mathcal{I}}=\left\{e_{ij}\middle| v_i,v_j\in\mathcal{D},i\neq j,e_{ij}=0\right\}\ $ and in the DTI is $\bar{\mathcal{A}}=\left\{e_{ij}\middle| v_i\in\mathcal{D},v_j\in\mathcal{T},i\neq j,e_{ij}=0\right\}$.

A non-edge in a DDI network (${\bar{e}}_{ij}\in\bar{\mathcal{I}}$) or a DTI network ($\in\bar{\mathcal{A}}$) represents a drug-drug or a drug-target interaction that has a probability $p\in\left(0,1\right)$ of being discovered in the future (i.e., missing information generating uncertainty). If $p=0$, we are sure that interaction between v_i and v_j is impossible (i.e., the negative information on drug interactions)."

Comment 2. The manuscript covered a literature in terms of the methods using the DrugBank database. However, this literature is not enough and more discussion about drug repositioning applications using drug-drug and drug-target network-based methods or other types of applications using DrugBank datasets can improve the manuscript, specifically the literature review. Thus, citing more relevant and recent literature on DTI and DDI prediction is recommended, which will enrich the paper.

Response: The reviewer is correct in pointing out that the Background section only provides details and citations on the drug repositioning applications. Accordingly, we corrected this downside by explaining the drug-drug and drug-target interaction prediction benefits; we cited the relevant recent literature (please, see the blue-text in section Background, first 2 paragraphs on page 2.)

The new paragraph reads, "As such, harnessing big amounts of data describing the intricate drug interactions (or relationships) with other entities can lead to uncovering new drug properties: previously unknown drug-drug [3, 4, 5, 6] or drug-target

Powered by Editorial Manager® and ProduXion Manager® from Aries Systems Corporation

interactions [7, 8, 9, 10, 11], drug repositioning [12, 13, 14]. Computational drug-drug interaction (DDI) prediction is critical in designing effective and safe drug combinations and developing drugs [5, 6]. Drug-target interaction (DTI) prediction is a key-step strategy in drug discovery that leads to uncovering new drugs for biological targets and new targets for known, approved drugs—representing a crucial step towards drug repositioning and identifying possible side effects [8, 11]."

[3] Zhang, W., Chen, Y., Liu, F., Luo, F., Tian, G. and Li, X., 2017. Predicting potential drug-drug interactions by integrating chemical, biological, phenotypic and network data. BMC bioinformatics, 18(1), pp.1-12.
[4] Zhu, J., Liu, Y., Zhang, Y., Chen, Z. and Wu, X., 2021. Multi-Attribute Discriminative Representation Learning for Prediction of Adverse Drug-Drug Interaction. IEEE Transactions on Pattern Analysis and Machine Intelligence.
[5] Qiu, Y., Zhang, Y., Deng, Y., Liu, S. and Zhang, W., 2021. A comprehensive review of computational methods for drug-drug interaction detection. IEEE/ACM Transactions on Computational Biology and Bioinformatics.
[6] Su, X., You, Z.H., Huang, D.S., Wang, L., Wong, L., Ji, B. and Zhao, B., 2022. Biomedical Knowledge Graph Embedding with Capsule Network for Multi-label Drug-Drug Interaction Prediction. IEEE Transactions on Knowledge and Data Engineering.
[7] Lu, Y., Guo, Y. and Korhonen, A., 2017. Link prediction in drug-target interactions network using similarity indices. BMC bioinformatics, 18(1), pp.1-9.
[8] Luo, Y., Zhao, X., Zhou, J., Yang, J., Zhang, Y., Kuang, W., Peng, J., Chen, L. and Zeng, J., 2017. A network integration approach for drug-target interaction prediction and computational drug repositioning from heterogeneous information. Nature communications, 8(1), pp.1-13.
[9] Wu, Z., Li, W., Liu, G. and Tang, Y., 2018. Network-based methods for prediction of drug-target interactions. Frontiers in pharmacology, 9, p.1134.
[10] Thafar, M.A., Olayan, R.S., Ashoor, H., Albaradei, S., Bajic, V.B., Gao, X., Gojobori, T. and Essack, M., 2020. DTiGEMS+: drug–target interaction prediction using graph embedding, graph mining, and similarity-based techniques. Journal of Cheminformatics, 12(1), pp.1-17.
[11] Thafar, M.A., Olayan, R.S., Albaradei, S., Bajic, V.B., Gojobori, T., Essack, M. and Gao, X., 2021. DTi2Vec: Drug–target interaction prediction using network embedding and ensemble learning. Journal of cheminformatics, 13(1), pp.1-18.
[12] Lotfi Shahreza, M., Ghadiri, N., Mousavi, S.R., Varshosaz, J. and Green, J.R., 2018. A review of network-based approaches to drug repositioning. Briefings in bioinformatics, 19(5), pp.878-892.
[13] Sadeghi, S.S. and Keyvanpour, M.R., 2019. An analytical review of computational drug repurposing. IEEE/ACM transactions on computational biology and bioinformatics, 18(2), pp.472-488.
[14] Badkas, A., De Landtsheer, S. and Sauter, T., 2021. Topological network measures for drug repositioning. Briefings in bioinformatics, 22(4), p.bbaa357.

Comment 3. The methodology section is a bit distracting. A summary paragraph can be included as well as the structure of this section can be improved.

Response: We appreciate this observation. The reviewer is correct; in the initially submitted manuscript, the Methodology section assembles all the subsections containing formal definitions, methods, and algorithms. Indeed, the Problem formulation is not a method, so we moved this part out of the Methods as a standalone/independent section (starting on page 3). We also added a summary paragraph to explain the structure of the Methods section, as suggested by the reviewer. The new paragraph, highlighted in blue on page 5, at the beginning of the section Methods, reads:
"In this section, we describe the statistical and computational methods employed in this paper: subsection Network analysis introduces the main metrics and centralities required for the computational and statistical analysis of complex drug networks; subsection Database quality presents the statistical methods we use to test the drug database integrity; subsection Network analysis robustness explains the computer simulation method we propose to assess the robustness of the centrality-based drug network analysis strategies; subsection Computational methods describes the tools we use in this paper to implement the required computational and statistical techniques."

	Comment 4. The benefit of Figure 3 is not clear. Better description with the purpose of the figure will add more value.<br><br>Response: We thank the reviewer for raising this issue. We introduced Figure 3 to show the evolution of size in drug-drug and drug-target interaction networks (i.e., as the number of drugs and targets) with the DrugBank versions. As shown, compared with the DDI and DTI sizes in the first available DrugBank version, the number of medicines/drugs almost quadrupled; in contrast, the number of targets increased at a much lower rate. This observation is in line with the other results reported in our paper, thus strengthening the overarching conclusion.<br><br>In the revised form of our manuscript, according to the reviewer's suggestion, we introduced Figure 3 with a clarifying statement. The statement now reads, "In Figure 3, panel a, we present the evolution of the number of nodes (i.e., drugs) in DDI networks; in Figure 3, panel b, we show the evolution of the number of nodes, drugs, and targets in DTI networks. As shown, from version 3.0 to 5.1.9, the number of medicines/drugs roughly quadrupled; in contrast, the number of targets increased at a much lower rate. Such an evolution suggests that, over the years, the complexity of processing and analyzing drug interaction networks grew considerably." In our revised manuscript, we highlighted this change with blue text, on page 7, section Results, subsection Network metrics and centrality analysis, paragraph 2.<br><br>Comment 5. The text can be more concise to improve the text quality especially in the discussion.<br><br>Response: The reviewer is correct: our Discussion section needs better structure and wording. According to the suggestion, we performed the following steps:<br>We renamed the subsections of the Discussion as Discussions on DDI networks and Discussions on DTI networks.<br>We rewrite the entire Discussion section to eliminate redundant wording, thus bringing more conciseness and clarity to the manuscript.<br>Please find the revised form of the Discussion section in blue, starting on page 10.
**Additional Information:**	
**Question**	**Response**
Are you submitting this manuscript to a special series or article collection?	No
**Experimental design and statistics**<br><br>Full details of the experimental design and statistical methods used should be given in the Methods section, as detailed in our Minimum Standards Reporting Checklist. Information essential to interpreting the data presented should be made available in the figure legends.<br><br>Have you included all the information requested in your manuscript?	Yes
**Resources**<br><br>A description of all resources used,	Yes

including antibodies, cell lines, animals and software tools, with enough information to allow them to be uniquely identified, should be included in the Methods section. Authors are strongly encouraged to cite Research Resource Identifiers (RRIDs) for antibodies, model organisms and tools, where possible.<br><br>Have you included the information requested as detailed in our Minimum Standards Reporting Checklist?	
**Availability of data and materials**<br><br>All datasets and code on which the conclusions of the paper rely must be either included in your submission or deposited in publicly available repositories (where available and ethically appropriate), referencing such data using a unique identifier in the references and in the "Availability of Data and Materials" section of your manuscript.<br><br>Have you have met the above requirement as detailed in our Minimum Standards Reporting Checklist?	Yes

Click here to access/download;LaTeX - Main Document (TeX file);main.tex

```
This is pdfTeX, Version 3.141592653-2.6-1.40.24 (TeX Live 2022)
(preloaded format=pdflatex 2022.10.28)  18 NOV 2022 10:11
entering extended mode
 restricted \write18 enabled.
 %&-line parsing enabled.
**main.tex
(./main.tex
LaTeX2e <2022-06-01> patch level 5
L3 programming layer <2022-10-26> (./oup-contemporary.cls
Document Class: oup-contemporary 2017/06/28, v1.1
(c:/TeXLive/2022/texmf-dist/tex/latex/base/article.cls
Document Class: article 2021/10/04 v1.4n Standard LaTeX document class
(c:/TeXLive/2022/texmf-dist/tex/latex/base/size10.clo
File: size10.clo 2021/10/04 v1.4n Standard LaTeX file (size option)
)
\c@part=\count185
\c@section=\count186
\c@subsection=\count187
\c@subsubsection=\count188
\c@paragraph=\count189
\c@subparagraph=\count190
\c@figure=\count191
\c@table=\count192
\abovecaptionskip=\skip47
\belowcaptionskip=\skip48
\bibindent=\dimen138
) (c:/TeXLive/2022/texmf-dist/tex/latex/base/inputenc.sty
Package: inputenc 2021/02/14 v1.3d Input encoding file
\inpenc@prehook=\toks16
\inpenc@posthook=\toks17
) (c:/TeXLive/2022/texmf-dist/tex/latex/base/fontenc.sty
Package: fontenc 2021/04/29 v2.0v Standard LaTeX package
) (c:/TeXLive/2022/texmf-dist/tex/generic/iftex/ifpdf.sty
Package: ifpdf 2019/10/25 v3.4 ifpdf legacy package. Use iftex instead.
(c:/TeXLive/2022/texmf-dist/tex/generic/iftex/iftex.sty
Package: iftex 2022/02/03 v1.0f TeX engine tests
)) (c:/TeXLive/2022/texmf-dist/tex/latex/microtype/microtype.sty
Package: microtype 2022/06/23 v3.0f Micro-typographical refinements (RS)
(c:/TeXLive/2022/texmf-dist/tex/latex/graphics/keyval.sty
Package: keyval 2022/05/29 v1.15 key=value parser (DPC)
\KV@toks@=\toks18
) (c:/TeXLive/2022/texmf-dist/tex/latex/etoolbox/etoolbox.sty
Package: etoolbox 2020/10/05 v2.5k e-TeX tools for LaTeX (JAW)
\etb@tempcnta=\count193
)
\MT@toks=\toks19
\MT@tempbox=\box51
\MT@count=\count194
LaTeX Info: Redefining \noprotrusionifhmode on input line 1045.
LaTeX Info: Redefining \leftprotrusion on input line 1046.
LaTeX Info: Redefining \rightprotrusion on input line 1056.
LaTeX Info: Redefining \textls on input line 1234.
\MT@outer@kern=\dimen139
LaTeX Info: Redefining \textmicrotypecontext on input line 1858.
```

```
\MT@listname@count=\count195
(c:/TeXLive/2022/texmf-dist/tex/latex/microtype/microtype-pdftex.def
File: microtype-pdftex.def 2022/06/23 v3.0f Definitions specific to
pdftex (RS)

LaTeX Info: Redefining \lsstyle on input line 900.
LaTeX Info: Redefining \lslig on input line 900.
\MT@outer@space=\skip49
)
Package microtype Info: Loading configuration file microtype.cfg.
(c:/TeXLive/2022/texmf-dist/tex/latex/microtype/microtype.cfg
File: microtype.cfg 2022/06/23 v3.0f microtype main configuration file
(RS)
)) (c:/TeXLive/2022/texmf-dist/tex/latex/euler/euler.sty
Package: euler 1995/03/05 v2.5
Package: `euler' v2.5 <1995/03/05> (FJ and FMi)
LaTeX Font Info:    Redeclaring symbol font `letters' on input line 35.
LaTeX Font Info:    Encoding `OML' has changed to `U' for symbol font
(Font)              `letters' in the math version `normal' on input line
35.
LaTeX Font Info:    Overwriting symbol font `letters' in version `normal'
(Font)                  OML/cmm/m/it --> U/eur/m/n on input line 35.
LaTeX Font Info:    Encoding `OML' has changed to `U' for symbol font
(Font)              `letters' in the math version `bold' on input line
35.
LaTeX Font Info:    Overwriting symbol font `letters' in version `bold'
(Font)                  OML/cmm/b/it --> U/eur/m/n on input line 35.
LaTeX Font Info:    Overwriting symbol font `letters' in version `bold'
(Font)                  U/eur/m/n --> U/eur/b/n on input line 36.
LaTeX Font Info:    Redeclaring math symbol \Gamma on input line 47.
LaTeX Font Info:    Redeclaring math symbol \Delta on input line 48.
LaTeX Font Info:    Redeclaring math symbol \Theta on input line 49.
LaTeX Font Info:    Redeclaring math symbol \Lambda on input line 50.
LaTeX Font Info:    Redeclaring math symbol \Xi on input line 51.
LaTeX Font Info:    Redeclaring math symbol \Pi on input line 52.
LaTeX Font Info:    Redeclaring math symbol \Sigma on input line 53.
LaTeX Font Info:    Redeclaring math symbol \Upsilon on input line 54.
LaTeX Font Info:    Redeclaring math symbol \Phi on input line 55.
LaTeX Font Info:    Redeclaring math symbol \Psi on input line 56.
LaTeX Font Info:    Redeclaring math symbol \Omega on input line 57.
\symEulerFraktur=\mathgroup4
LaTeX Font Info:    Overwriting symbol font `EulerFraktur' in version
`bold'
(Font)                  U/euf/m/n --> U/euf/b/n on input line 63.
LaTeX Info: Redefining \oldstylenums on input line 85.
\symEulerScript=\mathgroup5
LaTeX Font Info:    Overwriting symbol font `EulerScript' in version
`bold'
(Font)                  U/eus/m/n --> U/eus/b/n on input line 93.
LaTeX Font Info:    Redeclaring math symbol \aleph on input line 97.
LaTeX Font Info:    Redeclaring math symbol \Re on input line 98.
LaTeX Font Info:    Redeclaring math symbol \Im on input line 99.
LaTeX Font Info:    Redeclaring math delimiter \vert on input line 101.
```

```
LaTeX Font Info:    Redeclaring math delimiter \backslash on input line
103.
LaTeX Font Info:    Redeclaring math symbol \neg on input line 106.
LaTeX Font Info:    Redeclaring math symbol \wedge on input line 108.
LaTeX Font Info:    Redeclaring math symbol \vee on input line 110.
LaTeX Font Info:    Redeclaring math symbol \setminus on input line 112.
LaTeX Font Info:    Redeclaring math symbol \sim on input line 113.
LaTeX Font Info:    Redeclaring math symbol \mid on input line 114.
LaTeX Font Info:    Redeclaring math delimiter \arrowvert on input line
116.
LaTeX Font Info:    Redeclaring math symbol \mathsection on input line
117.
\symEulerExtension=\mathgroup6
LaTeX Font Info:    Redeclaring math symbol \coprod on input line 125.
LaTeX Font Info:    Redeclaring math symbol \prod on input line 125.
LaTeX Font Info:    Redeclaring math symbol \sum on input line 125.
LaTeX Font Info:    Redeclaring math symbol \intop on input line 130.
LaTeX Font Info:    Redeclaring math symbol \ointop on input line 131.
LaTeX Font Info:    Redeclaring math symbol \braceld on input line 132.
LaTeX Font Info:    Redeclaring math symbol \bracerd on input line 133.
LaTeX Font Info:    Redeclaring math symbol \bracelu on input line 134.
LaTeX Font Info:    Redeclaring math symbol \braceru on input line 135.
LaTeX Font Info:    Redeclaring math symbol \infty on input line 136.
LaTeX Font Info:    Redeclaring math symbol \nearrow on input line 153.
LaTeX Font Info:    Redeclaring math symbol \searrow on input line 154.
LaTeX Font Info:    Redeclaring math symbol \nwarrow on input line 155.
LaTeX Font Info:    Redeclaring math symbol \swarrow on input line 156.
LaTeX Font Info:    Redeclaring math symbol \Leftrightarrow on input line
157.
LaTeX Font Info:    Redeclaring math symbol \Leftarrow on input line 158.
LaTeX Font Info:    Redeclaring math symbol \Rightarrow on input line
159.
LaTeX Font Info:    Redeclaring math symbol \leftrightarrow on input line
160.
LaTeX Font Info:    Redeclaring math symbol \leftarrow on input line 161.
LaTeX Font Info:    Redeclaring math symbol \rightarrow on input line
163.
LaTeX Font Info:    Redeclaring math delimiter \uparrow on input line
166.
LaTeX Font Info:    Redeclaring math delimiter \downarrow on input line
168.
LaTeX Font Info:    Redeclaring math delimiter \updownarrow on input line
170.
LaTeX Font Info:    Redeclaring math delimiter \Uparrow on input line
172.
LaTeX Font Info:    Redeclaring math delimiter \Downarrow on input line
174.
LaTeX Font Info:    Redeclaring math delimiter \Updownarrow on input line
176.
LaTeX Font Info:    Redeclaring math symbol \leftharpoonup on input line
177.
LaTeX Font Info:    Redeclaring math symbol \leftharpoondown on input
line 178.
```

```
LaTeX Font Info:    Redeclaring math symbol \rightharpoonup on input line
179.
LaTeX Font Info:    Redeclaring math symbol \rightharpoondown on input
line 180
.
LaTeX Font Info:    Redeclaring math delimiter \lbrace on input line 182.
LaTeX Font Info:    Redeclaring math delimiter \rbrace on input line 184.
\symcmmigroup=\mathgroup7
LaTeX Font Info:    Overwriting symbol font `cmmigroup' in version `bold'
(Font)                  OML/cmm/m/it --> OML/cmm/b/it on input line 200.
LaTeX Font Info:    Redeclaring math accent \vec on input line 201.
LaTeX Font Info:    Redeclaring math symbol \triangleleft on input line
202.
LaTeX Font Info:    Redeclaring math symbol \triangleright on input line
203.
LaTeX Font Info:    Redeclaring math symbol \star on input line 204.
LaTeX Font Info:    Redeclaring math symbol \lhook on input line 205.
LaTeX Font Info:    Redeclaring math symbol \rhook on input line 206.
LaTeX Font Info:    Redeclaring math symbol \flat on input line 207.
LaTeX Font Info:    Redeclaring math symbol \natural on input line 208.
LaTeX Font Info:    Redeclaring math symbol \sharp on input line 209.
LaTeX Font Info:    Redeclaring math symbol \smile on input line 210.
LaTeX Font Info:    Redeclaring math symbol \frown on input line 211.
LaTeX Font Info:    Redeclaring math accent \grave on input line 245.
LaTeX Font Info:    Redeclaring math accent \acute on input line 246.
LaTeX Font Info:    Redeclaring math accent \tilde on input line 247.
LaTeX Font Info:    Redeclaring math accent \ddot on input line 248.
LaTeX Font Info:    Redeclaring math accent \check on input line 249.
LaTeX Font Info:    Redeclaring math accent \breve on input line 250.
LaTeX Font Info:    Redeclaring math accent \bar on input line 251.
LaTeX Font Info:    Redeclaring math accent \dot on input line 252.
LaTeX Font Info:    Redeclaring math accent \hat on input line 254.
) (c:/TeXLive/2022/texmf-dist/tex/latex/merriweather/merriweather.sty
Package: merriweather 2022/09/20 (Bob Tennent) Supports
Merriweather(Sans) font
s for all LaTeX engines.
(c:/TeXLive/2022/texmf-dist/tex/generic/iftex/ifxetex.sty
Package: ifxetex 2019/10/25 v0.7 ifxetex legacy package. Use iftex
instead.
) (c:/TeXLive/2022/texmf-dist/tex/generic/iftex/ifluatex.sty
Package: ifluatex 2019/10/25 v1.5 ifluatex legacy package. Use iftex
instead.
) (c:/TeXLive/2022/texmf-dist/tex/latex/base/textcomp.sty
Package: textcomp 2020/02/02 v2.0n Standard LaTeX package
) (c:/TeXLive/2022/texmf-dist/tex/latex/xkeyval/xkeyval.sty
Package: xkeyval 2022/06/16 v2.9 package option processing (HA)
(c:/TeXLive/2022/texmf-dist/tex/generic/xkeyval/xkeyval.tex
(c:/TeXLive/2022/te
xmf-dist/tex/generic/xkeyval/xkvutils.tex
\XKV@toks=\toks20
\XKV@tempa@toks=\toks21
)
\XKV@depth=\count196
File: xkeyval.tex 2014/12/03 v2.7a key=value parser (HA)
```

```
)) (c:/TeXLive/2022/texmf-dist/tex/latex/base/fontenc.sty
Package: fontenc 2021/04/29 v2.0v Standard LaTeX package
) (c:/TeXLive/2022/texmf-dist/tex/latex/fontaxes/fontaxes.sty
Package: fontaxes 2020/07/21 v1.0e Font selection axes
LaTeX Info: Redefining \upshape on input line 29.
LaTeX Info: Redefining \itshape on input line 31.
LaTeX Info: Redefining \slshape on input line 33.
LaTeX Info: Redefining \swshape on input line 35.
LaTeX Info: Redefining \scshape on input line 37.
LaTeX Info: Redefining \sscshape on input line 39.
LaTeX Info: Redefining \ulcshape on input line 41.
LaTeX Info: Redefining \textsw on input line 47.
LaTeX Info: Redefining \textssc on input line 48.
LaTeX Info: Redefining \textulc on input line 49.
)) (c:/TeXLive/2022/texmf-dist/tex/latex/mathastext/mathastext.sty
Package: mathastext 2019/11/16 v1.3w Use the text font in math mode (JFB)
\mst@exists@muskip=\muskip16
\mst@forall@muskip=\muskip17
\mst@prime@muskip=\muskip18
\mst@do@nonletters=\toks22
\mst@do@easynonletters=\toks23
\mst@do@az=\toks24
\mst@do@AZ=\toks25
\symmtoperatorfont=\mathgroup8
\symmtletterfont=\mathgroup9
** ! and ?
** punctuation: , . : ; and \colon
LaTeX Info: Redefining \relbar on input line 787.
LaTeX Info: Redefining \rightarrowfill on input line 790.
LaTeX Info: Redefining \leftarrowfill on input line 795.
** + and =
LaTeX Info: Redefining \Relbar on input line 886.
** adding = ; and + to \nfss@catcodes
** parentheses ( ) [ ] and slash /
** alldelims: < > \backslash \setminus | \vert \mid \{ and \}
LaTeX Font Info:    Redeclaring math delimiter \backslash on input line
932.
LaTeX Font Info:    Redeclaring math symbol \setminus on input line 944.
LaTeX Info: Redefining \models on input line 953.
** \# \mathdollar \% \&
** \imath and \jmath
LaTeX Font Info:    Overwriting math alphabet `\mathnormalbold' in
version `nor
mal'
(Font)                  T1/Merriwthr-OsF/b/it --> T1/Merriwthr-OsF/b/it
on inpu
t line 2140.
LaTeX Font Info:    Overwriting math alphabet `\mathnormalbold' in
version `bol
d'
(Font)                  T1/Merriwthr-OsF/b/it --> T1/Merriwthr-OsF/b/it
on inpu
t line 2140.
```

```
LaTeX Font Info:    Overwriting symbol font `mtletterfont' in version
`normal'
(Font)                  T1/Merriwthr-OsF/m/it --> T1/Merriwthr-OsF/m/it
on inpu
t line 2140.
LaTeX Font Info:    Overwriting symbol font `mtletterfont' in version
`bold'
(Font)                  T1/Merriwthr-OsF/m/it --> T1/Merriwthr-OsF/b/it
on inpu
t line 2140.
LaTeX Font Info:    Overwriting symbol font `mtoperatorfont' in version
`normal
'
(Font)                  T1/Merriwthr-OsF/m/n --> T1/Merriwthr-OsF/m/n on
input
line 2140.
LaTeX Font Info:    Overwriting symbol font `mtoperatorfont' in version
`bold'
(Font)                  T1/Merriwthr-OsF/m/n --> T1/Merriwthr-OsF/b/n on
input
line 2140.
LaTeX Font Info:    Overwriting math alphabet `\Mathbf' in version
`normal'
(Font)                  T1/Merriwthr-OsF/b/n --> T1/Merriwthr-OsF/b/n on
input
line 2140.
LaTeX Font Info:    Overwriting math alphabet `\Mathbf' in version `bold'
(Font)                  T1/Merriwthr-OsF/b/n --> T1/Merriwthr-OsF/b/n on
input
line 2140.
LaTeX Font Info:    Overwriting math alphabet `\Mathit' in version
`normal'
(Font)                  T1/Merriwthr-OsF/m/it --> T1/Merriwthr-OsF/m/it
on inpu
t line 2140.
LaTeX Font Info:    Overwriting math alphabet `\Mathit' in version `bold'
(Font)                  T1/Merriwthr-OsF/m/it --> T1/Merriwthr-OsF/b/it
on inpu
t line 2140.
LaTeX Font Info:    Overwriting math alphabet `\Mathsf' in version
`normal'
(Font)                  T1/MerriwthrSans-OsF/m/n --> T1/MerriwthrSans-
OsF/m/n o
n input line 2140.
LaTeX Font Info:    Overwriting math alphabet `\Mathsf' in version `bold'
(Font)                  T1/MerriwthrSans-OsF/m/n --> T1/MerriwthrSans-
OsF/b/n o
n input line 2140.
LaTeX Font Info:    Overwriting math alphabet `\Mathtt' in version
`normal'
(Font)                  T1/lmtt/m/n --> T1/lmtt/m/n on input line 2140.
LaTeX Font Info:    Overwriting math alphabet `\Mathtt' in version `bold'
(Font)                  T1/lmtt/m/n --> T1/lmtt/b/n on input line 2140.
** Latin letters in the normal (resp. bold) math versions are now
```

```
** set up to use the fonts T1/Merriwthr-OsF/m(b)/it
** Other characters (digits, ...) and \log-like names will be
** typeset with the n shape.
** \hbar
** minus as endash
** \HUGE has been (re)-defined.
** mathastext has declared larger sizes for subscripts.
** To keep LaTeX defaults, use option `defaultmathsizes'.
) (c:/TeXLive/2022/texmf-dist/tex/latex/relsize/relsize.sty
Package: relsize 2013/03/29 ver 4.1
) (c:/TeXLive/2022/texmf-dist/tex/latex/ragged2e/ragged2e.sty
Package: ragged2e 2021/12/15 v3.1 ragged2e Package
\CenteringLeftskip=\skip50
\RaggedLeftLeftskip=\skip51
\RaggedRightLeftskip=\skip52
\CenteringRightskip=\skip53
\RaggedLeftRightskip=\skip54
\RaggedRightRightskip=\skip55
\CenteringParfillskip=\skip56
\RaggedLeftParfillskip=\skip57
\RaggedRightParfillskip=\skip58
\JustifyingParfillskip=\skip59
\CenteringParindent=\skip60
\RaggedLeftParindent=\skip61
\RaggedRightParindent=\skip62
\JustifyingParindent=\skip63
) (c:/TeXLive/2022/texmf-dist/tex/latex/xcolor/xcolor.sty
Package: xcolor 2022/06/12 v2.14 LaTeX color extensions (UK)
(c:/TeXLive/2022/texmf-dist/tex/latex/graphics-cfg/color.cfg
File: color.cfg 2016/01/02 v1.6 sample color configuration
)
Package xcolor Info: Driver file: pdftex.def on input line 227.
(c:/TeXLive/2022/texmf-dist/tex/latex/graphics-def/pdftex.def
File: pdftex.def 2022/09/22 v1.2b Graphics/color driver for pdftex
\stockwidth=\dimen140
\stockheight=\dimen141
) (c:/TeXLive/2022/texmf-dist/tex/latex/graphics/mathcolor.ltx)
Package xcolor Info: Model `cmy' substituted by `cmy0' on input line
1353.
Package xcolor Info: Model `hsb' substituted by `rgb' on input line 1357.
Package xcolor Info: Model `RGB' extended on input line 1369.
Package xcolor Info: Model `HTML' substituted by `rgb' on input line
1371.
Package xcolor Info: Model `Hsb' substituted by `hsb' on input line 1372.
Package xcolor Info: Model `tHsb' substituted by `hsb' on input line
1373.
Package xcolor Info: Model `HSB' substituted by `hsb' on input line 1374.
Package xcolor Info: Model `Gray' substituted by `gray' on input line
1375.
Package xcolor Info: Model `wave' substituted by `hsb' on input line
1376.
) (c:/TeXLive/2022/texmf-dist/tex/latex/colortbl/colortbl.sty
Package: colortbl 2022/06/20 v1.0f Color table columns (DPC)
(c:/TeXLive/2022/texmf-dist/tex/latex/tools/array.sty
```

```
Package: array 2022/03/10 v2.5f Tabular extension package (FMi)
\col@sep=\dimen142
\ar@mcellbox=\box52
\extrarowheight=\dimen143
\NC@list=\toks26
\extratabsurround=\skip64
\backup@length=\skip65
\ar@cellbox=\box53
)
\everycr=\toks27
\minrowclearance=\skip66
\rownum=\count197
) (c:/TeXLive/2022/texmf-dist/tex/latex/graphics/graphicx.sty
Package: graphicx 2021/09/16 v1.2d Enhanced LaTeX Graphics (DPC,SPQR)
(c:/TeXLive/2022/texmf-dist/tex/latex/graphics/graphics.sty
Package: graphics 2022/03/10 v1.4e Standard LaTeX Graphics (DPC,SPQR)
(c:/TeXLive/2022/texmf-dist/tex/latex/graphics/trig.sty
Package: trig 2021/08/11 v1.11 sin cos tan (DPC)
) (c:/TeXLive/2022/texmf-dist/tex/latex/graphics-cfg/graphics.cfg
File: graphics.cfg 2016/06/04 v1.11 sample graphics configuration
)
Package graphics Info: Driver file: pdftex.def on input line 107.
)
\Gin@req@height=\dimen144
\Gin@req@width=\dimen145
) (c:/TeXLive/2022/texmf-dist/tex/latex/xpatch/xpatch.sty
(c:/TeXLive/2022/texm
f-dist/tex/latex/l3kernel/expl3.sty
Package: expl3 2022-10-26 L3 programming layer (loader)
(c:/TeXLive/2022/texmf-dist/tex/latex/l3backend/l3backend-pdftex.def
File: l3backend-pdftex.def 2022-10-26 L3 backend support: PDF output
(pdfTeX)
\l__color_backend_stack_int=\count198
\l__pdf_internal_box=\box54
))
Package: xpatch 2020/03/25 v0.3a Extending etoolbox patching commands
(c:/TeXLive/2022/texmf-dist/tex/latex/l3packages/xparse/xparse.sty
Package: xparse 2022-06-22 L3 Experimental document command parser
)) (c:/TeXLive/2022/texmf-dist/tex/latex/environ/environ.sty
Package: environ 2014/05/04 v0.3 A new way to define environments
(c:/TeXLive/2022/texmf-dist/tex/latex/trimspaces/trimspaces.sty
Package: trimspaces 2009/09/17 v1.1 Trim spaces around a token list
)
\@envbody=\toks28
) (c:/TeXLive/2022/texmf-dist/tex/latex/lastpage/lastpage.sty
Package: lastpage 2021/09/03 v1.2n Refers to last page's name (HMM; JPG)
) (c:/TeXLive/2022/texmf-dist/tex/latex/graphics/rotating.sty
Package: rotating 2016/08/11 v2.16d rotated objects in LaTeX
(c:/TeXLive/2022/texmf-dist/tex/latex/base/ifthen.sty
Package: ifthen 2022/04/13 v1.1d Standard LaTeX ifthen package (DPC)
)
\c@r@tfl@t=\count199
\rotFPtop=\skip67
\rotFPbot=\skip68
```

```
\rot@float@box=\box55
\rot@mess@toks=\toks29
) (c:/TeXLive/2022/texmf-dist/tex/latex/graphics/lscape.sty
Package: lscape 2020/05/28 v3.02 Landscape Pages (DPC)
) (c:/TeXLive/2022/texmf-dist/tex/latex/tools/afterpage.sty
Package: afterpage 2014/10/28 v1.08 After-Page Package (DPC)
\AP@output=\toks30
\AP@partial=\box56
\AP@footins=\box57
) (c:/TeXLive/2022/texmf-dist/tex/latex/textpos/textpos.sty
Package: textpos 2022/07/23 v1.10.1
Package textpos Info: choosing support for LaTeX3 on input line 60.
\TP@textbox=\box58
\TP@holdbox=\box59
\TPHorizModule=\dimen146
\TPVertModule=\dimen147
\TP@margin=\dimen148
\TP@absmargin=\dimen149
Grid set 16 x 16 = 37.34424pt x 52.81541pt
\TPboxrulesize=\dimen150
\TP@ox=\dimen151
\TP@oy=\dimen152
\TP@tbargs=\toks31
TextBlockOrigin set to 0pt x 0pt
) (c:/TeXLive/2022/texmf-dist/tex/latex/url/url.sty
\Urlmuskip=\muskip19
Package: url 2013/09/16  ver 3.4  Verb mode for urls, etc.
) (c:/TeXLive/2022/texmf-dist/tex/latex/newfloat/newfloat.sty
Package: newfloat 2019/09/02 v1.1l Defining new floating environments
(AR)
Package newfloat Info: `rotating' package detected.
) (c:/TeXLive/2022/texmf-dist/tex/latex/mdframed/mdframed.sty
Package: mdframed 2013/07/01 1.9b: mdframed
(c:/TeXLive/2022/texmf-dist/tex/latex/kvoptions/kvoptions.sty
Package: kvoptions 2022-06-15 v3.15 Key value format for package options
(HO)
(c:/TeXLive/2022/texmf-dist/tex/generic/ltxcmds/ltxcmds.sty
Package: ltxcmds 2020-05-10 v1.25 LaTeX kernel commands for general use
(HO)
) (c:/TeXLive/2022/texmf-dist/tex/latex/kvsetkeys/kvsetkeys.sty
Package: kvsetkeys 2022-10-05 v1.19 Key value parser (HO)
)) (c:/TeXLive/2022/texmf-dist/tex/latex/zref/zref-abspage.sty
Package: zref-abspage 2022-04-07 v2.34 Module abspage for zref (HO)
(c:/TeXLive/2022/texmf-dist/tex/latex/zref/zref-base.sty
Package: zref-base 2022-04-07 v2.34 Module base for zref (HO)
(c:/TeXLive/2022/texmf-dist/tex/generic/infwarerr/infwarerr.sty
Package: infwarerr 2019/12/03 v1.5 Providing info/warning/error messages
(HO)
) (c:/TeXLive/2022/texmf-dist/tex/generic/kvdefinekeys/kvdefinekeys.sty
Package: kvdefinekeys 2019-12-19 v1.6 Define keys (HO)
) (c:/TeXLive/2022/texmf-dist/tex/generic/pdftexcmds/pdftexcmds.sty
Package: pdftexcmds 2020-06-27 v0.33 Utility functions of pdfTeX for
LuaTeX (HO
)
```

```
Package pdftexcmds Info: \pdf@primitive is available.
Package pdftexcmds Info: \pdf@ifprimitive is available.
Package pdftexcmds Info: \pdfdraftmode found.
) (c:/TeXLive/2022/texmf-dist/tex/generic/etexcmds/etexcmds.sty
Package: etexcmds 2019/12/15 v1.7 Avoid name clashes with e-TeX commands
(HO)
) (c:/TeXLive/2022/texmf-dist/tex/latex/auxhook/auxhook.sty
Package: auxhook 2019-12-17 v1.6 Hooks for auxiliary files (HO)
)
Package zref Info: New property list: main on input line 767.
Package zref Info: New property: default on input line 768.
Package zref Info: New property: page on input line 769.
) (c:/TeXLive/2022/texmf-dist/tex/latex/base/atbegshi-ltx.sty
Package: atbegshi-ltx 2021/01/10 v1.0c Emulation of the original atbegshi
package with kernel methods
)
\c@abspage=\count266
Package zref Info: New property: abspage on input line 65.
) (c:/TeXLive/2022/texmf-dist/tex/latex/needspace/needspace.sty
Package: needspace 2010/09/12 v1.3d reserve vertical space
)
\mdf@templength=\skip69
\c@mdf@globalstyle@cnt=\count267
\mdf@skipabove@length=\skip70
\mdf@skipbelow@length=\skip71
\mdf@leftmargin@length=\skip72
\mdf@rightmargin@length=\skip73
\mdf@innerleftmargin@length=\skip74
\mdf@innerrightmargin@length=\skip75
\mdf@innertopmargin@length=\skip76
\mdf@innerbottommargin@length=\skip77
\mdf@splittopskip@length=\skip78
\mdf@splitbottomskip@length=\skip79
\mdf@outermargin@length=\skip80
\mdf@innermargin@length=\skip81
\mdf@linewidth@length=\skip82
\mdf@innerlinewidth@length=\skip83
\mdf@middlelinewidth@length=\skip84
\mdf@outerlinewidth@length=\skip85
\mdf@roundcorner@length=\skip86
\mdf@footenotedistance@length=\skip87
\mdf@userdefinedwidth@length=\skip88
\mdf@needspace@length=\skip89
\mdf@frametitleaboveskip@length=\skip90
\mdf@frametitlebelowskip@length=\skip91
\mdf@frametitlerulewidth@length=\skip92
\mdf@frametitleleftmargin@length=\skip93
\mdf@frametitlerightmargin@length=\skip94
\mdf@shadowsize@length=\skip95
\mdf@extratopheight@length=\skip96
\mdf@subtitleabovelinewidth@length=\skip97
\mdf@subtitlebelowlinewidth@length=\skip98
\mdf@subtitleaboveskip@length=\skip99
\mdf@subtitlebelowskip@length=\skip100
```

```
\mdf@subtitleinneraboveskip@length=\skip101
\mdf@subtitleinnerbelowskip@length=\skip102
\mdf@subsubtitleabovelinewidth@length=\skip103
\mdf@subsubtitlebelowlinewidth@length=\skip104
\mdf@subsubtitleaboveskip@length=\skip105
\mdf@subsubtitlebelowskip@length=\skip106
\mdf@subsubtitleinneraboveskip@length=\skip107
\mdf@subsubtitleinnerbelowskip@length=\skip108
(c:/TeXLive/2022/texmf-dist/tex/latex/mdframed/md-frame-0.mdf
File: md-frame-0.mdf 2013/07/01\ 1.9b: md-frame-0
)
\mdf@frametitlebox=\box60
\mdf@footnotebox=\box61
\mdf@splitbox@one=\box62
\mdf@splitbox@two=\box63
\mdf@splitbox@save=\box64
\mdfsplitboxwidth=\skip109
\mdfsplitboxtotalwidth=\skip110
\mdfsplitboxheight=\skip111
\mdfsplitboxdepth=\skip112
\mdfsplitboxtotalheight=\skip113
\mdfframetitleboxwidth=\skip114
\mdfframetitleboxtotalwidth=\skip115
\mdfframetitleboxheight=\skip116
\mdfframetitleboxdepth=\skip117
\mdfframetitleboxtotalheight=\skip118
\mdffootnoteboxwidth=\skip119
\mdffootnoteboxtotalwidth=\skip120
\mdffootnoteboxheight=\skip121
\mdffootnoteboxdepth=\skip122
\mdffootnoteboxtotalheight=\skip123
\mdftotallinewidth=\skip124
\mdfboundingboxwidth=\skip125
\mdfboundingboxtotalwidth=\skip126
\mdfboundingboxheight=\skip127
\mdfboundingboxdepth=\skip128
\mdfboundingboxtotalheight=\skip129
\mdf@freevspace@length=\skip130
\mdf@horizontalwidthofbox@length=\skip131
\mdf@verticalmarginwhole@length=\skip132
\mdf@horizontalspaceofbox=\skip133
\mdfsubtitleheight=\skip134
\mdfsubsubtitleheight=\skip135
\c@mdfcountframes=\count268

****** mdframed patching \endmdf@trivlist

****** -- success******

\mdf@envdepth=\count269
\c@mdf@env@i=\count270
\c@mdf@env@ii=\count271
\c@mdf@zref@counter=\count272
Package zref Info: New property: mdf@pagevalue on input line 895.
```

```
) (c:/TeXLive/2022/texmf-dist/tex/latex/titlesec/titlesec.sty
Package: titlesec 2021/07/05 v2.14 Sectioning titles
\ttl@box=\box65
\beforetitleunit=\skip136
\aftertitleunit=\skip137
\ttl@plus=\dimen153
\ttl@minus=\dimen154
\ttl@toksa=\toks32
\titlewidth=\dimen155
\titlewidthlast=\dimen156
\titlewidthfirst=\dimen157
) (c:/TeXLive/2022/texmf-dist/tex/latex/koma-script/scrextend.sty
Package: scrextend 2022/10/12 v3.38 KOMA-Script package (extend other
classes w
ith features of KOMA-Script classes)
(c:/TeXLive/2022/texmf-dist/tex/latex/koma-script/scrkbase.sty
Package: scrkbase 2022/10/12 v3.38 KOMA-Script package (KOMA-Script-
dependent b
asics and keyval usage)
(c:/TeXLive/2022/texmf-dist/tex/latex/koma-script/scrbase.sty
Package: scrbase 2022/10/12 v3.38 KOMA-Script package (KOMA-Script-
independent
basics and keyval usage)
(c:/TeXLive/2022/texmf-dist/tex/latex/koma-script/scrlfile.sty
Package: scrlfile 2022/10/12 v3.38 KOMA-Script package (file load hooks)
(c:/TeXLive/2022/texmf-dist/tex/latex/koma-script/scrlfile-hook.sty
Package: scrlfile-hook 2022/10/12 v3.38 KOMA-Script package (using LaTeX
hooks)

(c:/TeXLive/2022/texmf-dist/tex/latex/koma-script/scrlogo.sty
Package: scrlogo 2022/10/12 v3.38 KOMA-Script package (logo)
)))
Applying: [2021/05/01] Usage of raw or classic option list on input line
252.
Already applied: [0000/00/00] Usage of raw or classic option list on
input line
 368.
))
Package scrextend Info: unexpected definition of `\@makefnmark'.
(scrextend)             Trying to patch it on input line 1709.
Package scrextend Info: patch seems to be successfull on input line 1709.
)

LaTeX Font Warning: Font shape `T1/cmr/m/n' in size <7.5> not available
(Font)              size <7> substituted on input line 65.

(c:/TeXLive/2022/texmf-dist/tex/latex/tools/calc.sty
Package: calc 2017/05/25 v4.3 Infix arithmetic (KKT,FJ)
\calc@Acount=\count273
\calc@Bcount=\count274
\calc@Adimen=\dimen158
\calc@Bdimen=\dimen159
\calc@Askip=\skip138
\calc@Bskip=\skip139
```

```
LaTeX Info: Redefining \setlength on input line 80.
LaTeX Info: Redefining \addtolength on input line 81.
\calc@Ccount=\count275
\calc@Cskip=\skip140
) (c:/TeXLive/2022/texmf-dist/tex/latex/geometry/geometry.sty
Package: geometry 2020/01/02 v5.9 Page Geometry
(c:/TeXLive/2022/texmf-dist/tex/generic/iftex/ifvtex.sty
Package: ifvtex 2019/10/25 v1.7 ifvtex legacy package. Use iftex instead.
)
\Gm@cnth=\count276
\Gm@cntv=\count277
\c@Gm@tempcnt=\count278
\Gm@bindingoffset=\dimen160
\Gm@wd@mp=\dimen161
\Gm@odd@mp=\dimen162
\Gm@even@mp=\dimen163
\Gm@layoutwidth=\dimen164
\Gm@layoutheight=\dimen165
\Gm@layouthoffset=\dimen166
\Gm@layoutvoffset=\dimen167
\Gm@dimlist=\toks33
) (c:/TeXLive/2022/texmf-dist/tex/latex/hyperref/hyperref.sty
Package: hyperref 2022-09-22 v7.00t Hypertext links for LaTeX
(c:/TeXLive/2022/texmf-dist/tex/generic/pdfescape/pdfescape.sty
Package: pdfescape 2019/12/09 v1.15 Implements pdfTeX's escape features
(HO)
) (c:/TeXLive/2022/texmf-dist/tex/latex/hycolor/hycolor.sty
Package: hycolor 2020-01-27 v1.10 Color options for hyperref/bookmark
(HO)
) (c:/TeXLive/2022/texmf-dist/tex/latex/letltxmacro/letltxmacro.sty
Package: letltxmacro 2019/12/03 v1.6 Let assignment for LaTeX macros (HO)
) (c:/TeXLive/2022/texmf-dist/tex/latex/hyperref/nameref.sty
Package: nameref 2022-05-17 v2.50 Cross-referencing by name of section
(c:/TeXLive/2022/texmf-dist/tex/latex/refcount/refcount.sty
Package: refcount 2019/12/15 v3.6 Data extraction from label references
(HO)
) (c:/TeXLive/2022/texmf-
dist/tex/generic/gettitlestring/gettitlestring.sty
Package: gettitlestring 2019/12/15 v1.6 Cleanup title references (HO)
)
\c@section@level=\count279
)
\@linkdim=\dimen168
\Hy@linkcounter=\count280
\Hy@pagecounter=\count281
(c:/TeXLive/2022/texmf-dist/tex/latex/hyperref/pd1enc.def
File: pd1enc.def 2022-09-22 v7.00t Hyperref: PDFDocEncoding definition
(HO)
Now handling font encoding PD1 ...
... no UTF-8 mapping file for font encoding PD1
) (c:/TeXLive/2022/texmf-dist/tex/generic/intcalc/intcalc.sty
Package: intcalc 2019/12/15 v1.3 Expandable calculations with integers
(HO)
)
```

```
\Hy@SavedSpaceFactor=\count282
(c:/TeXLive/2022/texmf-dist/tex/latex/hyperref/puenc.def
File: puenc.def 2022-09-22 v7.00t Hyperref: PDF Unicode definition (HO)
Now handling font encoding PU ...
... no UTF-8 mapping file for font encoding PU
)
Package hyperref Info: Option `colorlinks' set `true' on input line 4045.
Package hyperref Info: Hyper figures OFF on input line 4162.
Package hyperref Info: Link nesting OFF on input line 4167.
Package hyperref Info: Hyper index ON on input line 4170.
Package hyperref Info: Plain pages OFF on input line 4177.
Package hyperref Info: Backreferencing OFF on input line 4182.
Package hyperref Info: Implicit mode ON; LaTeX internals redefined.
Package hyperref Info: Bookmarks ON on input line 4410.
\c@Hy@tempcnt=\count283
LaTeX Info: Redefining \url on input line 4748.
\XeTeXLinkMargin=\dimen169
(c:/TeXLive/2022/texmf-dist/tex/generic/bitset/bitset.sty
Package: bitset 2019/12/09 v1.3 Handle bit-vector datatype (HO)
(c:/TeXLive/2022/texmf-dist/tex/generic/bigintcalc/bigintcalc.sty
Package: bigintcalc 2019/12/15 v1.5 Expandable calculations on big
integers (HO
)
))
\Fld@menulength=\count284
\Field@Width=\dimen170
\Fld@charsize=\dimen171
Package hyperref Info: Hyper figures OFF on input line 6027.
Package hyperref Info: Link nesting OFF on input line 6032.
Package hyperref Info: Hyper index ON on input line 6035.
Package hyperref Info: backreferencing OFF on input line 6042.
Package hyperref Info: Link coloring ON on input line 6045.
Package hyperref Info: Link coloring with OCG OFF on input line 6052.
Package hyperref Info: PDF/A mode OFF on input line 6057.
\Hy@abspage=\count285
\c@Item=\count286
\c@Hfootnote=\count287
)
Package hyperref Info: Driver (autodetected): hpdftex.
(c:/TeXLive/2022/texmf-dist/tex/latex/hyperref/hpdftex.def
File: hpdftex.def 2022-09-22 v7.00t Hyperref driver for pdfTeX
(c:/TeXLive/2022/texmf-dist/tex/latex/base/atveryend-ltx.sty
Package: atveryend-ltx 2020/08/19 v1.0a Emulation of the original
atveryend pac
kage
with kernel methods
)
\HyAnn@Count=\count288
\Fld@listcount=\count289
\c@bookmark@seq@number=\count290
(c:/TeXLive/2022/texmf-dist/tex/latex/rerunfilecheck/rerunfilecheck.sty
Package: rerunfilecheck 2022-07-10 v1.10 Rerun checks for auxiliary files
(HO)
(c:/TeXLive/2022/texmf-dist/tex/generic/uniquecounter/uniquecounter.sty
```

```
Package: uniquecounter 2019/12/15 v1.4 Provide unlimited unique counter
(HO)
)
Package uniquecounter Info: New unique counter `rerunfilecheck' on input
line 2
85.
)
\Hy@SectionHShift=\skip141
) (c:/TeXLive/2022/texmf-dist/tex/latex/preprint/authblk.sty
Package: authblk 2001/02/27 1.3 (PWD)
\affilsep=\skip142
\@affilsep=\skip143
\c@Maxaffil=\count291
\c@authors=\count292
\c@affil=\count293
) (c:/TeXLive/2022/texmf-dist/tex/latex/footmisc/footmisc.sty
Package: footmisc 2022/03/08 v6.0d a miscellany of footnote facilities
\FN@temptoken=\toks34
\footnotemargin=\dimen172
\@outputbox@depth=\dimen173
Package footmisc Info: Declaring symbol style bringhurst on input line
695.
Package footmisc Info: Declaring symbol style chicago on input line 703.
Package footmisc Info: Declaring symbol style wiley on input line 712.
Package footmisc Info: Declaring symbol style lamport-robust on input
line 723.

Package footmisc Info: Declaring symbol style lamport* on input line 743.
Package footmisc Info: Declaring symbol style lamport*-robust on input
line 764
.
) (c:/TeXLive/2022/texmf-dist/tex/latex/fancyhdr/fancyhdr.sty
Package: fancyhdr 2022/05/18 v4.0.3 Extensive control of page headers and
foote
rs
\f@nch@headwidth=\skip144
\f@nch@O@elh=\skip145
\f@nch@O@erh=\skip146
\f@nch@O@olh=\skip147
\f@nch@O@orh=\skip148
\f@nch@O@elf=\skip149
\f@nch@O@erf=\skip150
\f@nch@O@olf=\skip151
\f@nch@O@orf=\skip152
) (c:/TeXLive/2022/texmf-dist/tex/generic/alphalph/alphalph.sty
Package: alphalph 2019/12/09 v2.6 Convert numbers to letters (HO)
)
\c@authorfn=\count294
(c:/TeXLive/2022/texmf-dist/tex/latex/abstract/abstract.sty
Package: abstract 2009/06/08 v1.2a configurable abstracts
\abstitleskip=\skip153
\absleftindent=\skip154
\absrightindent=\skip155
\absparindent=\skip156
```

```
\absparsep=\skip157
)
Package newfloat Info: New float `keypoints' with options
`placement=t!,name=kp
t' on input line 286.
\c@keypoints=\count295
\newfloat@ftype=\count296
Package newfloat Info: float type `keypoints'=8 on input line 286.
(c:/TeXLive/2022/texmf-dist/tex/latex/enumitem/enumitem.sty
Package: enumitem 2019/06/20 v3.9 Customized lists
\labelindent=\skip158
\enit@outerparindent=\dimen174
\enit@toks=\toks35
\enit@inbox=\box66
\enit@count@id=\count297
\enitdp@description=\count298
) (c:/TeXLive/2022/texmf-dist/tex/latex/quoting/quoting.sty
Package: quoting 2014/01/28 v0.1c Consolidated environment for displayed
text
\quo@toppartop=\skip159
) (c:/TeXLive/2022/texmf-dist/tex/latex/sttools/stfloats.sty
Package: stfloats 2017/03/27 v3.3 Improve float mechanism and
baselineskip sett
ings
\@dblbotnum=\count299
\c@dblbotnumber=\count300
) (c:/TeXLive/2022/texmf-dist/tex/latex/booktabs/booktabs.sty
Package: booktabs 2020/01/12 v1.61803398 Publication quality tables
\heavyrulewidth=\dimen175
\lightrulewidth=\dimen176
\cmidrulewidth=\dimen177
\belowrulesep=\dimen178
\belowbottomsep=\dimen179
\aboverulesep=\dimen180
\abovetopsep=\dimen181
\cmidrulesep=\dimen182
\cmidrulekern=\dimen183
\defaultaddspace=\dimen184
\@cmidla=\count301
\@cmidlb=\count302
\@aboverulesep=\dimen185
\@belowrulesep=\dimen186
\@thisruleclass=\count303
\@lastruleclass=\count304
\@thisrulewidth=\dimen187
) (c:/TeXLive/2022/texmf-dist/tex/latex/tools/tabularx.sty
Package: tabularx 2020/01/15 v2.11c `tabularx' package (DPC)
\TX@col@width=\dimen188
\TX@old@table=\dimen189
\TX@old@col=\dimen190
\TX@target=\dimen191
\TX@delta=\dimen192
\TX@cols=\count305
\TX@ftn=\toks36
```

```
)
\enitdp@tablenotes=\count306
(c:/TeXLive/2022/texmf-dist/tex/latex/caption/caption.sty
Package: caption 2022/03/01 v3.6b Customizing captions (AR)
(c:/TeXLive/2022/texmf-dist/tex/latex/caption/caption3.sty
Package: caption3 2022/03/17 v2.3b caption3 kernel (AR)
\caption@tempdima=\dimen193
\captionmargin=\dimen194
\caption@leftmargin=\dimen195
\caption@rightmargin=\dimen196
\caption@width=\dimen197
\caption@indent=\dimen198
\caption@parindent=\dimen199
\caption@hangindent=\dimen256
Package caption Info: Standard document class detected.
)
\c@caption@flags=\count307
\c@continuedfloat=\count308
Package caption Info: hyperref package is loaded.
Package caption Info: rotating package is loaded.
) (c:/TeXLive/2022/texmf-dist/tex/latex/natbib/natbib.sty
Package: natbib 2010/09/13 8.31b (PWD, AO)
\bibhang=\skip160
\bibsep=\skip161
LaTeX Info: Redefining \cite on input line 694.
\c@NAT@ctr=\count309
)) (c:/TeXLive/2022/texmf-dist/tex/latex/tools/enumerate.sty
Package: enumerate 2015/07/23 v3.00 enumerate extensions (DPC)
\@enLab=\toks37
) (c:/TeXLive/2022/texmf-dist/tex/latex/amsmath/amsmath.sty
Package: amsmath 2022/04/08 v2.17n AMS math features
\@mathmargin=\skip162
For additional information on amsmath, use the `?' option.
(c:/TeXLive/2022/texmf-dist/tex/latex/amsmath/amstext.sty
Package: amstext 2021/08/26 v2.01 AMS text
(c:/TeXLive/2022/texmf-dist/tex/latex/amsmath/amsgen.sty
File: amsgen.sty 1999/11/30 v2.0 generic functions
\@emptytoks=\toks38
\ex@=\dimen257
)) (c:/TeXLive/2022/texmf-dist/tex/latex/amsmath/amsbsy.sty
Package: amsbsy 1999/11/29 v1.2d Bold Symbols
\pmbraise@=\dimen258
) (c:/TeXLive/2022/texmf-dist/tex/latex/amsmath/amsopn.sty
Package: amsopn 2022/04/08 v2.04 operator names
)
\inf@bad=\count310
LaTeX Info: Redefining \frac on input line 234.
\uproot@=\count311
\leftroot@=\count312
LaTeX Info: Redefining \overline on input line 399.
LaTeX Info: Redefining \colon on input line 410.
\classnum@=\count313
\DOTSCASE@=\count314
LaTeX Info: Redefining \ldots on input line 496.
```

```
LaTeX Info: Redefining \dots on input line 499.
LaTeX Info: Redefining \cdots on input line 620.
\Mathstrutbox@=\box67
\strutbox@=\box68
LaTeX Info: Redefining \big on input line 722.
LaTeX Info: Redefining \Big on input line 723.
LaTeX Info: Redefining \bigg on input line 724.
LaTeX Info: Redefining \Bigg on input line 725.
\big@size=\dimen259
LaTeX Font Info:    Redeclaring font encoding OML on input line 743.
LaTeX Font Info:    Redeclaring font encoding OMS on input line 744.
\macc@depth=\count315
LaTeX Info: Redefining \bmod on input line 905.
LaTeX Info: Redefining \pmod on input line 910.
LaTeX Info: Redefining \smash on input line 940.
LaTeX Info: Redefining \relbar on input line 970.
LaTeX Info: Redefining \Relbar on input line 971.
\c@MaxMatrixCols=\count316
\dotsspace@=\muskip20
\c@parentequation=\count317
\dspbrk@lvl=\count318
\tag@help=\toks39
\row@=\count319
\column@=\count320
\maxfields@=\count321
\andhelp@=\toks40
\eqnshift@=\dimen260
\alignsep@=\dimen261
\tagshift@=\dimen262
\tagwidth@=\dimen263
\totwidth@=\dimen264
\lineht@=\dimen265
\@envbody=\toks41
\multlinegap=\skip163
\multlinetaggap=\skip164
\mathdisplay@stack=\toks42
LaTeX Info: Redefining \[ on input line 2953.
LaTeX Info: Redefining \] on input line 2954.
) (c:/TeXLive/2022/texmf-dist/tex/latex/tools/bm.sty
Package: bm 2022/01/05 v1.2f Bold Symbol Support (DPC/FMi)
\symboldoperators=\mathgroup10
\symboldletters=\mathgroup11
\symboldsymbols=\mathgroup12
Package bm Info: No bold for \OMX/cmex/m/n, using \pmb.
\symboldEulerFraktur=\mathgroup13
Package bm Info: No bold for \U/euex/m/n, using \pmb.
LaTeX Font Info:    Redeclaring math alphabet \mathbf on input line 149.
) (c:/TeXLive/2022/texmf-dist/tex/latex/amsfonts/amsfonts.sty
Package: amsfonts 2013/01/14 v3.01 Basic AMSFonts support
\symAMSa=\mathgroup14
\symAMSb=\mathgroup15
LaTeX Font Info:    Redeclaring math symbol \hbar on input line 98.
LaTeX Info: Redefining \frak on input line 111.
) (c:/TeXLive/2022/texmf-dist/tex/latex/algorithms/algorithm.sty
```

```
Package: algorithm 2009/08/24 v0.1 Document Style `algorithm' - floating
enviro
nment
(c:/TeXLive/2022/texmf-dist/tex/latex/float/float.sty
Package: float 2001/11/08 v1.3d Float enhancements (AL)
\c@float@type=\count322
\float@exts=\toks43
\float@box=\box69
\@float@everytoks=\toks44
\@floatcapt=\box70
)
\@float@every@algorithm=\toks45
\c@algorithm=\count323
) (c:/TeXLive/2022/texmf-dist/tex/latex/algorithmicx/algpseudocode.sty
Package: algpseudocode
(c:/TeXLive/2022/texmf-dist/tex/latex/algorithmicx/algorithmicx.sty
Package: algorithmicx 2005/04/27 v1.2 Algorithmicx
Document Style algorithmicx 1.2 - a greatly improved `algorithmic' style
\c@ALG@line=\count324
\c@ALG@rem=\count325
\c@ALG@nested=\count326
\ALG@tlm=\skip165
\ALG@thistlm=\skip166
\c@ALG@Lnr=\count327
\c@ALG@blocknr=\count328
\c@ALG@storecount=\count329
\c@ALG@tmpcounter=\count330
\ALG@tmplength=\skip167
)
Document Style - pseudocode environments for use with the `algorithmicx'
style
) (c:/TeXLive/2022/texmf-dist/tex/latex/xurl/xurl.sty
Package: xurl 2022/01/09 v 0.10 modify URL breaks
) (c:/TeXLive/2022/texmf-dist/tex/latex/lineno/lineno.sty
Package: lineno 2005/11/02 line numbers on paragraphs v4.41
\linenopenalty=\count331
\output=\toks46
\linenoprevgraf=\count332
\linenumbersep=\dimen266
\linenumberwidth=\dimen267
\c@linenumber=\count333
\c@pagewiselinenumber=\count334
\c@LN@truepage=\count335
\c@internallinenumber=\count336
\c@internallinenumbers=\count337
\quotelinenumbersep=\dimen268
\bframerule=\dimen269
\bframesep=\dimen270
\bframebox=\box71
LaTeX Info: Redefining \\ on input line 3056.
)
\c@example=\count338
\c@remark=\count339
\c@definition=\count340
```

```
(c:/TeXLive/2022/texmf-dist/tex/latex/siunitx/siunitx.sty
Package: siunitx 2022-10-23 v3.1.9 A comprehensive (SI) units package
\l__siunitx_angle_tmp_dim=\dimen271
\l__siunitx_angle_marker_box=\box72
\l__siunitx_angle_unit_box=\box73
\l__siunitx_compound_count_int=\count341
(c:/TeXLive/2022/texmf-dist/tex/latex/translations/translations.sty
Package: translations 2022/02/05 v1.12 internationalization of LaTeX2e
packages
 (CN)
)
\l__siunitx_number_exponent_fixed_int=\count342
\l__siunitx_number_min_decimal_int=\count343
\l__siunitx_number_min_integer_int=\count344
\l__siunitx_number_round_precision_int=\count345
\l__siunitx_number_group_first_int=\count346
\l__siunitx_number_group_size_int=\count347
\l__siunitx_number_group_minimum_int=\count348
\l__siunitx_table_tmp_box=\box74
\l__siunitx_table_tmp_dim=\dimen272
\l__siunitx_table_column_width_dim=\dimen273
\l__siunitx_table_integer_box=\box75
\l__siunitx_table_decimal_box=\box76
\l__siunitx_table_before_box=\box77
\l__siunitx_table_after_box=\box78
\l__siunitx_table_before_dim=\dimen274
\l__siunitx_table_carry_dim=\dimen275
\l__siunitx_unit_tmp_int=\count349
\l__siunitx_unit_position_int=\count350
\l__siunitx_unit_total_int=\count351
)
Package translations Info: No language package found. I am going to use
`englis
h' as default language. on input line 73.
LaTeX Font Info:    Trying to load font information for T1+Merriwthr-OsF
on inp
ut line 73.
(c:/TeXLive/2022/texmf-dist/tex/latex/merriweather/T1Merriwthr-OsF.fd
File: T1Merriwthr-OsF.fd 2020/08/30 (autoinst) Font definitions for
T1/Merriwth
r-OsF.
)
LaTeX Font Info:    Font shape `T1/Merriwthr-OsF/m/n' will be
(Font)              scaled to size 7.5pt on input line 73.
(./main.aux)
\openout1 = `main.aux'.

LaTeX Font Info:    Checking defaults for OML/cmm/m/it on input line 73.
LaTeX Font Info:    ... okay on input line 73.
LaTeX Font Info:    Checking defaults for OMS/cmsy/m/n on input line 73.
LaTeX Font Info:    ... okay on input line 73.
LaTeX Font Info:    Checking defaults for OT1/cmr/m/n on input line 73.
LaTeX Font Info:    ... okay on input line 73.
LaTeX Font Info:    Checking defaults for T1/cmr/m/n on input line 73.
```

```
LaTeX Font Info:    ... okay on input line 73.
LaTeX Font Info:    Checking defaults for TS1/cmr/m/n on input line 73.
LaTeX Font Info:    ... okay on input line 73.
LaTeX Font Info:    Checking defaults for OMX/cmex/m/n on input line 73.
LaTeX Font Info:    ... okay on input line 73.
LaTeX Font Info:    Checking defaults for U/cmr/m/n on input line 73.
LaTeX Font Info:    ... okay on input line 73.
LaTeX Font Info:    Checking defaults for PD1/pdf/m/n on input line 73.
LaTeX Font Info:    ... okay on input line 73.
LaTeX Font Info:    Checking defaults for PU/pdf/m/n on input line 73.
LaTeX Font Info:    ... okay on input line 73.
LaTeX Info: Redefining \microtypecontext on input line 73.
Package microtype Info: Applying patch `item' on input line 73.
Package microtype Info: Applying patch `toc' on input line 73.
Package microtype Info: Applying patch `eqnum' on input line 73.

Package microtype Warning: Unable to apply patch `footnote' on input line
73.

Package microtype Info: Generating PDF output.
Package microtype Info: Character protrusion enabled (level 2).
Package microtype Info: Using default protrusion set `alltext'.
Package microtype Info: Automatic font expansion enabled (level 2),
(microtype)             stretch: 20, shrink: 20, step: 1, non-selected.
Package microtype Info: Using default expansion set `alltext-nott'.
LaTeX Info: Redefining \showhyphens on input line 73.
Package microtype Info: No adjustment of tracking.
Package microtype Info: No adjustment of interword spacing.
Package microtype Info: No adjustment of character kerning.
Package microtype Info: Loading generic protrusion settings for font
family
(microtype)             `Merriwthr-OsF' (encoding: T1).
(microtype)             For optimal results, create family-specific
settings.
(microtype)             See the microtype manual for details.
LaTeX Font Info:    Redeclaring symbol font `operators' on input line 73.
LaTeX Font Info:    Encoding `OT1' has changed to `T1' for symbol font
(Font)              `operators' in the math version `normal' on input
line 73.
LaTeX Font Info:    Overwriting symbol font `operators' in version
`normal'
(Font)                  OT1/cmr/m/n --> T1/Merriwthr-OsF/m/up on input
line 73.

LaTeX Font Info:    Encoding `OT1' has changed to `T1' for symbol font
(Font)              `operators' in the math version `bold' on input line
73.
LaTeX Font Info:    Overwriting symbol font `operators' in version `bold'
(Font)                  OT1/cmr/bx/n --> T1/Merriwthr-OsF/m/up on input
line 73
.
LaTeX Font Info:    Overwriting symbol font `operators' in version `bold'
(Font)                  T1/Merriwthr-OsF/m/up --> T1/Merriwthr-OsF/b/up
on inpu
```

```
t line 73.
LaTeX Font Info:    Redeclaring math alphabet \mathbf on input line 73.
LaTeX Font Info:    Redeclaring math alphabet \mathsf on input line 73.
LaTeX Font Info:    Overwriting math alphabet `\mathsf' in version
`normal'
(Font)                  OT1/cmss/m/n --> T1/MerriwthrSans-OsF/m/up on
input lin
e 73.
LaTeX Font Info:    Overwriting math alphabet `\mathsf' in version `bold'
(Font)                  OT1/cmss/bx/n --> T1/MerriwthrSans-OsF/m/up on
input li
ne 73.
LaTeX Font Info:    Redeclaring math alphabet \mathit on input line 73.
LaTeX Font Info:    Overwriting math alphabet `\mathit' in version
`normal'
(Font)                  OT1/cmr/m/it --> T1/Merriwthr-OsF/m/it on input
line 73
.
LaTeX Font Info:    Overwriting math alphabet `\mathit' in version `bold'
(Font)                  OT1/cmr/bx/it --> T1/Merriwthr-OsF/m/it on input
line 7
3.
LaTeX Font Info:    Redeclaring math alphabet \mathtt on input line 73.
LaTeX Font Info:    Overwriting math alphabet `\mathtt' in version
`normal'
(Font)                  OT1/cmtt/m/n --> T1/lmtt/m/up on input line 73.
LaTeX Font Info:    Overwriting math alphabet `\mathtt' in version `bold'
(Font)                  OT1/cmtt/m/n --> T1/lmtt/m/up on input line 73.
LaTeX Font Info:    Overwriting math alphabet `\mathsf' in version `bold'
(Font)                  T1/MerriwthrSans-OsF/m/up --> T1/MerriwthrSans-
OsF/b/up
 on input line 73.
LaTeX Font Info:    Overwriting math alphabet `\mathit' in version `bold'
(Font)                  T1/Merriwthr-OsF/m/it --> T1/Merriwthr-OsF/b/it
on inpu
t line 73.
\c@mv@tabular=\count352
\c@mv@boldtabular=\count353
Package mathastext Info: current meaning of amsmath \resetMathstrut@
saved on i
nput line 73.
(c:/TeXLive/2022/texmf-dist/tex/context/base/mkii/supp-pdf.mkii
[Loading MPS to PDF converter (version 2006.09.02).]
\scratchcounter=\count354
\scratchdimen=\dimen276
\scratchbox=\box79
\nofMPsegments=\count355
\nofMParguments=\count356
\everyMPshowfont=\toks47
\MPscratchCnt=\count357
\MPscratchDim=\dimen277
\MPnumerator=\count358
\makeMPintoPDFobject=\count359
\everyMPtoPDFconversion=\toks48
```

```
) (c:/TeXLive/2022/texmf-dist/tex/latex/epstopdf-pkg/epstopdf-base.sty
Package: epstopdf-base 2020-01-24 v2.11 Base part for package epstopdf
Package epstopdf-base Info: Redefining graphics rule for `.eps' on input
line 4
85.
(c:/TeXLive/2022/texmf-dist/tex/latex/latexconfig/epstopdf-sys.cfg
File: epstopdf-sys.cfg 2010/07/13 v1.3 Configuration of (r)epstopdf for
TeX Liv
e
))
Package lastpage Info: Please have a look at the pageslts package at
(lastpage)             https://www.ctan.org/pkg/pageslts
(lastpage)             ! on input line 73.
Package newfloat Info: `float' package detected.
*geometry* driver: auto-detecting
*geometry* detected driver: pdftex
*geometry* verbose mode - [ preamble ] result:
* driver: pdftex
* paper: a4paper
* layout: <same size as paper>
* layoutoffset:(h,v)=(0.0pt,0.0pt)
* modes: includefoot twoside
* h-part:(L,W,R)=(54.64pt, 488.22787pt, 54.64pt)
* v-part:(T,H,B)=(66.0pt, 745.04684pt, 34.0pt)
* \paperwidth=597.50787pt
* \paperheight=845.04684pt
* \textwidth=488.22787pt
* \textheight=715.04684pt
* \oddsidemargin=-17.62999pt
* \evensidemargin=-17.62999pt
* \topmargin=-47.76999pt
* \headheight=17.5pt
* \headsep=24.0pt
* \topskip=10.0pt
* \footskip=30.0pt
* \marginparwidth=48.0pt
* \marginparsep=10.0pt
* \columnsep=18.0pt
* \skip\footins=22.0pt plus 2.0pt
* \hoffset=0.0pt
* \voffset=0.0pt
* \mag=1000
* \@twocolumntrue
* \@twosidetrue
* \@mparswitchtrue
* \@reversemarginfalse
* (1in=72.27pt=25.4mm, 1cm=28.453pt)

Package hyperref Info: Link coloring ON on input line 73.
(./main.out) (./main.out)
\@outlinefile=\write3
\openout3 = `main.out'.

\@gscitedetails=\box80
```

```
\@gscitedetailsheight=\skip168
\@gsheadbox=\box81
\@gsheadboxheight=\skip169
LaTeX Font Info:    Font shape `T1/Merriwthr-OsF/b/n' will be
(Font)              scaled to size 6.5pt on input line 73.
LaTeX Font Info:    Calculating math sizes for size <7.5> on input line
73.

LaTeX Font Warning: Font shape `T1/Merriwthr-OsF/m/up' undefined
(Font)              using `T1/Merriwthr-OsF/m/n' instead on input line
73.

LaTeX Font Info:    Font shape `T1/Merriwthr-OsF/m/up' will be
(Font)              scaled to size 6.24973pt on input line 73.
LaTeX Font Info:    Font shape `T1/Merriwthr-OsF/m/up' will be
(Font)              scaled to size 5.24997pt on input line 73.
LaTeX Font Info:    Trying to load font information for U+eur on input
line 73.

(c:/TeXLive/2022/texmf-dist/tex/latex/amsfonts/ueur.fd
File: ueur.fd 2013/01/14 v3.01 Euler Roman
) (c:/TeXLive/2022/texmf-dist/tex/latex/microtype/mt-eur.cfg
File: mt-eur.cfg 2006/07/31 v1.1 microtype config. file: AMS Euler Roman
(RS)
)

LaTeX Font Warning: Font shape `OMS/cmsy/m/n' in size <7.5> not available
(Font)              size <7> substituted on input line 73.

LaTeX Font Info:    Trying to load font information for U+euf on input
line 73.

(c:/TeXLive/2022/texmf-dist/tex/latex/amsfonts/ueuf.fd
File: ueuf.fd 2013/01/14 v3.01 Euler Fraktur
) (c:/TeXLive/2022/texmf-dist/tex/latex/microtype/mt-euf.cfg
File: mt-euf.cfg 2006/07/03 v1.1 microtype config. file: AMS Euler
Fraktur (RS)

)
LaTeX Font Info:    Trying to load font information for U+eus on input
line 73.

(c:/TeXLive/2022/texmf-dist/tex/latex/amsfonts/ueus.fd
File: ueus.fd 2013/01/14 v3.01 Euler Script
) (c:/TeXLive/2022/texmf-dist/tex/latex/microtype/mt-eus.cfg
File: mt-eus.cfg 2006/07/28 v1.2 microtype config. file: AMS Euler Script
(RS)
)
LaTeX Font Info:    Trying to load font information for U+euex on input
line 73
.
(c:/TeXLive/2022/texmf-dist/tex/latex/amsfonts/ueuex.fd
File: ueuex.fd 2013/01/14 v3.01 Euler extra symbols
)
```

```
LaTeX Font Warning: Font shape `OML/cmm/m/it' in size <7.5> not available
(Font)              size <7> substituted on input line 73.

LaTeX Font Info:    Font shape `T1/Merriwthr-OsF/m/n' will be
(Font)              scaled to size 6.24973pt on input line 73.
LaTeX Font Info:    Font shape `T1/Merriwthr-OsF/m/n' will be
(Font)              scaled to size 5.24997pt on input line 73.
LaTeX Font Info:    Font shape `T1/Merriwthr-OsF/m/it' will be
(Font)              scaled to size 7.5pt on input line 73.
LaTeX Font Info:    Font shape `T1/Merriwthr-OsF/m/it' will be
(Font)              scaled to size 6.24973pt on input line 73.
LaTeX Font Info:    Font shape `T1/Merriwthr-OsF/m/it' will be
(Font)              scaled to size 5.24997pt on input line 73.

LaTeX Font Warning: Font shape `OT1/cmr/bx/n' in size <7.5> not available
(Font)              size <7> substituted on input line 73.

(c:/TeXLive/2022/texmf-dist/tex/latex/microtype/mt-cmr.cfg
File: mt-cmr.cfg 2013/05/19 v2.2 microtype config. file: Computer Modern
Roman
(RS)
)

LaTeX Font Warning: Font shape `OMS/cmsy/b/n' in size <7.5> not available
(Font)              size <7> substituted on input line 73.

LaTeX Font Info:    Trying to load font information for U+msa on input
line 73.

(c:/TeXLive/2022/texmf-dist/tex/latex/amsfonts/umsa.fd
File: umsa.fd 2013/01/14 v3.01 AMS symbols A
) (c:/TeXLive/2022/texmf-dist/tex/latex/microtype/mt-msa.cfg
File: mt-msa.cfg 2006/02/04 v1.1 microtype config. file: AMS symbols (a)
(RS)
)
LaTeX Font Info:    Trying to load font information for U+msb on input
line 73.

(c:/TeXLive/2022/texmf-dist/tex/latex/amsfonts/umsb.fd
File: umsb.fd 2013/01/14 v3.01 AMS symbols B
) (c:/TeXLive/2022/texmf-dist/tex/latex/microtype/mt-msb.cfg
File: mt-msb.cfg 2005/06/01 v1.0 microtype config. file: AMS symbols (b)
(RS)
)
LaTeX Font Info:    Font shape `T1/Merriwthr-OsF/m/n' will be
(Font)              scaled to size 8.0pt on input line 73.
LaTeX Font Info:    Font shape `T1/Merriwthr-OsF/m/it' will be
(Font)              scaled to size 8.0pt on input line 73.
LaTeX Font Info:    Font shape `T1/Merriwthr-OsF/b/it' will be
(Font)              scaled to size 8.0pt on input line 73.
Package caption Info: Begin \AtBeginDocument code.
Package caption Info: float package is loaded.
Package caption Info: End \AtBeginDocument code.
```

```
(c:/TeXLive/2022/texmf-dist/tex/latex/translations/translations-basic-
dictionar
y-english.trsl
File: translations-basic-dictionary-english.trsl (english translation
file `tra
nslations-basic-dictionary')
)
Package translations Info: loading dictionary `translations-basic-
dictionary' f
or `english'. on input line 73.
TextBlockOrigin set to 4pc+6.64pt x 4pc+6pt
<oup.pdf, id=161, 49.18375pt x 48.18pt>
File: oup.pdf Graphic file (type pdf)
<use oup.pdf>
Package pdftex.def Info: oup.pdf  used on input line 89.
(pdftex.def)             Requested size: 59.24683pt x 58.038pt.
<gigascience-logo.pdf, id=162, 99.37125pt x 33.12375pt>
File: gigascience-logo.pdf Graphic file (type pdf)
<use gigascience-logo.pdf>
Package pdftex.def Info: gigascience-logo.pdf  used on input line 89.
(pdftex.def)             Requested size: 126.00902pt x 42.0pt.

Overfull \hbox (54.64pt too wide) in paragraph at lines 89--89
[][]
 []

LaTeX Font Info:    Font shape `T1/Merriwthr-OsF/m/n' will be
(Font)              scaled to size 14.0pt on input line 89.
LaTeX Font Info:    Font shape `T1/Merriwthr-OsF/m/n' will be
(Font)              scaled to size 8.99997pt on input line 89.
LaTeX Font Info:    Calculating math sizes for size <14> on input line
89.
LaTeX Font Info:    Font shape `T1/Merriwthr-OsF/m/up' will be
(Font)              scaled to size 14.0pt on input line 89.
LaTeX Font Info:    Font shape `T1/Merriwthr-OsF/m/up' will be
(Font)              scaled to size 11.66617pt on input line 89.
LaTeX Font Info:    Font shape `T1/Merriwthr-OsF/m/up' will be
(Font)              scaled to size 9.79996pt on input line 89.
LaTeX Font Info:    Font shape `T1/Merriwthr-OsF/m/n' will be
(Font)              scaled to size 11.66617pt on input line 89.
LaTeX Font Info:    Font shape `T1/Merriwthr-OsF/m/n' will be
(Font)              scaled to size 9.79996pt on input line 89.
LaTeX Font Info:    Font shape `T1/Merriwthr-OsF/m/it' will be
(Font)              scaled to size 14.0pt on input line 89.
LaTeX Font Info:    Font shape `T1/Merriwthr-OsF/m/it' will be
(Font)              scaled to size 11.66617pt on input line 89.
LaTeX Font Info:    Font shape `T1/Merriwthr-OsF/m/it' will be
(Font)              scaled to size 9.79996pt on input line 89.
LaTeX Font Info:    Font shape `T1/Merriwthr-OsF/b/n' will be
(Font)              scaled to size 18.0pt on input line 89.
LaTeX Font Info:    Font shape `T1/Merriwthr-OsF/m/n' will be
(Font)              scaled to size 13.0pt on input line 89.
```

```
LaTeX Font Info:    Calculating math sizes for size <13> on input line
89.
LaTeX Font Info:    Font shape `T1/Merriwthr-OsF/m/up' will be
(Font)              scaled to size 13.0pt on input line 89.
LaTeX Font Info:    Font shape `T1/Merriwthr-OsF/m/up' will be
(Font)              scaled to size 10.83287pt on input line 89.
LaTeX Font Info:    Font shape `T1/Merriwthr-OsF/m/up' will be
(Font)              scaled to size 9.09996pt on input line 89.

LaTeX Font Warning: Font shape `OMS/cmsy/m/n' in size <13> not available
(Font)              size <12> substituted on input line 89.


LaTeX Font Warning: Font shape `OML/cmm/m/it' in size <13> not available
(Font)              size <12> substituted on input line 89.

LaTeX Font Info:    Font shape `T1/Merriwthr-OsF/m/n' will be
(Font)              scaled to size 10.83287pt on input line 89.
LaTeX Font Info:    Font shape `T1/Merriwthr-OsF/m/n' will be
(Font)              scaled to size 9.09996pt on input line 89.
LaTeX Font Info:    Font shape `T1/Merriwthr-OsF/m/it' will be
(Font)              scaled to size 13.0pt on input line 89.
LaTeX Font Info:    Font shape `T1/Merriwthr-OsF/m/it' will be
(Font)              scaled to size 10.83287pt on input line 89.
LaTeX Font Info:    Font shape `T1/Merriwthr-OsF/m/it' will be
(Font)              scaled to size 9.09996pt on input line 89.

LaTeX Font Warning: Font shape `OT1/cmr/bx/n' in size <13> not available
(Font)              size <12> substituted on input line 89.


LaTeX Font Warning: Font shape `OMS/cmsy/b/n' in size <13> not available
(Font)              size <12> substituted on input line 89.

LaTeX Font Info:    Trying to load font information for TS1+Merriwthr-OsF
on in
put line 89.
(c:/TeXLive/2022/texmf-dist/tex/latex/merriweather/TS1Merriwthr-OsF.fd
File: TS1Merriwthr-OsF.fd 2020/08/30 (autoinst) Font definitions for
TS1/Merriw
thr-OsF.
)
LaTeX Font Info:    Font shape `TS1/Merriwthr-OsF/m/n' will be
(Font)              scaled to size 10.83287pt on input line 89.
Package microtype Info: Loading generic protrusion settings for font
family
(microtype)             `Merriwthr-OsF' (encoding: TS1).
(microtype)             For optimal results, create family-specific
settings.
(microtype)             See the microtype manual for details.
LaTeX Font Info:    Font shape `T1/Merriwthr-OsF/m/n' will be
(Font)              scaled to size 9.0pt on input line 89.
LaTeX Font Info:    Font shape `T1/Merriwthr-OsF/m/up' will be
(Font)              scaled to size 9.0pt on input line 89.
```

```
LaTeX Font Info:    Font shape `T1/Merriwthr-OsF/m/up' will be
(Font)              scaled to size 7.0pt on input line 89.
LaTeX Font Info:    Font shape `T1/Merriwthr-OsF/m/up' will be
(Font)              scaled to size 5.0pt on input line 89.
LaTeX Font Info:    Font shape `T1/Merriwthr-OsF/m/n' will be
(Font)              scaled to size 7.0pt on input line 89.
LaTeX Font Info:    Font shape `T1/Merriwthr-OsF/m/n' will be
(Font)              scaled to size 5.0pt on input line 89.
LaTeX Font Info:    Font shape `T1/Merriwthr-OsF/m/it' will be
(Font)              scaled to size 9.0pt on input line 89.
LaTeX Font Info:    Font shape `T1/Merriwthr-OsF/m/it' will be
(Font)              scaled to size 7.0pt on input line 89.
LaTeX Font Info:    Font shape `T1/Merriwthr-OsF/m/it' will be
(Font)              scaled to size 5.0pt on input line 89.
LaTeX Font Info:    Font shape `T1/Merriwthr-OsF/m/n' will be
(Font)              scaled to size 6.5pt on input line 89.
LaTeX Font Info:    Calculating math sizes for size <6.5> on input line
89.
LaTeX Font Info:    Font shape `T1/Merriwthr-OsF/m/up' will be
(Font)              scaled to size 6.5pt on input line 89.
LaTeX Font Info:    Font shape `T1/Merriwthr-OsF/m/up' will be
(Font)              scaled to size 5.41643pt on input line 89.
LaTeX Font Info:    Font shape `T1/Merriwthr-OsF/m/up' will be
(Font)              scaled to size 4.54997pt on input line 89.

LaTeX Font Warning: Font shape `OMS/cmsy/m/n' in size <6.5> not available
(Font)              size <6> substituted on input line 89.


LaTeX Font Warning: Font shape `OMS/cmsy/m/n' in size <5.41643> not
available
(Font)              size <5> substituted on input line 89.


LaTeX Font Warning: Font shape `OMS/cmsy/m/n' in size <4.54997> not
available
(Font)              size <5> substituted on input line 89.


LaTeX Font Warning: Font shape `OML/cmm/m/it' in size <6.5> not available
(Font)              size <6> substituted on input line 89.


LaTeX Font Warning: Font shape `OML/cmm/m/it' in size <5.41643> not
available
(Font)              size <5> substituted on input line 89.


LaTeX Font Warning: Font shape `OML/cmm/m/it' in size <4.54997> not
available
(Font)              size <5> substituted on input line 89.

LaTeX Font Info:    Font shape `T1/Merriwthr-OsF/m/n' will be
(Font)              scaled to size 5.41643pt on input line 89.
```

```
LaTeX Font Info:    Font shape `T1/Merriwthr-OsF/m/n' will be
(Font)              scaled to size 4.54997pt on input line 89.
LaTeX Font Info:    Font shape `T1/Merriwthr-OsF/m/it' will be
(Font)              scaled to size 6.5pt on input line 89.
LaTeX Font Info:    Font shape `T1/Merriwthr-OsF/m/it' will be
(Font)              scaled to size 5.41643pt on input line 89.
LaTeX Font Info:    Font shape `T1/Merriwthr-OsF/m/it' will be
(Font)              scaled to size 4.54997pt on input line 89.

LaTeX Font Warning: Font shape `OT1/cmr/bx/n' in size <6.5> not available
(Font)              size <6> substituted on input line 89.


LaTeX Font Warning: Font shape `OT1/cmr/bx/n' in size <5.41643> not
available
(Font)              size <5> substituted on input line 89.


LaTeX Font Warning: Font shape `OT1/cmr/bx/n' in size <4.54997> not
available
(Font)              size <5> substituted on input line 89.


LaTeX Font Warning: Font shape `OMS/cmsy/b/n' in size <6.5> not available
(Font)              size <6> substituted on input line 89.


LaTeX Font Warning: Font shape `OMS/cmsy/b/n' in size <5.41643> not
available
(Font)              size <5> substituted on input line 89.


LaTeX Font Warning: Font shape `OMS/cmsy/b/n' in size <4.54997> not
available
(Font)              size <5> substituted on input line 89.

LaTeX Font Info:    Font shape `TS1/Merriwthr-OsF/m/n' will be
(Font)              scaled to size 5.41643pt on input line 89.

Overfull \hbox (54.64pt too wide) in paragraph at lines 89--89
[][][]
 []

LaTeX Font Info:    Font shape `T1/Merriwthr-OsF/b/n' will be
(Font)              scaled to size 10.0pt on input line 89.
LaTeX Font Info:    Font shape `T1/Merriwthr-OsF/b/n' will be
(Font)              scaled to size 8.0pt on input line 89.

Overfull \hbox (54.64pt too wide) in paragraph at lines 89--89
[][][]
 []

Package mdframed Info: mdframed works in twoside mode on input line 92.
LaTeX Font Info:    Font shape `T1/Merriwthr-OsF/b/n' will be
```

```
(Font)               scaled to size 8.2pt on input line 92.
LaTeX Font Info:    Font shape `TS1/Merriwthr-OsF/m/n' will be
(Font)               scaled to size 7.5pt on input line 94.
Package mdframed Info: mdframed inside float
 mdframed uses option nobreak mdframed on input line 101.
Package mdframed Info: mdframed inside a box
 mdframed uses option nobreak mdframed on input line 101.
LaTeX Font Info:    Font shape `T1/Merriwthr-OsF/b/n' will be
(Font)               scaled to size 7.5pt on input line 105.

Package natbib Warning: Citation `recanatini2020drug' on page 1 undefined on in
put line 105.


Package natbib Warning: Citation `azuaje2013drug' on page 1 undefined on input
line 105.


Package natbib Warning: Citation `zhang2017predicting' on page 1 undefined on i
nput line 105.


Package natbib Warning: Citation `zhu2021multi' on page 1 undefined on input li
ne 105.


Package natbib Warning: Citation `qiu2021comprehensive' on page 1 undefined on
input line 105.


Package natbib Warning: Citation `su2022biomedical' on page 1 undefined on inpu
t line 105.


Package natbib Warning: Citation `lu2017link' on page 1 undefined on input line
 105.


Package natbib Warning: Citation `luo2017network' on page 1 undefined on input
line 105.


Package natbib Warning: Citation `wu2018network' on page 1 undefined on input l
ine 105.
```

```
Package natbib Warning: Citation `thafar2020dtigems+' on page 1 undefined
on in
put line 105.


Package natbib Warning: Citation `thafar2021dti2vec' on page 1 undefined
on inp
ut line 105.


Package natbib Warning: Citation `lotfi2018review' on page 1 undefined on
input
 line 105.


Package natbib Warning: Citation `sadeghi2019analytical' on page 1
undefined on
 input line 105.


Package natbib Warning: Citation `badkas2020topological' on page 1
undefined on
 input line 105.


Underfull \vbox (badness 10000) has occurred while \output is active []


Underfull \vbox (badness 10000) has occurred while \output is active []

LaTeX Font Info:    Font shape `T1/Merriwthr-OsF/m/n' will be
(Font)              scaled to size 7.8pt on input line 106.
LaTeX Font Info:    Font shape `T1/Merriwthr-OsF/b/n' will be
(Font)              scaled to size 7.8pt on input line 106.
[1{c:/TeXLive/2022/texmf-var/fonts/map/pdftex/updmap/pdftex.map}


 <./oup.pdf> <./gigascience-logo.pdf>]

Package natbib Warning: Citation `qiu2021comprehensive' on page 2
undefined on
input line 107.


Package natbib Warning: Citation `su2022biomedical' on page 2 undefined
on inpu
t line 107.


Package natbib Warning: Citation `luo2017network' on page 2 undefined on
input
line 107.
```

Package natbib Warning: Citation `thafar2021dti2vec' on page 2 undefined on inp
ut line 107.

Package natbib Warning: Citation `jourdan2020drug' on page 2 undefined on input
 line 109.

Package natbib Warning: Citation `bolgar2013drug' on page 2 undefined on input
line 109.

Package natbib Warning: Citation `sridhar2016probabilistic' on page 2 undefined
 on input line 109.

Package natbib Warning: Citation `lin2020kgnn' on page 2 undefined on input lin
e 109.

Package natbib Warning: Citation `feng2020dpddi' on page 2 undefined on input l
ine 109.

Package natbib Warning: Citation `dickson2009cost' on page 2 undefined on input
 line 111.

Package natbib Warning: Citation `chen2006discovery' on page 2 undefined on inp
ut line 111.

Package natbib Warning: Citation `fda2021url' on page 2 undefined on input line
 111.

Package natbib Warning: Citation `sardana2011drug' on page 2 undefined on input
 line 111.

Package natbib Warning: Citation `serafin2020drug' on page 2 undefined on input
 line 111.

Package natbib Warning: Citation `gysi2021network' on page 2 undefined on input
 line 111.

Package natbib Warning: Citation `altman1995statistics' on page 2 undefined on
input line 113.

Underfull \hbox (badness 1478) in paragraph at lines 113--114
\T1/Merriwthr-OsF/m/n/7.5 (+20) lems. The most crit-i-cal is-sue that af-fects
the ro-bust-ness of
 []

Package natbib Warning: Citation `mestres2008data' on page 2 undefined on input
 line 115.

Package natbib Warning: Citation `wishart2018drugbank' on page 2 undefined on i
nput line 119.

Package natbib Warning: Citation `avizienis2004basic' on page 2 undefined on in
put line 119.

Package natbib Warning: Citation `mestres2008data' on page 2 undefined on input
 line 119.

Package natbib Warning: Citation `bleakley2009supervised' on page 2 undefined o
n input line 121.

Package natbib Warning: Citation `cheng2012prediction' on page 2 undefined on i
nput line 121.

Package natbib Warning: Citation `lu2015toward' on page 2 undefined on input li
ne 121.

```
Package natbib Warning: Citation `xue2018review' on page 2 undefined on
input l
ine 121.


Package natbib Warning: Citation `wu2013network' on page 2 undefined on
input l
ine 121.


Package natbib Warning: Citation `udrescu2016clustering' on page 2
undefined on
 input line 121.


Package natbib Warning: Citation `yamanishi2008prediction' on page 2
undefined
on input line 121.


Package natbib Warning: Citation `mestres2009topology' on page 2
undefined on i
nput line 121.


Package natbib Warning: Citation `tabei2012identification' on page 2
undefined
on input line 121.


Package natbib Warning: Citation `tanoli2020interactive' on page 2
undefined on
 input line 121.


Package natbib Warning: Citation `udrescu2020uncovering' on page 2
undefined on
 input line 121.


Package natbib Warning: Citation `udrescu2020uncovering' on page 2
undefined on
 input line 121.


Underfull \vbox (badness 1077) has occurred while \output is active []

LaTeX Font Info:    Font shape `T1/Merriwthr-OsF/m/it' will be
(Font)              scaled to size 7.8pt on input line 122.
[2]

Package natbib Warning: Citation `newman2006structure' on page 3
undefined on i
nput line 128.
```

```
Package natbib Warning: Citation `mestres2008data' on page 3 undefined on
input
 line 136.


Package natbib Warning: Citation `avram2022novel' on page 3 undefined on
input
line 136.


LaTeX Warning: File `Fig/DDI-evo-deg-1.pdf' not found on input line 140.


! Package pdftex.def Error: File `Fig/DDI-evo-deg-1.pdf' not found: using
draft
 setting.

See the pdftex.def package documentation for explanation.
Type  H <return>  for immediate help.
 ...

l.140 ...[width=\linewidth]{Fig/DDI-evo-deg-1.pdf}
                                                  }
Try typing  <return>  to proceed.
If that doesn't work, type  X <return>  to quit.

LaTeX Font Info:    Trying to load font information for T1+lmtt on input
line 1
40.
(c:/TeXLive/2022/texmf-dist/tex/latex/lm/t1lmtt.fd
File: t1lmtt.fd 2015/05/01 v1.6.1 Font defs for Latin Modern
)
Package microtype Info: Loading generic protrusion settings for font
family
(microtype)             `lmtt' (encoding: T1).
(microtype)             For optimal results, create family-specific
settings.
(microtype)             See the microtype manual for details.
LaTeX Font Info:    Font shape `T1/Merriwthr-OsF/m/n' will be
(Font)              scaled to size 6.0pt on input line 141.
LaTeX Font Info:    Font shape `T1/Merriwthr-OsF/b/n' will be
(Font)              scaled to size 6.0pt on input line 141.

LaTeX Warning: File `Fig/DTI-evo-new.pdf' not found on input line 145.


! Package pdftex.def Error: File `Fig/DTI-evo-new.pdf' not found: using
draft s
etting.

See the pdftex.def package documentation for explanation.
Type  H <return>  for immediate help.
```

```
 ...

l.145 ...cs[width=\linewidth]{Fig/DTI-evo-new.pdf}
                                                  }
Try typing  <return>  to proceed.
If that doesn't work, type  X <return>  to quit.


Package natbib Warning: Citation `wishart2018drugbank' on page 3
undefined on i
nput line 151.


Package natbib Warning: Citation `wishart2008drugbank' on page 3
undefined on i
nput line 151.


Package natbib Warning: Citation `wishart2006drugbank' on page 3
undefined on i
nput line 151.


Package natbib Warning: Citation `wishart2018drugbank' on page 3
undefined on i
nput line 151.

LaTeX Font Info:    Font shape `T1/Merriwthr-OsF/m/up' will be
(Font)              scaled to size 7.5pt on input line 153.
LaTeX Font Info:    Font shape `T1/Merriwthr-OsF/b/n' will be
(Font)              scaled to size 8.5pt on input line 160.

Package natbib Warning: Citation `newman2002structure' on page 3
undefined on i
nput line 161.


Package natbib Warning: Citation `wang2003complex' on page 3 undefined on
input
 line 161.


Package natbib Warning: Citation `newman2006structure' on page 3
undefined on i
nput line 161.


Package natbib Warning: Citation `barabasi2013network' on page 3
undefined on i
nput line 161.

[3]
Underfull \vbox (badness 10000) has occurred while \output is active []
```

```
Underfull \vbox (badness 10000) has occurred while \output is active []

[4]
Underfull \hbox (badness 1701) in paragraph at lines 173--174
[][]\T1/Merriwthr-OsF/m/up/7.5 (+20) Using the com-plex net-work for-mal-
ism, i
n DDI net-works,
 []


Underfull \vbox (badness 2096) has occurred while \output is active []


Package natbib Warning: Citation `mestres2008data' on page 5 undefined on
input
 line 182.

! Undefined control sequence.
\enit@endenumerate ->\enit@after
                                 \endlist \ifx \enit@series \relax \else
\if...
l.183 \end{enumerate}

The control sequence at the end of the top line
of your error message was never \def'ed. If you have
misspelled it (e.g., `\hobx'), type `I' and the correct
spelling (e.g., `I\hbox'). Otherwise just continue,
and I'll forget about whatever was undefined.

[5]
! Undefined control sequence.
\enit@endenumerate ...\else \ifnum \enit@resuming
                                                  =\@ne
\enit@setresumekeys ...
l.183 \end{enumerate}

The control sequence at the end of the top line
of your error message was never \def'ed. If you have
misspelled it (e.g., `\hobx'), type `I' and the correct
spelling (e.g., `I\hbox'). Otherwise just continue,
and I'll forget about whatever was undefined.

! Missing number, treated as zero.
<to be read again>
                   =
l.183 \end{enumerate}

A number should have been here; I inserted `0'.
(If you can't figure out why I needed to see a number,
look up `weird error' in the index to The TeXbook.)

! Undefined control sequence.
\enit@setresumekeys ...xpandafter {\enit@savekeys
```

```
                                                  }\xdef \enit@afterlist
{#2...
l.183 \end{enumerate}

The control sequence at the end of the top line
of your error message was never \def'ed. If you have
misspelled it (e.g., `\hobx'), type `I' and the correct
spelling (e.g., `I\hbox'). Otherwise just continue,
and I'll forget about whatever was undefined.

! Undefined control sequence.
\enit@setresumekeys ...it@toks }\ifnum \enit@type
                                                  =\z@ #3\def
\enit@noexcs {...
l.183 \end{enumerate}

The control sequence at the end of the top line
of your error message was never \def'ed. If you have
misspelled it (e.g., `\hobx'), type `I' and the correct
spelling (e.g., `I\hbox'). Otherwise just continue,
and I'll forget about whatever was undefined.

! Missing number, treated as zero.
<to be read again>
                   =
l.183 \end{enumerate}

A number should have been here; I inserted `0'.
(If you can't figure out why I needed to see a number,
look up `weird error' in the index to The TeXbook.)

! Undefined control sequence.
<argument> enit@resume@series@\enit@series

l.183 \end{enumerate}

The control sequence at the end of the top line
of your error message was never \def'ed. If you have
misspelled it (e.g., `\hobx'), type `I' and the correct
spelling (e.g., `I\hbox'). Otherwise just continue,
and I'll forget about whatever was undefined.

! Undefined control sequence.
\enit@endenumerate ...t \fi \ifnum \enit@resuming
                                                  =\thr@@
\enit@setresumekey...
l.183 \end{enumerate}

The control sequence at the end of the top line
of your error message was never \def'ed. If you have
misspelled it (e.g., `\hobx'), type `I' and the correct
spelling (e.g., `I\hbox'). Otherwise just continue,
and I'll forget about whatever was undefined.
```

```
! Missing number, treated as zero.
<to be read again>
                   =
l.183 \end{enumerate}

A number should have been here; I inserted `0'.
(If you can't figure out why I needed to see a number,
look up `weird error' in the index to The TeXbook.)

! Undefined control sequence.
\enit@setresumekeys ...xpandafter {\enit@savekeys
                                                  }\xdef \enit@afterlist
{#2...
l.183 \end{enumerate}

The control sequence at the end of the top line
of your error message was never \def'ed. If you have
misspelled it (e.g., `\hobx'), type `I' and the correct
spelling (e.g., `I\hbox'). Otherwise just continue,
and I'll forget about whatever was undefined.

! Undefined control sequence.
\enit@setresumekeys ...it@toks }\ifnum \enit@type
                                                  =\z@ #3\def
\enit@noexcs {...
l.183 \end{enumerate}

The control sequence at the end of the top line
of your error message was never \def'ed. If you have
misspelled it (e.g., `\hobx'), type `I' and the correct
spelling (e.g., `I\hbox'). Otherwise just continue,
and I'll forget about whatever was undefined.

! Missing number, treated as zero.
<to be read again>
                   =
l.183 \end{enumerate}

A number should have been here; I inserted `0'.
(If you can't figure out why I needed to see a number,
look up `weird error' in the index to The TeXbook.)


Package natbib Warning: Citation `wang2003complex' on page 6 undefined on
input
 line 186.

! Undefined control sequence.
\enit@endenumerate ->\enit@after
                                \endlist \ifx \enit@series \relax \else
\if...
l.188 \end{enumerate}

The control sequence at the end of the top line
```

```
of your error message was never \def'ed. If you have
misspelled it (e.g., `\hobx'), type `I' and the correct
spelling (e.g., `I\hbox'). Otherwise just continue,
and I'll forget about whatever was undefined.

! Undefined control sequence.
\enit@endenumerate ...\else \ifnum \enit@resuming
                                                  =\@ne
\enit@setresumekeys ...
l.188 \end{enumerate}

The control sequence at the end of the top line
of your error message was never \def'ed. If you have
misspelled it (e.g., `\hobx'), type `I' and the correct
spelling (e.g., `I\hbox'). Otherwise just continue,
and I'll forget about whatever was undefined.

! Missing number, treated as zero.
<to be read again>
                   =
l.188 \end{enumerate}

A number should have been here; I inserted `0'.
(If you can't figure out why I needed to see a number,
look up `weird error' in the index to The TeXbook.)

! Undefined control sequence.
\enit@setresumekeys ...xpandafter {\enit@savekeys
                                                  }\xdef \enit@afterlist
{#2...
l.188 \end{enumerate}

The control sequence at the end of the top line
of your error message was never \def'ed. If you have
misspelled it (e.g., `\hobx'), type `I' and the correct
spelling (e.g., `I\hbox'). Otherwise just continue,
and I'll forget about whatever was undefined.

! Undefined control sequence.
\enit@setresumekeys ...it@toks }\ifnum \enit@type
                                                  =\z@ #3\def
\enit@noexcs {...
l.188 \end{enumerate}

The control sequence at the end of the top line
of your error message was never \def'ed. If you have
misspelled it (e.g., `\hobx'), type `I' and the correct
spelling (e.g., `I\hbox'). Otherwise just continue,
and I'll forget about whatever was undefined.

! Missing number, treated as zero.
<to be read again>
                   =
l.188 \end{enumerate}
```

```
A number should have been here; I inserted `0'.
(If you can't figure out why I needed to see a number,
look up `weird error' in the index to The TeXbook.)

! Undefined control sequence.
<argument> enit@resume@series@\enit@series

l.188 \end{enumerate}

The control sequence at the end of the top line
of your error message was never \def'ed. If you have
misspelled it (e.g., `\hobx'), type `I' and the correct
spelling (e.g., `I\hbox'). Otherwise just continue,
and I'll forget about whatever was undefined.

! Undefined control sequence.
\enit@endenumerate ...t \fi \ifnum \enit@resuming
                                                  =\thr@@
\enit@setresumekey...
l.188 \end{enumerate}

The control sequence at the end of the top line
of your error message was never \def'ed. If you have
misspelled it (e.g., `\hobx'), type `I' and the correct
spelling (e.g., `I\hbox'). Otherwise just continue,
and I'll forget about whatever was undefined.

! Missing number, treated as zero.
<to be read again>
                   =
l.188 \end{enumerate}

A number should have been here; I inserted `0'.
(If you can't figure out why I needed to see a number,
look up `weird error' in the index to The TeXbook.)

! Undefined control sequence.
\enit@setresumekeys ...xpandafter {\enit@savekeys
                                                  }\xdef \enit@afterlist
{#2...
l.188 \end{enumerate}

The control sequence at the end of the top line
of your error message was never \def'ed. If you have
misspelled it (e.g., `\hobx'), type `I' and the correct
spelling (e.g., `I\hbox'). Otherwise just continue,
and I'll forget about whatever was undefined.

! Undefined control sequence.
\enit@setresumekeys ...it@toks }\ifnum \enit@type
                                                  =\z@ #3\def
\enit@noexcs {...
l.188 \end{enumerate}
```

```
The control sequence at the end of the top line
of your error message was never \def'ed. If you have
misspelled it (e.g., `\hobx'), type `I' and the correct
spelling (e.g., `I\hbox'). Otherwise just continue,
and I'll forget about whatever was undefined.

! Missing number, treated as zero.
<to be read again>
                   =
l.188 \end{enumerate}

A number should have been here; I inserted `0'.
(If you can't figure out why I needed to see a number,
look up `weird error' in the index to The TeXbook.)

LaTeX Font Info:    Font shape `T1/Merriwthr-OsF/b/sl' in size <7.5> not
availa
ble
(Font)              Font shape `T1/Merriwthr-OsF/b/it' tried instead on
input l
ine 199.
LaTeX Font Info:    Font shape `T1/Merriwthr-OsF/b/it' will be
(Font)              scaled to size 7.5pt on input line 199.

Underfull \hbox (badness 2781) in paragraph at lines 218--219
[]\T1/Merriwthr-OsF/m/up/7.5 (+20) The \T1/Merriwthr-OsF/m/it/7.5 (+20)
net-wor
k den-sity \T1/Merriwthr-OsF/m/up/7.5 (+20) is the ra-tio be-tween the
num-ber
of
 []


Package natbib Warning: Citation `jeong2001lethality' on page 6 undefined
on in
put line 238.


Package natbib Warning: Citation `koschutzki2004comparison' on page 6
undefined
 on input line 238.


Package natbib Warning: Citation `salavati2019ranking' on page 6
undefined on i
nput line 238.


Package natbib Warning: Citation `yildirim2007drug' on page 6 undefined
on inpu
t line 240.
```

Package natbib Warning: Citation `maccuish2010clustering' on page 6 undefined o
n input line 240.

Package natbib Warning: Citation `udrescu2016clustering' on page 6 undefined on
 input line 240.

Package natbib Warning: Citation `gysi2021network' on page 6 undefined on input
 line 240.

Underfull \vbox (badness 3849) has occurred while \output is active []

[6]

Package natbib Warning: Citation `estrada2012structure' on page 7 undefined on
input line 264.

Package natbib Warning: Citation `wang2003complex' on page 7 undefined on input
 line 268.

Package natbib Warning: Citation `topirceanu2014genetically' on page 7 undefine
d on input line 268.

Package natbib Warning: Citation `mestres2008data' on page 7 undefined on input
 line 272.

Package natbib Warning: Citation `orita2013agreement' on page 7 undefined on in
put line 272.

Package natbib Warning: Citation `orita2013agreement' on page 7 undefined on in
put line 272.

Package natbib Warning: Citation `morzy2016benford' on page 7 undefined on inpu
t line 279.

```
Package natbib Warning: Citation `grandison2008biological' on page 7
undefined
on input line 279.


Package natbib Warning: Citation `karthik2016elucidating' on page 7
undefined o
n input line 279.


Package natbib Warning: Citation `mestres2008data' on page 7 undefined on
input
 line 283.


Underfull \vbox (badness 2753) has occurred while \output is active []

Package hyperref Info: bookmark level for unknown algorithm defaults to 0
on in
put line 288.
! Missing \endcsname inserted.
<to be read again>
                   \ALG@currentblock@0
l.291 \State
             $E_t \Leftarrow \left\{ e_{ij} = (v_i, v_j)\vert v_i, v_j\in
V ...
The control sequence marked <to be read again> should
not appear between \csname and \endcsname.

! Extra \endcsname.
\ALG@makebeginrepeat ...\ALG@thisblock \endcsname
                                                  \relax \def
\ALG@thisblock...
l.291 \State
             $E_t \Leftarrow \left\{ e_{ij} = (v_i, v_j)\vert v_i, v_j\in
V ...
I'm ignoring this, since I wasn't doing a \csname.


! LaTeX Error: Lonely \item--perhaps a missing list environment.

See the LaTeX manual or LaTeX Companion for explanation.
Type  H <return>  for immediate help.
 ...

l.291 \State
             $E_t \Leftarrow \left\{ e_{ij} = (v_i, v_j)\vert v_i, v_j\in
V ...

Try typing  <return>  to proceed.
If that doesn't work, type  X <return>  to quit.


Underfull \hbox (badness 10000) detected at line 291
```

```
[][]
 []

! Missing \endcsname inserted.
<to be read again>
                   \ALG@currentblock@0
l.293 \For
           {$r$ in 0 to $R$}
The control sequence marked <to be read again> should
not appear between \csname and \endcsname.

! Extra \endcsname.
\ALG@makebeginrepeat ...\ALG@thisblock \endcsname
                                                  \relax \def
\ALG@thisblock...
l.293 \For
           {$r$ in 0 to $R$}
I'm ignoring this, since I wasn't doing a \csname.

LaTeX Font Info:    Font shape `T1/Merriwthr-OsF/m/n' will be
(Font)              scaled to size 6.25008pt on input line 293.
! Missing number, treated as zero.
<to be read again>
                   \ALG@currentblock@0
l.307 \end{algorithmic}

A number should have been here; I inserted `0'.
(If you can't figure out why I needed to see a number,
look up `weird error' in the index to The TeXbook.)

! Missing = inserted for \ifnum.
<to be read again>
                   \ALG@currentblock@0
l.307 \end{algorithmic}

I was expecting to see `<', `=', or `>'. Didn't.

! Missing number, treated as zero.
<to be read again>
                   \ALG@currentblock@0
l.307 \end{algorithmic}

A number should have been here; I inserted `0'.
(If you can't figure out why I needed to see a number,
look up `weird error' in the index to The TeXbook.)

[7]

Package natbib Warning: Citation `lotfi2018review' on page 8 undefined on
input
 line 324.
```

```
Package natbib Warning: Citation `sadeghi2019analytical' on page 8
undefined on
 input line 324.


Package natbib Warning: Citation `recanatini2020drug' on page 8 undefined
on in
put line 324.


Package natbib Warning: Citation `xue2018review' on page 8 undefined on
input l
ine 324.


Package natbib Warning: Citation `kastrin2018predicting' on page 8
undefined on
 input line 324.


Package natbib Warning: Citation `goh2007human' on page 8 undefined on
input li
ne 324.


LaTeX Warning: File `Fig/no-ddi.pdf' not found on input line 333.


! Package pdftex.def Error: File `Fig/no-ddi.pdf' not found: using draft
settin
g.

See the pdftex.def package documentation for explanation.
Type  H <return>  for immediate help.
 ...

l.333 ...ics[width=0.46\linewidth]{Fig/no-ddi.pdf}

}\phantom{spa}{\includegra...

Try typing  <return>  to proceed.
If that doesn't work, type  X <return>  to quit.


LaTeX Warning: File `Fig/no-dti.pdf' not found on input line 333.


! Package pdftex.def Error: File `Fig/no-dti.pdf' not found: using draft
settin
g.

See the pdftex.def package documentation for explanation.
Type  H <return>  for immediate help.
 ...
```

```
l.333 ...ics[width=0.52\linewidth]{Fig/no-dti.pdf}
                                                  }\\ \vspace{0.2cm}
Try typing  <return>  to proceed.
If that doesn't work, type  X <return>  to quit.


Overfull \hbox (2.45076pt too wide) in paragraph at lines 333--333
 [][][][]
 []

LaTeX Font Info:    Font shape `T1/Merriwthr-OsF/m/up' will be
(Font)              scaled to size 6.0pt on input line 335.
LaTeX Font Info:    Font shape `T1/Merriwthr-OsF/m/it' will be
(Font)              scaled to size 6.0pt on input line 335.

LaTeX Warning: File `Fig/links-ddi.pdf' not found on input line 343.


! Package pdftex.def Error: File `Fig/links-ddi.pdf' not found: using
draft set
ting.

See the pdftex.def package documentation for explanation.
Type  H <return>  for immediate help.
 ...

l.343 ...[width=0.49\linewidth]{Fig/links-ddi.pdf}
                                                  }
{\includegraphics[width=...

Try typing  <return>  to proceed.
If that doesn't work, type  X <return>  to quit.


LaTeX Warning: File `Fig/links-dti.pdf' not found on input line 343.


! Package pdftex.def Error: File `Fig/links-dti.pdf' not found: using
draft set
ting.

See the pdftex.def package documentation for explanation.
Type  H <return>  for immediate help.
 ...

l.343 ...[width=0.49\linewidth]{Fig/links-dti.pdf}
                                                  } \\
Try typing  <return>  to proceed.
If that doesn't work, type  X <return>  to quit.


LaTeX Warning: File `Fig/dens-ddi.pdf' not found on input line 345.
```

```
! Package pdftex.def Error: File `Fig/dens-ddi.pdf' not found: using
draft sett
ing.

See the pdftex.def package documentation for explanation.
Type  H <return>  for immediate help.
 ...

l.345 ...s[width=0.49\linewidth]{Fig/dens-ddi.pdf}
                                                  }
{\includegraphics[width...

Try typing  <return>  to proceed.
If that doesn't work, type  X <return>  to quit.


LaTeX Warning: File `Fig/dens-dti.pdf' not found on input line 345.


! Package pdftex.def Error: File `Fig/dens-dti.pdf' not found: using
draft sett
ing.

See the pdftex.def package documentation for explanation.
Type  H <return>  for immediate help.
 ...

l.345 ...s[width=0.49\linewidth]{Fig/dens-dti.pdf}
                                                  } \\
Try typing  <return>  to proceed.
If that doesn't work, type  X <return>  to quit.


LaTeX Warning: File `Fig/apl.pdf' not found on input line 354.


! Package pdftex.def Error: File `Fig/apl.pdf' not found: using draft
setting.

See the pdftex.def package documentation for explanation.
Type  H <return>  for immediate help.
 ...

l.354 ...aphics[width=0.49\linewidth]{Fig/apl.pdf}
                                                  }
{\includegraphics[width=...

Try typing  <return>  to proceed.
If that doesn't work, type  X <return>  to quit.


LaTeX Warning: File `Fig/dia.pdf' not found on input line 354.
```

```
! Package pdftex.def Error: File `Fig/dia.pdf' not found: using draft
setting.

See the pdftex.def package documentation for explanation.
Type  H <return>  for immediate help.
 ...

l.354 ...aphics[width=0.49\linewidth]{Fig/dia.pdf}
                                                  } \\
Try typing  <return>  to proceed.
If that doesn't work, type  X <return>  to quit.


LaTeX Warning: File `Fig/ad-ddi.pdf' not found on input line 361.


! Package pdftex.def Error: File `Fig/ad-ddi.pdf' not found: using draft
settin
g.

See the pdftex.def package documentation for explanation.
Type  H <return>  for immediate help.
 ...

l.361 ...ics[width=0.49\linewidth]{Fig/ad-ddi.pdf}
                                                  }
{\includegraphics[width=...

Try typing  <return>  to proceed.
If that doesn't work, type  X <return>  to quit.


LaTeX Warning: File `Fig/ad-dti.pdf' not found on input line 361.


! Package pdftex.def Error: File `Fig/ad-dti.pdf' not found: using draft
settin
g.

See the pdftex.def package documentation for explanation.
Type  H <return>  for immediate help.
 ...

l.361 ...ics[width=0.49\linewidth]{Fig/ad-dti.pdf}
                                                  } \\
Try typing  <return>  to proceed.
If that doesn't work, type  X <return>  to quit.


LaTeX Warning: File `Fig/cc-ddi.pdf' not found on input line 370.
```

```
! Package pdftex.def Error: File `Fig/cc-ddi.pdf' not found: using draft
settin
g.

See the pdftex.def package documentation for explanation.
Type  H <return>  for immediate help.
 ...

l.370 ...raphics[width=\linewidth]{Fig/cc-ddi.pdf}
                                                  }
Try typing  <return>  to proceed.
If that doesn't work, type  X <return>  to quit.


Package natbib Warning: Citation `wang2003complex' on page 8 undefined on
input
 line 374.


Package natbib Warning: Citation `barabasi2009scale' on page 8 undefined
on inp
ut line 374.


LaTeX Warning: File `Fig/degree-alpha.pdf' not found on input line 378.


! Package pdftex.def Error: File `Fig/degree-alpha.pdf' not found: using
draft
setting.

See the pdftex.def package documentation for explanation.
Type  H <return>  for immediate help.
 ...

l.378 ...s[width=\linewidth]{Fig/degree-alpha.pdf}
                                                  }
Try typing  <return>  to proceed.
If that doesn't work, type  X <return>  to quit.


Package natbib Warning: Citation `lotfi2018review' on page 8 undefined on
input
 line 382.


Package natbib Warning: Citation `badkas2020topological' on page 8
undefined on
 input line 382.


Package natbib Warning: Citation `gysi2021network' on page 8 undefined on
input
 line 382.
```

```
Package natbib Warning: Citation `wang2003complex' on page 8 undefined on
input
 line 382.


Package natbib Warning: Citation `topirceanu2014genetically' on page 8
undefine
d on input line 382.


Package natbib Warning: Citation `wang2003complex' on page 8 undefined on
input
 line 384.


LaTeX Warning: File `Fig/degDistrib30.pdf' not found on input line 390.


! Package pdftex.def Error: File `Fig/degDistrib30.pdf' not found: using
draft
setting.

See the pdftex.def package documentation for explanation.
Type  H <return>  for immediate help.
 ...

l.390 ...dth=0.35\linewidth]{Fig/degDistrib30.pdf}
                                                  }
Try typing  <return>  to proceed.
If that doesn't work, type  X <return>  to quit.


LaTeX Warning: File `Fig/degDistrib519.pdf' not found on input line 391.


! Package pdftex.def Error: File `Fig/degDistrib519.pdf' not found: using
draft
 setting.

See the pdftex.def package documentation for explanation.
Type  H <return>  for immediate help.
 ...

l.391 ...th=0.35\linewidth]{Fig/degDistrib519.pdf}
                                                  } \\ a) \hspace{5.5cm}
b) ...

Try typing  <return>  to proceed.
If that doesn't work, type  X <return>  to quit.


LaTeX Warning: File `Fig/betDistrib30.pdf' not found on input line 392.
```

```
! Package pdftex.def Error: File `Fig/betDistrib30.pdf' not found: using
draft
setting.

See the pdftex.def package documentation for explanation.
Type  H <return>  for immediate help.
 ...

l.392 ...dth=0.35\linewidth]{Fig/betDistrib30.pdf}
                                                  }
Try typing  <return>  to proceed.
If that doesn't work, type  X <return>  to quit.


LaTeX Warning: File `Fig/betDistrib519.pdf' not found on input line 393.


! Package pdftex.def Error: File `Fig/betDistrib519.pdf' not found: using
draft
 setting.

See the pdftex.def package documentation for explanation.
Type  H <return>  for immediate help.
 ...

l.393 ...th=0.35\linewidth]{Fig/betDistrib519.pdf}
                                                  } \\ c) \hspace{5.5cm}
d)
Try typing  <return>  to proceed.
If that doesn't work, type  X <return>  to quit.


Package natbib Warning: Citation `alstott2014powerlaw' on page 8
undefined on i
nput line 394.


LaTeX Warning: File `Fig/eigDistrib30.pdf' not found on input line 398.


! Package pdftex.def Error: File `Fig/eigDistrib30.pdf' not found: using
draft
setting.

See the pdftex.def package documentation for explanation.
Type  H <return>  for immediate help.
 ...

l.398 ...dth=0.35\linewidth]{Fig/eigDistrib30.pdf}
                                                  }
Try typing  <return>  to proceed.
If that doesn't work, type  X <return>  to quit.
```

```
LaTeX Warning: File `Fig/eigDistrib519.pdf' not found on input line 399.


! Package pdftex.def Error: File `Fig/eigDistrib519.pdf' not found: using
draft
 setting.

See the pdftex.def package documentation for explanation.
Type  H <return>  for immediate help.
 ...

l.399 ...th=0.35\linewidth]{Fig/eigDistrib519.pdf}
                                                  } \\ a) \hspace{5.5cm}
b) ...

Try typing  <return>  to proceed.
If that doesn't work, type  X <return>  to quit.


LaTeX Warning: File `Fig/pagDistrib30.pdf' not found on input line 400.


! Package pdftex.def Error: File `Fig/pagDistrib30.pdf' not found: using
draft
setting.

See the pdftex.def package documentation for explanation.
Type  H <return>  for immediate help.
 ...

l.400 ...dth=0.35\linewidth]{Fig/pagDistrib30.pdf}
                                                  }
Try typing  <return>  to proceed.
If that doesn't work, type  X <return>  to quit.


LaTeX Warning: File `Fig/pagDistrib519.pdf' not found on input line 401.


! Package pdftex.def Error: File `Fig/pagDistrib519.pdf' not found: using
draft
 setting.

See the pdftex.def package documentation for explanation.
Type  H <return>  for immediate help.
 ...

l.401 ...th=0.35\linewidth]{Fig/pagDistrib519.pdf}
                                                  } \\ c) \hspace{5.5cm}
d) ...

Try typing  <return>  to proceed.
```

```
If that doesn't work, type  X <return>  to quit.


LaTeX Warning: File `Fig/cloDistrib30.pdf' not found on input line 402.


! Package pdftex.def Error: File `Fig/cloDistrib30.pdf' not found: using
draft
setting.

See the pdftex.def package documentation for explanation.
Type  H <return>  for immediate help.
 ...

l.402 ...dth=0.35\linewidth]{Fig/cloDistrib30.pdf}
                                                  }
Try typing  <return>  to proceed.
If that doesn't work, type  X <return>  to quit.


LaTeX Warning: File `Fig/cloDistrib519.pdf' not found on input line 403.


! Package pdftex.def Error: File `Fig/cloDistrib519.pdf' not found: using
draft
 setting.

See the pdftex.def package documentation for explanation.
Type  H <return>  for immediate help.
 ...

l.403 ...th=0.35\linewidth]{Fig/cloDistrib519.pdf}
                                                  } \\ e) \hspace{5.5cm}
f)
Try typing  <return>  to proceed.
If that doesn't work, type  X <return>  to quit.


Package natbib Warning: Citation `udrescu2016clustering' on page 8
undefined on
 input line 404.


LaTeX Warning: File `Fig/degDistrib30-DTI-a.pdf' not found on input line
411.


! Package pdftex.def Error: File `Fig/degDistrib30-DTI-a.pdf' not found:
using
draft setting.

See the pdftex.def package documentation for explanation.
Type  H <return>  for immediate help.
 ...
```

```
l.411 ...35\linewidth]{Fig/degDistrib30-DTI-a.pdf}
                                                  }
Try typing  <return>  to proceed.
If that doesn't work, type  X <return>  to quit.


LaTeX Warning: File `Fig/degDistrib519-DTI-a.pdf' not found on input line
412.


! Package pdftex.def Error: File `Fig/degDistrib519-DTI-a.pdf' not found:
using
 draft setting.

See the pdftex.def package documentation for explanation.
Type  H <return>  for immediate help.
 ...

l.412 ...5\linewidth]{Fig/degDistrib519-DTI-a.pdf}
                                                  } \\ a) \hspace{5.5cm}
b) ...

Try typing  <return>  to proceed.
If that doesn't work, type  X <return>  to quit.


LaTeX Warning: File `Fig/degDistrib30-DTI-d.pdf' not found on input line
413.


! Package pdftex.def Error: File `Fig/degDistrib30-DTI-d.pdf' not found:
using
draft setting.

See the pdftex.def package documentation for explanation.
Type  H <return>  for immediate help.
 ...

l.413 ...35\linewidth]{Fig/degDistrib30-DTI-d.pdf}
                                                  }
Try typing  <return>  to proceed.
If that doesn't work, type  X <return>  to quit.


LaTeX Warning: File `Fig/degDistrib519-DTI-d.pdf' not found on input line
414.


! Package pdftex.def Error: File `Fig/degDistrib519-DTI-d.pdf' not found:
using
 draft setting.

See the pdftex.def package documentation for explanation.
```

```
Type  H <return>  for immediate help.
 ...

l.414 ...5\linewidth]{Fig/degDistrib519-DTI-d.pdf}
                                                  } \\ c) \hspace{5.5cm}
d)
Try typing  <return>  to proceed.
If that doesn't work, type  X <return>  to quit.


LaTeX Warning: File `Fig/degDistrib30-DTI-t.pdf' not found on input line
416.


! Package pdftex.def Error: File `Fig/degDistrib30-DTI-t.pdf' not found:
using
draft setting.

See the pdftex.def package documentation for explanation.
Type  H <return>  for immediate help.
 ...

l.416 ...35\linewidth]{Fig/degDistrib30-DTI-t.pdf}
                                                  }
Try typing  <return>  to proceed.
If that doesn't work, type  X <return>  to quit.


LaTeX Warning: File `Fig/degDistrib519-DTI-t.pdf' not found on input line
417.


! Package pdftex.def Error: File `Fig/degDistrib519-DTI-t.pdf' not found:
using
 draft setting.

See the pdftex.def package documentation for explanation.
Type  H <return>  for immediate help.
 ...

l.417 ...5\linewidth]{Fig/degDistrib519-DTI-t.pdf}
                                                  } \\ e) \hspace{5.5cm}
f)
Try typing  <return>  to proceed.
If that doesn't work, type  X <return>  to quit.


LaTeX Warning: File `Fig/betDistrib30-DTI.pdf' not found on input line
419.


! Package pdftex.def Error: File `Fig/betDistrib30-DTI.pdf' not found:
using dr
aft setting.
```

```
See the pdftex.def package documentation for explanation.
Type  H <return>  for immediate help.
 ...

l.419 ...0.35\linewidth]{Fig/betDistrib30-DTI.pdf}
                                                  }
Try typing  <return>  to proceed.
If that doesn't work, type  X <return>  to quit.


LaTeX Warning: File `Fig/betDistrib519-DTI.pdf' not found on input line
420.


! Package pdftex.def Error: File `Fig/betDistrib519-DTI.pdf' not found:
using d
raft setting.

See the pdftex.def package documentation for explanation.
Type  H <return>  for immediate help.
 ...

l.420 ....35\linewidth]{Fig/betDistrib519-DTI.pdf}
                                                  } \\ g) \hspace{5.5cm}
h)
Try typing  <return>  to proceed.
If that doesn't work, type  X <return>  to quit.


Package natbib Warning: Citation `alstott2014powerlaw' on page 8
undefined on i
nput line 421.


LaTeX Warning: Float too large for page by 82.73116pt on input line 422.


LaTeX Warning: File `Fig/eigDistrib30-DTI.pdf' not found on input line
425.


! Package pdftex.def Error: File `Fig/eigDistrib30-DTI.pdf' not found:
using dr
aft setting.

See the pdftex.def package documentation for explanation.
Type  H <return>  for immediate help.
 ...

l.425 ...0.35\linewidth]{Fig/eigDistrib30-DTI.pdf}
                                                  }
Try typing  <return>  to proceed.
If that doesn't work, type  X <return>  to quit.
```

```
LaTeX Warning: File `Fig/eigDistrib519-DTI.pdf' not found on input line
426.


! Package pdftex.def Error: File `Fig/eigDistrib519-DTI.pdf' not found:
using d
raft setting.

See the pdftex.def package documentation for explanation.
Type  H <return>  for immediate help.
 ...

l.426 ....35\linewidth]{Fig/eigDistrib519-DTI.pdf}
                                                  } \\ a) \hspace{5.5cm}
b) ...

Try typing  <return>  to proceed.
If that doesn't work, type  X <return>  to quit.


LaTeX Warning: File `Fig/pagDistrib30-DTI.pdf' not found on input line
427.


! Package pdftex.def Error: File `Fig/pagDistrib30-DTI.pdf' not found:
using dr
aft setting.

See the pdftex.def package documentation for explanation.
Type  H <return>  for immediate help.
 ...

l.427 ...0.35\linewidth]{Fig/pagDistrib30-DTI.pdf}
                                                  }
Try typing  <return>  to proceed.
If that doesn't work, type  X <return>  to quit.


LaTeX Warning: File `Fig/pagDistrib519-DTI.pdf' not found on input line
428.


! Package pdftex.def Error: File `Fig/pagDistrib519-DTI.pdf' not found:
using d
raft setting.

See the pdftex.def package documentation for explanation.
Type  H <return>  for immediate help.
 ...

l.428 ....35\linewidth]{Fig/pagDistrib519-DTI.pdf}
```

```
                                                  } \\ c) \hspace{5.5cm}
d)
Try typing  <return>  to proceed.
If that doesn't work, type  X <return>  to quit.


LaTeX Warning: File `Fig/cloDistrib30-DTI.pdf' not found on input line
430.


! Package pdftex.def Error: File `Fig/cloDistrib30-DTI.pdf' not found:
using dr
aft setting.

See the pdftex.def package documentation for explanation.
Type  H <return>  for immediate help.
 ...

l.430 ...0.35\linewidth]{Fig/cloDistrib30-DTI.pdf}
                                                  }
Try typing  <return>  to proceed.
If that doesn't work, type  X <return>  to quit.


LaTeX Warning: File `Fig/cloDistrib519-DTI.pdf' not found on input line
431.


! Package pdftex.def Error: File `Fig/cloDistrib519-DTI.pdf' not found:
using d
raft setting.

See the pdftex.def package documentation for explanation.
Type  H <return>  for immediate help.
 ...

l.431 ....35\linewidth]{Fig/cloDistrib519-DTI.pdf}
                                                  } \\ e) \hspace{5.5cm}
f)
Try typing  <return>  to proceed.
If that doesn't work, type  X <return>  to quit.


Package natbib Warning: Citation `morzy2016benford' on page 8 undefined
on inpu
t line 441.


Package natbib Warning: Citation `kossovsky2021mistaken' on page 8
undefined on
 input line 441.

[8]
```

```
LaTeX Warning: File `Fig/benford30-deg.pdf' not found on input line 449.


! Package pdftex.def Error: File `Fig/benford30-deg.pdf' not found: using
draft
 setting.

See the pdftex.def package documentation for explanation.
Type  H <return>  for immediate help.
 ...

l.449 ...th=0.35\linewidth]{Fig/benford30-deg.pdf}
                                                  }
Try typing  <return>  to proceed.
If that doesn't work, type  X <return>  to quit.


LaTeX Warning: File `Fig/qq-benford30-deg.pdf' not found on input line
450.


! Package pdftex.def Error: File `Fig/qq-benford30-deg.pdf' not found:
using dr
aft setting.

See the pdftex.def package documentation for explanation.
Type  H <return>  for immediate help.
 ...

l.450 ...0.35\linewidth]{Fig/qq-benford30-deg.pdf}
                                                  } \\ \phantom{cccccc}a)
\h...

Try typing  <return>  to proceed.
If that doesn't work, type  X <return>  to quit.


LaTeX Warning: File `Fig/benford30-bet.pdf' not found on input line 451.


! Package pdftex.def Error: File `Fig/benford30-bet.pdf' not found: using
draft
 setting.

See the pdftex.def package documentation for explanation.
Type  H <return>  for immediate help.
 ...

l.451 ...th=0.35\linewidth]{Fig/benford30-bet.pdf}
                                                  }
{\includegraphics[width=...

Try typing  <return>  to proceed.
If that doesn't work, type  X <return>  to quit.
```

```
LaTeX Warning: File `Fig/qq-benford30-bet.pdf' not found on input line
451.


! Package pdftex.def Error: File `Fig/qq-benford30-bet.pdf' not found:
using dr
aft setting.

See the pdftex.def package documentation for explanation.
Type  H <return>  for immediate help.
 ...

l.451 ...0.35\linewidth]{Fig/qq-benford30-bet.pdf}
                                                  } \\ \phantom{cccccc}c)
\h...

Try typing  <return>  to proceed.
If that doesn't work, type  X <return>  to quit.


LaTeX Warning: File `Fig/benford519-deg.pdf' not found on input line 456.


! Package pdftex.def Error: File `Fig/benford519-deg.pdf' not found:
using draf
t setting.

See the pdftex.def package documentation for explanation.
Type  H <return>  for immediate help.
 ...

l.456 ...h=0.35\linewidth]{Fig/benford519-deg.pdf}
                                                  }
Try typing  <return>  to proceed.
If that doesn't work, type  X <return>  to quit.


LaTeX Warning: File `Fig/qq-benford519-deg.pdf' not found on input line
457.


! Package pdftex.def Error: File `Fig/qq-benford519-deg.pdf' not found:
using d
raft setting.

See the pdftex.def package documentation for explanation.
Type  H <return>  for immediate help.
 ...

l.457 ....35\linewidth]{Fig/qq-benford519-deg.pdf}
                                                  } \\ \phantom{cccccc}a)
\h...
```

```
Try typing  <return>  to proceed.
If that doesn't work, type  X <return>  to quit.


LaTeX Warning: File `Fig/benford519-bet.pdf' not found on input line 458.


! Package pdftex.def Error: File `Fig/benford519-bet.pdf' not found:
using draf
t setting.

See the pdftex.def package documentation for explanation.
Type  H <return>  for immediate help.
 ...

l.458 ...h=0.35\linewidth]{Fig/benford519-bet.pdf}
                                                  }
Try typing  <return>  to proceed.
If that doesn't work, type  X <return>  to quit.


LaTeX Warning: File `Fig/qq-benford519-bet.pdf' not found on input line
459.


! Package pdftex.def Error: File `Fig/qq-benford519-bet.pdf' not found:
using d
raft setting.

See the pdftex.def package documentation for explanation.
Type  H <return>  for immediate help.
 ...

l.459 ....35\linewidth]{Fig/qq-benford519-bet.pdf}
                                                  } \\ \phantom{cccccc}c)
\h...

Try typing  <return>  to proceed.
If that doesn't work, type  X <return>  to quit.


LaTeX Warning: File `Fig/degBenford.pdf' not found on input line 465.


! Package pdftex.def Error: File `Fig/degBenford.pdf' not found: using
draft se
tting.

See the pdftex.def package documentation for explanation.
Type  H <return>  for immediate help.
 ...

l.465 ...width=0.48\linewidth]{Fig/degBenford.pdf}
```

```
                                                  }
Try typing  <return>  to proceed.
If that doesn't work, type  X <return>  to quit.


LaTeX Warning: File `Fig/degBenfordPearson.pdf' not found on input line
466.


! Package pdftex.def Error: File `Fig/degBenfordPearson.pdf' not found:
using d
raft setting.

See the pdftex.def package documentation for explanation.
Type  H <return>  for immediate help.
 ...

l.466 ....48\linewidth]{Fig/degBenfordPearson.pdf}
                                                  } \\ \phantom{cccccc}a)
\h...

Try typing  <return>  to proceed.
If that doesn't work, type  X <return>  to quit.


LaTeX Warning: File `Fig/betBenford.pdf' not found on input line 467.


! Package pdftex.def Error: File `Fig/betBenford.pdf' not found: using
draft se
tting.

See the pdftex.def package documentation for explanation.
Type  H <return>  for immediate help.
 ...

l.467 ...width=0.48\linewidth]{Fig/betBenford.pdf}
                                                  }
{\includegraphics[width=...

Try typing  <return>  to proceed.
If that doesn't work, type  X <return>  to quit.


LaTeX Warning: File `Fig/betBenfordPearson.pdf' not found on input line
467.


! Package pdftex.def Error: File `Fig/betBenfordPearson.pdf' not found:
using d
raft setting.

See the pdftex.def package documentation for explanation.
Type  H <return>  for immediate help.
```

```
 ...

l.467 ....48\linewidth]{Fig/betBenfordPearson.pdf}
                                                  } \\ \phantom{cccccc}c)
\h...

Try typing  <return>  to proceed.
If that doesn't work, type  X <return>  to quit.


LaTeX Warning: File `Fig/dti-benford-30-degree-d.pdf' not found on input
line 4
80.


! Package pdftex.def Error: File `Fig/dti-benford-30-degree-d.pdf' not
found: u
sing draft setting.

See the pdftex.def package documentation for explanation.
Type  H <return>  for immediate help.
 ...

l.480 ...newidth]{Fig/dti-benford-30-degree-d.pdf}
                                                  }
Try typing  <return>  to proceed.
If that doesn't work, type  X <return>  to quit.


LaTeX Warning: File `Fig/dti-qq-benford-30-degree-d.pdf' not found on
input lin
e 481.


! Package pdftex.def Error: File `Fig/dti-qq-benford-30-degree-d.pdf' not
found
: using draft setting.

See the pdftex.def package documentation for explanation.
Type  H <return>  for immediate help.
 ...

l.481 ...idth]{Fig/dti-qq-benford-30-degree-d.pdf}
                                                  } \\ \phantom{cccccc}a)
\h...

Try typing  <return>  to proceed.
If that doesn't work, type  X <return>  to quit.


LaTeX Warning: File `Fig/dti-benford-30-degree-t.pdf' not found on input
line 4
82.
```

```
! Package pdftex.def Error: File `Fig/dti-benford-30-degree-t.pdf' not
found: u
sing draft setting.

See the pdftex.def package documentation for explanation.
Type  H <return>  for immediate help.
 ...

l.482 ...newidth]{Fig/dti-benford-30-degree-t.pdf}
                                                  }
{\includegraphics[width=...

Try typing  <return>  to proceed.
If that doesn't work, type  X <return>  to quit.


LaTeX Warning: File `Fig/dti-qq-benford-30-degree-t.pdf' not found on
input lin
e 482.


! Package pdftex.def Error: File `Fig/dti-qq-benford-30-degree-t.pdf' not
found
: using draft setting.

See the pdftex.def package documentation for explanation.
Type  H <return>  for immediate help.
 ...

l.482 ...idth]{Fig/dti-qq-benford-30-degree-t.pdf}
                                                  } \\ \phantom{cccccc}c)
\h...

Try typing  <return>  to proceed.
If that doesn't work, type  X <return>  to quit.


LaTeX Warning: File `Fig/dti-benford-30-degree-a.pdf' not found on input
line 4
83.


! Package pdftex.def Error: File `Fig/dti-benford-30-degree-a.pdf' not
found: u
sing draft setting.

See the pdftex.def package documentation for explanation.
Type  H <return>  for immediate help.
 ...

l.483 ...newidth]{Fig/dti-benford-30-degree-a.pdf}
                                                  }
{\includegraphics[width=...
```

```
Try typing  <return>  to proceed.
If that doesn't work, type  X <return>  to quit.


LaTeX Warning: File `Fig/dti-qq-benford-30-degree-a.pdf' not found on
input lin
e 483.


! Package pdftex.def Error: File `Fig/dti-qq-benford-30-degree-a.pdf' not
found
: using draft setting.

See the pdftex.def package documentation for explanation.
Type  H <return>  for immediate help.
 ...

l.483 ...idth]{Fig/dti-qq-benford-30-degree-a.pdf}
                                                  } \\ \phantom{cccccc}e)
\h...

Try typing  <return>  to proceed.
If that doesn't work, type  X <return>  to quit.


LaTeX Warning: File `Fig/dti-benford-519-degree-d.pdf' not found on input
line
488.


! Package pdftex.def Error: File `Fig/dti-benford-519-degree-d.pdf' not
found:
using draft setting.

See the pdftex.def package documentation for explanation.
Type  H <return>  for immediate help.
 ...

l.488 ...ewidth]{Fig/dti-benford-519-degree-d.pdf}
                                                  }
Try typing  <return>  to proceed.
If that doesn't work, type  X <return>  to quit.


LaTeX Warning: File `Fig/dti-qq-benford-519-degree-d.pdf' not found on
input li
ne 489.


! Package pdftex.def Error: File `Fig/dti-qq-benford-519-degree-d.pdf'
not foun
d: using draft setting.
```

```
See the pdftex.def package documentation for explanation.
Type  H <return>  for immediate help.
 ...

l.489 ...dth]{Fig/dti-qq-benford-519-degree-d.pdf}
                                                  } \\ \phantom{cccccc}a)
\h...

Try typing  <return>  to proceed.
If that doesn't work, type  X <return>  to quit.


LaTeX Warning: File `Fig/dti-benford-519-degree-t.pdf' not found on input
line
490.


! Package pdftex.def Error: File `Fig/dti-benford-519-degree-t.pdf' not
found:
using draft setting.

See the pdftex.def package documentation for explanation.
Type  H <return>  for immediate help.
 ...

l.490 ...ewidth]{Fig/dti-benford-519-degree-t.pdf}
                                                  }
{\includegraphics[width=...

Try typing  <return>  to proceed.
If that doesn't work, type  X <return>  to quit.


LaTeX Warning: File `Fig/dti-qq-benford-519-degree-t.pdf' not found on
input li
ne 490.


! Package pdftex.def Error: File `Fig/dti-qq-benford-519-degree-t.pdf'
not foun
d: using draft setting.

See the pdftex.def package documentation for explanation.
Type  H <return>  for immediate help.
 ...

l.490 ...dth]{Fig/dti-qq-benford-519-degree-t.pdf}
                                                  } \\ \phantom{cccccc}c)
\h...

Try typing  <return>  to proceed.
If that doesn't work, type  X <return>  to quit.
```

```
LaTeX Warning: File `Fig/dti-benford-519-degree-a.pdf' not found on input
line
491.


! Package pdftex.def Error: File `Fig/dti-benford-519-degree-a.pdf' not
found:
using draft setting.

See the pdftex.def package documentation for explanation.
Type  H <return>  for immediate help.
 ...

l.491 ...ewidth]{Fig/dti-benford-519-degree-a.pdf}
                                                  }
{\includegraphics[width=...

Try typing  <return>  to proceed.
If that doesn't work, type  X <return>  to quit.


LaTeX Warning: File `Fig/dti-qq-benford-519-degree-a.pdf' not found on
input li
ne 491.


! Package pdftex.def Error: File `Fig/dti-qq-benford-519-degree-a.pdf'
not foun
d: using draft setting.

See the pdftex.def package documentation for explanation.
Type  H <return>  for immediate help.
 ...

l.491 ...dth]{Fig/dti-qq-benford-519-degree-a.pdf}
                                                  } \\ \phantom{cccccc}e)
\h...

Try typing  <return>  to proceed.
If that doesn't work, type  X <return>  to quit.


LaTeX Warning: File `Fig/dti-benford-30-bet.pdf' not found on input line
496.


! Package pdftex.def Error: File `Fig/dti-benford-30-bet.pdf' not found:
using
draft setting.

See the pdftex.def package documentation for explanation.
Type  H <return>  for immediate help.
 ...
```

```
l.496 ...35\linewidth]{Fig/dti-benford-30-bet.pdf}
                                                  }
Try typing  <return>  to proceed.
If that doesn't work, type  X <return>  to quit.


LaTeX Warning: File `Fig/dti-qq-benford-30-bet.pdf' not found on input
line 497
.


! Package pdftex.def Error: File `Fig/dti-qq-benford-30-bet.pdf' not
found: usi
ng draft setting.

See the pdftex.def package documentation for explanation.
Type  H <return>  for immediate help.
 ...

l.497 ...linewidth]{Fig/dti-qq-benford-30-bet.pdf}
                                                  } \\ \phantom{cccccc}a)
\h...

Try typing  <return>  to proceed.
If that doesn't work, type  X <return>  to quit.


LaTeX Warning: File `Fig/dti-benford-519-bet.pdf' not found on input line
498.


! Package pdftex.def Error: File `Fig/dti-benford-519-bet.pdf' not found:
using
 draft setting.

See the pdftex.def package documentation for explanation.
Type  H <return>  for immediate help.
 ...

l.498 ...5\linewidth]{Fig/dti-benford-519-bet.pdf}
                                                  }
{\includegraphics[width=...

Try typing  <return>  to proceed.
If that doesn't work, type  X <return>  to quit.


LaTeX Warning: File `Fig/dti-qq-benford-519-bet.pdf' not found on input
line 49
8.


! Package pdftex.def Error: File `Fig/dti-qq-benford-519-bet.pdf' not
found: us
```

```
ing draft setting.

See the pdftex.def package documentation for explanation.
Type  H <return>  for immediate help.
 ...

l.498 ...inewidth]{Fig/dti-qq-benford-519-bet.pdf}
                                                  } \\ \phantom{cccccc}c)
\h...

Try typing  <return>  to proceed.
If that doesn't work, type  X <return>  to quit.


Underfull \vbox (badness 2478) has occurred while \output is active []


Package natbib Warning: Citation `bhardwaj2011performance' on page 9
undefined
on input line 505.


Package natbib Warning: Citation `christensen2005fast' on page 9
undefined on i
nput line 505.


Underfull \vbox (badness 1642) has occurred while \output is active []

[9]

LaTeX Warning: File `Fig/KendallDeg30.pdf' not found on input line 513.


! Package pdftex.def Error: File `Fig/KendallDeg30.pdf' not found: using
draft
setting.

See the pdftex.def package documentation for explanation.
Type  H <return>  for immediate help.
 ...

l.513 ...dth=0.48\linewidth]{Fig/KendallDeg30.pdf}
                                                  }
Try typing  <return>  to proceed.
If that doesn't work, type  X <return>  to quit.


LaTeX Warning: File `Fig/KendallDeg518.pdf' not found on input line 514.


! Package pdftex.def Error: File `Fig/KendallDeg518.pdf' not found: using
draft
 setting.
```

```
See the pdftex.def package documentation for explanation.
Type  H <return>  for immediate help.
 ...

l.514 ...th=0.48\linewidth]{Fig/KendallDeg518.pdf}
                                                  } \\ a) \hspace{8cm} b)
\\...

Try typing  <return>  to proceed.
If that doesn't work, type  X <return>  to quit.


LaTeX Warning: File `Fig/KendallDeg.pdf' not found on input line 515.


! Package pdftex.def Error: File `Fig/KendallDeg.pdf' not found: using
draft se
tting.

See the pdftex.def package documentation for explanation.
Type  H <return>  for immediate help.
 ...

l.515 ...width=0.48\linewidth]{Fig/KendallDeg.pdf}
                                                  }
Try typing  <return>  to proceed.
If that doesn't work, type  X <return>  to quit.


LaTeX Warning: File `Fig/KendallBet.pdf' not found on input line 516.


! Package pdftex.def Error: File `Fig/KendallBet.pdf' not found: using
draft se
tting.

See the pdftex.def package documentation for explanation.
Type  H <return>  for immediate help.
 ...

l.516 ...width=0.48\linewidth]{Fig/KendallBet.pdf}
                                                  } \\ c) \hspace{8cm} d)
Try typing  <return>  to proceed.
If that doesn't work, type  X <return>  to quit.


Underfull \vbox (badness 1383) has occurred while \output is active []


LaTeX Warning: File `Fig/DB30alphaRU.pdf' not found on input line 525.
```

```
! Package pdftex.def Error: File `Fig/DB30alphaRU.pdf' not found: using
draft s
etting.

See the pdftex.def package documentation for explanation.
Type  H <return>  for immediate help.
 ...

l.525 ...idth=0.48\linewidth]{Fig/DB30alphaRU.pdf}
                                                  }
Try typing  <return>  to proceed.
If that doesn't work, type  X <return>  to quit.


LaTeX Warning: File `Fig/DB518alphaRU.pdf' not found on input line 526.


! Package pdftex.def Error: File `Fig/DB518alphaRU.pdf' not found: using
draft
setting.

See the pdftex.def package documentation for explanation.
Type  H <return>  for immediate help.
 ...

l.526 ...dth=0.48\linewidth]{Fig/DB518alphaRU.pdf}
                                                  } \\ a) \hspace{8cm} b)
Try typing  <return>  to proceed.
If that doesn't work, type  X <return>  to quit.


Underfull \vbox (badness 4872) has occurred while \output is active []

[10]
Underfull \vbox (badness 10000) has occurred while \output is active []


Package natbib Warning: Citation `tilson2016recommendations' on page 11
undefin
ed on input line 542.

[11]

Package natbib Warning: Citation `dumbreck2015drug' on page 12 undefined
on inp
ut line 544.


Package natbib Warning: Citation `monteith2020potential' on page 12
undefined o
n input line 544.
```

```
Package natbib Warning: Citation `phansalkar2013drug' on page 12
undefined on i
nput line 549.


Package natbib Warning: Citation `pirnejad2019preventing' on page 12
undefined
on input line 549.


Package natbib Warning: Citation `phansalkar2013criteria' on page 12
undefined
on input line 551.


Package natbib Warning: Citation `kontsioti2022reference' on page 12
undefined
on input line 551.


Package natbib Warning: Citation `assiri2022anti' on page 12 undefined on
input
 line 556.


Package natbib Warning: Citation `rohani2019drug' on page 12 undefined on
input
 line 558.


Package natbib Warning: Citation `avram2022novel' on page 12 undefined on
input
 line 566.


Package natbib Warning: Citation `chen2020prediction' on page 12
undefined on i
nput line 568.


Package natbib Warning: Citation `ye2021unified' on page 12 undefined on
input
line 568.


Package natbib Warning: Citation `hu2019predicting' on page 12 undefined
on inp
ut line 568.


Package natbib Warning: Citation `luo2017network' on page 12 undefined on
input
 line 568.
```

```
Package natbib Warning: Citation `thafar2020dtigems+' on page 12
undefined on i
nput line 568.


Package natbib Warning: Citation `lee2019deepconv' on page 12 undefined
on inpu
t line 568.


Package natbib Warning: Citation `hassanzadeh2022does' on page 12
undefined on
input line 568.

[12]

Package natbib Warning: Citation `zong2022beta' on page 13 undefined on
input l
ine 570.


Package natbib Warning: Citation `bagherian2021machine' on page 13
undefined on
 input line 577.


Package natbib Warning: Citation `chen2016drug' on page 13 undefined on
input l
ine 577.


Package natbib Warning: Citation `altman1995statistics' on page 13
undefined on
 input line 579.

[13]
Underfull \vbox (badness 10000) has occurred while \output is active []


Underfull \vbox (badness 10000) has occurred while \output is active []

[14]

Package natbib Warning: Citation `zong2022beta' on page 15 undefined on
input l
ine 587.


Package natbib Warning: Citation `panda2018wait' on page 15 undefined on
input
line 587.
```

```
Package natbib Warning: Citation `shi2019predicting' on page 15 undefined
on in
put line 589.


Package natbib Warning: Citation `udrescu2020uncovering' on page 15
undefined o
n input line 589.

No file main.bbl.

AED: lastpage setting LastPage
[15] [16] [17] [18] [19] [20] [21] [22] [23] [24] [25]

Package natbib Warning: There were undefined citations.

(./main.aux)

LaTeX Font Warning: Size substitutions with differences
(Font)              up to 1.32504pt have occurred.


LaTeX Font Warning: Some font shapes were not available, defaults
substituted.

Package rerunfilecheck Info: File `main.out' has not changed.
(rerunfilecheck)             Checksum:
63AC39A42C3ED6CA250C3FCF59F7A313;7758.
 )
Here is how much of TeX's memory you used:
 28408 strings out of 475066
 577895 string characters out of 5782772
 931286 words of memory out of 5000000
 48308 multiletter control sequences out of 15000+600000
 1989473 words of font info for 785 fonts, out of 8000000 for 9000
 1141 hyphenation exceptions out of 8191
 123i,13n,131p,1878b,1054s stack positions out of
10000i,1000n,20000p,200000b,200000s
{c:/TeXLive/2022/texmf-dist/fonts/enc/dvips/lm/lm-
ec.enc}{c:/TeXLive/2022/tex
mf-
dist/fonts/enc/dvips/merriweather/merriwthr_posqbl.enc}{c:/TeXLive/2022/t
exm
f-
dist/fonts/enc/dvips/merriweather/merriwthr_owzwzj.enc}<c:/TeXLive/2022/t
exmf
-dist/fonts/type1/sorkin/merriweather/Merriwthr-
Bold.pfb><c:/TeXLive/2022/texmf
-dist/fonts/type1/sorkin/merriweather/Merriwthr-
BoldItalic.pfb><c:/TeXLive/2022
/texmf-dist/fonts/type1/sorkin/merriweather/Merriwthr-
Italic.pfb><c:/TeXLive/20
22/texmf-dist/fonts/type1/sorkin/merriweather/Merriwthr-
Regular.pfb><c:/TeXLive
```

```
/2022/texmf-
dist/fonts/type1/public/amsfonts/cmextra/cmex8.pfb><c:/TeXLive/2022
/texmf-
dist/fonts/type1/public/amsfonts/cm/cmsy5.pfb><c:/TeXLive/2022/texmf-dis
t/fonts/type1/public/amsfonts/cm/cmsy6.pfb><c:/TeXLive/2022/texmf-
dist/fonts/ty
pe1/public/amsfonts/cm/cmsy7.pfb><c:/TeXLive/2022/texmf-
dist/fonts/type1/public
/amsfonts/euler/euex8.pfb><c:/TeXLive/2022/texmf-
dist/fonts/type1/public/amsfon
ts/euler/eurb7.pfb><c:/TeXLive/2022/texmf-
dist/fonts/type1/public/amsfonts/eule
r/eurm7.pfb><c:/TeXLive/2022/texmf-
dist/fonts/type1/public/amsfonts/euler/eusm7
.pfb><c:/TeXLive/2022/texmf-
dist/fonts/type1/public/lm/lmtt8.pfb><c:/TeXLive/20
22/texmf-dist/fonts/type1/public/amsfonts/symbols/msbm7.pfb>
Output written on main.pdf (25 pages, 467121 bytes).
PDF statistics:
 481 PDF objects out of 1000 (max. 8388607)
 416 compressed objects within 5 object streams
 103 named destinations out of 1000 (max. 500000)
 232259 words of extra memory for PDF output out of 266212 (max.
10000000)
```

Click here to access/download;LaTeX - Figure (eps, ps, etc.);ad-ddi.pdf

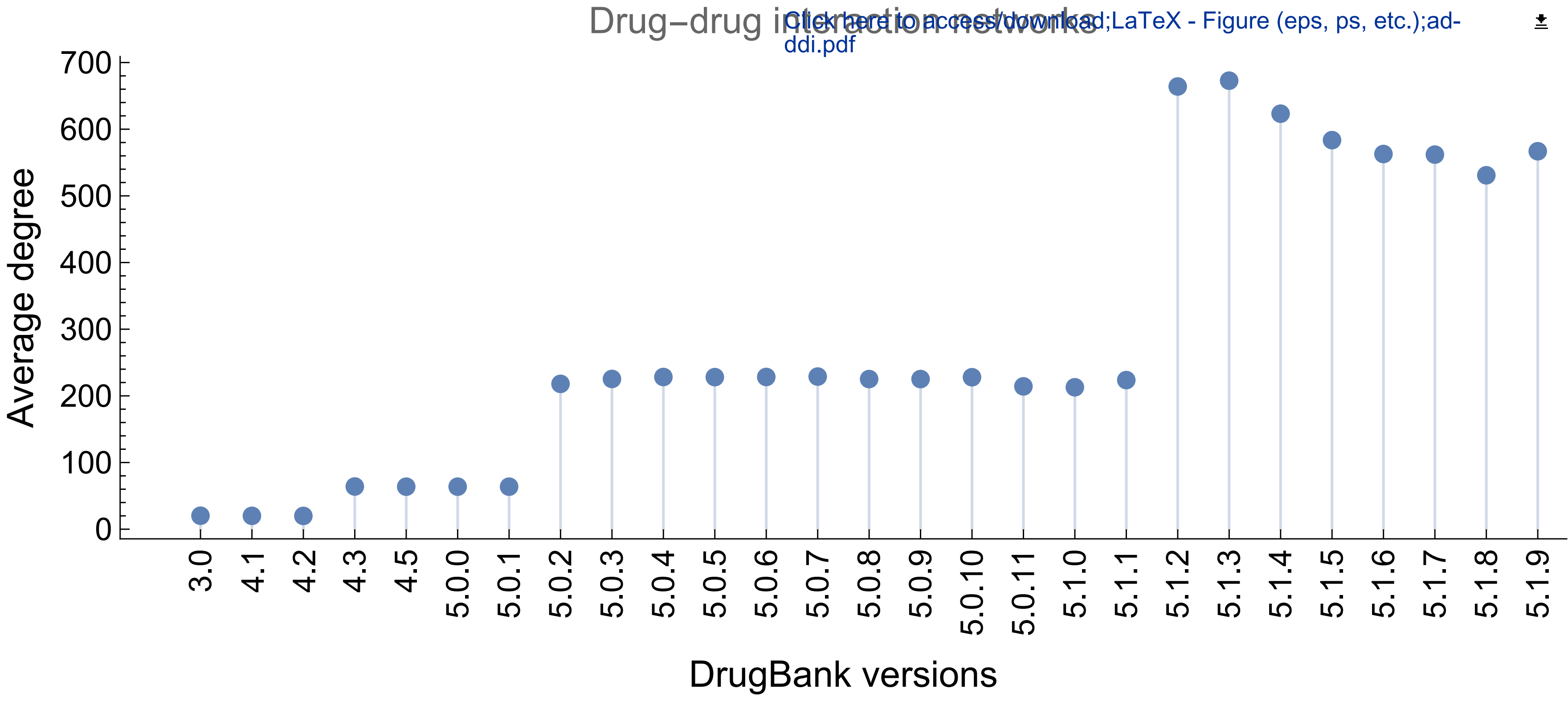

Click here to access/download;LaTeX - Figure (eps, ps, etc.);ad-dti.pdf

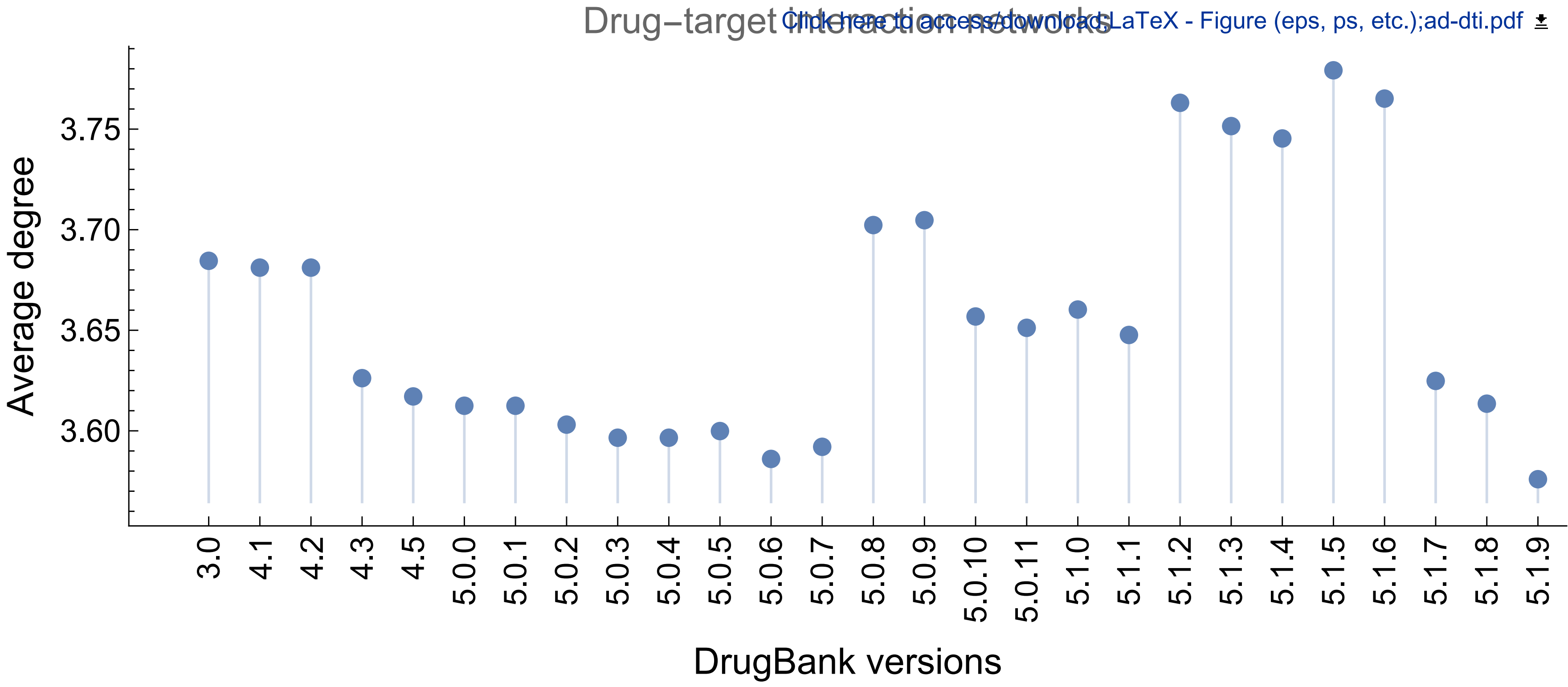

Click here to access/download;LaTeX - Figure (eps, ps, etc.);apl.pdf

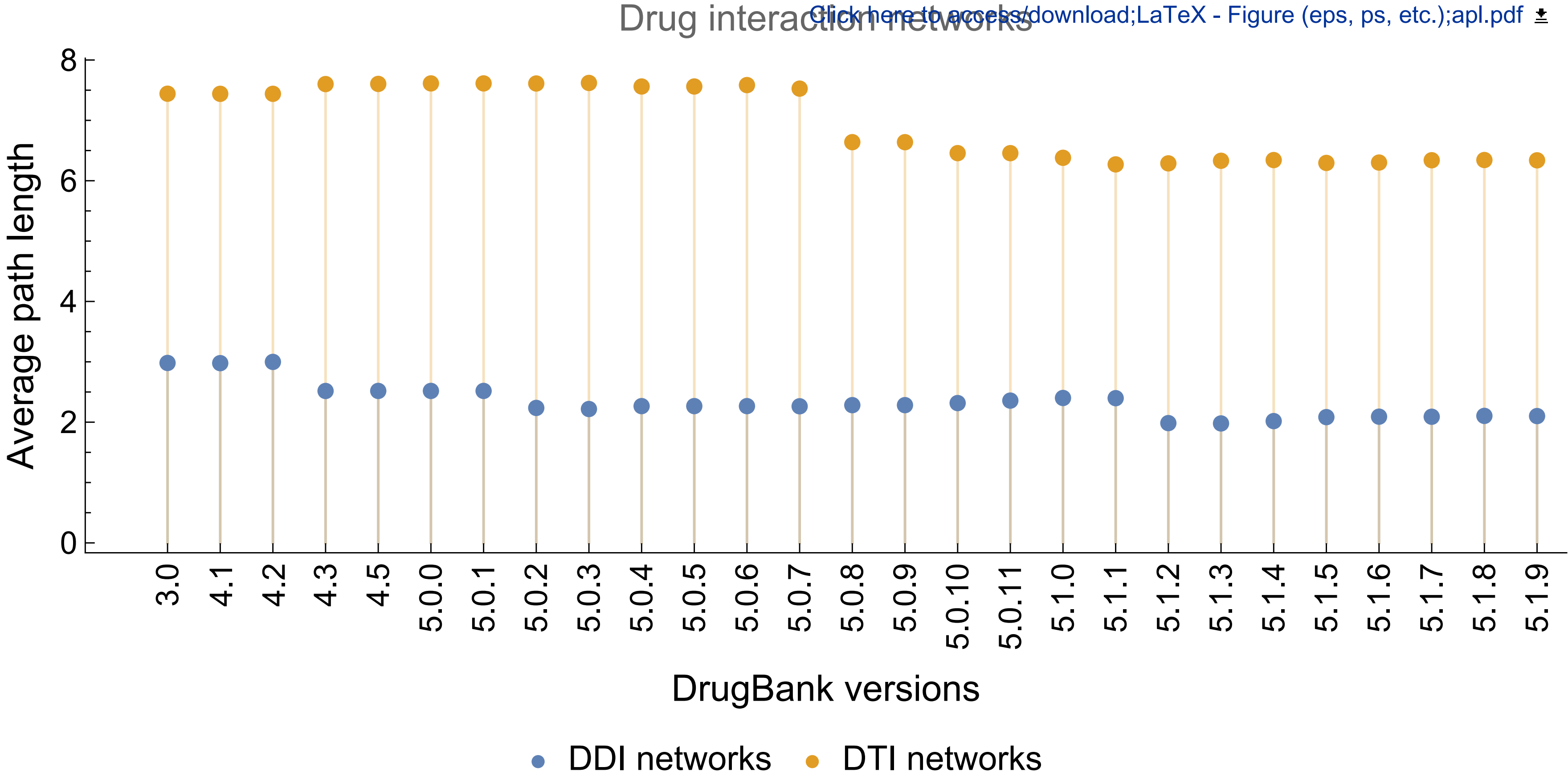

Click here to access/download;LaTeX - Figure (eps, ps, etc.);benford30-bet.pdf

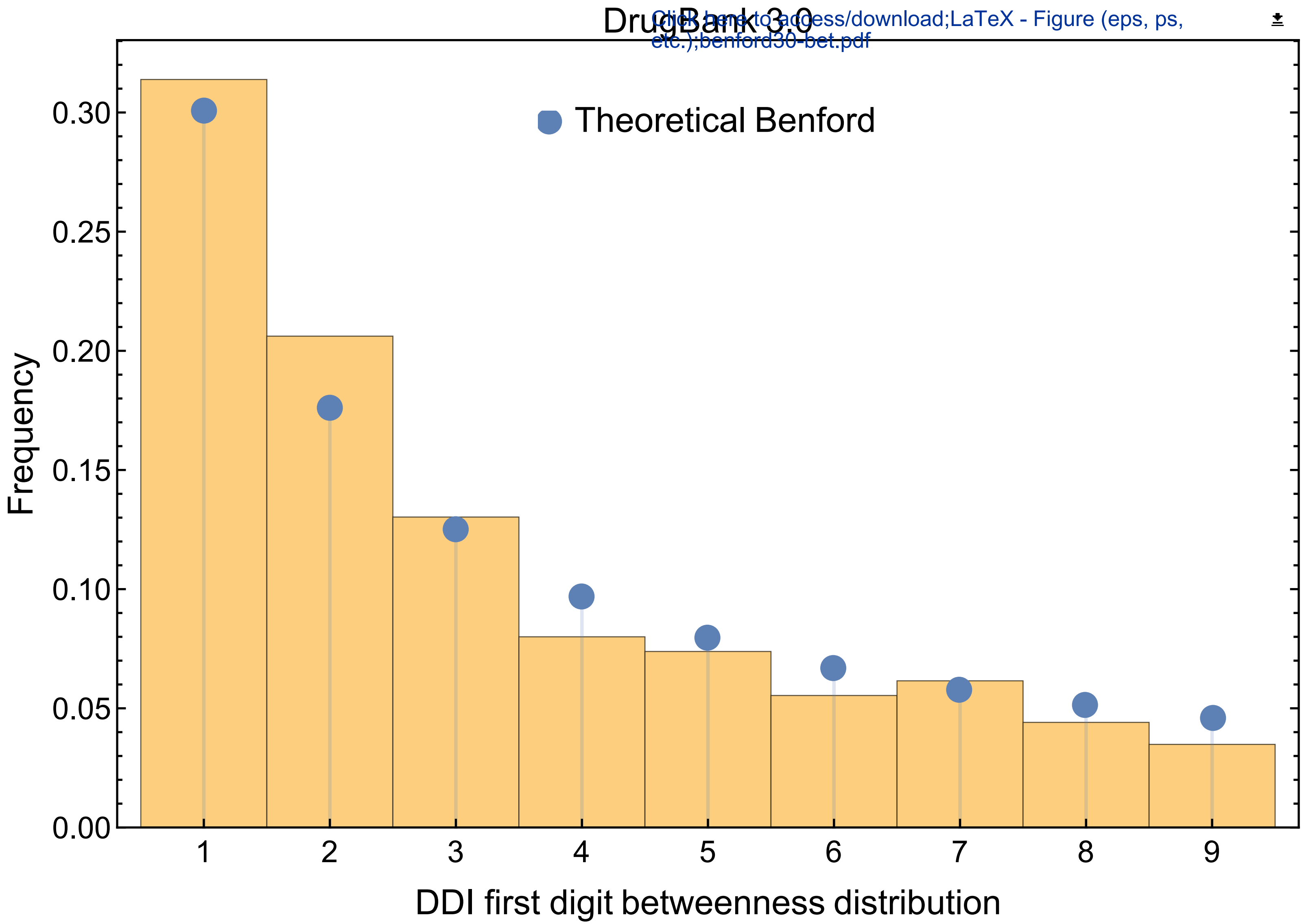

Click here to access/download;LaTeX - Figure (eps, ps, etc.);benford30-deg.pdf

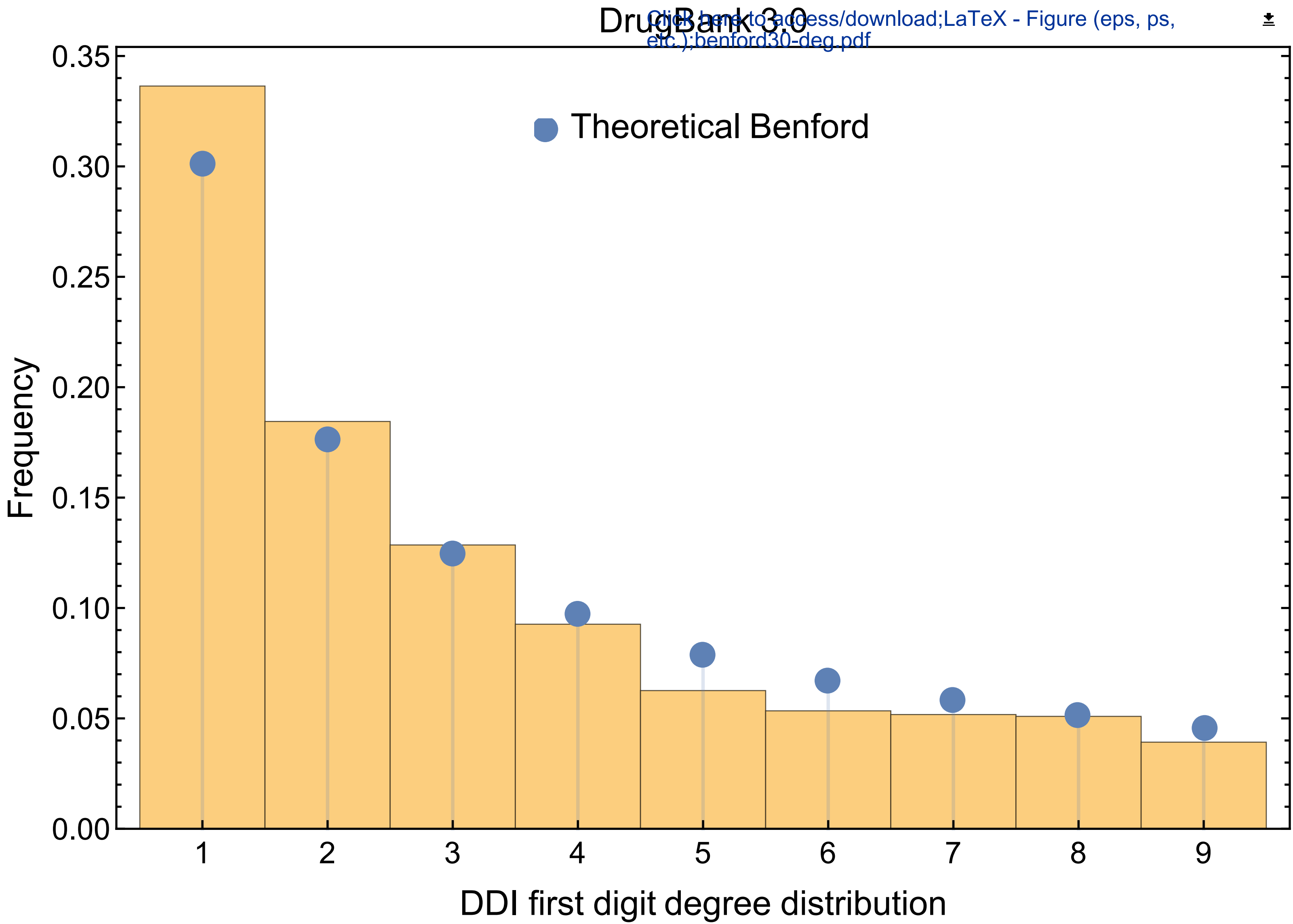

Click here to access/download;LaTeX - Figure (eps, ps, etc.);benford519-bet.pdf

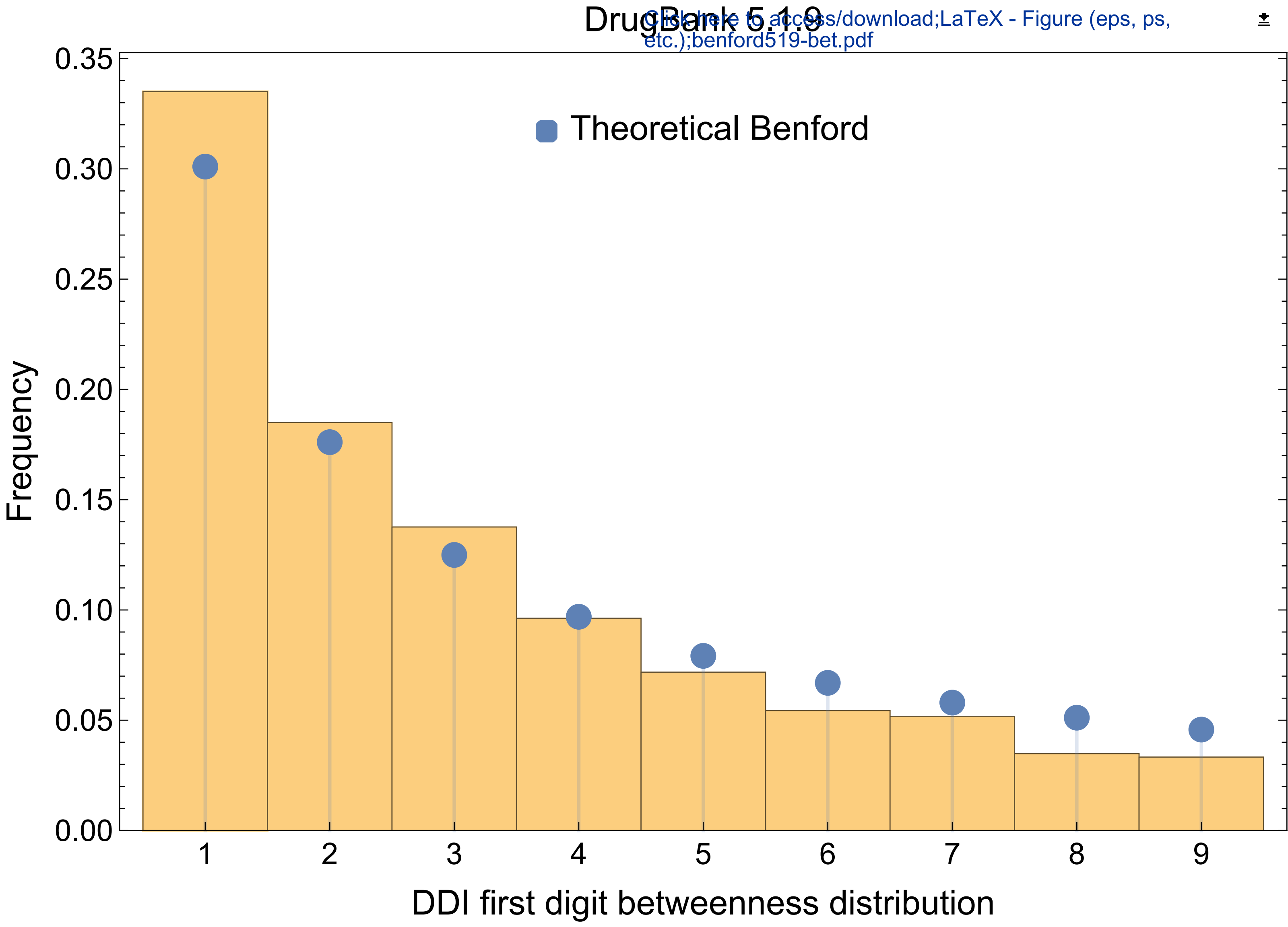

Click here to access/download;LaTeX - Figure (eps, ps, etc.);benford519-deg.pdf

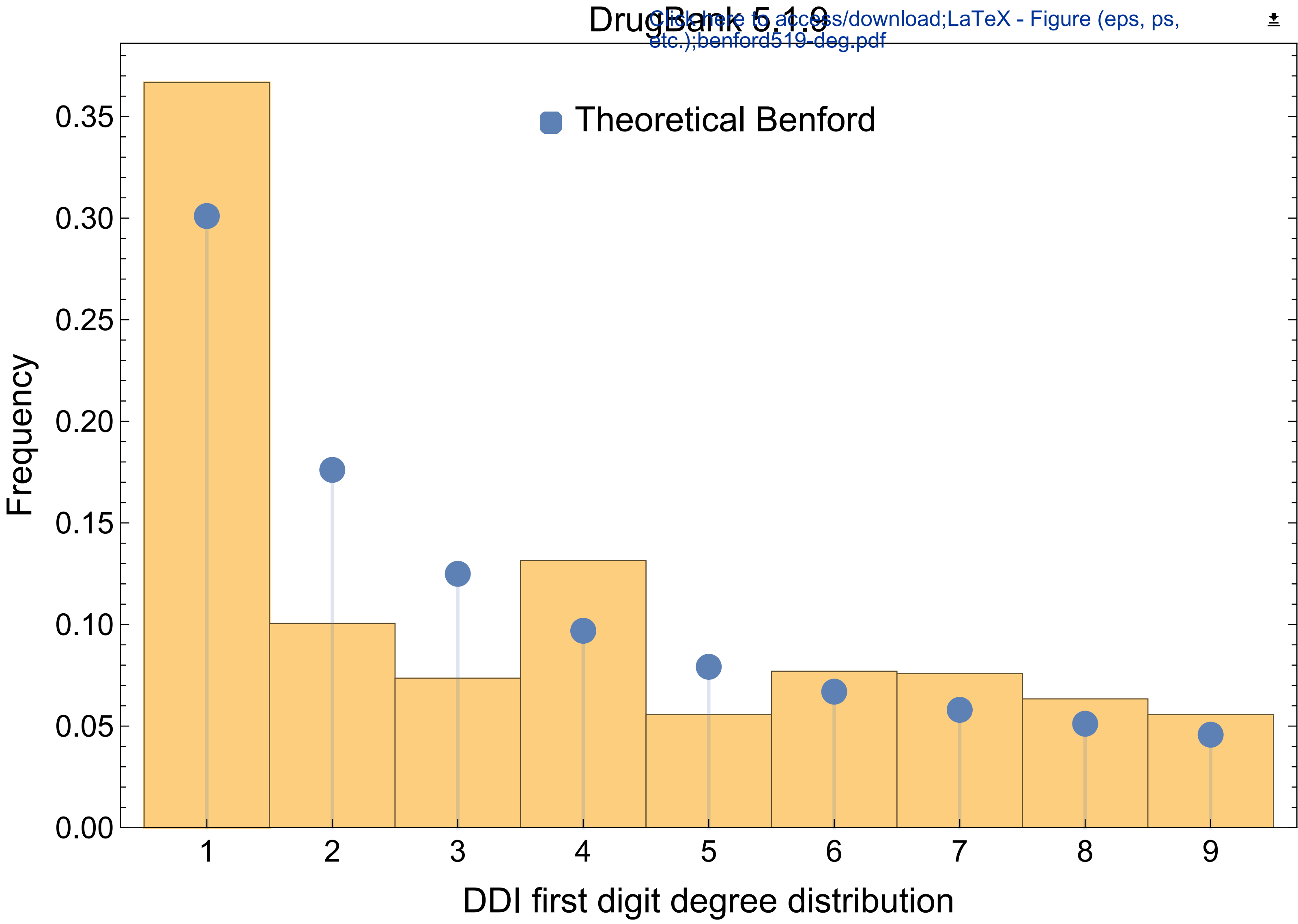

Click here to access/download;LaTeX - Figure (eps, ps, etc.);betBenford.pdf

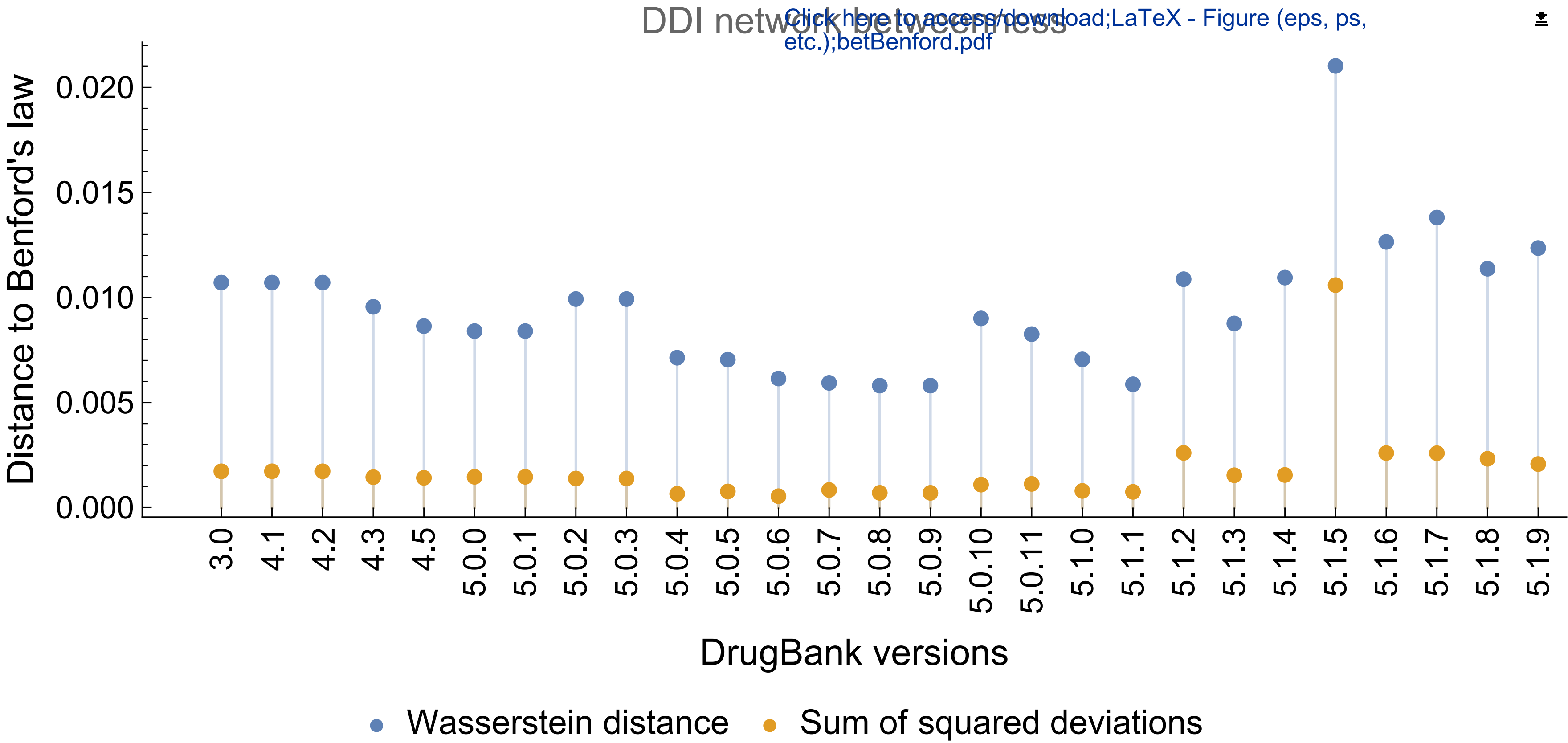

Click here to access/download;LaTeX - Figure (eps, ps, etc.);betBenfordPearson.pdf

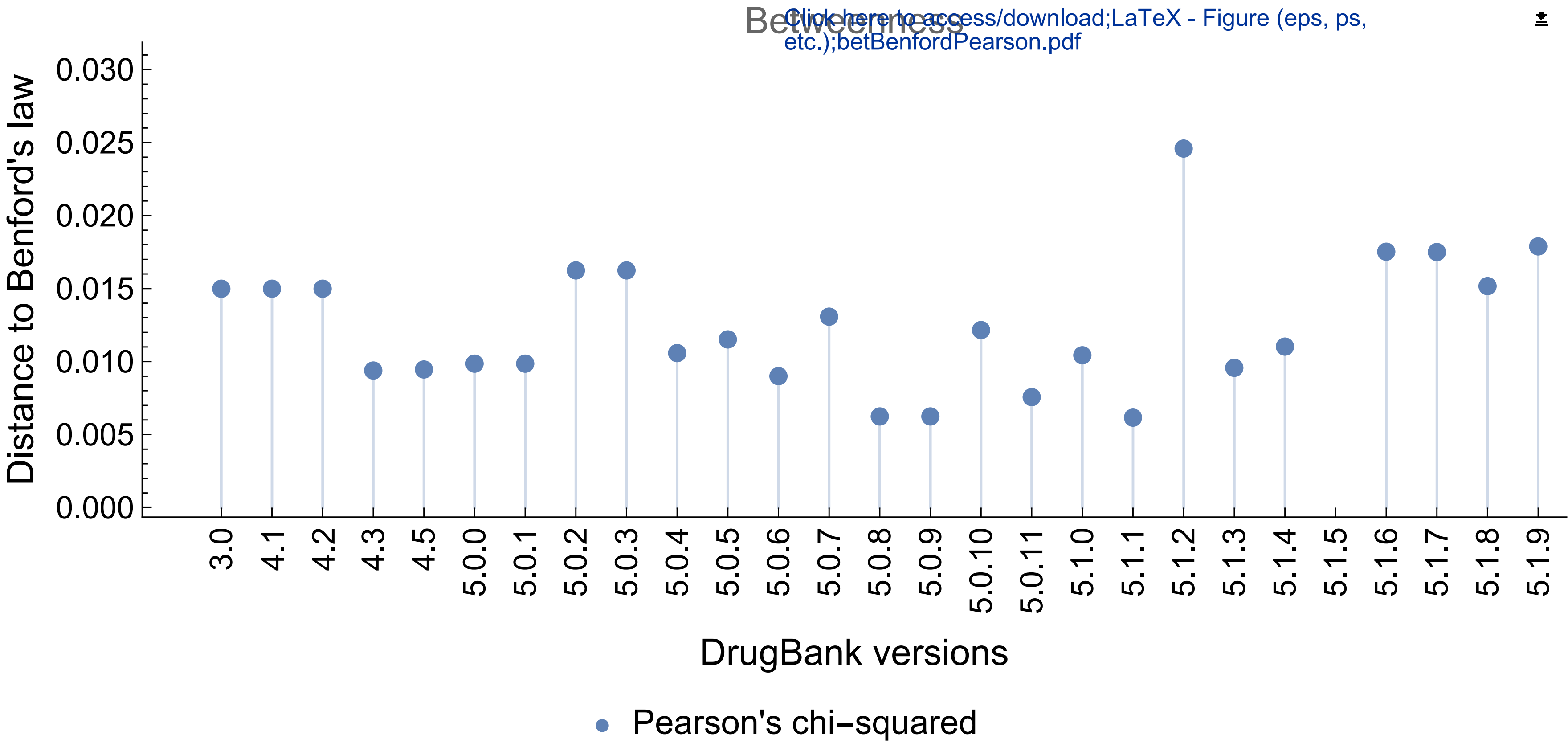

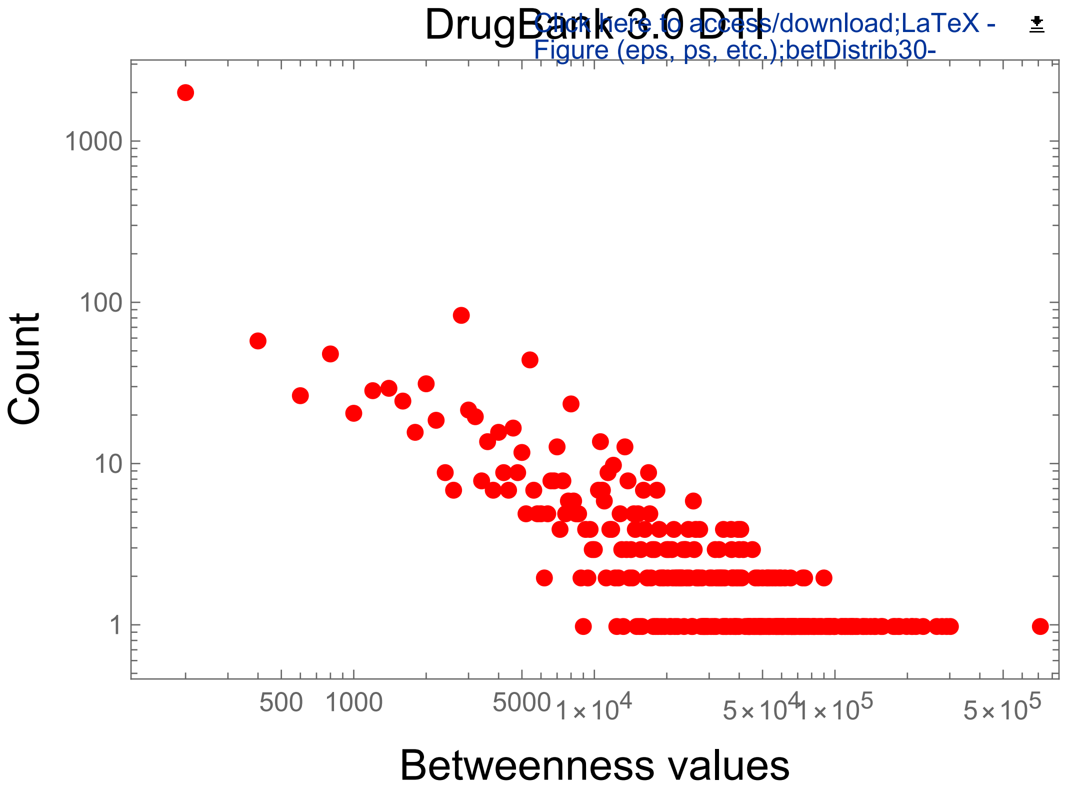

Click here to access/download;LaTeX - Figure (eps, ps, etc.);betDistrib30-

Click here to access/download;LaTeX - Figure (eps, ps, etc.);betDistrib30.pdf

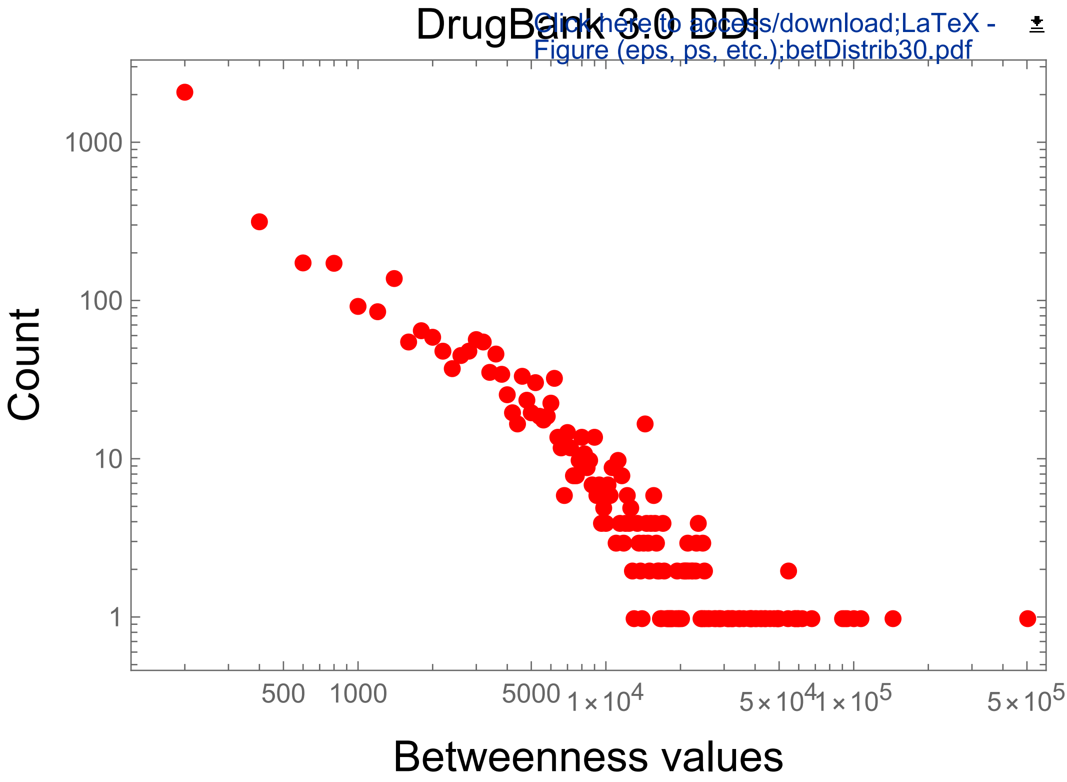

Click here to access/download;LaTeX - Figure (eps, ps, etc.);betDistrib519-

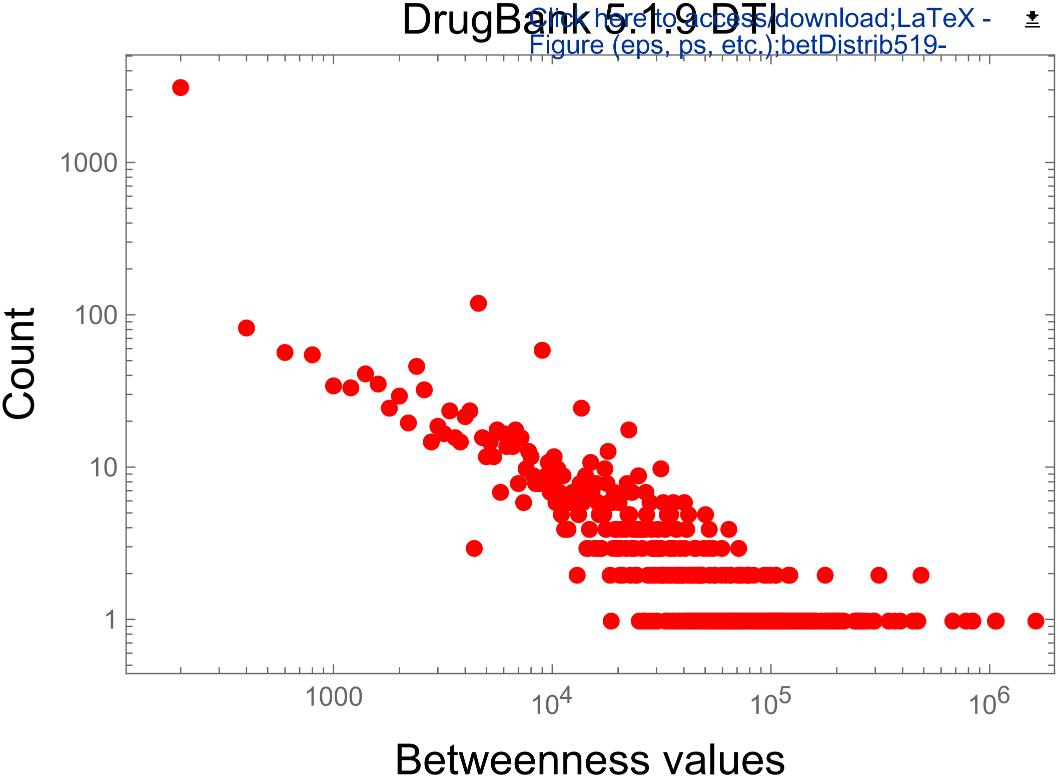

Click here to access/download;LaTeX - Figure (eps, ps, etc.);betDistrib519.pdf

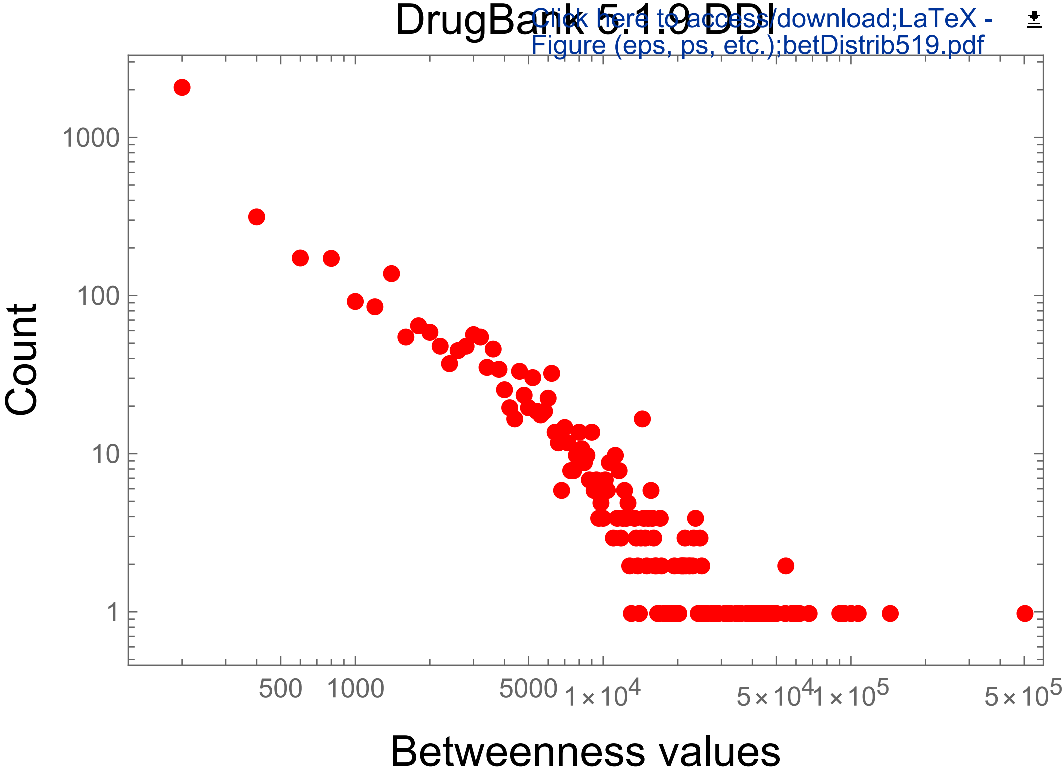

Click here to access/download;LaTeX - Figure (eps, ps, etc.);cc-ddi.pdf

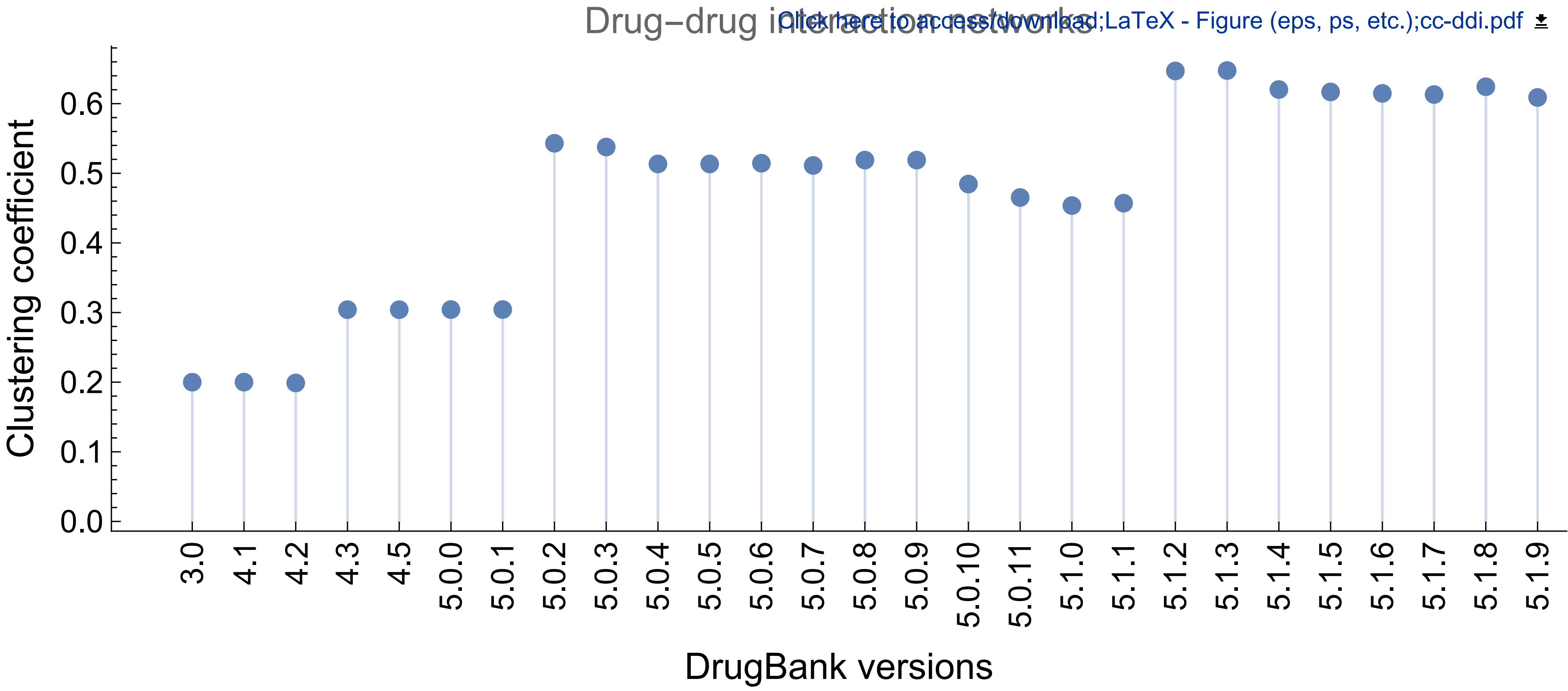

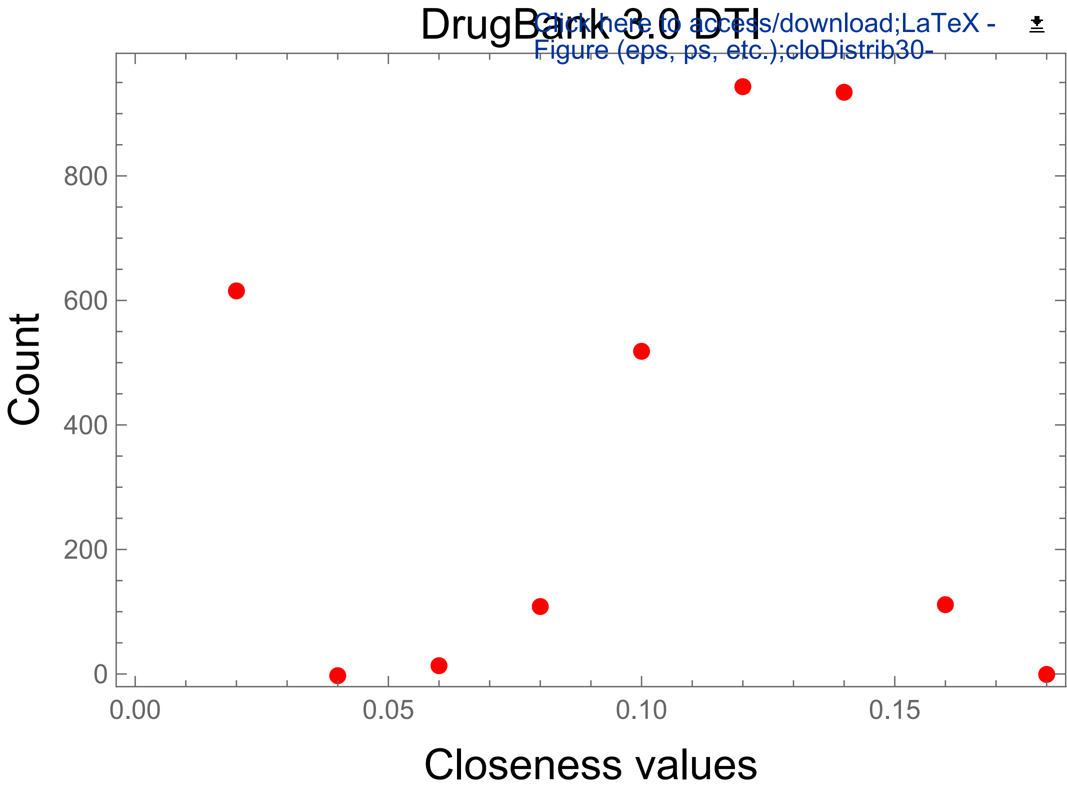
DrugBank 3.0 DTI
Click here to access/download;LaTeX - Figure (eps, ps, etc.);cloDistrib30-
Count
800
600
400
200
0
0.00
0.05
0.10
0.15
Closeness values

Click here to access/download;LaTeX - Figure (eps, ps, etc.);cloDistrib30.pdf

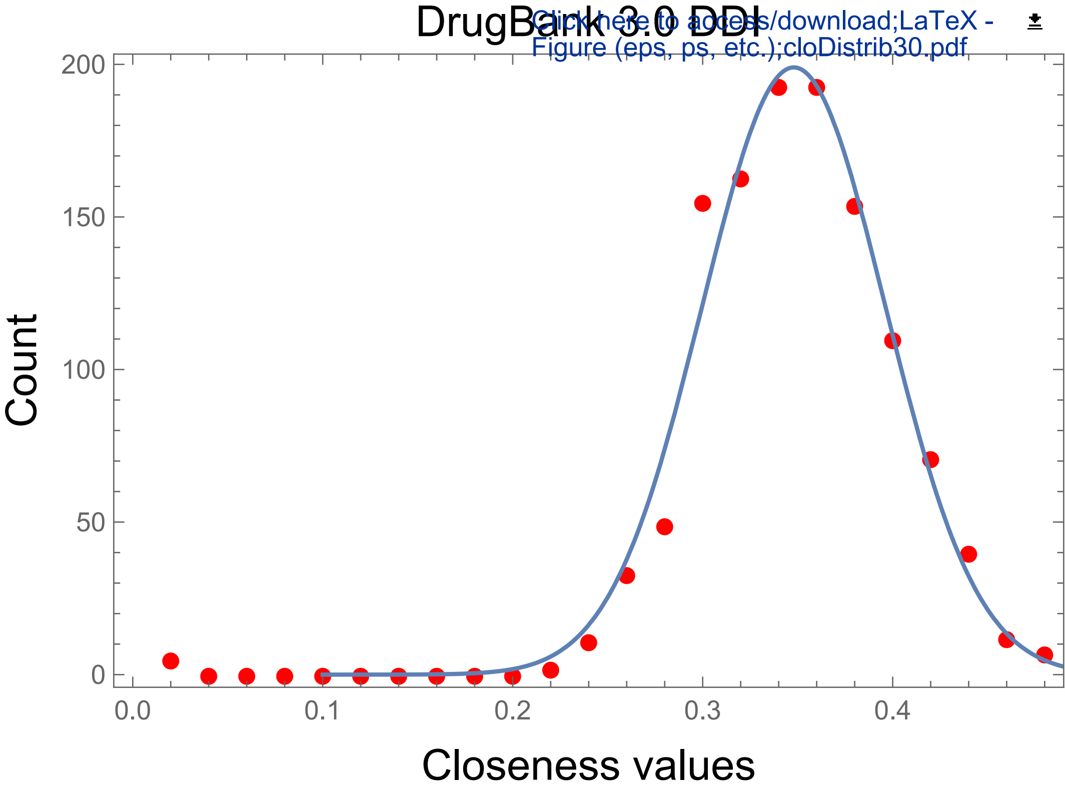

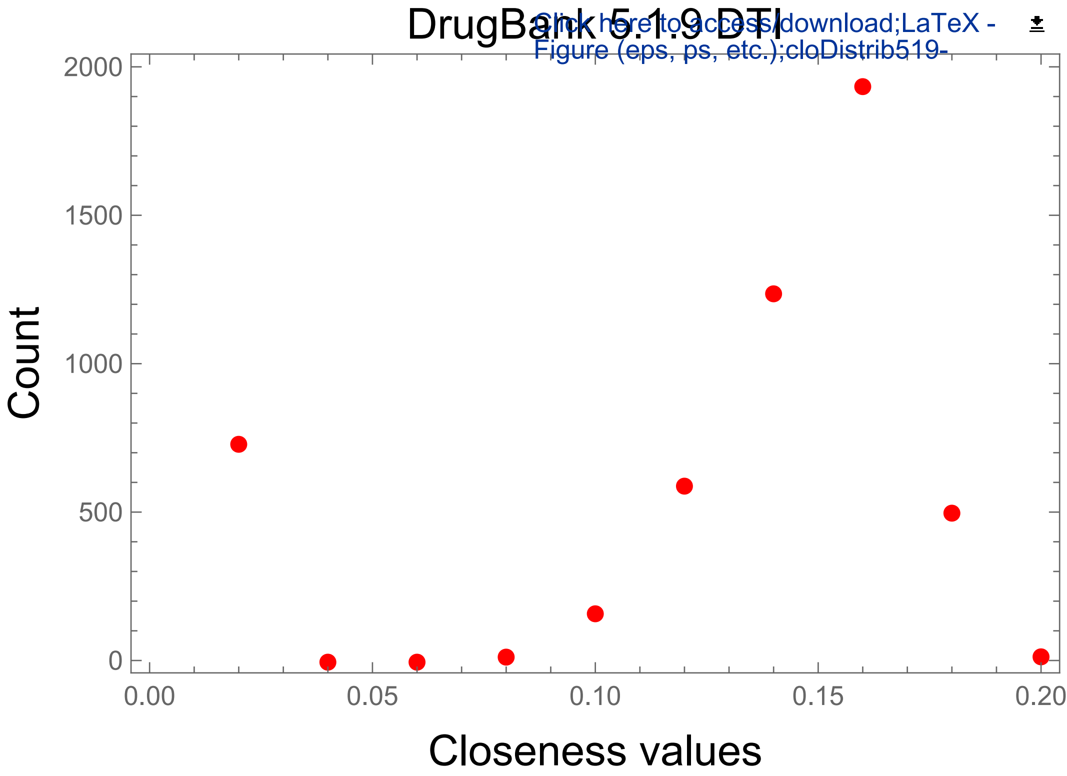

Click here to access/download;LaTeX - Figure (eps, ps, etc.);cloDistrib519-

Click here to access/download;LaTeX - Figure (eps, ps, etc.);cloDistrib519.pdf

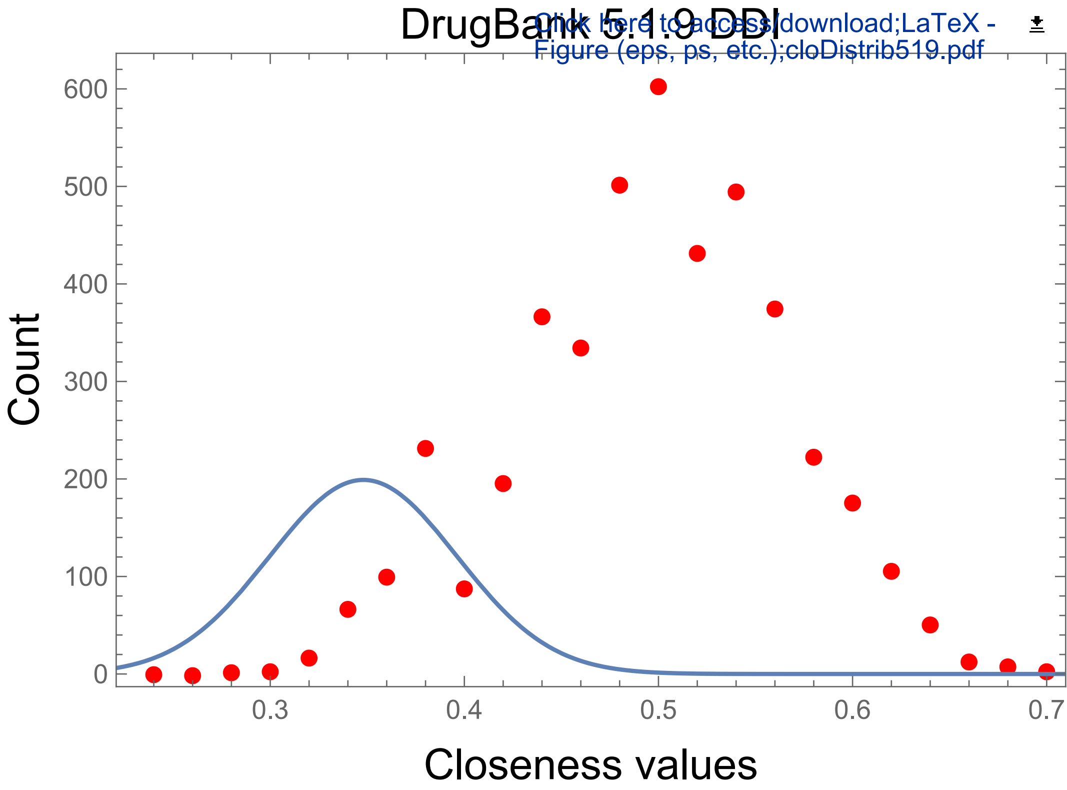

Click here to access/download;LaTeX - Figure (eps, ps, etc.);DB30alphaRU.pdf

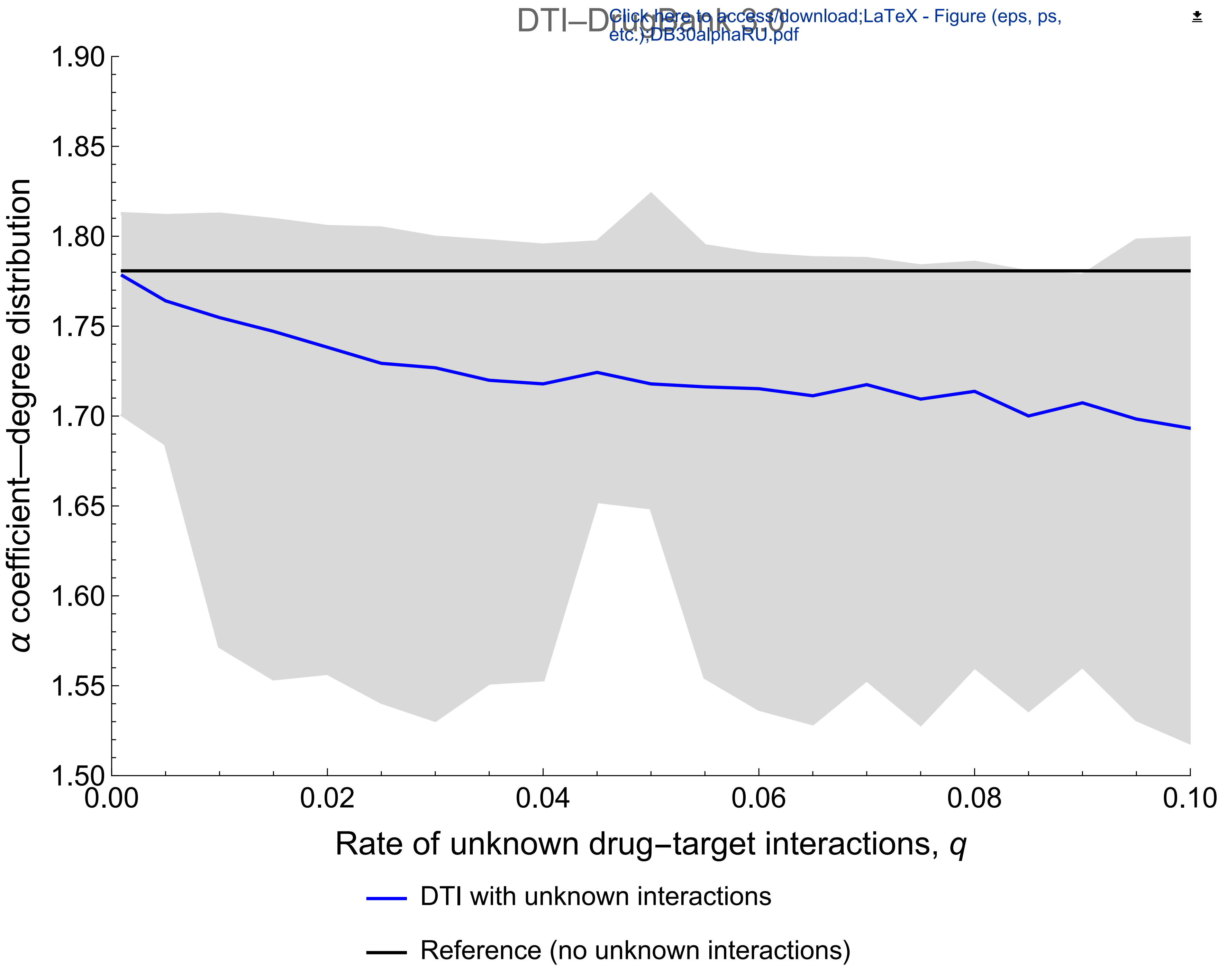

Click here to access/download;LaTeX - Figure (eps, ps, etc.);DB518alphaRU.pdf

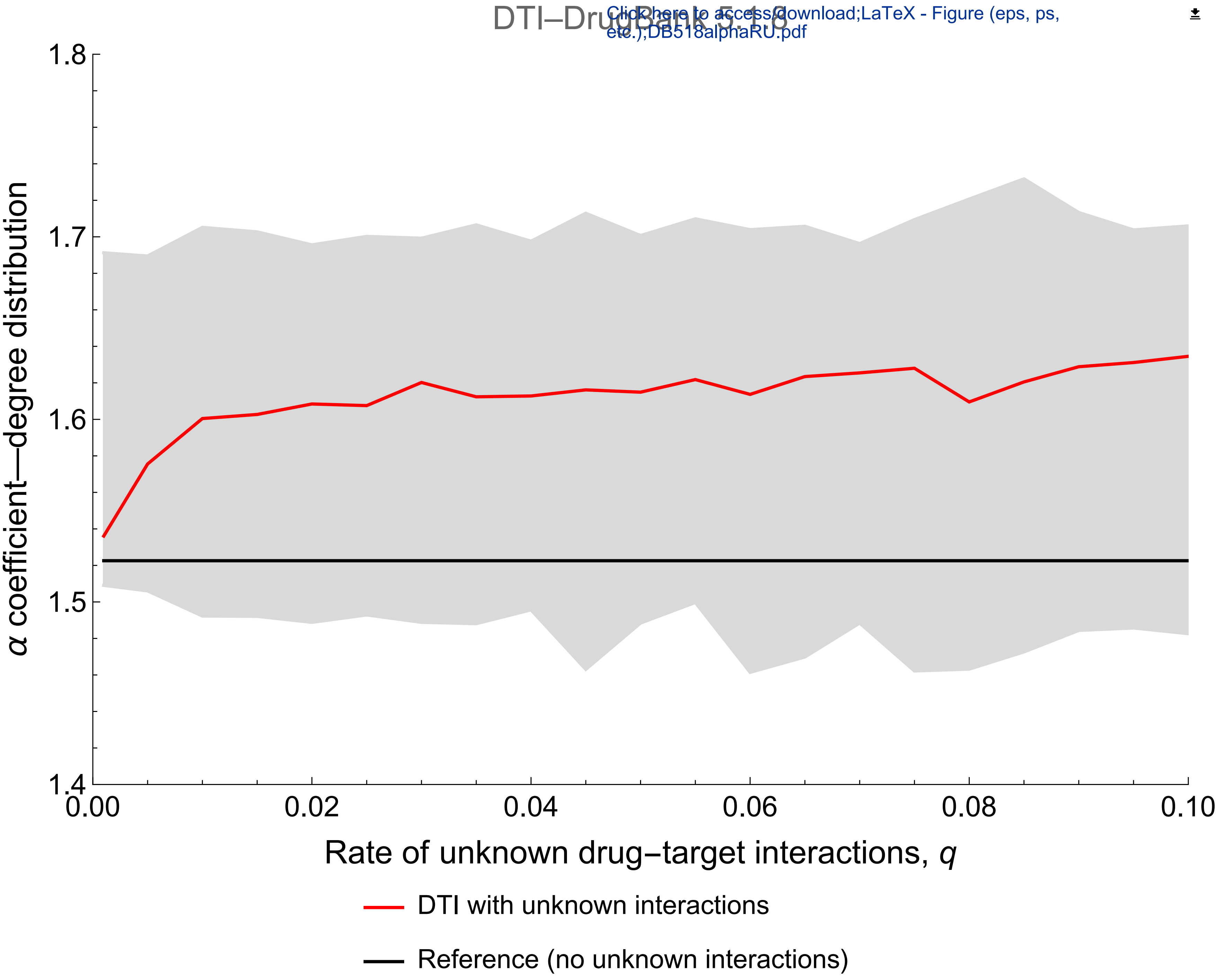

Click here to access/download;LaTeX - Figure (eps, ps, etc.);DDI-evol-deg-1.pdf

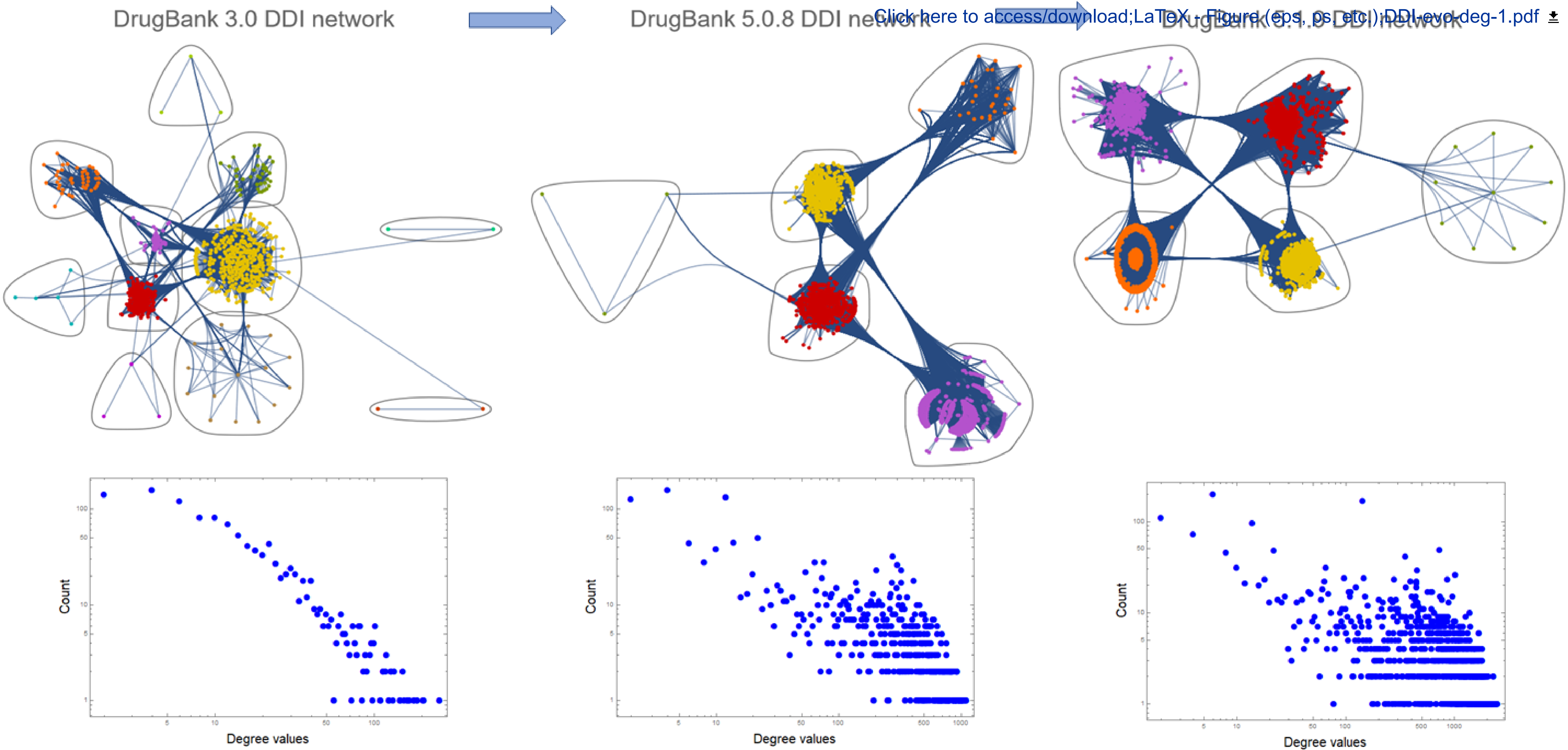

Click here to access/download;LaTeX - Figure (eps, ps, etc.);degBenford.pdf

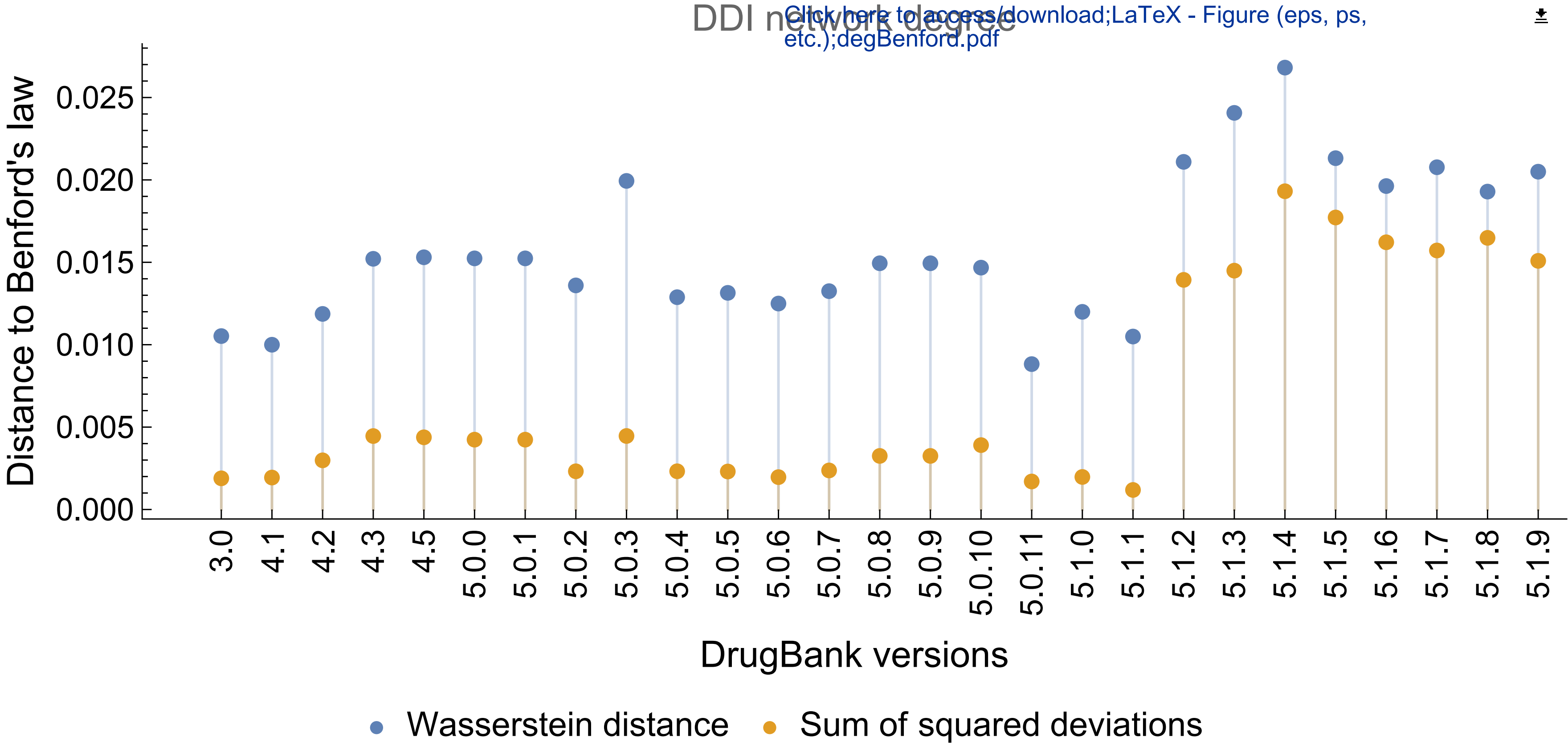

Click here to access/download;LaTeX - Figure (eps, ps, etc.);degBenfordPearson.pdf

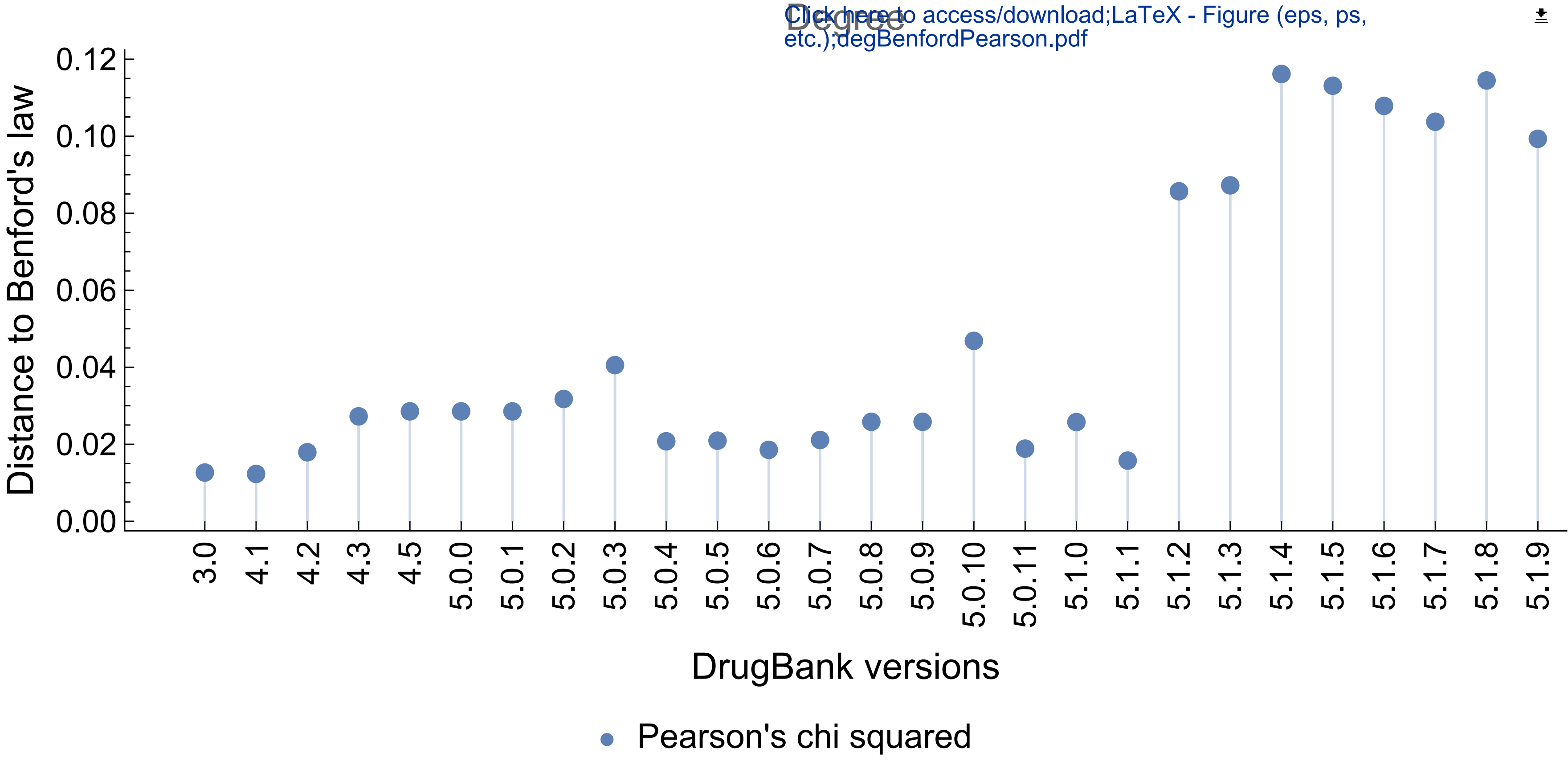

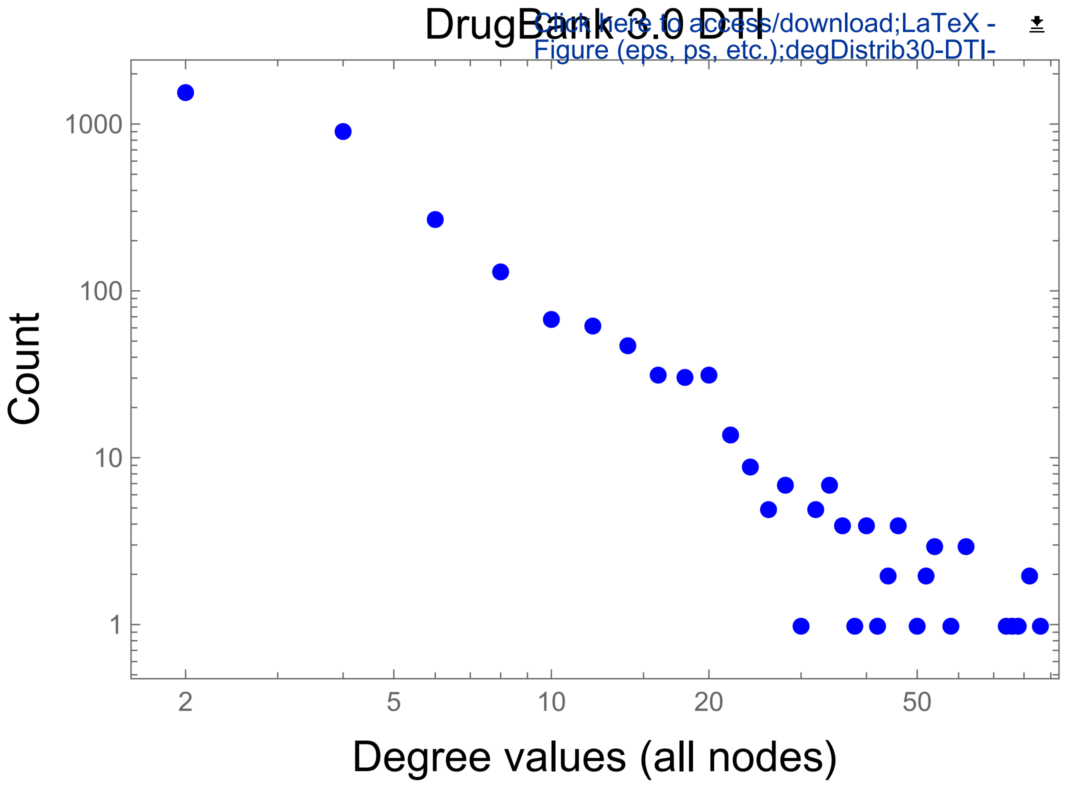

Click here to access/download;LaTeX - Figure (eps, ps, etc.);degDistrib30-DTI-

Click here to access/download;LaTeX - Figure (eps, ps, etc.);degDistrib30-DTI-

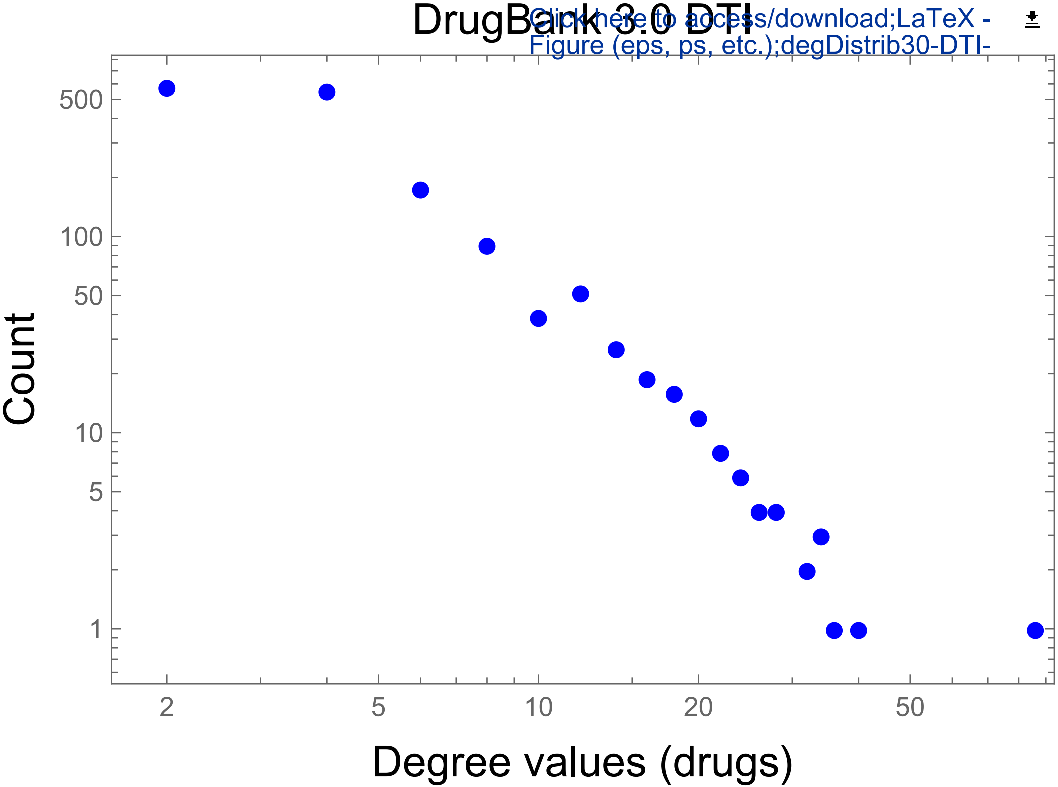

Click here to access/download;LaTeX - Figure (eps, ps, etc.);degDistrib30-DTI-

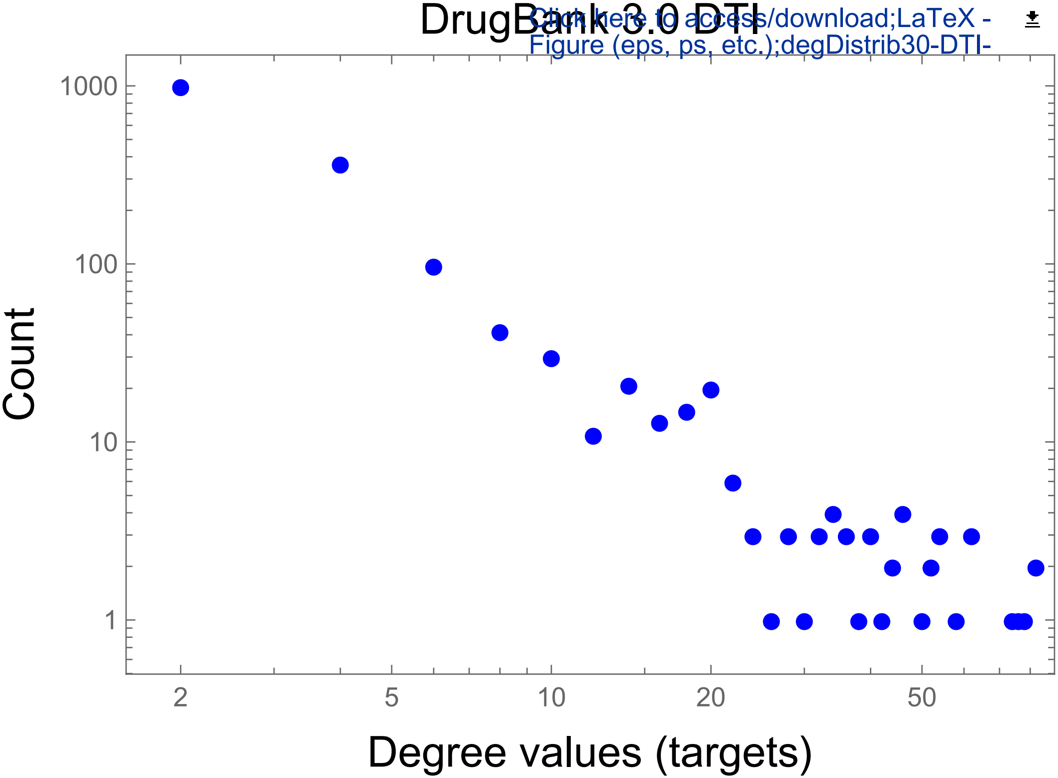

Click here to access/download;LaTeX - Figure (eps, ps, etc.);degDistrib30.pdf

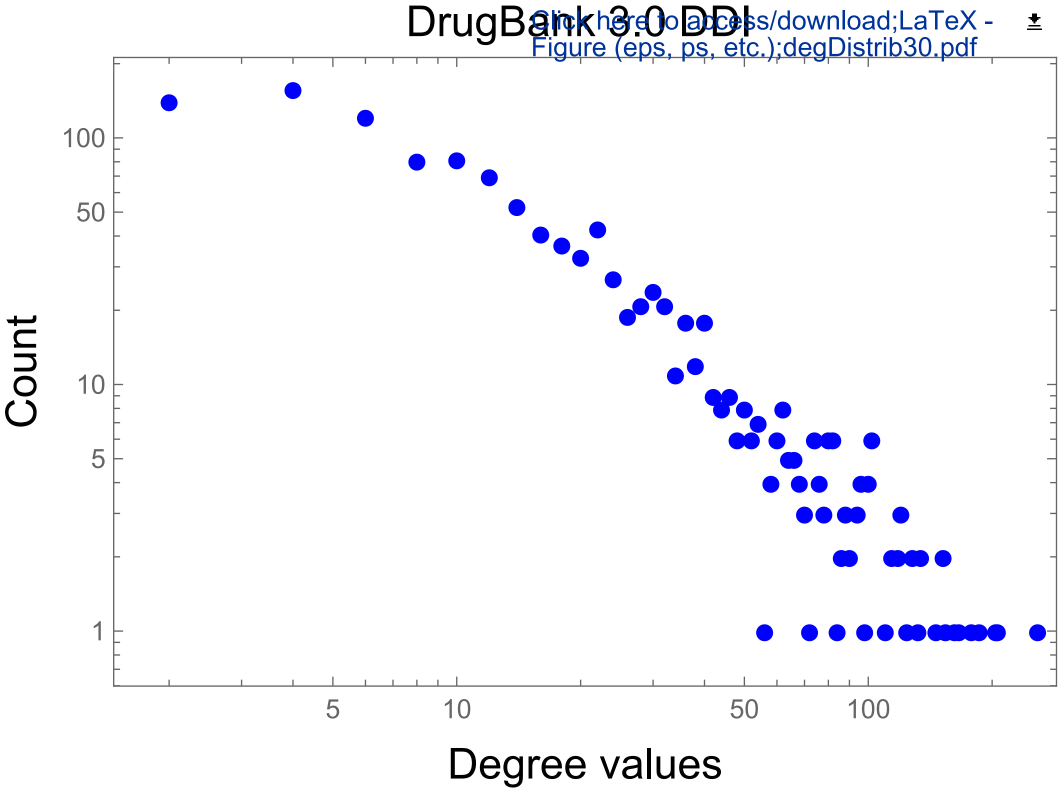

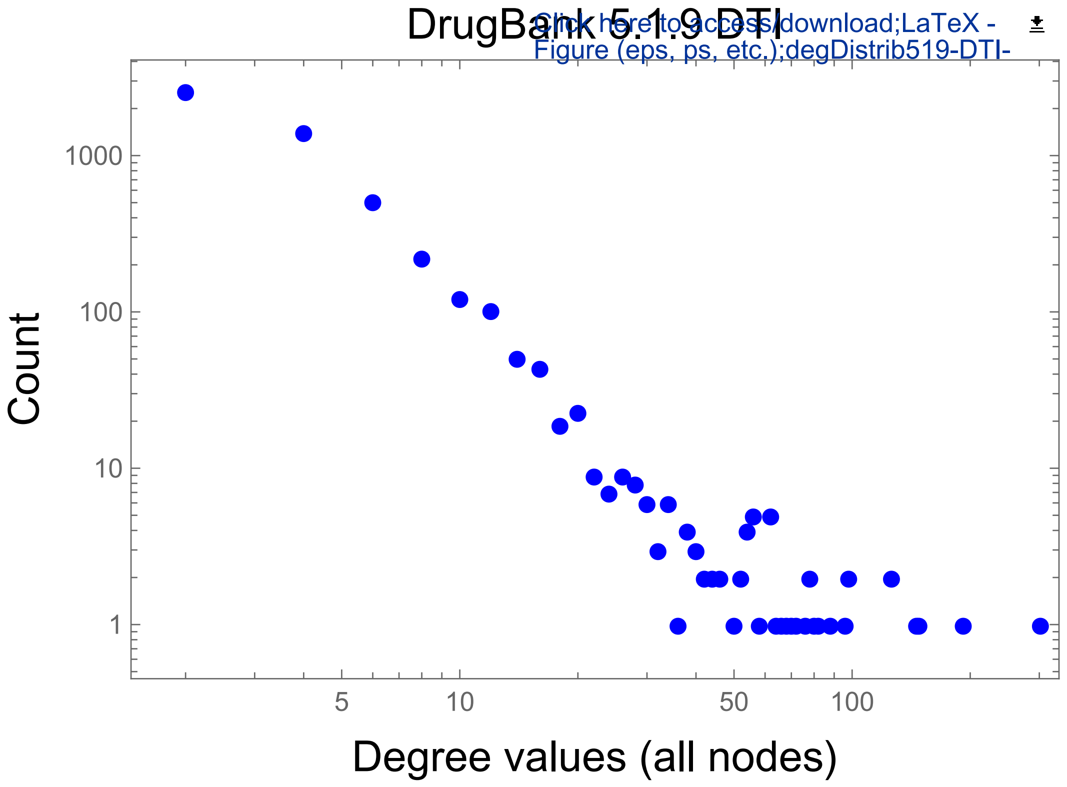
DrugBank 5.1.9 DTI
Click here to access/download;LaTeX - Figure (eps, ps, etc.);degDistrib519-DTI-
Count
1000
100
10
1
5
10
50
100
Degree values (all nodes)

Click here to access/download;LaTeX - Figure (eps, ps, etc.);degDistrib519-DTI-

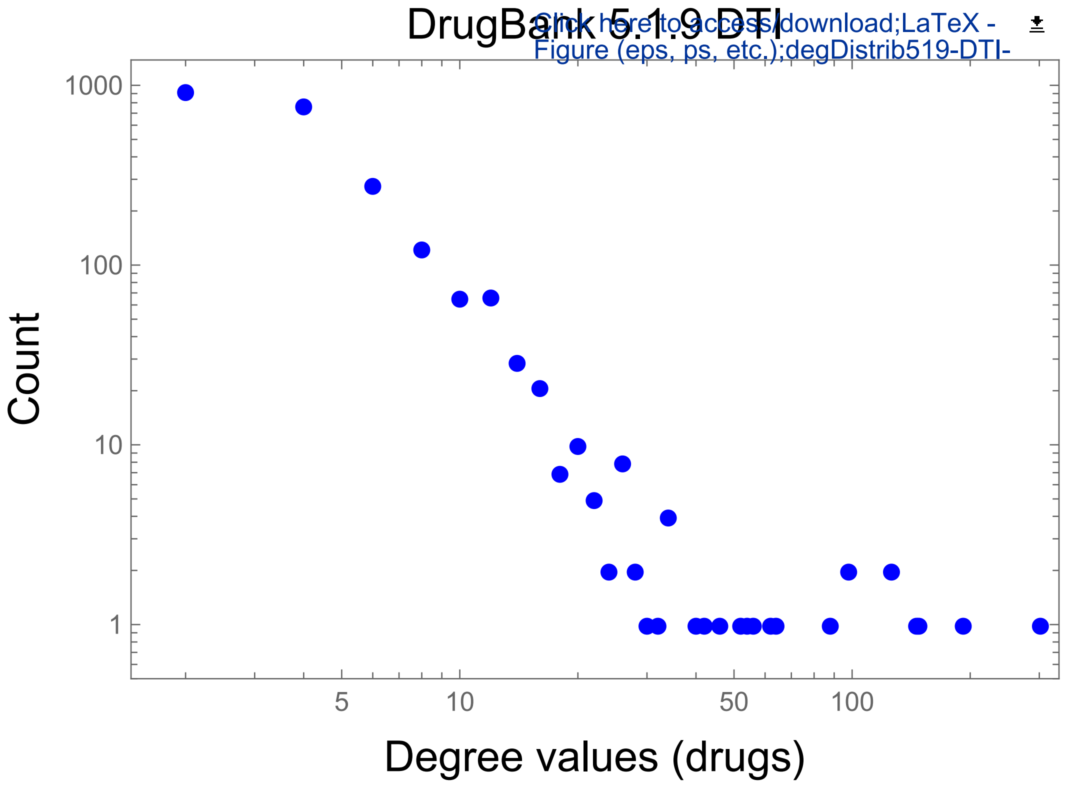

Click here to access/download;LaTeX - Figure (eps, ps, etc.);degDistrib519-DTI-

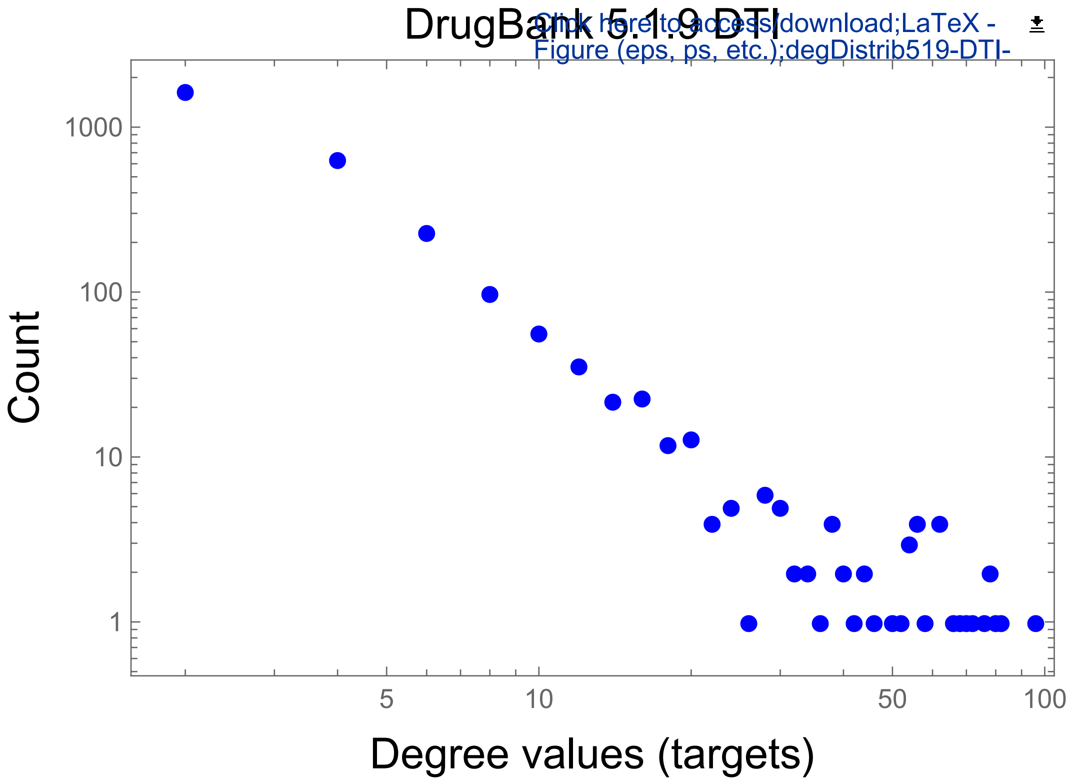

Click here to access/download;LaTeX - Figure (eps, ps, etc.);degDistrib519.pdf

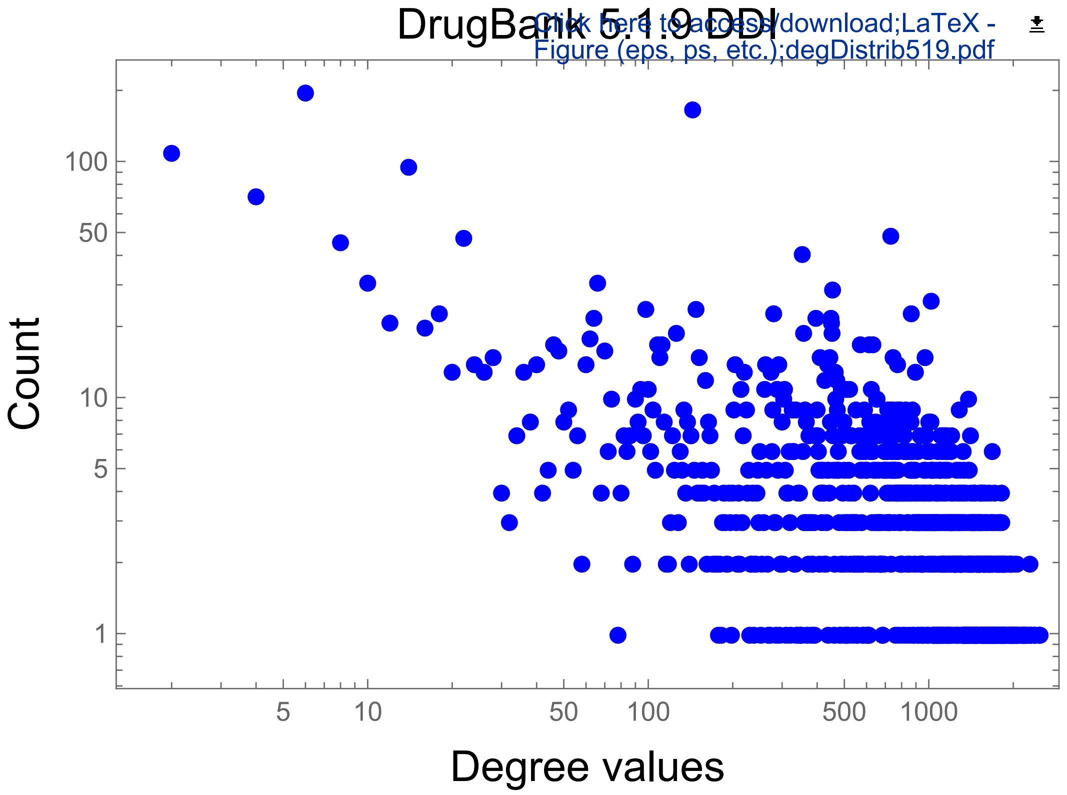

Click here to access/download;LaTeX - Figure (eps, ps, etc.);degree-alpha.pdf

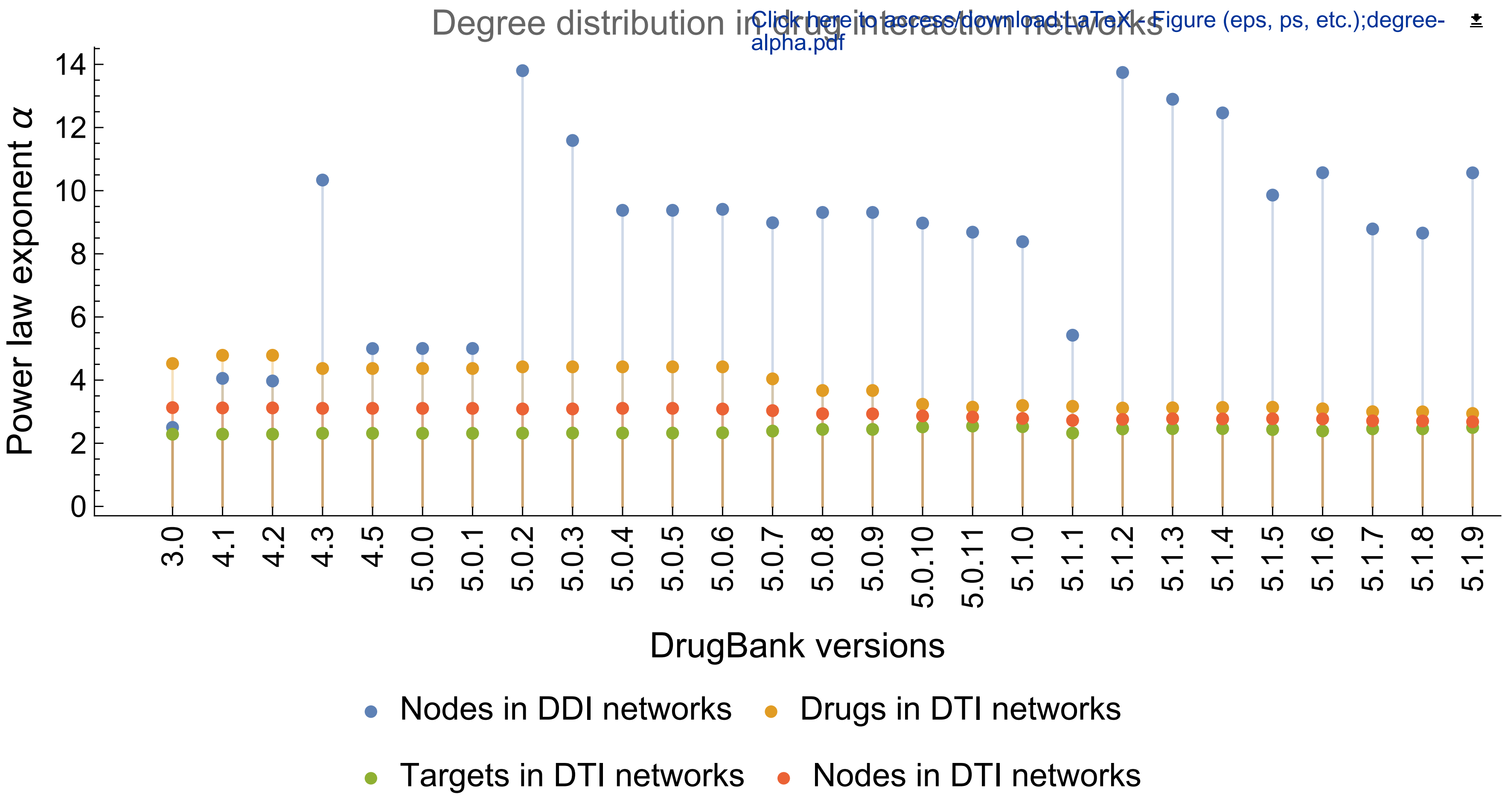

Click here to access/download;LaTeX - Figure (eps, ps, etc.);dens-ddi.pdf

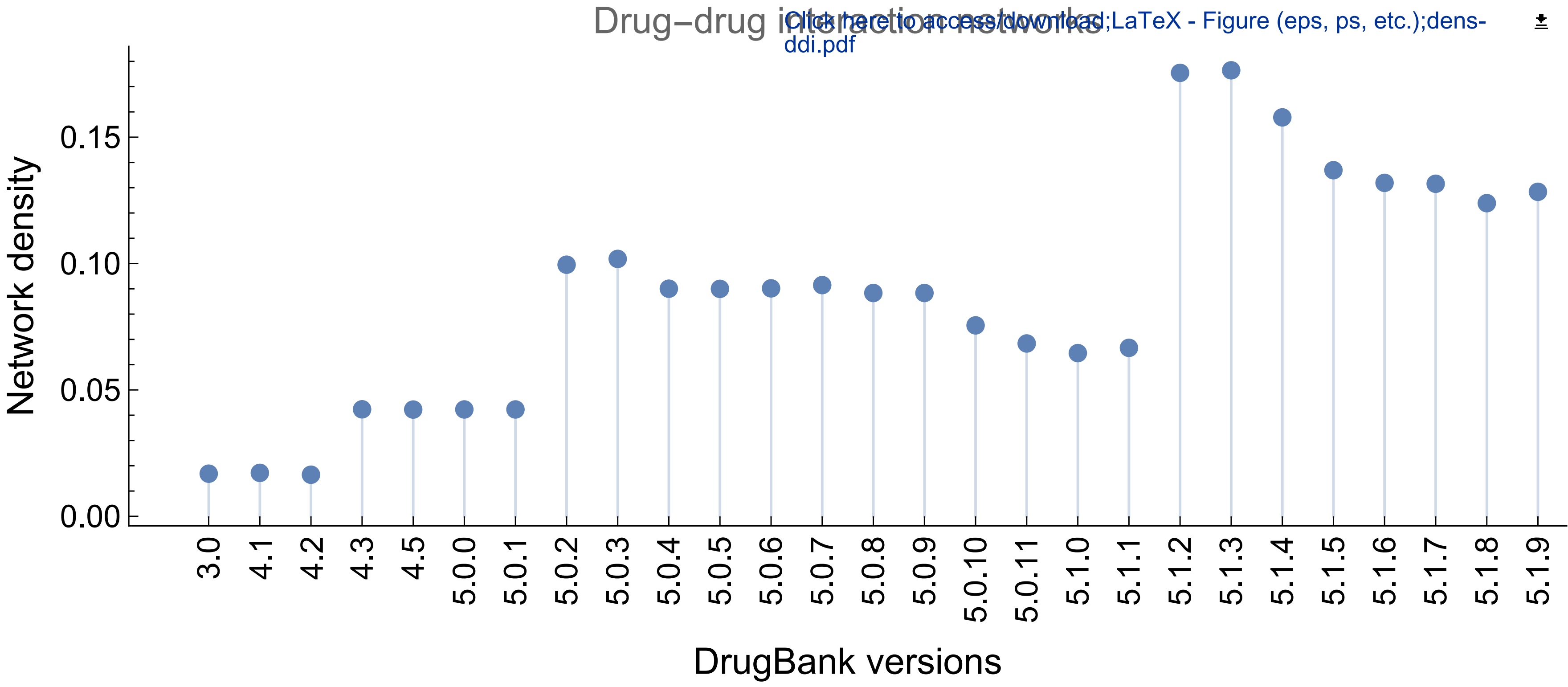

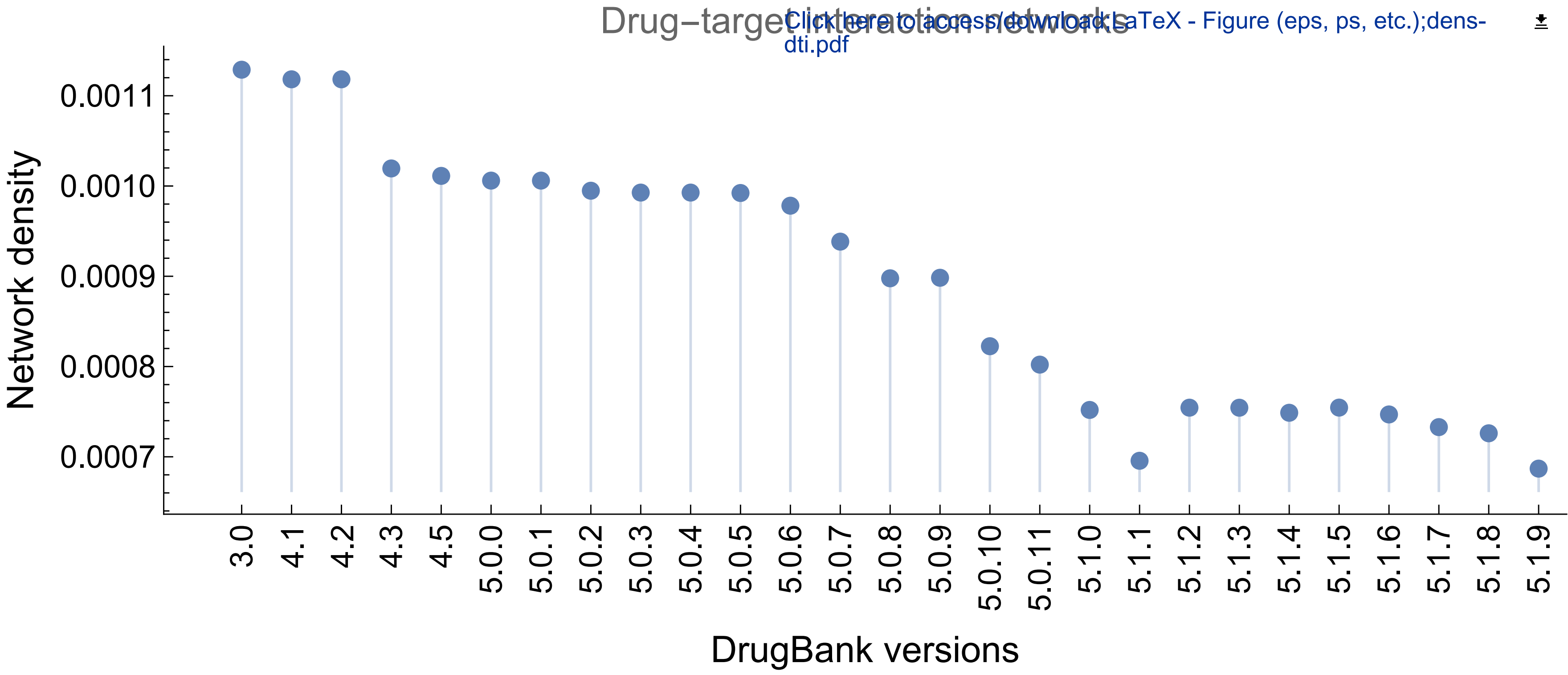

Click here to access/download;LaTeX - Figure (eps, ps, etc.);dens-dti.pdf

Click here to access/download;LaTeX - Figure (eps, ps, etc.);dia.pdf

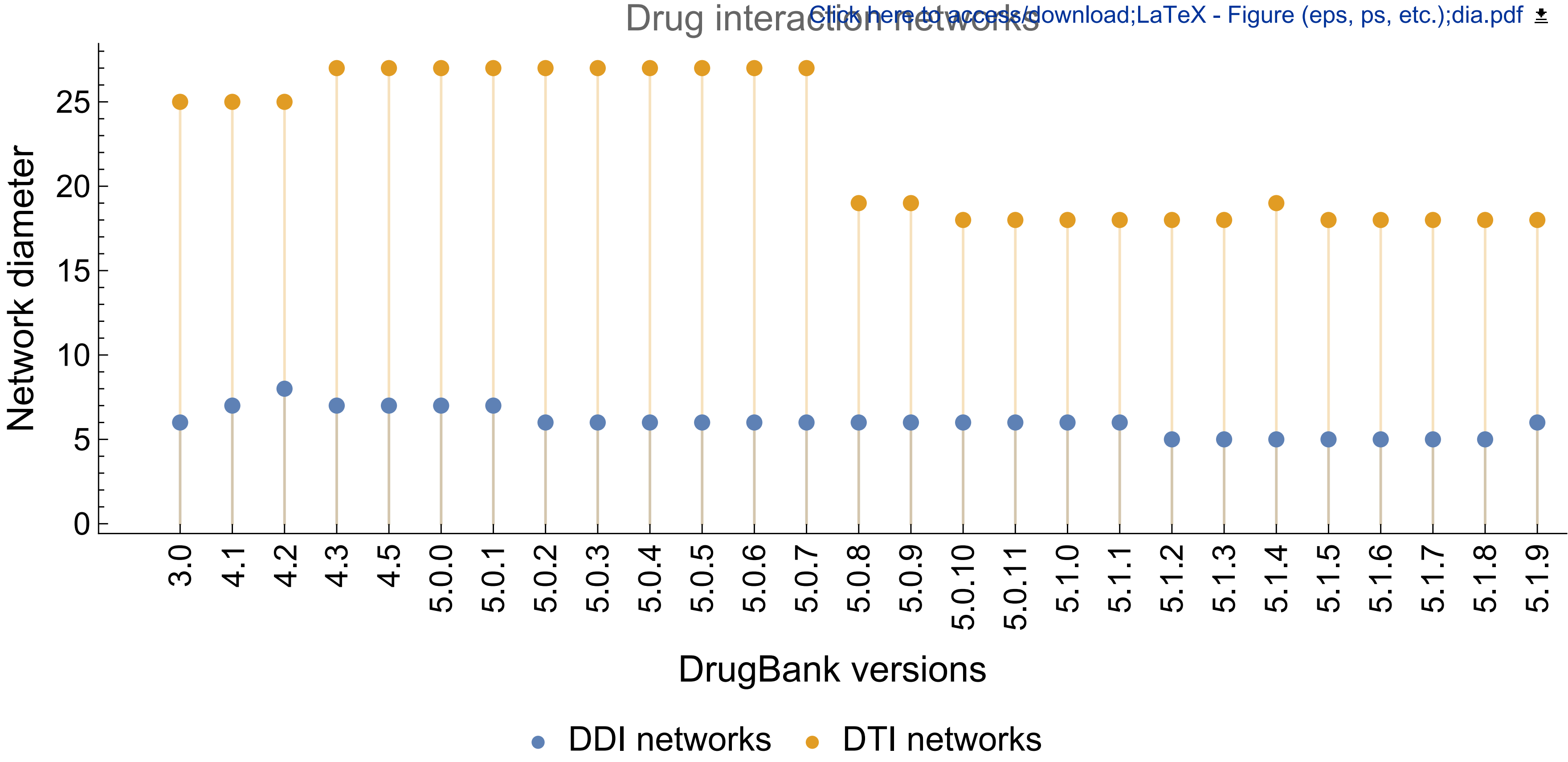

Click here to access/download;LaTeX - Figure (eps, ps, etc.);dti-benford-30-bet.pdf

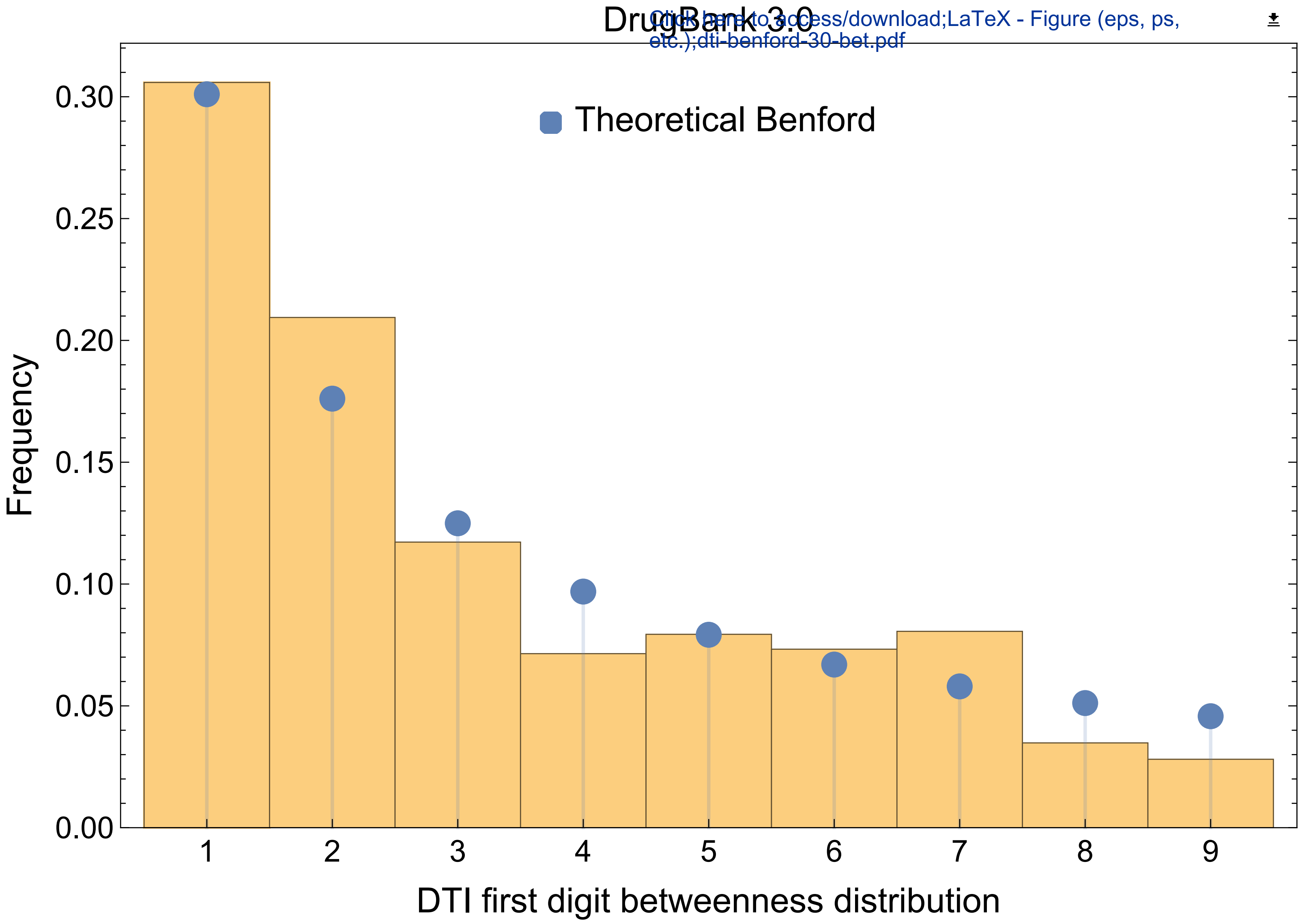

Click here to access/download;LaTeX - Figure (eps, ps, etc.);dti-benford-30-degree-a.pdf

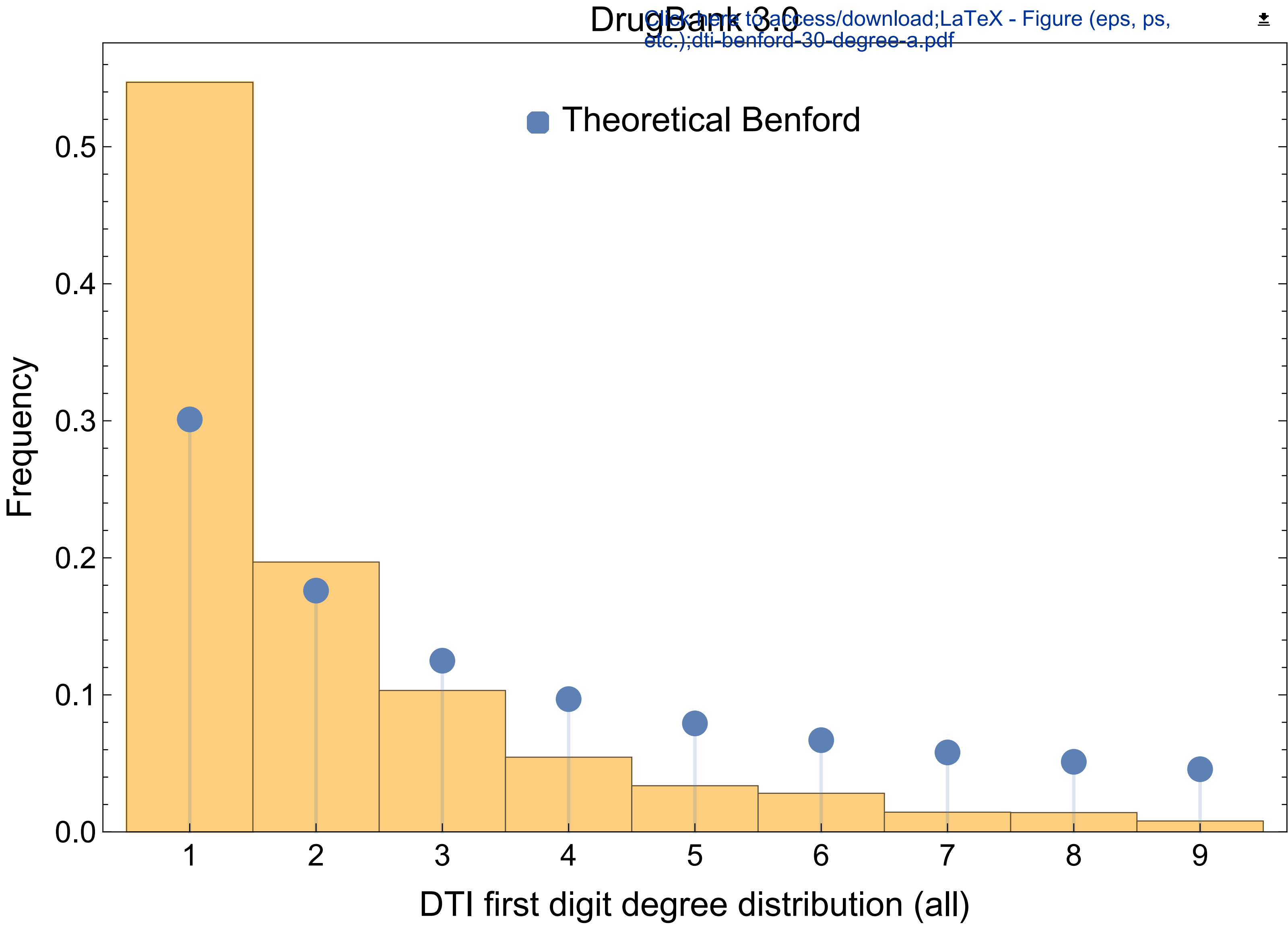

Click here to access/download;LaTeX - Figure (eps, ps, etc.);dti-benford-30-degree-d.pdf

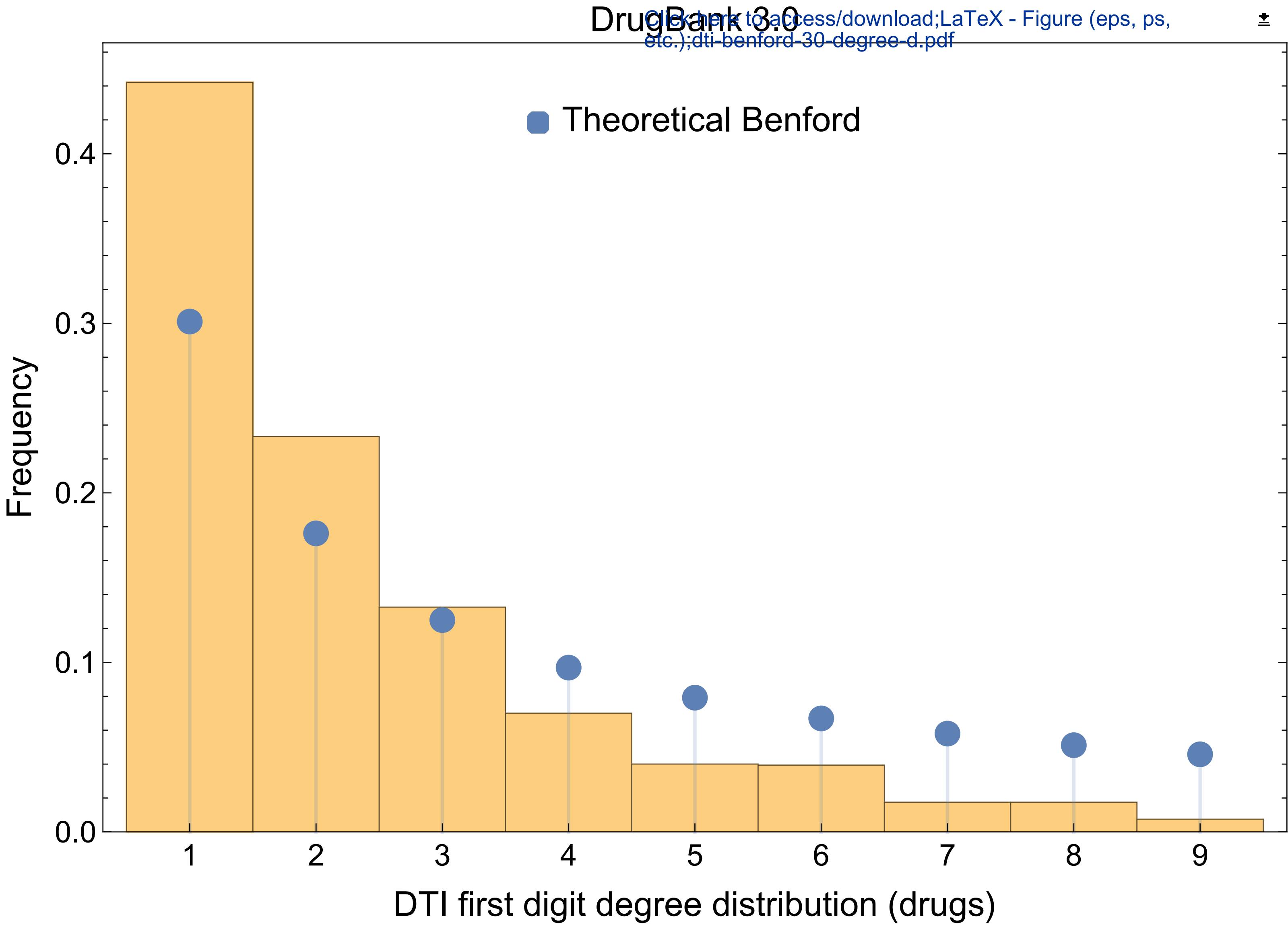

Click here to access/download;LaTeX - Figure (eps, ps, etc.);dti-benford-30-degree-t.pdf

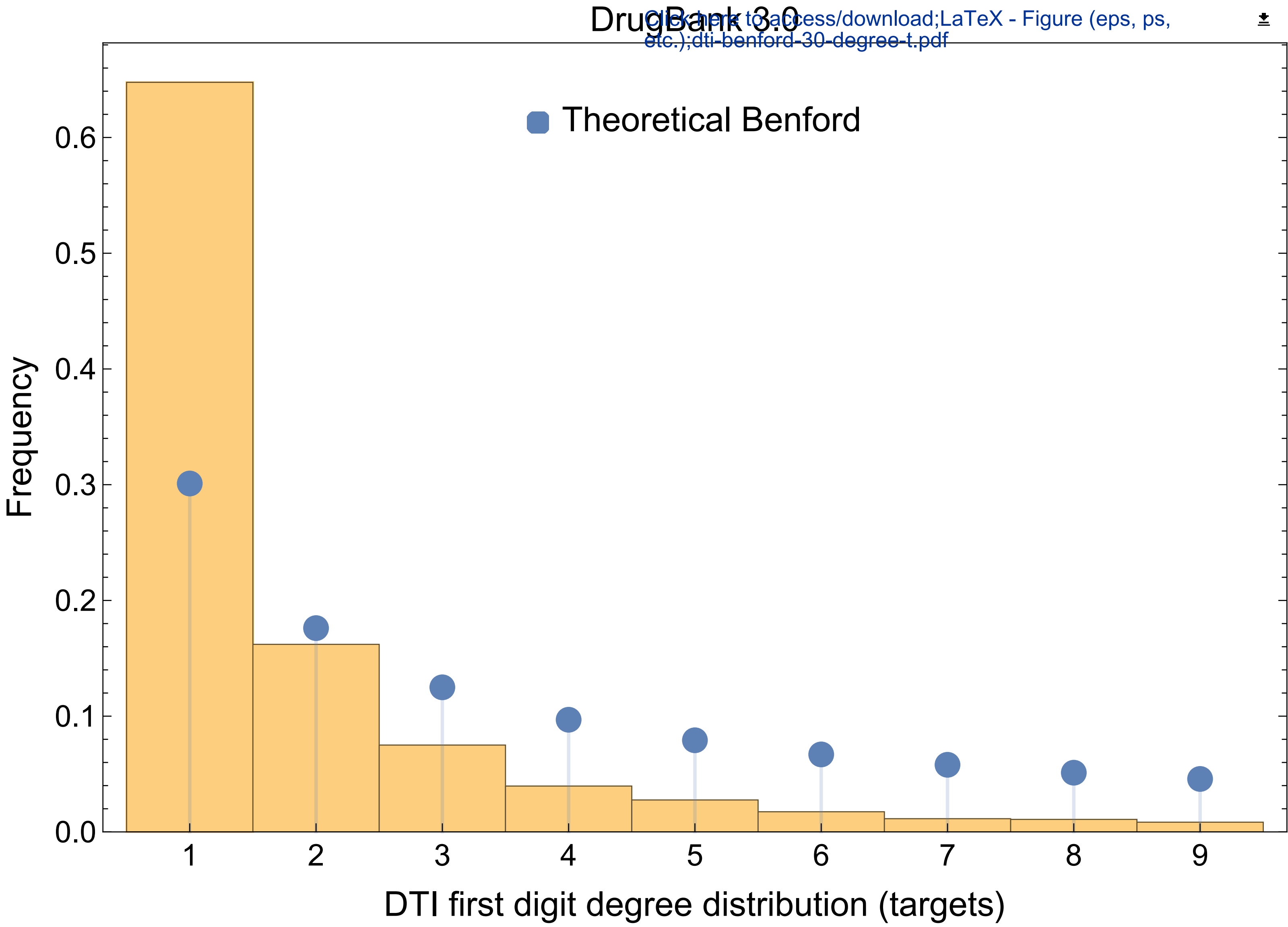

Click here to access/download;LaTeX - Figure (eps, ps, etc.);dti-benford-519-bet.pdf

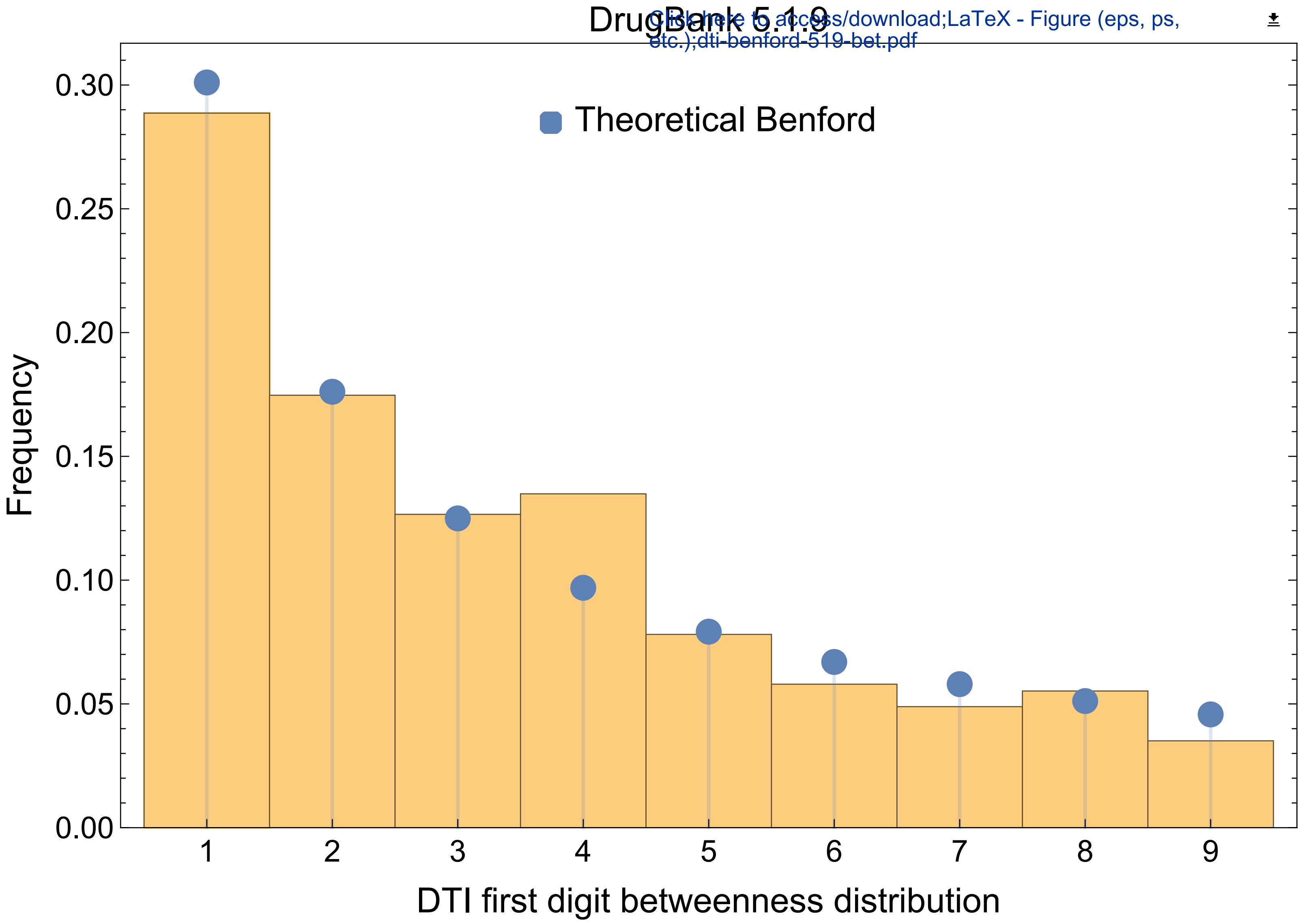

Click here to access/download;LaTeX - Figure (eps, ps, etc.);dti-benford-519-degree-a.pdf

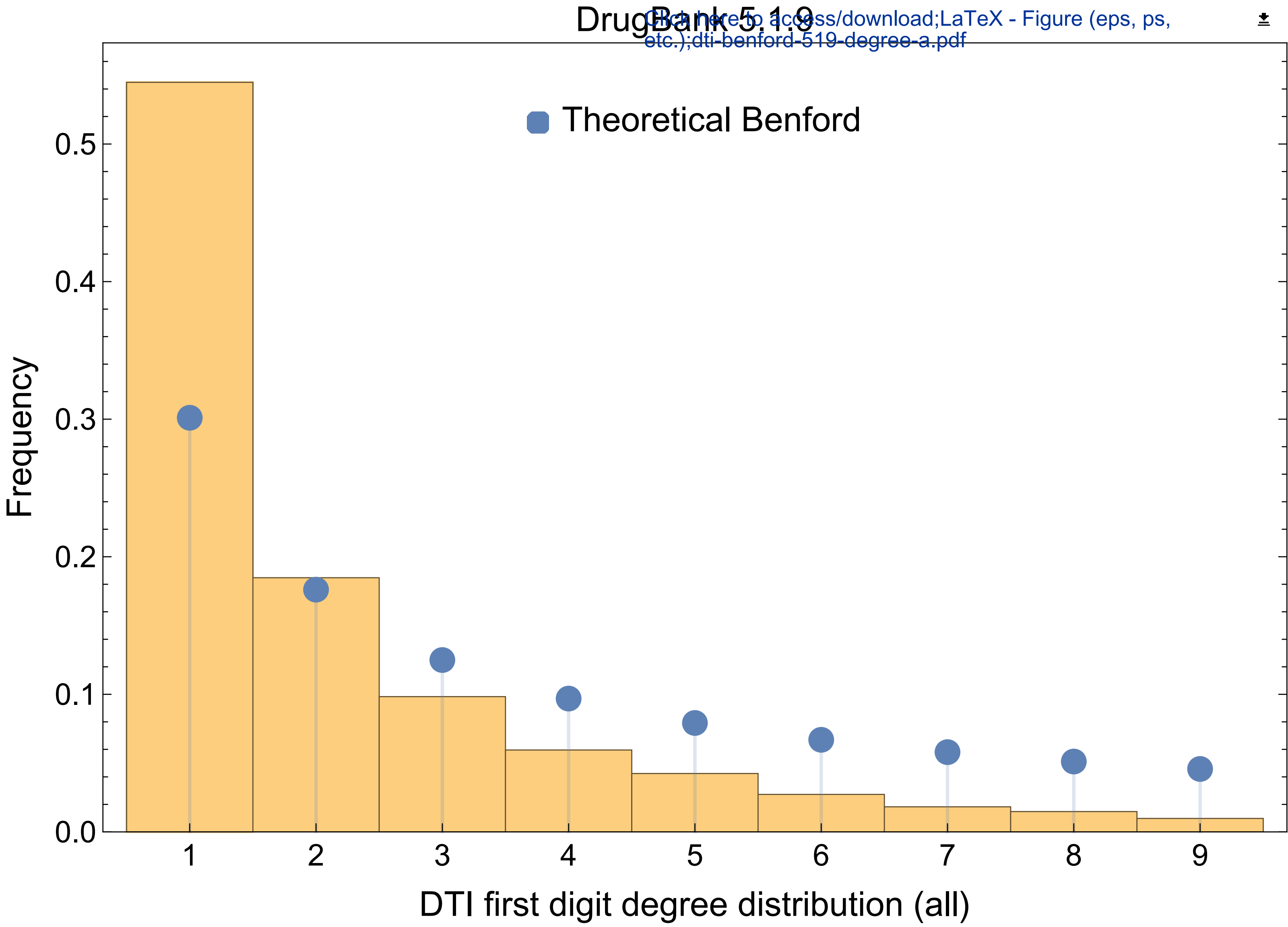

Click here to access/download;LaTeX - Figure (eps, ps, etc.);dti-benford-519-degree-d.pdf

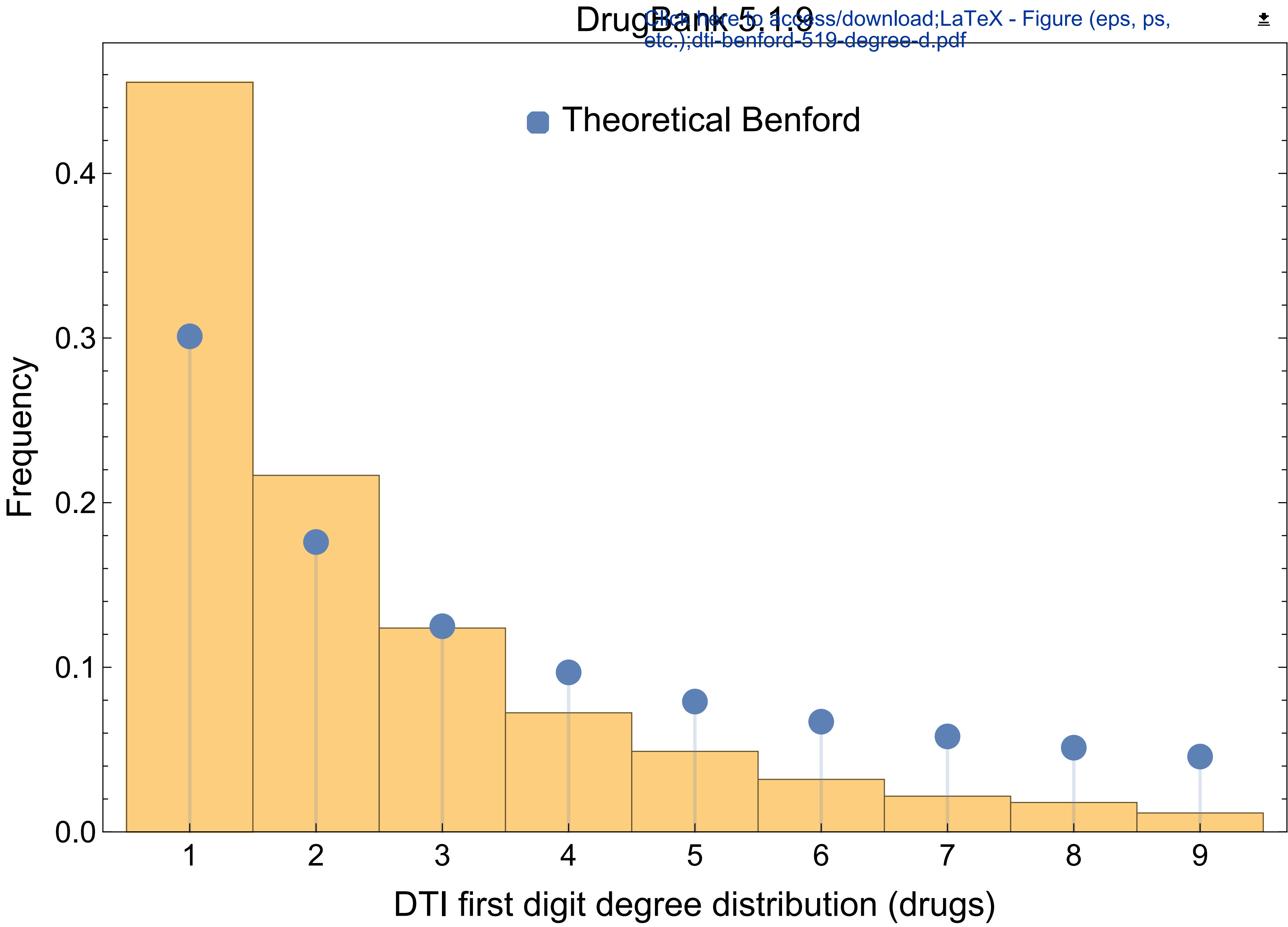

Click here to access/download;LaTeX - Figure (eps, ps, etc.);dti-benford-519-degree-t.pdf

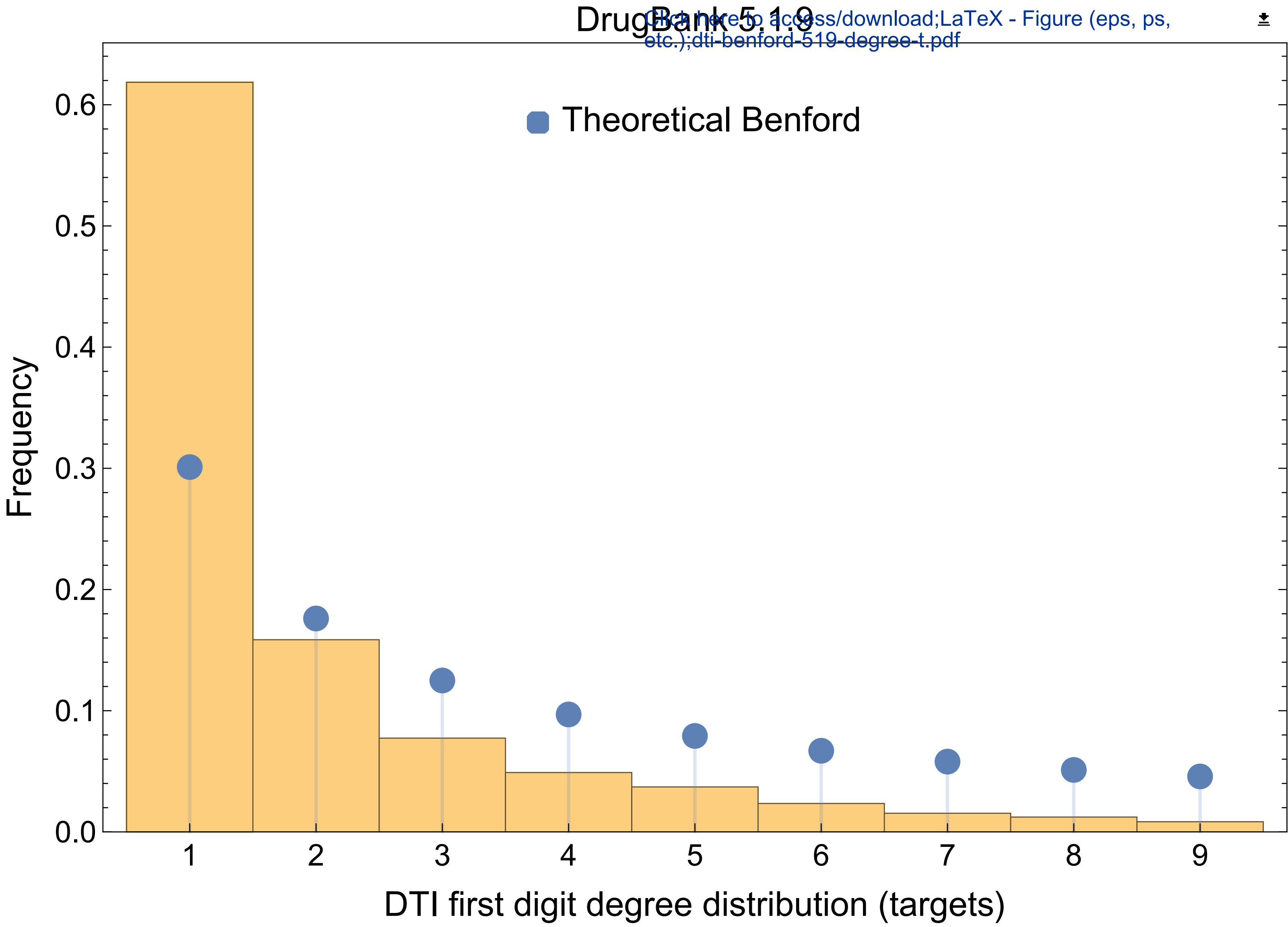

Click here to access/download;LaTeX - Figure (eps, ps, etc.);DTI-evo-new.pdf

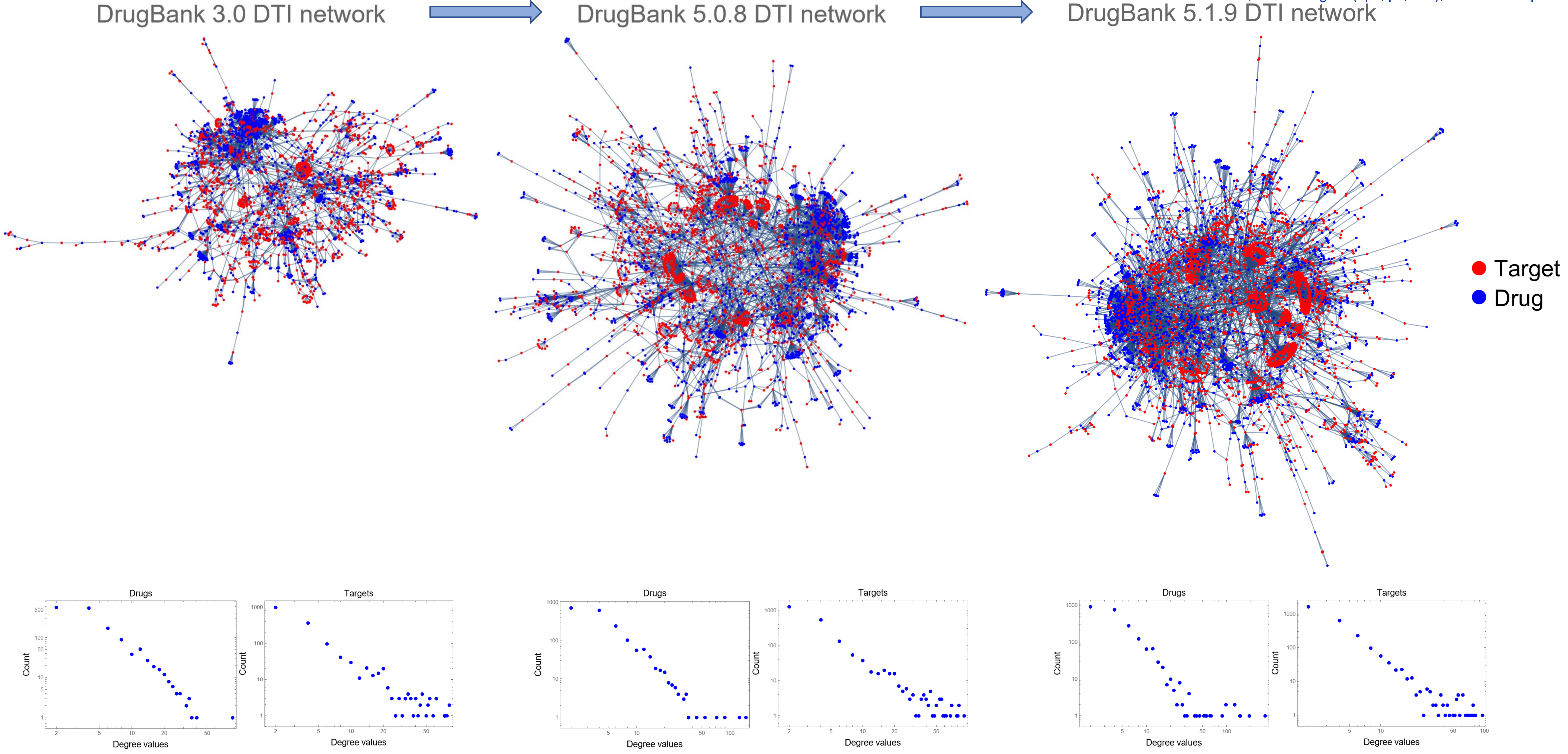

Click here to access/download;LaTeX - Figure (eps, ps, etc.);dti-qq-benford-30-bet.pdf

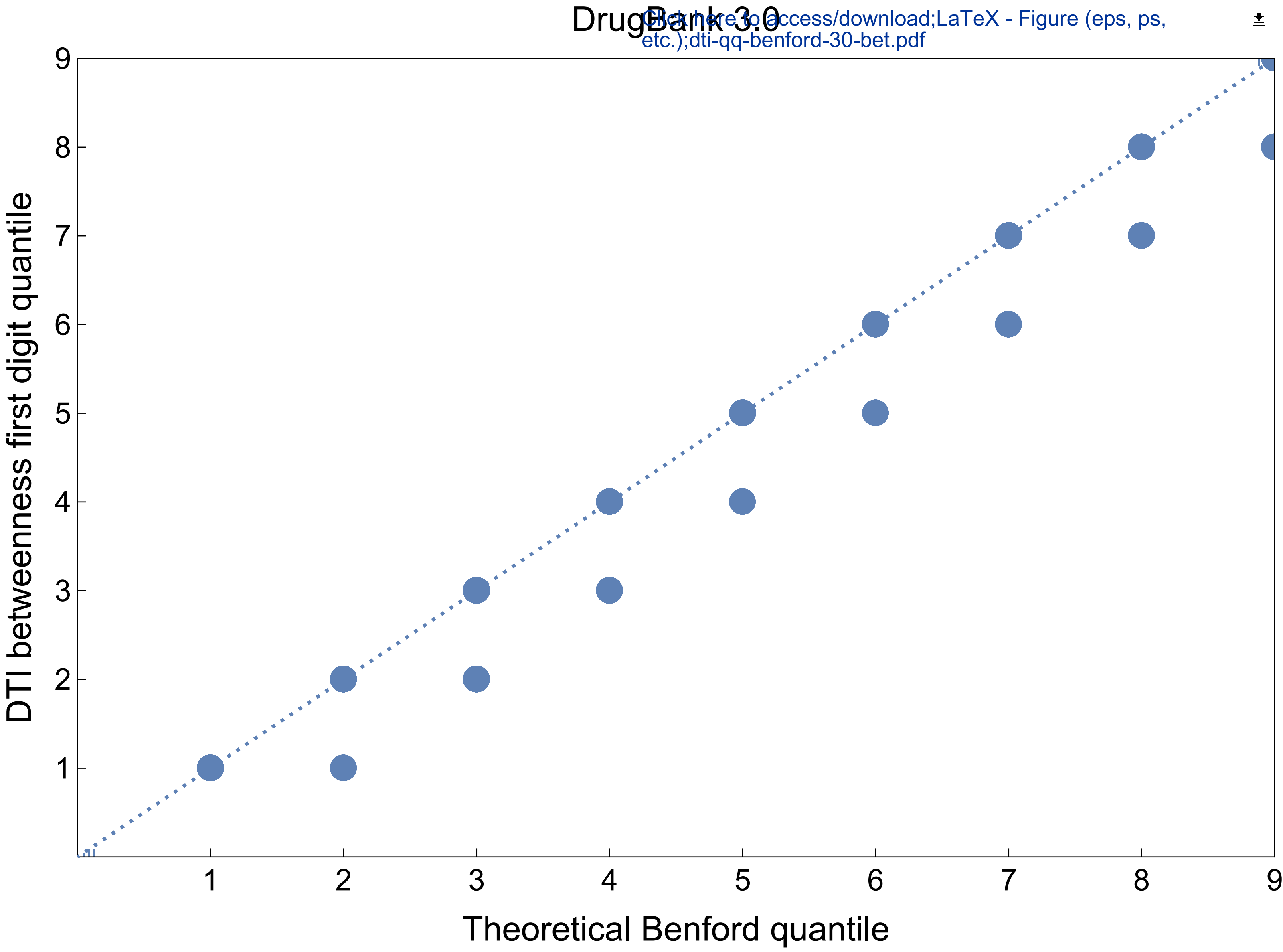

Click here to access/download;LaTeX - Figure (eps, ps, etc.);dti-qq-benford-30-degree-a.pdf

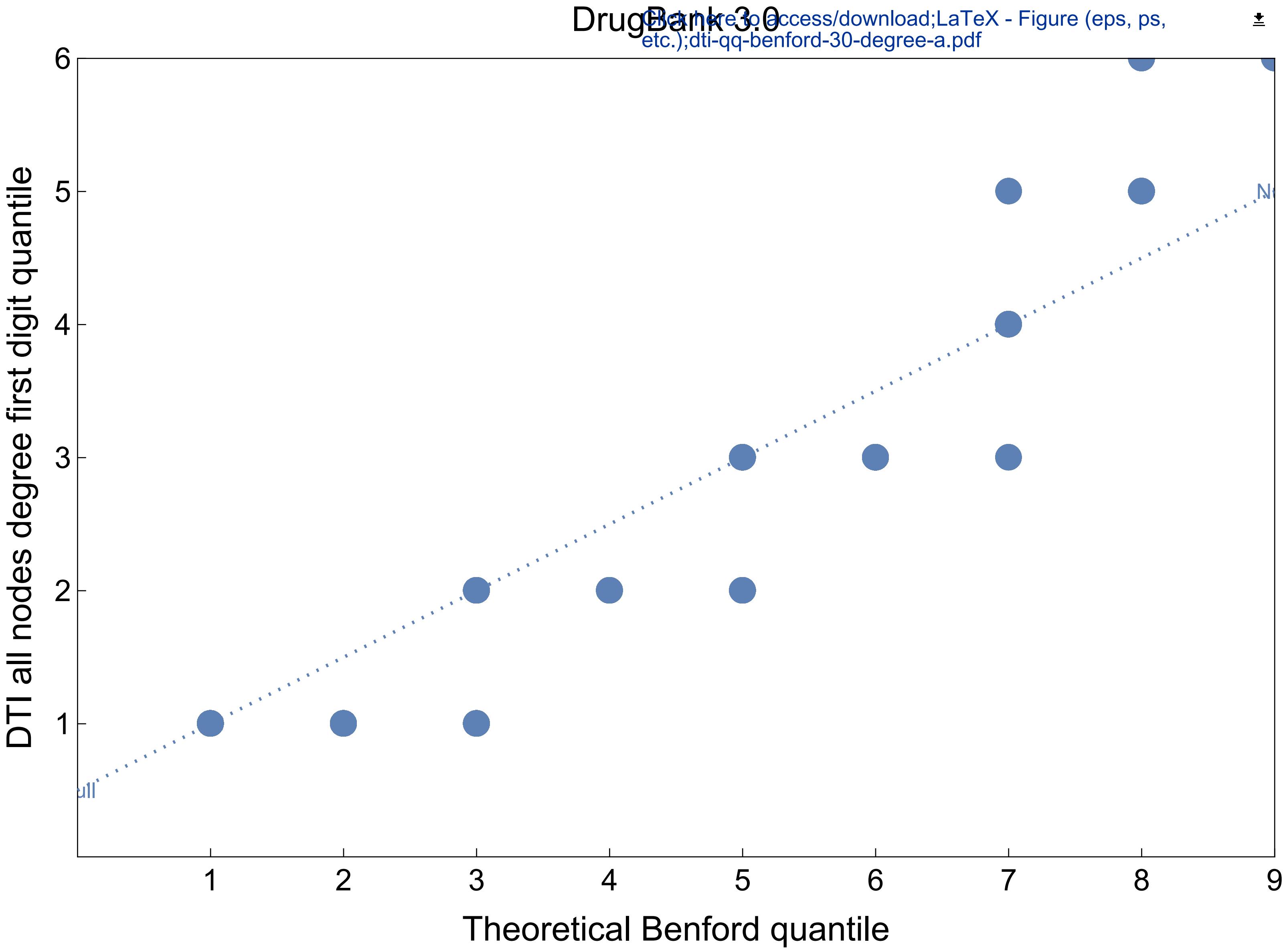

Click here to access/download;LaTeX - Figure (eps, ps, etc.);dti-qq-benford-30-degree-d.pdf

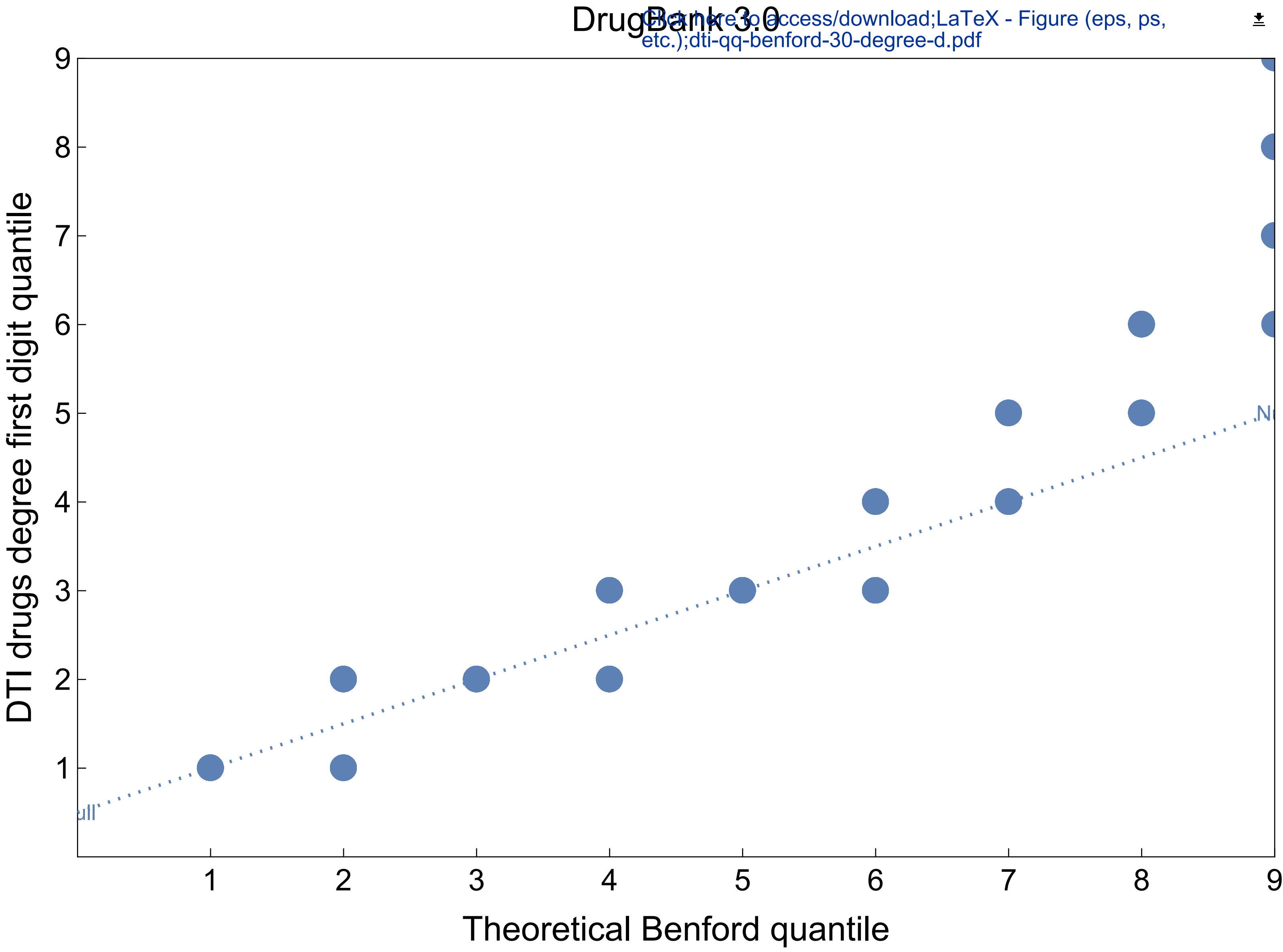

Click here to access/download;LaTeX - Figure (eps, ps, etc.);dti-qq-benford-30-degree-t.pdf

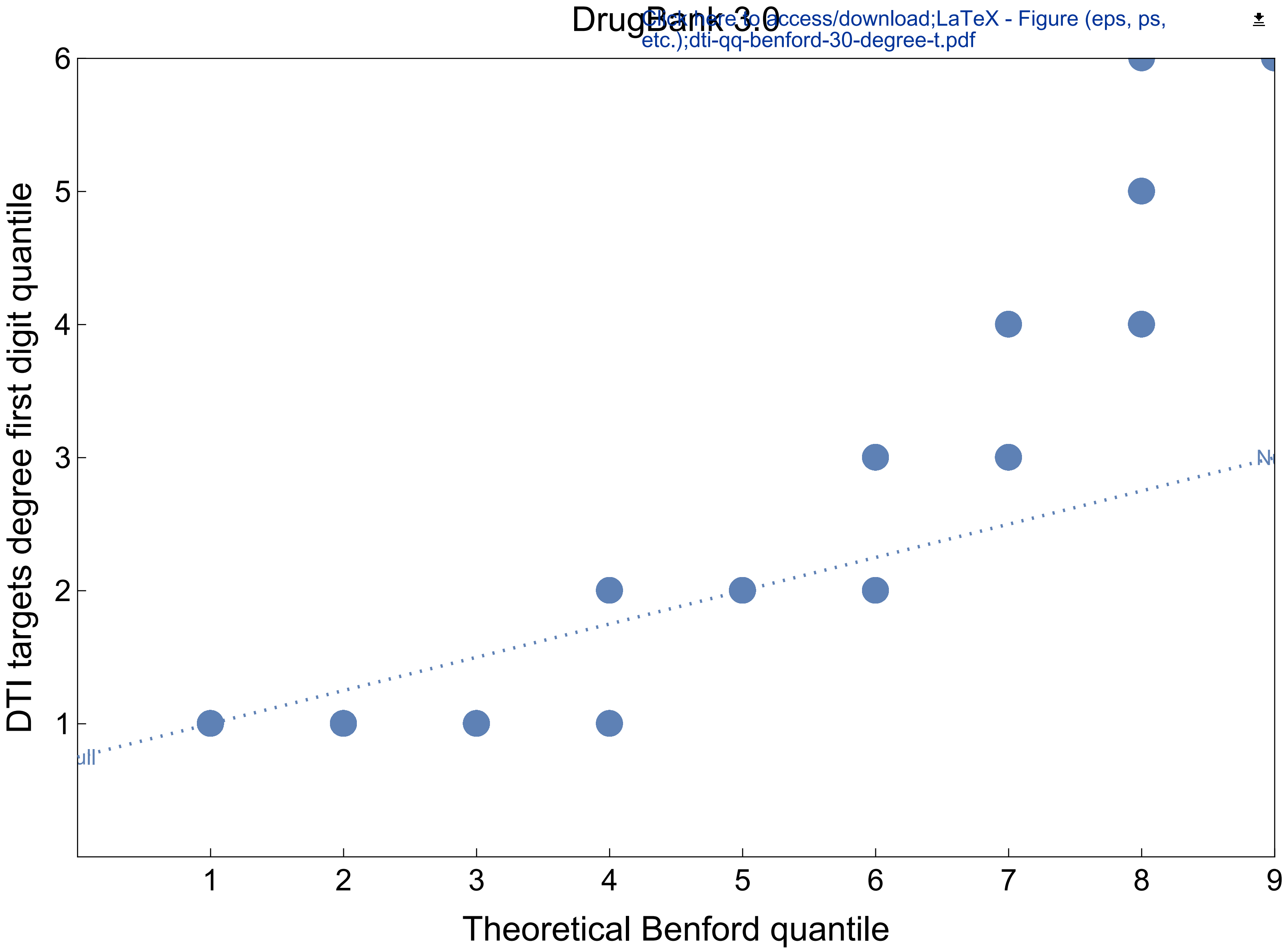

Click here to access/download;LaTeX - Figure (eps, ps, etc.);dti-qq-benford-519-bet.pdf

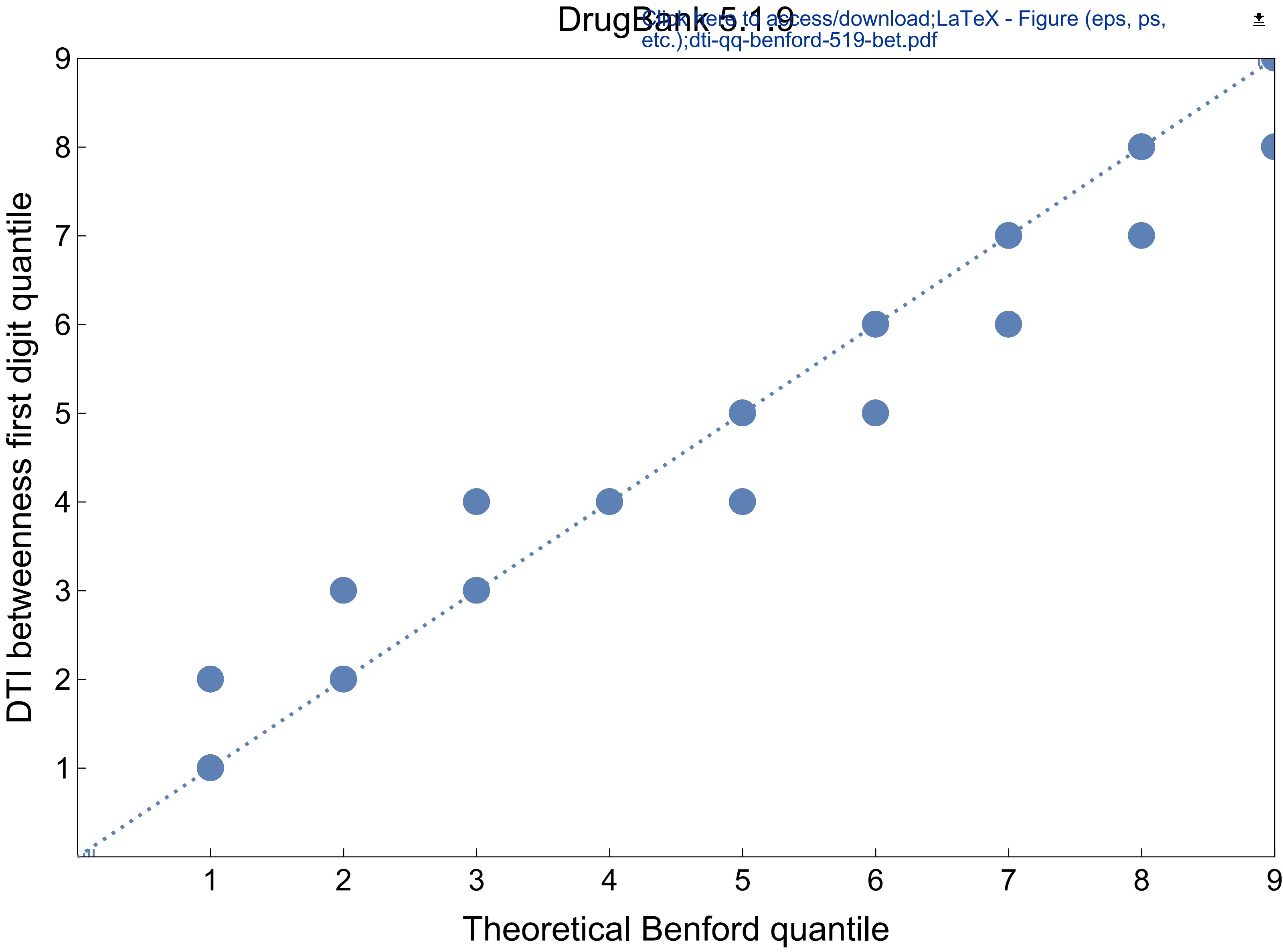

Click here to access/download;LaTeX - Figure (eps, ps, etc.);dti-qq-benford-519-degree-a.pdf

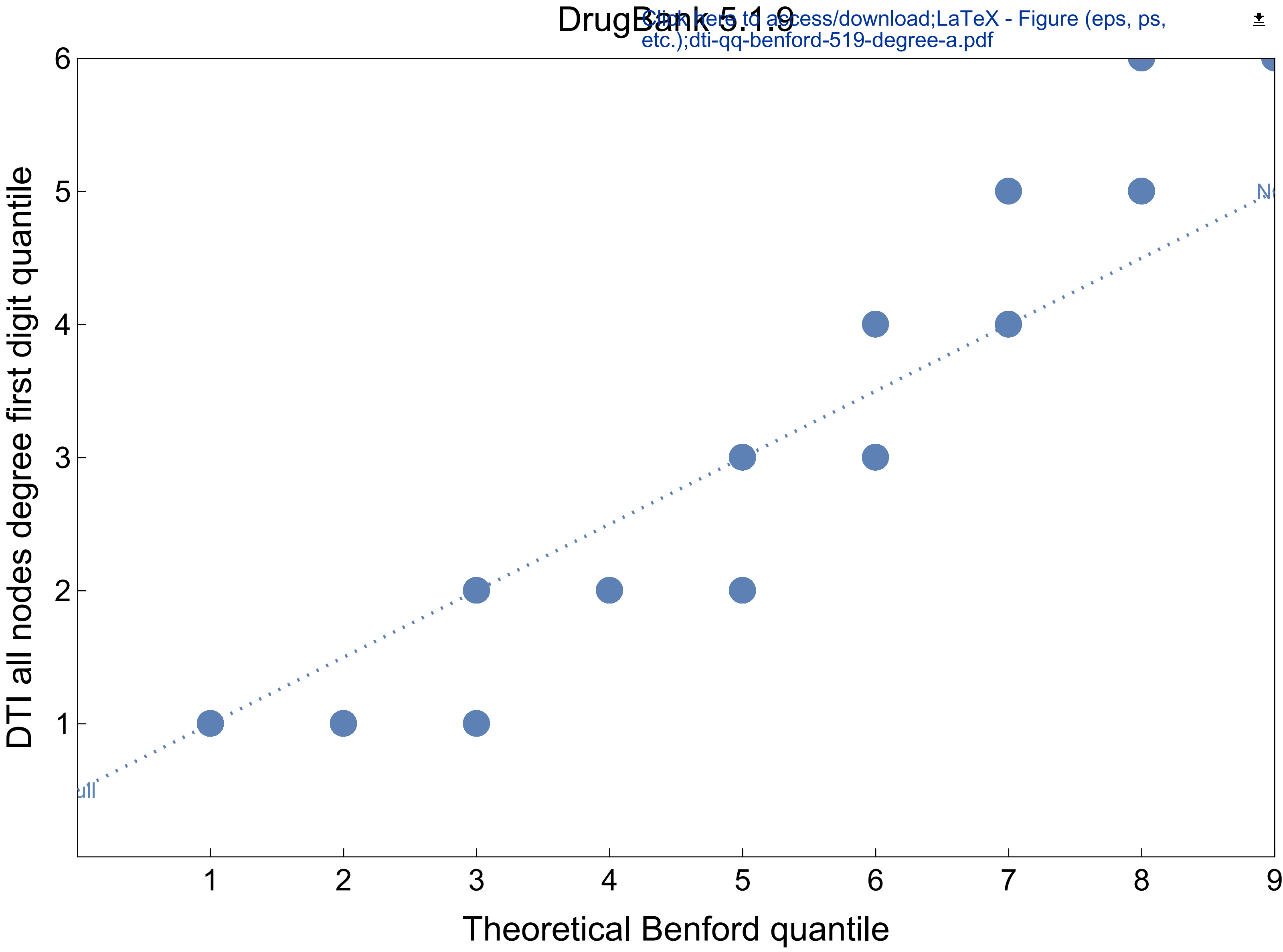

Click here to access/download;LaTeX - Figure (eps, ps, etc.);dti-qq-benford-519-degree-d.pdf

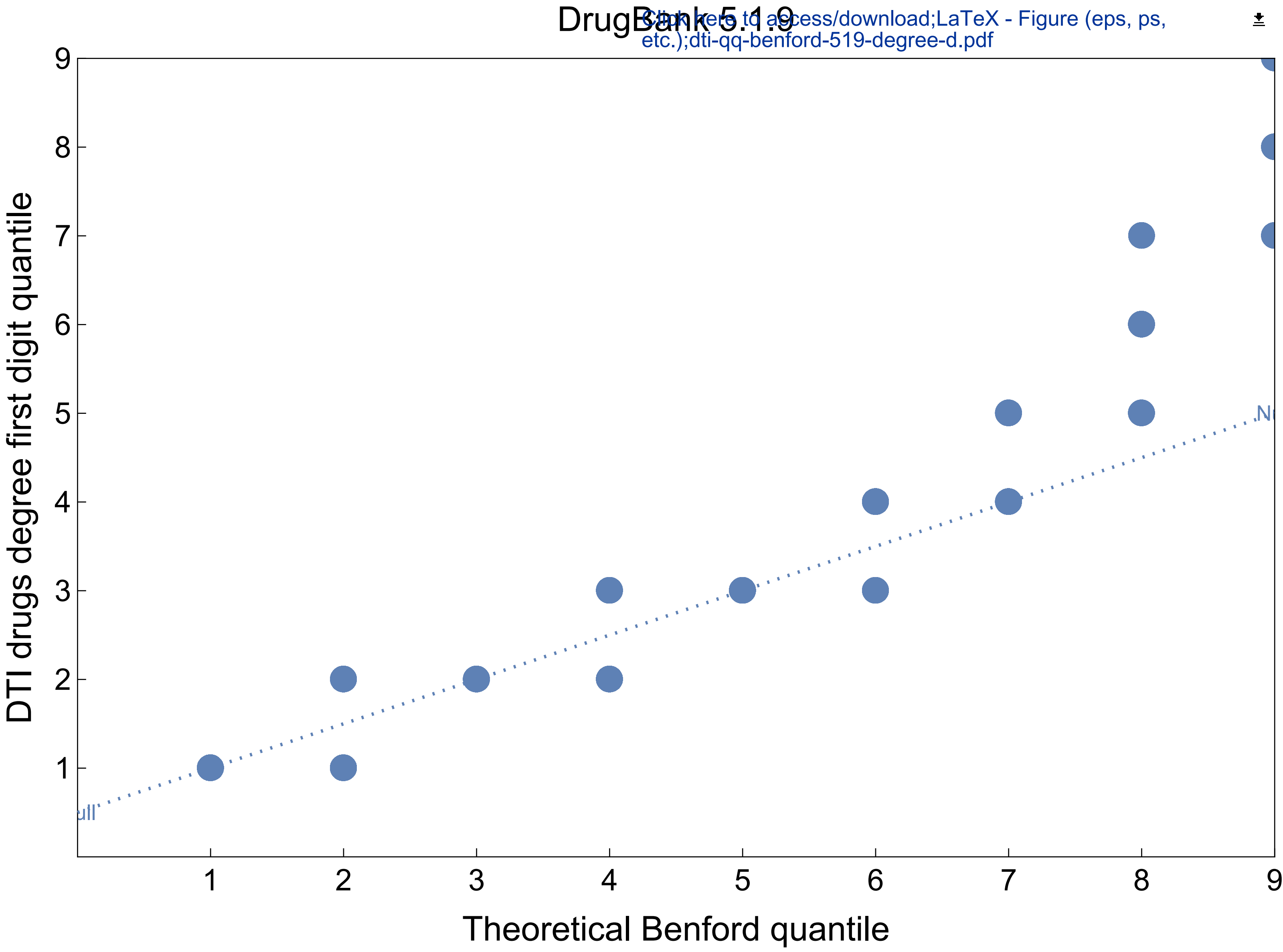

Click here to access/download;LaTeX - Figure (eps, ps, etc.);dti-qq-benford-519-degree-t.pdf

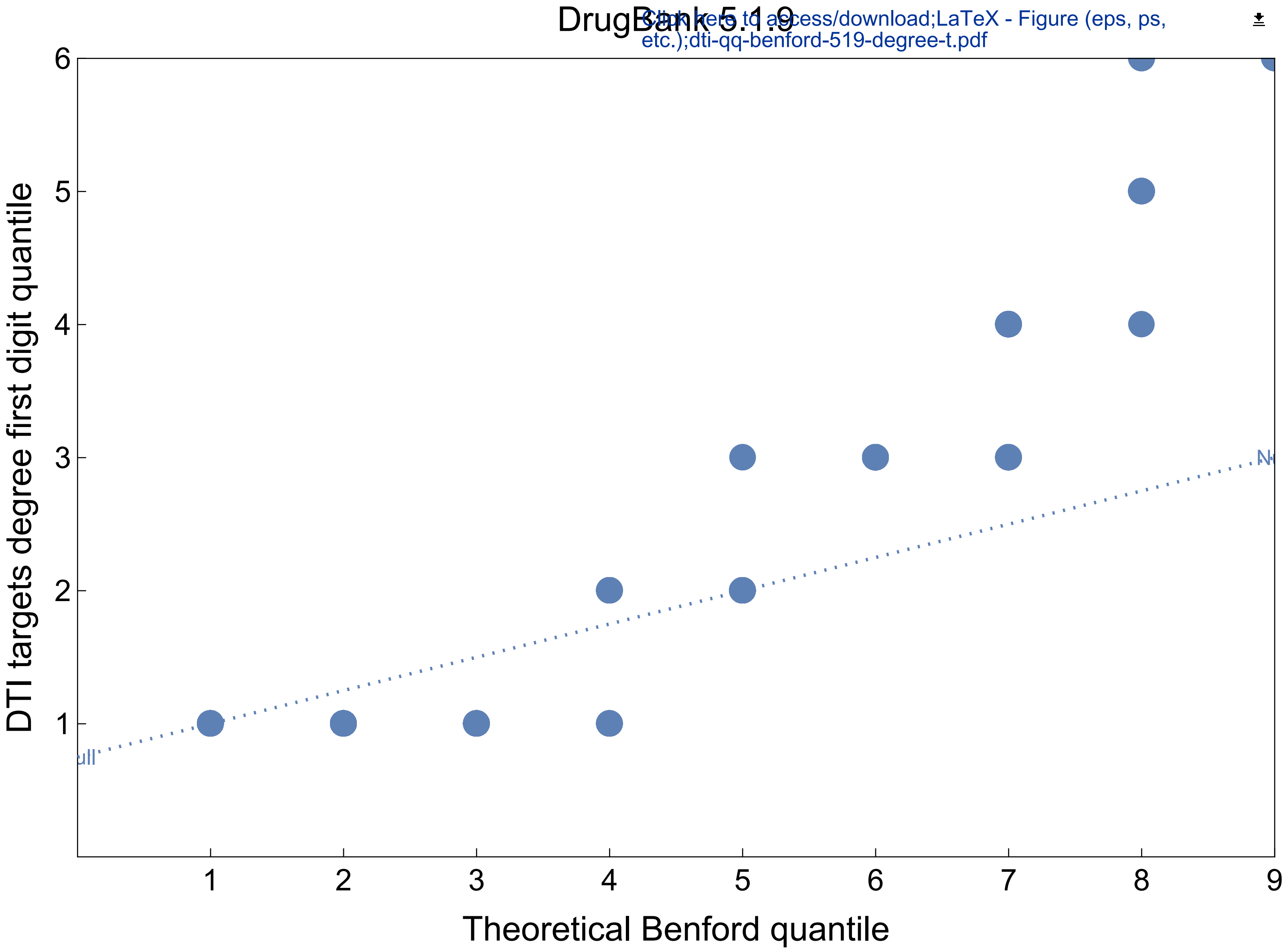

Click here to access/download;LaTeX -
Figure (eps, ps, etc.);eigDistrib30-

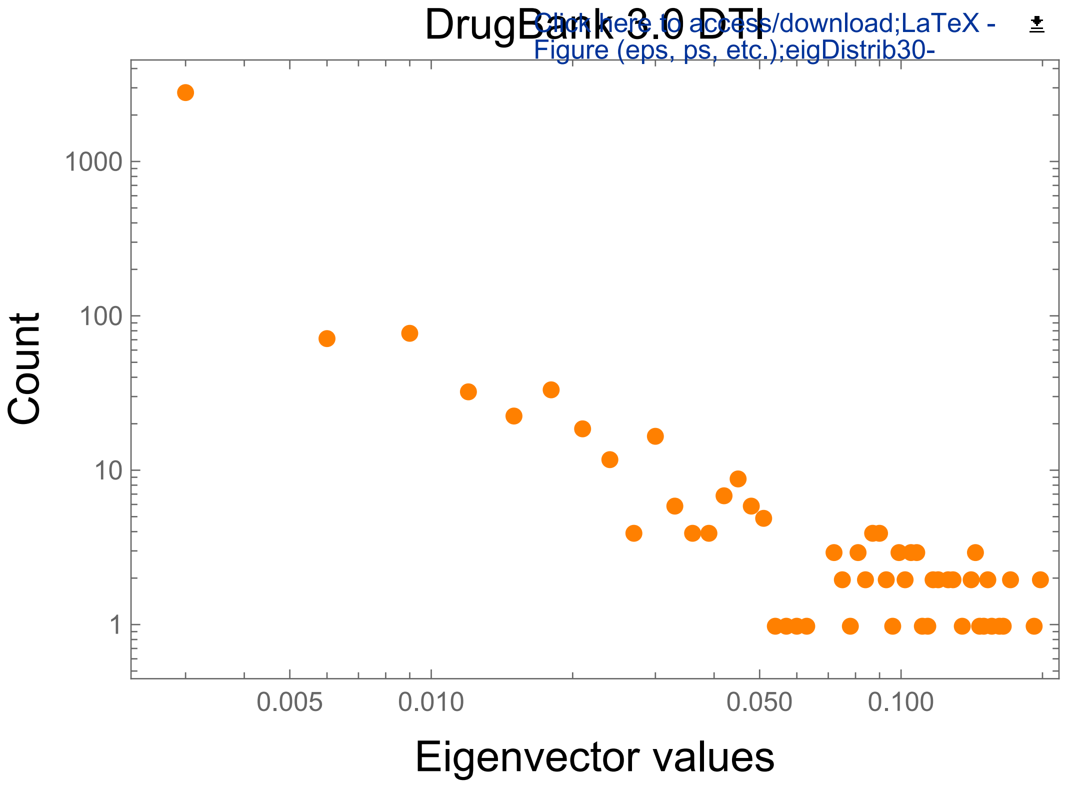

Click here to access/download;LaTeX - Figure (eps, ps, etc.);eigDistrib30.pdf

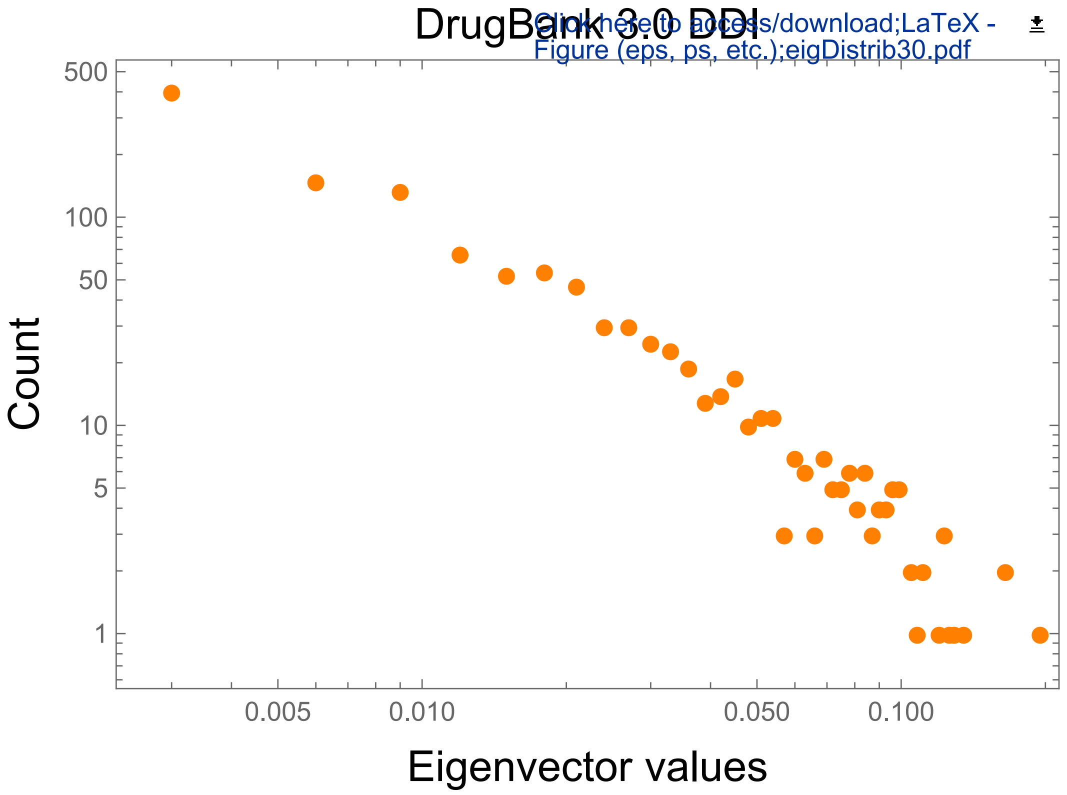

Click here to access/download;LaTeX - Figure (eps, ps, etc.);eigDistrib519-

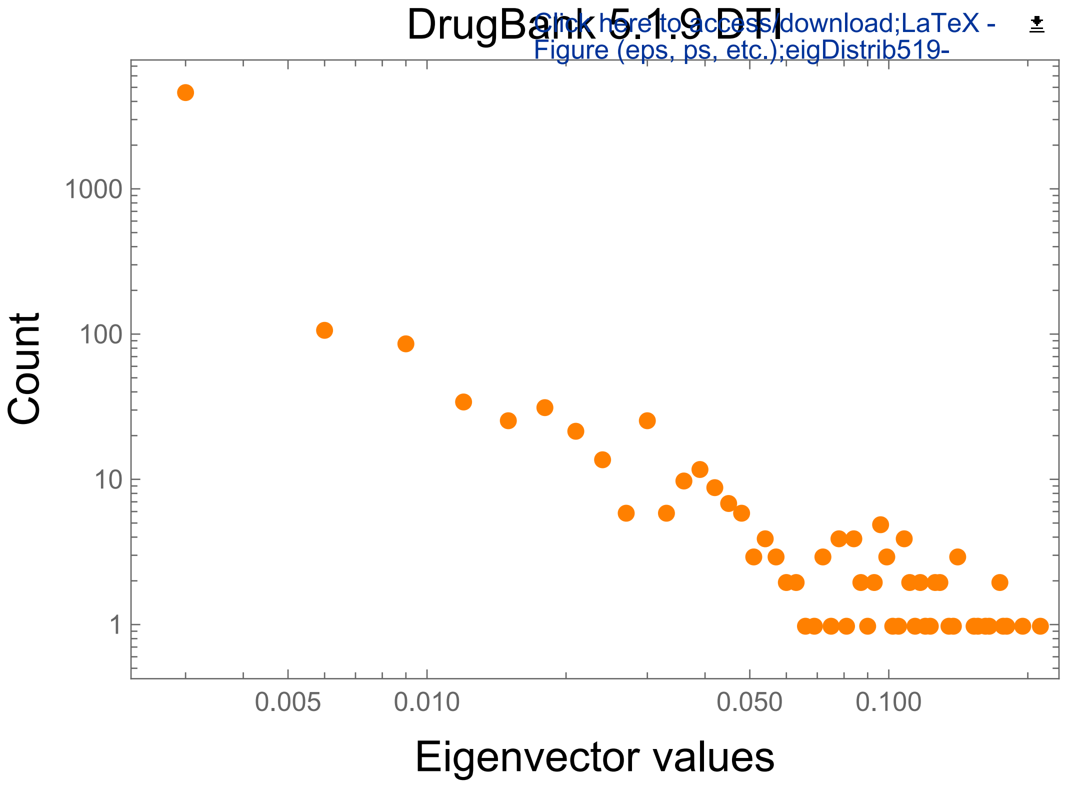

Click here to access/download;LaTeX -
Figure (eps, ps, etc.);eigDistrib519.pdf

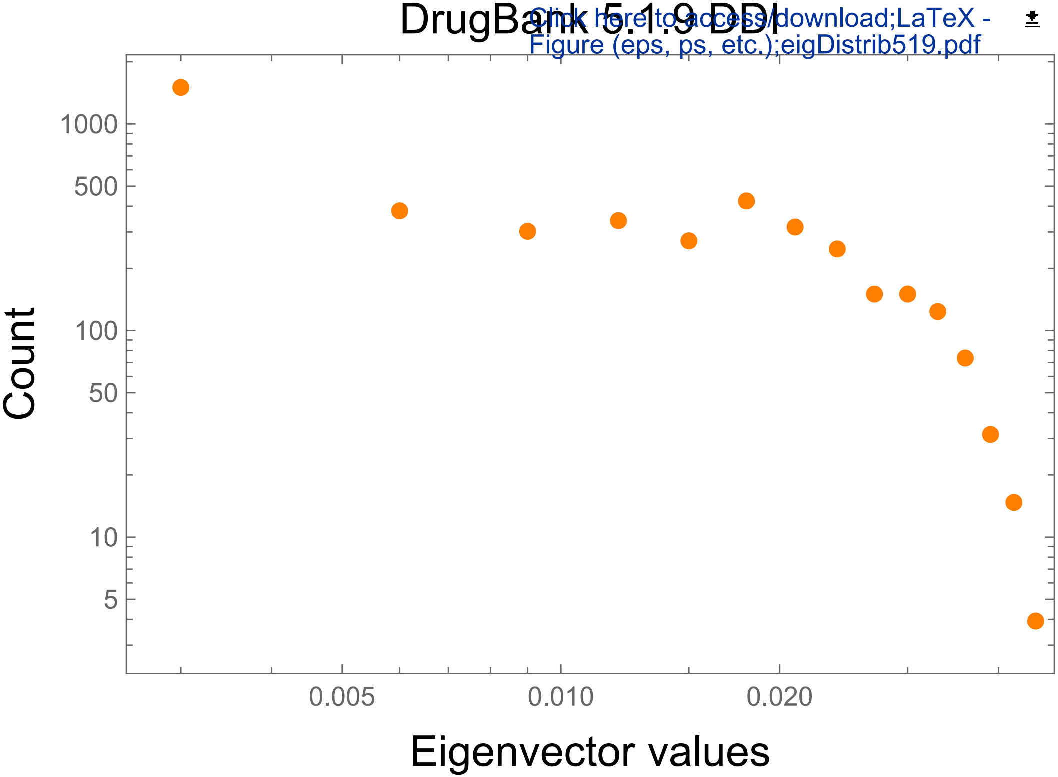

Click here to access/download;LaTeX - Figure (eps, ps, etc.);KendallBet.pdf

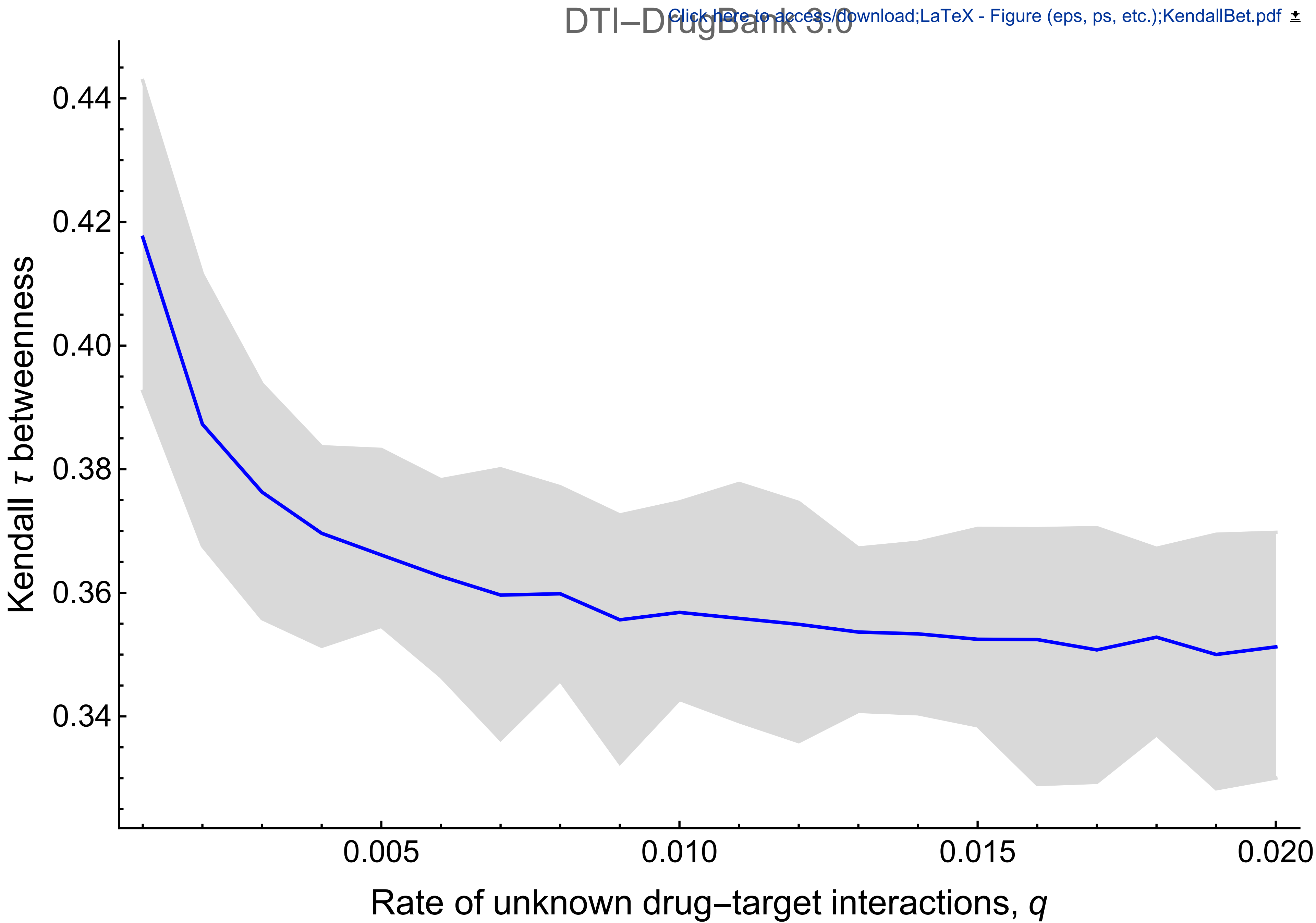

Click here to access/download;LaTeX - Figure (eps, ps, etc.);KendallDeg.pdf

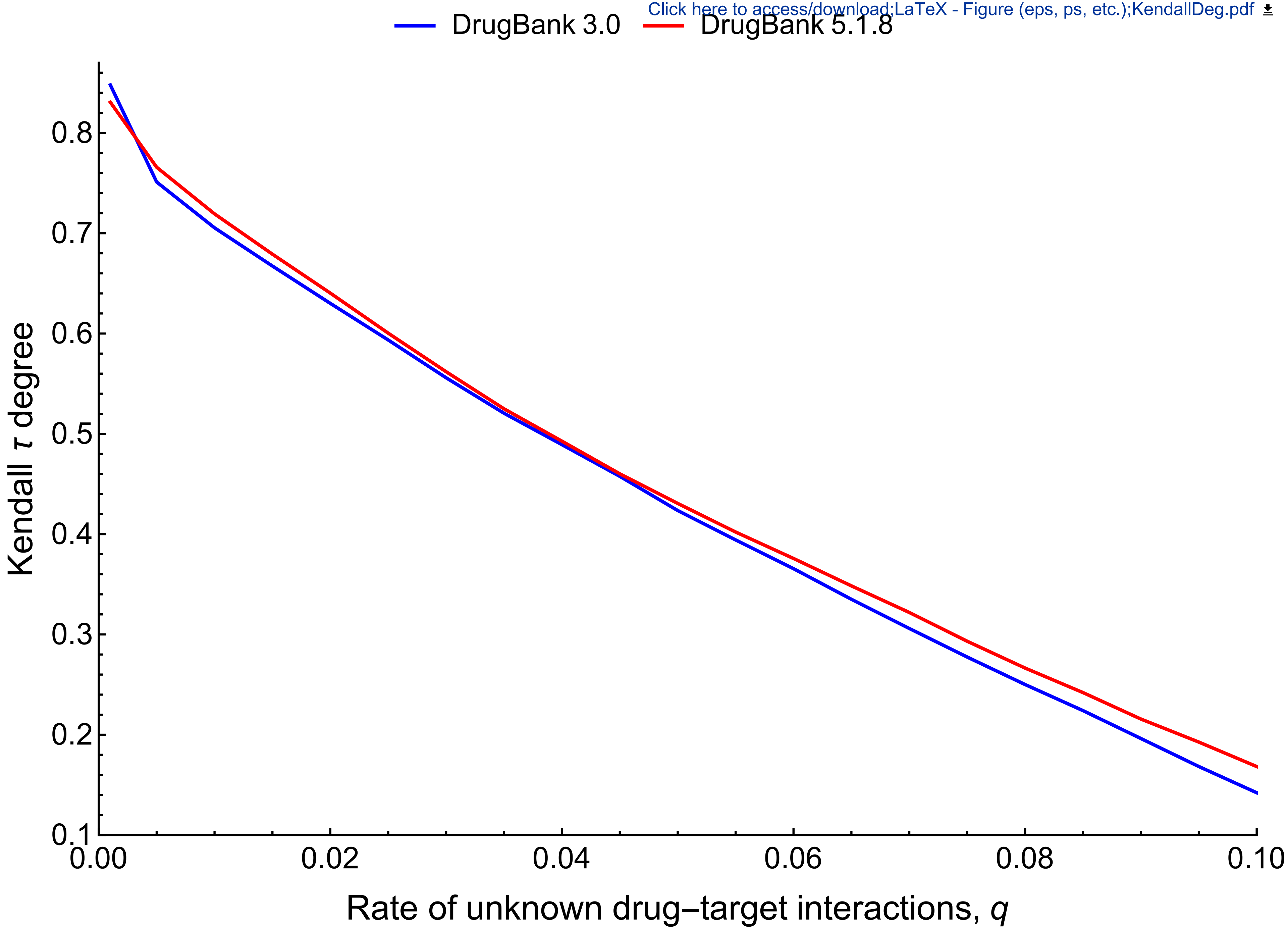

Click here to access/download;LaTeX - Figure (eps, ps, etc.);KendallDeg30.pdf

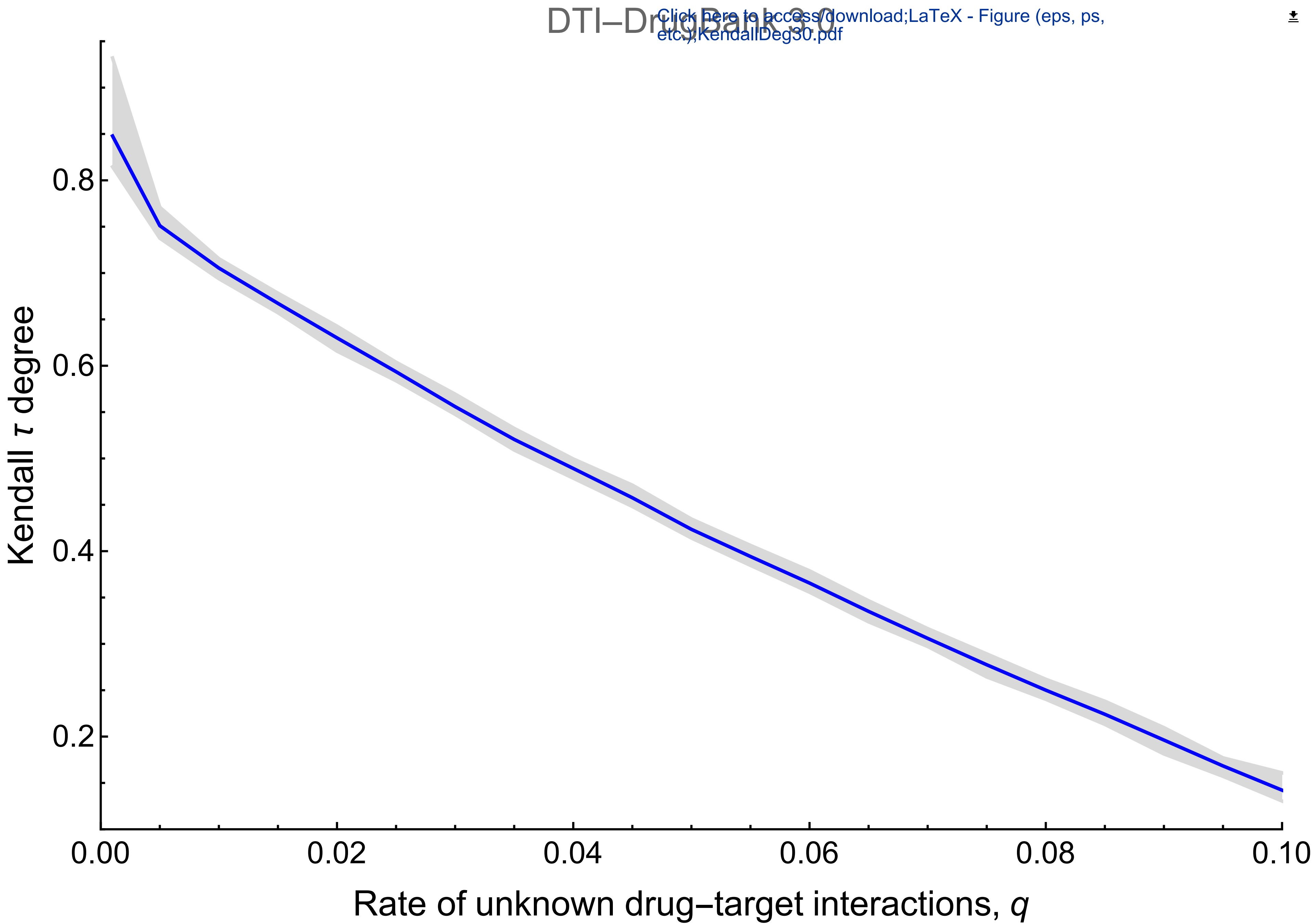

Click here to access/download;LaTeX - Figure (eps, ps, etc.);KendallDeg518.pdf

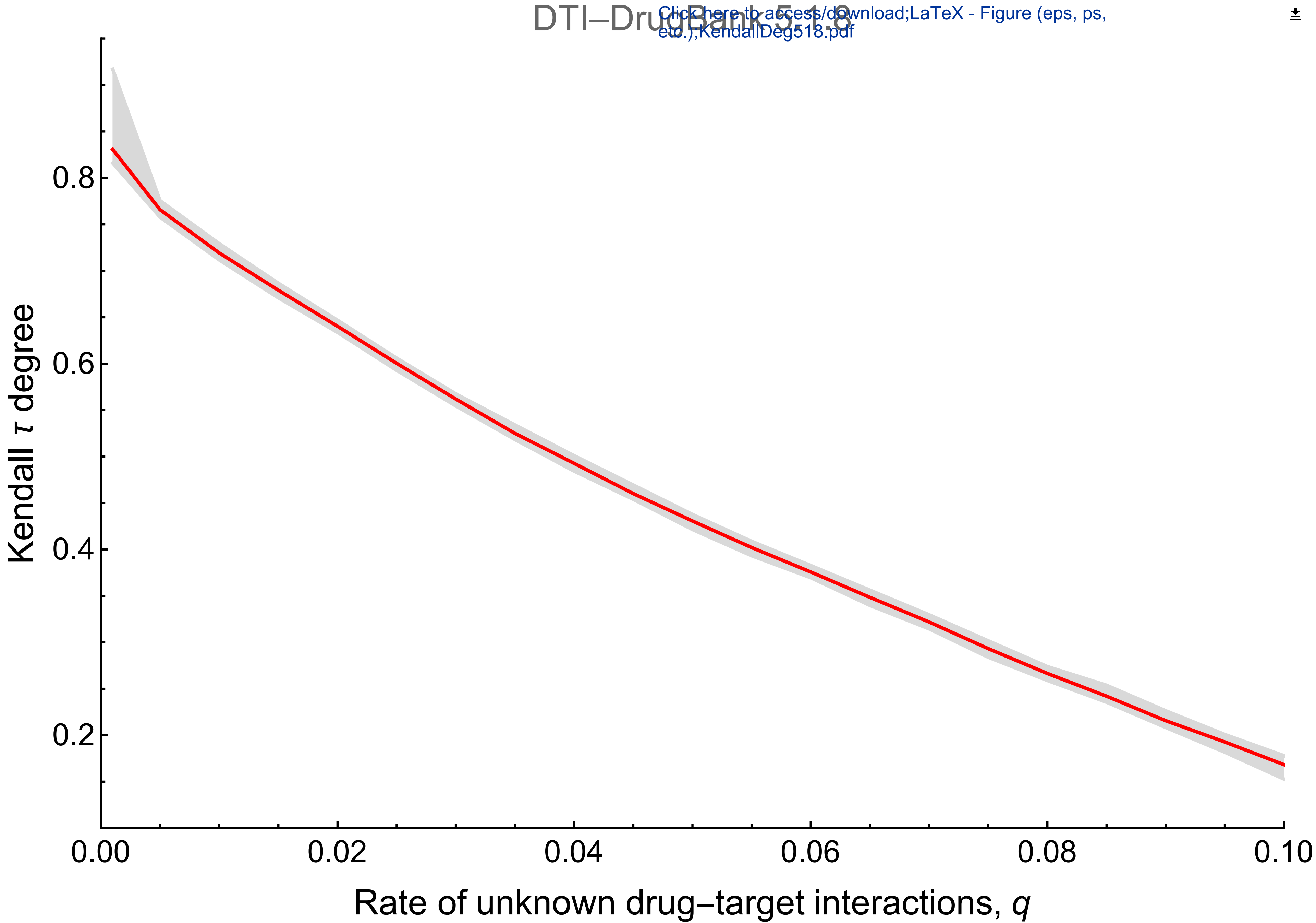

Click here to access/download;LaTeX - Figure (eps, ps, etc.);links-ddi.pdf

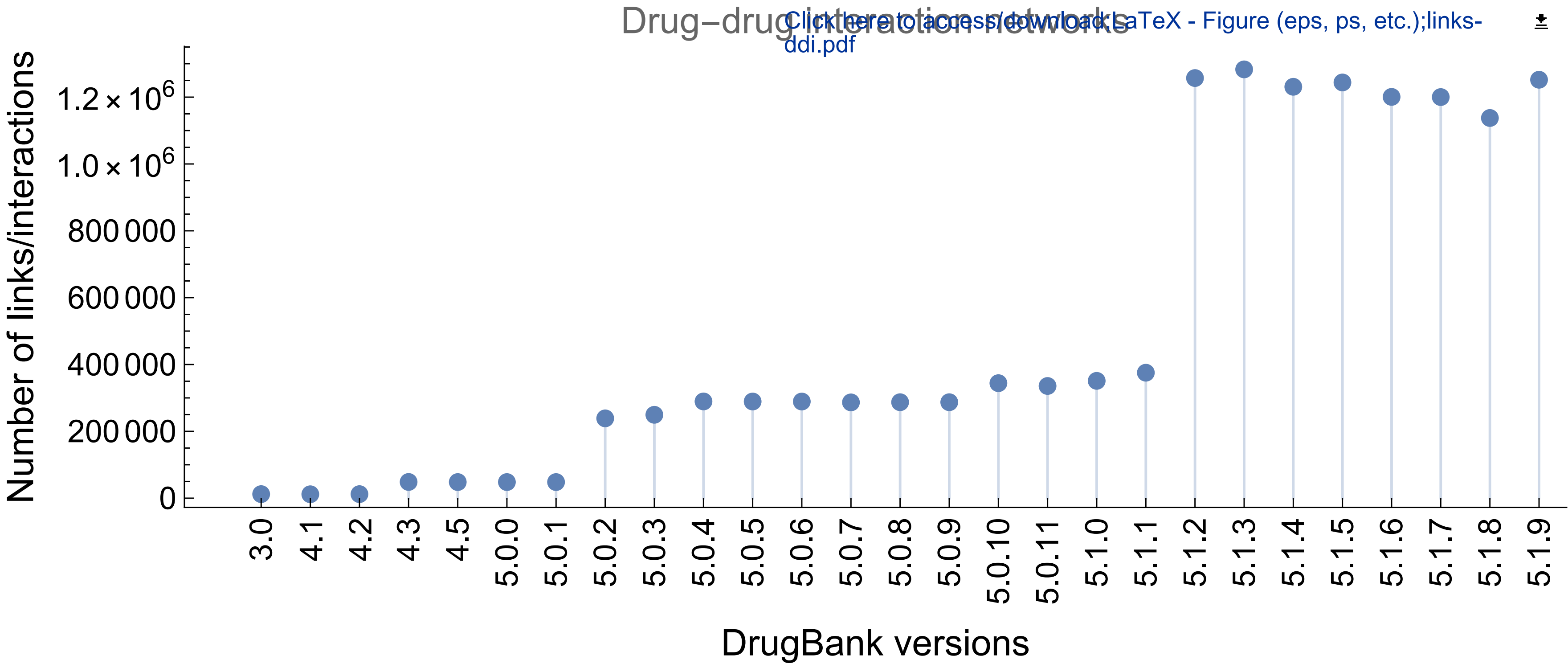

Click here to access/download;LaTeX - Figure (eps, ps, etc.);links-dti.pdf

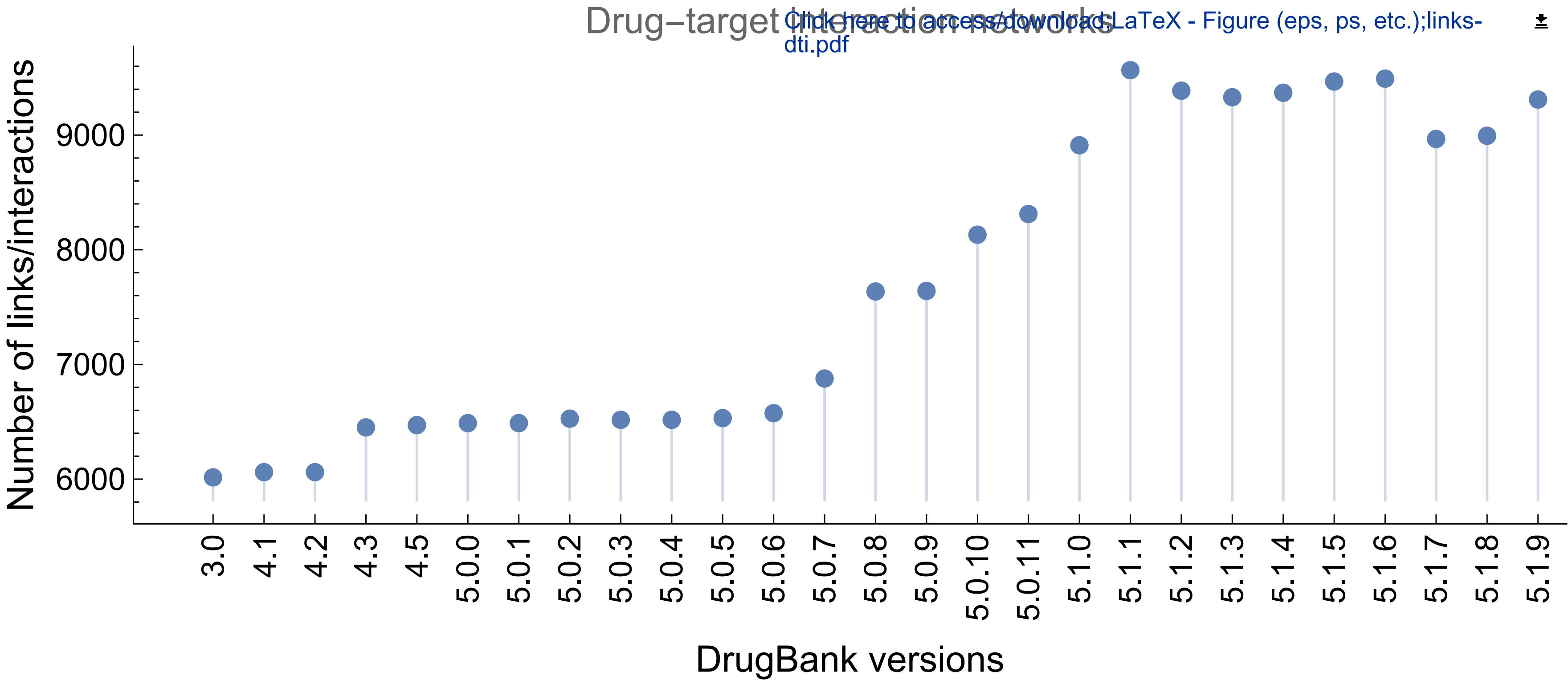

Click here to access/download;LaTeX - Figure (eps, ps, etc.);no-ddi.pdf

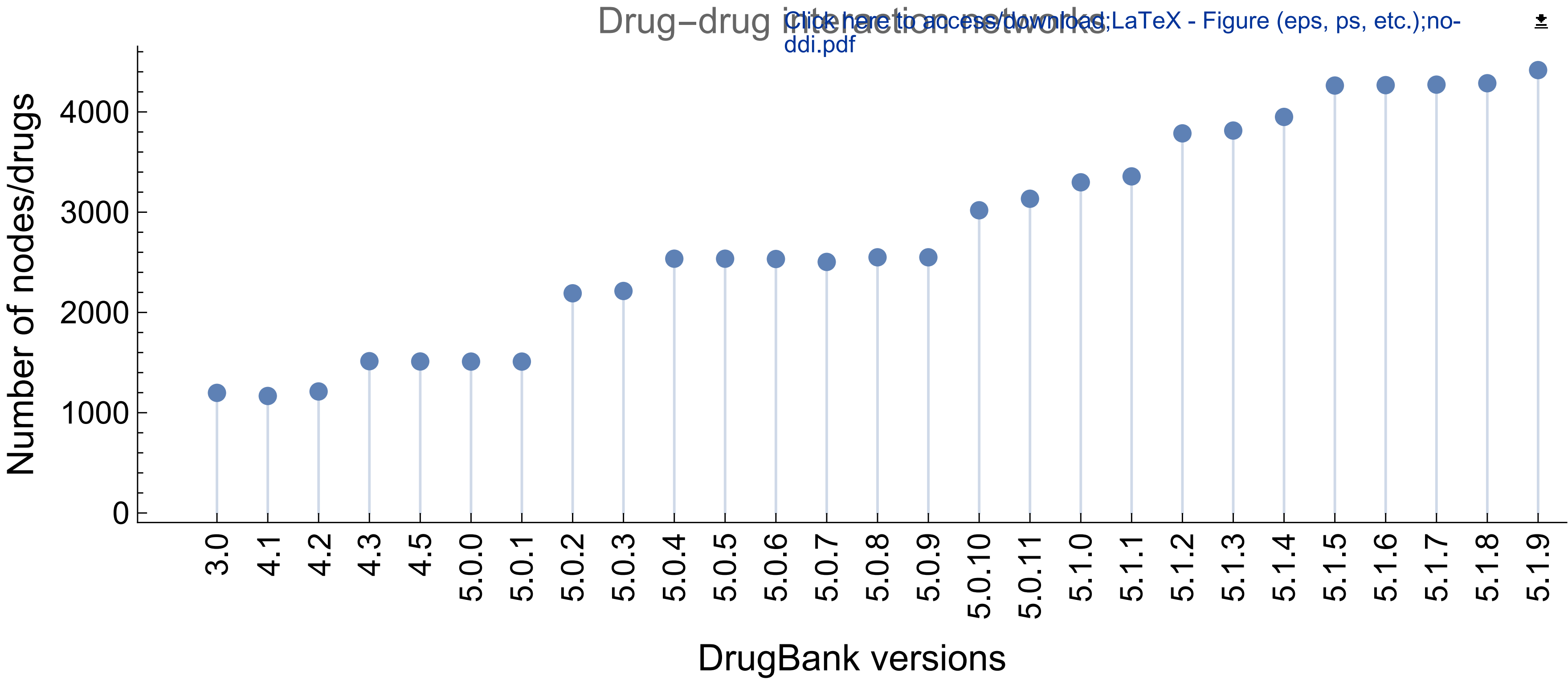

Click here to access/download;LaTeX - Figure (eps, ps, etc.);no-dti.pdf

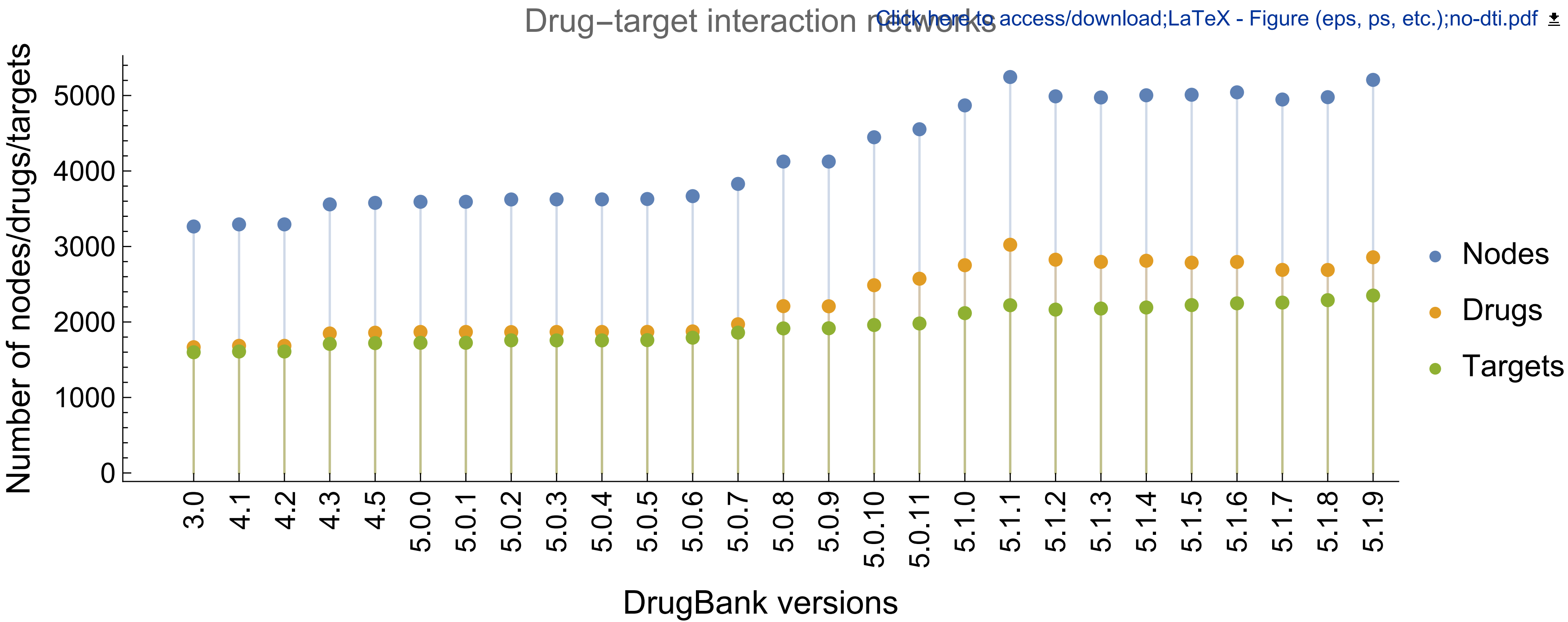

Click here to access/download;LaTeX - Figure (eps, ps, etc.);pagDistrib30-

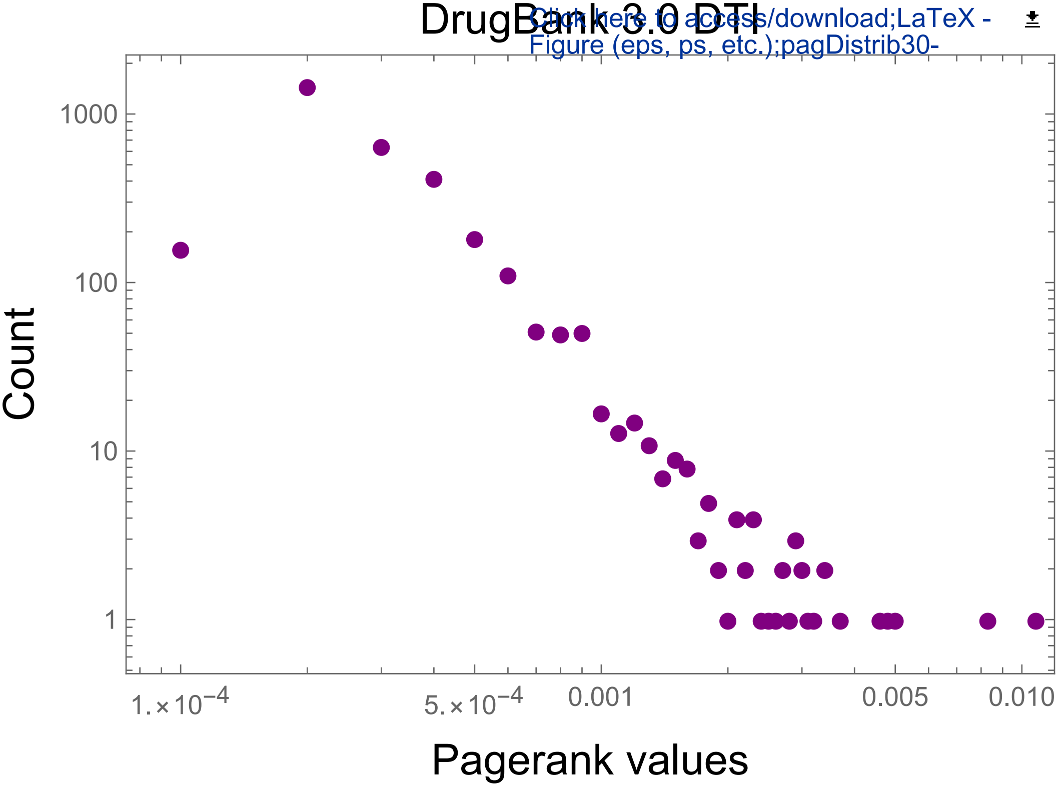

Click here to access/download;LaTeX - Figure (eps, ps, etc.);pagDistrib30.pdf

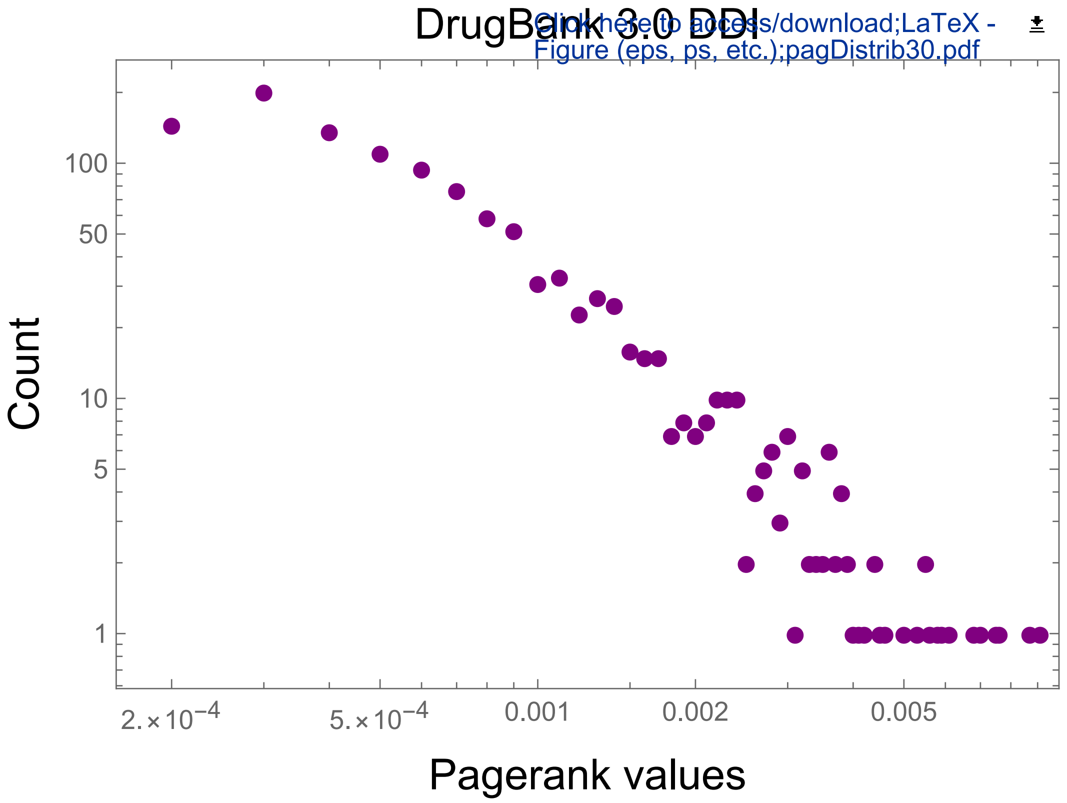

Click here to access/download;LaTeX - Figure (eps, ps, etc.);pagDistrib519-

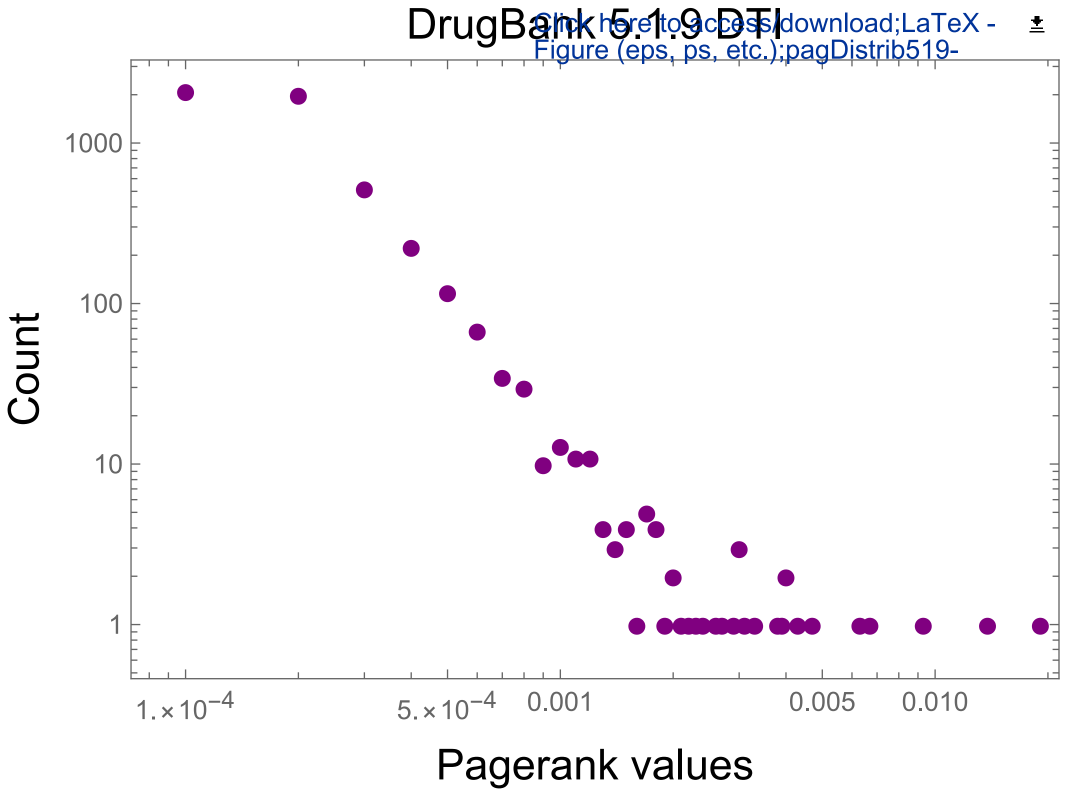

Click here to access/download;LaTeX - Figure (eps, ps, etc.);pagDistrib519.pdf

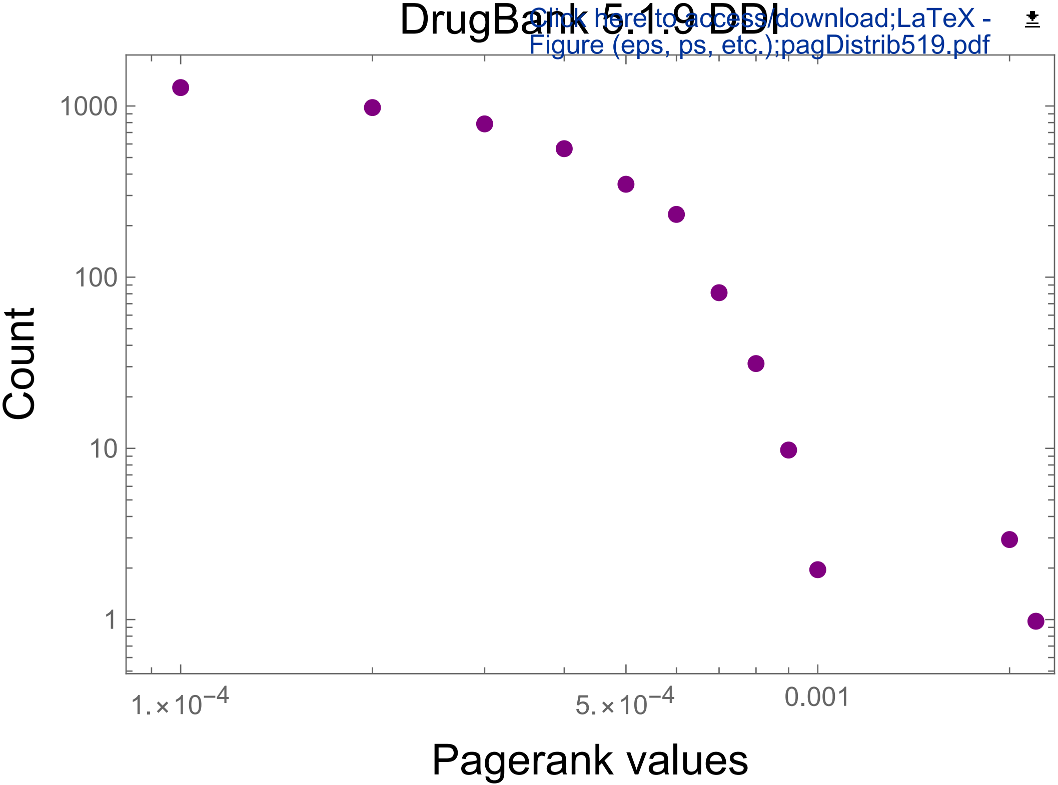

Click here to access/download;LaTeX - Figure (eps, ps, etc.);qq-benford30-bet.pdf

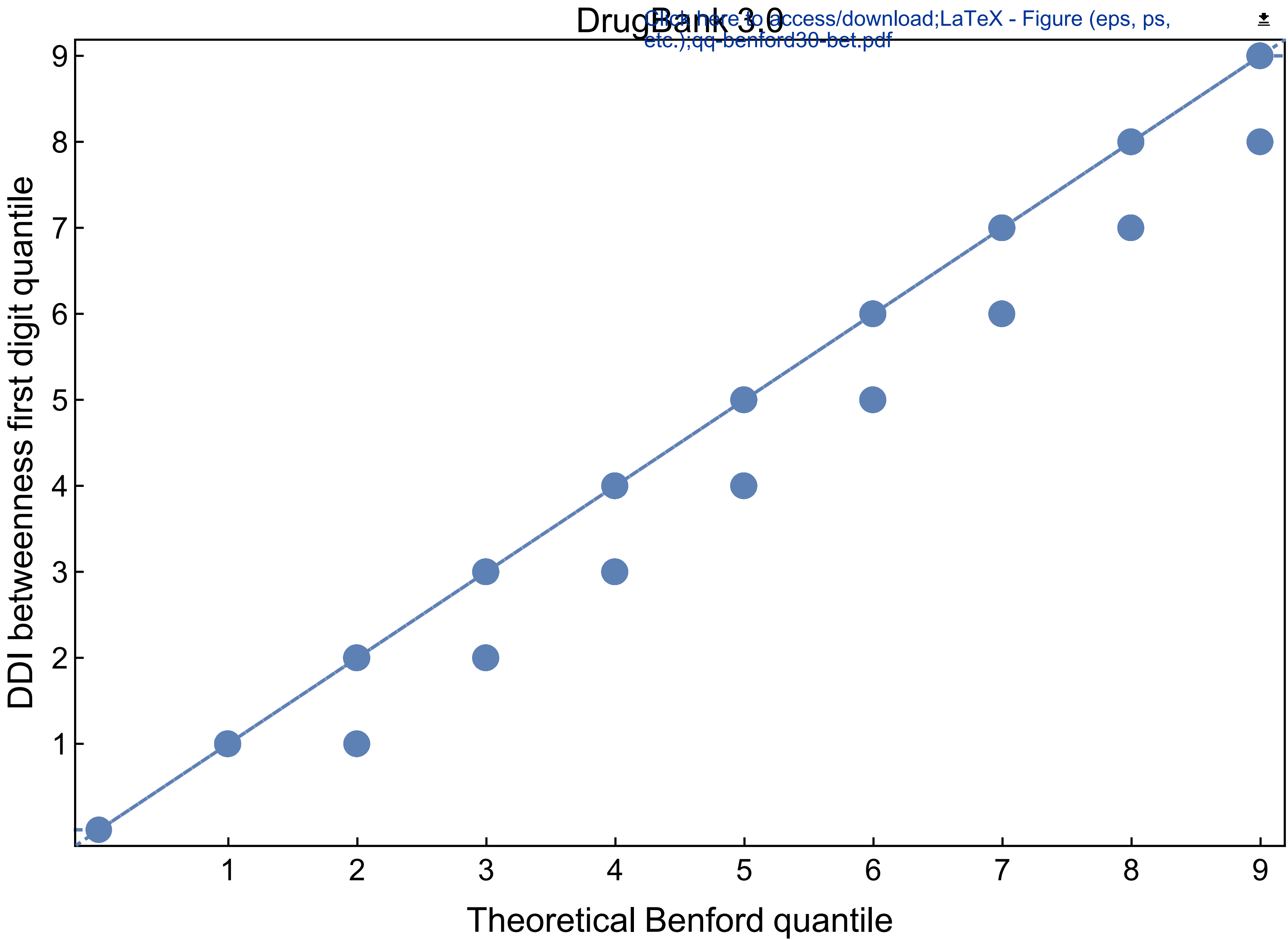

Click here to access/download;LaTeX - Figure (eps, ps, etc.);qq-benford30-deg.pdf

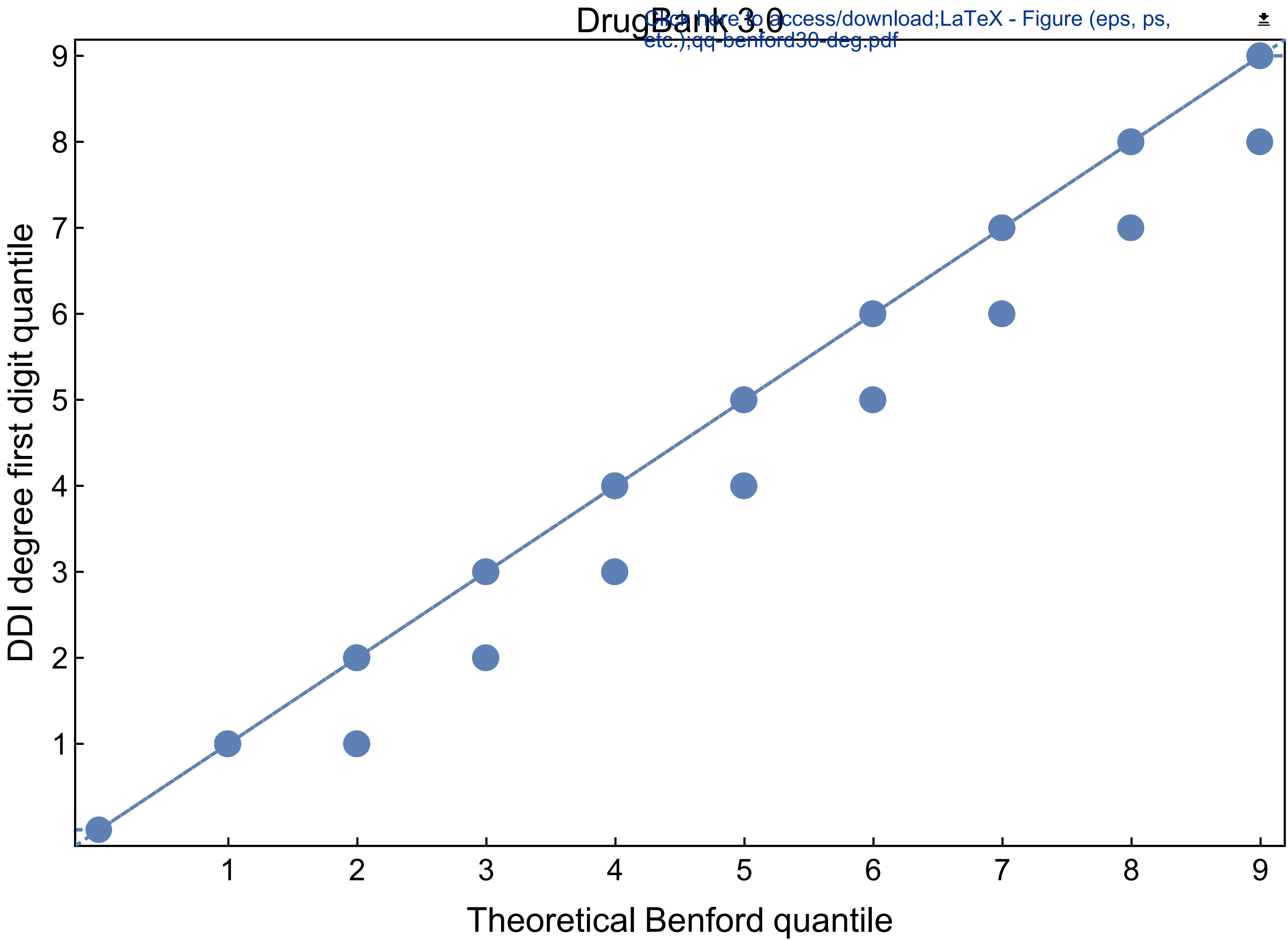

Click here to access/download;LaTeX - Figure (eps, ps, etc.);qq-benford519-bet.pdf

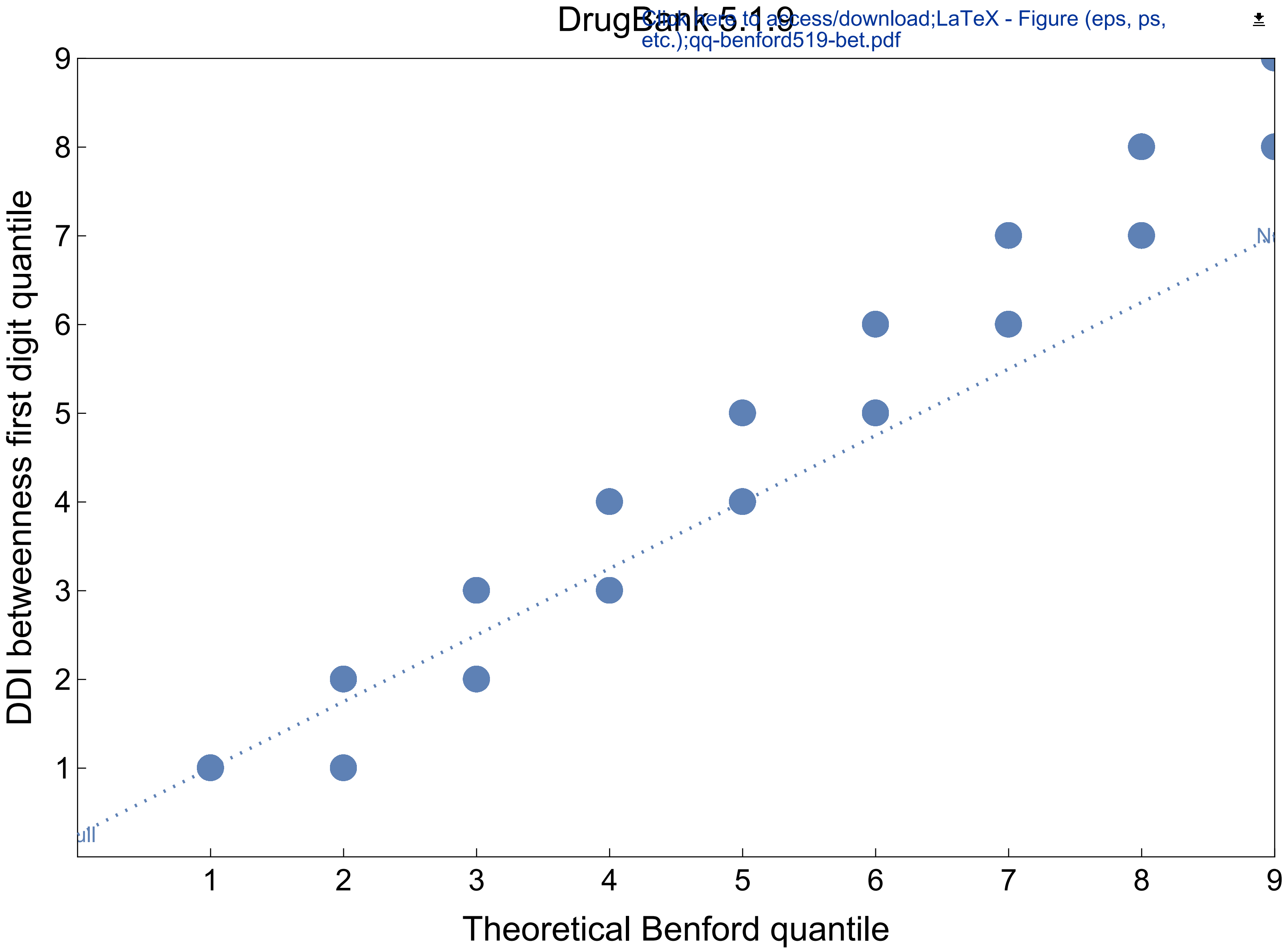

Click here to access/download;LaTeX - Figure (eps, ps, etc.);qq-benford519-deg.pdf

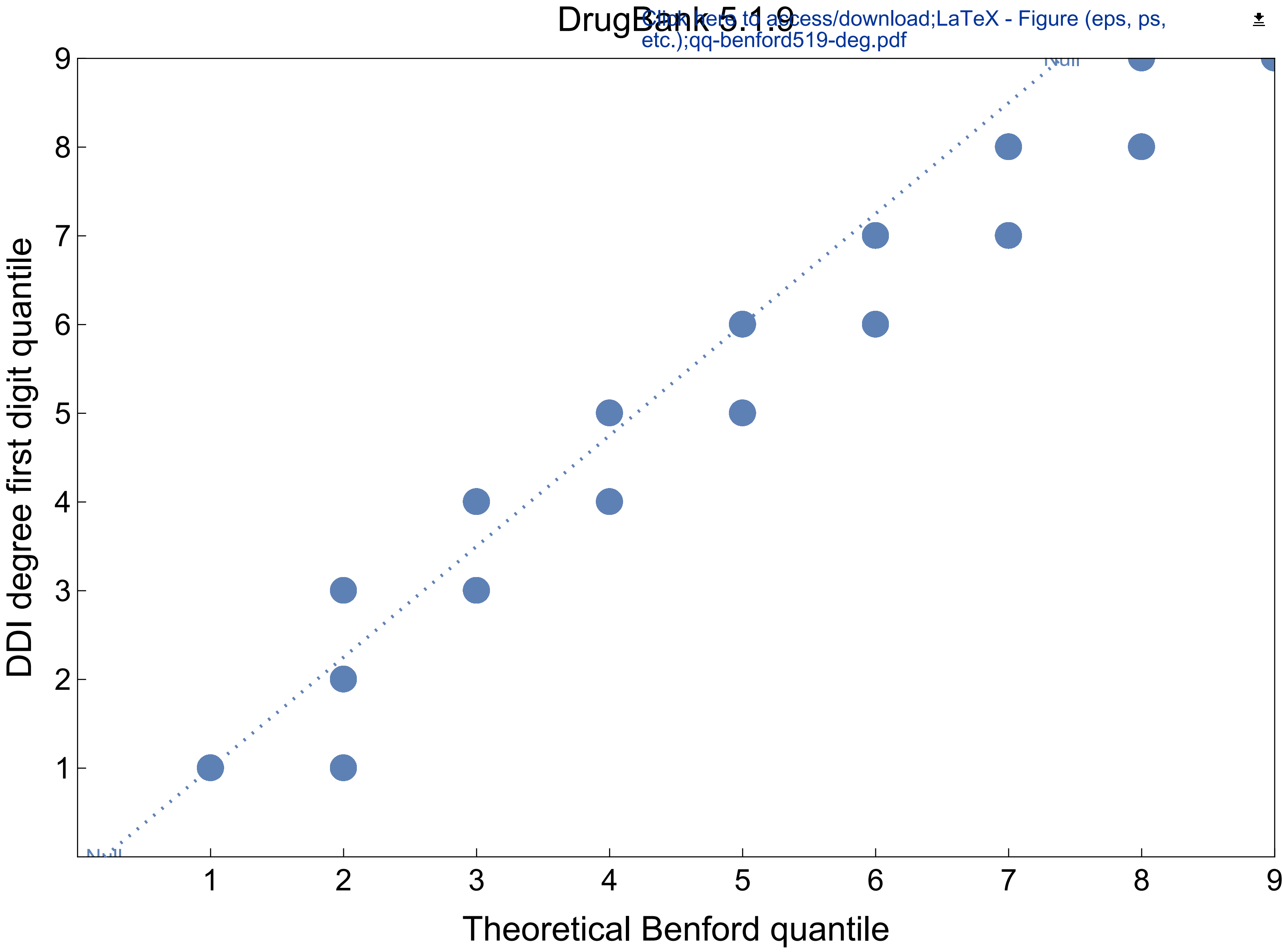

Manuscript

Click here to access/download;Manuscript;The_curse_and_blessing_of_abu

GigaScience, 2022, 1–21

doi: xx.xxxx/xxxx
Manuscript in Preparation
Paper

PAPER

The curse and blessing of abundance—the evolution of drug interaction databases and their impact on drug network analysis

Mihai Udrescu[1,*], Sebastian Mihai Ardelean[1] and Lucreţia Udrescu[2]

[1]Department of Computer and Information Technology, Politehnica University of Timișoara, Vasile Pârvan Blvd.,300223, Timișoara, Romania and [2]Department I—Drug Analysis, "Victor Babeș" University of Medicine and Pharmacy Timișoara, Eftimie Murgu Sq., 300041, Timișoara, Romania

*mudrescu@cs.upt.ro

Abstract

Background
Widespread bioinformatics applications such as drug repositioning or drug-drug interaction prediction rely on the recent advances in machine learning, complex network science, and comprehensive drug datasets comprising the latest research results in molecular biology, biochemistry, or pharmacology. The problem is that there is much uncertainty in these drug datasets—we know the drug-drug or drug-target interactions reported in the research papers, but we cannot know if the not reported interactions are absent or yet to be discovered. This uncertainty hampers the accuracy of such bioinformatics applications.
Results
We use complex network statistics tools and simulations of randomly-inserted previously unaccounted interactions in drug-drug and drug-target interaction networks—built with data from DrugBank versions released over the last decade—to investigate whether the abundance of new research data (included in the latest dataset versions) mitigate the uncertainty issue. Our results show that the drug-drug interaction networks built with the latest dataset versions become very dense and, therefore, almost impossible to analyze with conventional complex network methods. On the other hand, for the latest drug database versions, drug-target networks still include much uncertainty; however, the robustness of complex network analysis methods slightly improves.
Conclusions
Our big data analysis results pinpoint future research directions to improve the quality and practicality of drug databases for bioinformatics applications: benchmarking for drug-target interaction prediction and drug-drug interaction severity standardization.

Key words: drug-drug interaction networks; drug-target interaction networks; drug database integrity; analysis robustness of drug networks

Background

Spurred by the fast development of efficient complex network analysis tools based on machine learning and the evergrowing drug/medicine databases we witnessed over the last decade, processing drug interaction networks became an appealing drug design method [1, 2]. Such drug interaction networks can represent various relationships or interactions involving active substances, e.g., drug-drug interactions, drug-target interactions, drug-gene interactions, drug-disease relationships, drug–adverse reaction relationships. As such, harnessing big amounts of data describing the intricate drug interactions (or relationships) with other entities

Compiled on: November 18, 2022.
Draft manuscript prepared by the author.

Key Points

- Many notable and helpful bioinformatics applications, such as drug repositioning and drug-drug interaction prediction, employ machine learning methods and statistical analysis on drug-drug and drug-target interaction network features (i.e., parameters/metrics and centralities).
- The main problem with the approach based on the drug interaction network analysis is that there is much uncertainty in drug databases: the reported drug-drug and drug-target interactions are certain, but it is uncertain if the non-reported interactions do not exist or are yet to be uncovered.
- Versioned drug databases—including DrugBank—record research results accumulated over the last decade, thus enabling network analysis that exposes the weak points and provides hints to improve the drug databases' practicality and the drug network analysis's accuracy.
- Our complex network analysis on the evolution of drug-drug and drug-target interaction networks shows that the drug-drug interaction networks made with the latest dataset versions have become too dense to analyze with established complex network methods.
- We do not notice the same density increase in drug-target networks; despite containing much uncertainty, the robustness of drug-target network analysis methods scarcely improves with the evolution of drug database versions.
- Our investigation provides guidance for future studies to improve the usefulness of drug databases and the accuracy of bioinformatics applications: standardization of drug-drug interaction labeling and delivering a comprehensive benchmark dataset for drug-target interaction prediction.

can lead to uncovering new drug properties: previously unknown drug-drug [3, 4, 5, 6] or drug-target interactions [7, 8, 9, 10, 11], drug repositioning [12, 13, 14].

Computational drug-drug interaction (DDI) prediction is critical in designing effective and safe drug combinations and developing drugs [5, 6]. Drug-target interaction (DTI) prediction is a key-step strategy in drug discovery that leads to uncovering new drugs for biological targets and new targets for known, approved drugs—representing a crucial step towards drug repositioning and identifying possible side effects [8, 11].

Drug repositioning (or repurposing) means finding new applications for drugs already in use [15]; the drug propensity for multiple functions underpins this undertaking. Before the upswing of big data and machine learning, pharmacologists and medical doctors mainly relied on serendipity to uncover drug repositionings [16]. The most illustrative example is aspirin, introduced as an antipyretic but—over time—revealed as having painkiller and antiplatelet effects. The prediction of drug-drug interactions and drug-drug interaction severity is another drug-interaction network analysis application with substantial benefits in therapeutic practice [17, 18, 19]. It is clear that predicting either adverse or synergistic interactions in drug-drug interaction networks help to tailor effective therapies for patients with multiple comorbidities.

In comparison with traditional drug design, drug repositioning entails simpler testing and validation procedures (because many adverse events and effects, as well as interactions with food and other drugs, are already known and tested), which translates into reduced costs and approval times [20, 21]. (In the FDA New Drug Therapy Approvals 2021, 50 drugs are are *new molecular entities* or *new therapeutic biologics*, and 17 are *drug repositionings* [22].) All these arguments make drug repositioning tempting for exceptional therapeutical cases, such as orphan diseases—for which there is insufficient research funding [23]—and new-pathogen epidemic diseases [24]—for which timing is essential. Particularly relevant is the case of the COVID-19 pandemic, where drug repositioning proved to be a valuable therapeutical method for quick public healthcare response, given that the conventional drug design requires a substantial amount of time. Indeed, many COVID-19 repositioning predictions uncovered with big data exploration and complex drug networks were confirmed by in vitro and in vivo experiments [25].

Despite the advances in machine learning, big data mining, complex network analysis, and the undeniable benefits of computational drug repositioning, the field still has significant problems. The most critical issue that affects the robustness of computer-based drug network analysis is that the drug interactions/relationships (e.g., with other drugs, targets, adverse reactions, or diseases) in drug databases primarily reflect what we know as positive information from in vitro and in vivo experiments. We have little negative information, i.e., interactions we know for sure that do not exist. If we do not have information about a specific drug interacting with a particular target, this does not mean that there is no way they interact; after all, "absence of evidence is not evidence of absence" [26].

Consequently, in computational drug repositioning, we do not have a robust ground truth to operate with; this can seriously affect the analysis accuracy of complex drug interaction networks. Mestres et al. have articulated this idea very eloquently [27] by showing how the network analysis based on degree centrality hierarchization of drugs in a network built with information from one database is affected by adding the information from another database. (In complex networks, a centrality expresses the importance of a network node/vertex; most network-based analysis approaches use either centrality-hierarchization or community detection methods.) Nonetheless, the enormous benefits of confirming (with in vitro and in vivo methods) even a few drug repositionings—uncovered by computational big data approaches—offset such accuracy problems.

As long as we do not have comprehensive negative information, the issue of uncertainty in drug datasets remains. However, the fact that—over the years—the comprehensiveness of drug datasets (such as DrugBank [28]) has constantly grown, and experts curated the data according to the latest literature results, may suggest that the integrity of the data improved. (The integrity characterizes the "absence of improper data alterations" [29].) Indeed, the constant growth of the most comprehensive drug database (reflected in the evolution of the various complex drug network parameters and centralities) may have mitigated the concerns formulated by Mestres et al. [27]. Our paper investigates how the evolution of knowledge on drug interactions mirrored by the DrugBank database, impacts the robustness of drug interaction network analysis based on centrality hierarchization methods; future research can exploit the insight we get to advance drug repositioning and drug-drug interaction prediction methods.

We build the drug-drug interaction (DDI) and drug-target interaction (DTI) networks with the data from DrugBank versions 3.0 to 5.1.9. Over the years, many research papers used DDI [30, 31, 32, 33, 34, 35] and DTI networks [36, 37, 38, 39, 40] for computational drug repositioning and drug-drug interaction pre-

diction. A drug-drug interaction occurs when one drug influences the action/effect of another drug in a biological environment. Such a drug-drug relationship signifies that one drug augments or, conversely, that mitigates the effect of the other one; either way, this generally translates into a clinically potential harmful situation. A drug-target interaction exists if the drug exerts a specific action upon a biological target (generally, a protein or enzyme), thus producing a pharmacological effect [40].

We found the motivation of our research in the visual inspection of the evolution of DDI and DTI networks over the years and across the successive DrugBank versions. Figure 1 shows how the DDI network density increased from version 3.0 to 5.1.9, via 5.0.8, such that the number of network clusters/communities substantially decreased; moreover, as shown in the panels below, the initial power-law degree distribution in DrugBank 3.0 DDI is altered in the subsequent versions. In contrast with the DDI network evolution presented in Figure 1, the equivalent evolution for DTI networks in Figure 2 does not increase the density substantially, and the structure does not change too much despite the increasing size of the networks—from version 3.0 to 5.0.8 and 5.1.9. Also, the panels below the DTI networks in Figure 2 show that the DTI degree distributions in both drugs and targets are power-law across all DrugBank versions. Indeed, this discrepancy between DDI and DTI network evolution over time (as reflected by the successive DrugBank data versions) inspires the study we present in this paper; the results obtained provide valuable insight for researchers developing big data techniques in systems and networks pharmacology, aiming at applications such as computational drug repositioning or drug-drug interaction prediction.

The contributions of our DDI and DTI network analysis across the DrugBank database versions are:

- We present for the first time the evolution of various complex network parameters and centralities [41], in drug-drug and drug-target interaction networks as the knowledge on drug-drug and drug-target interactions grew over more than a decade; we comment on the far-reaching consequences of data evolution on the computational analysis tools used in drug repositioning.
- We estimate the integrity of the processed data using Benford's law on the most prominent network centralities (i.e., degree and betweenness).
- We test the robustness of centrality-hierarchization analysis methods in drug interaction networks using our algorithm that automatically adds unknown interactions in ascending ratios to notice how this process affects the drugs' order of importance.

Accordingly, one main finding is that the DDI network parameters and centralities distributions were close to those of the typical complex networks in the earlier DrugBank versions but deviated markedly in the latest versions. Instead, the DTI parameters and network centralities distributions oscillate but remain close to the typical complex network ranges across all DrugBank versions. (We explain the typical characteristics of real-world complex networks in subsection Typical complex network characteristics.)

Such an evolution of the DDI networks owes to their enormous increase in density (i.e., many recent experimental results report new drug-drug interactions); however, typical complex networks are patently sparse. The overarching conclusion is that complex network analysis became irrelevant in the DDI networks built with the latest database versions data. Also, the integrity analysis of the DTI data finds that even the latest DrugBank versions still miss many unaccounted drug-target interactions; therefore, the robustness of the centrality hierarchization analysis (i.e., degree, as suggested in [27]) in DTI networks improved only marginally with the new data in the latest versions. Such a situation calls for an intensified effort to uncover new drug-target interactions; indeed, in 2021, only 17 new drug-target interactions were approved [42].

Data description

We chose the DrugBank dataset [28, 43, 44] because it is the most comprehensive and robust drug database, being curated manually by experts and scientists, according to the latest scientific discoveries reported in the literature; it is also versioned, which allows for analyzing the evolution of knowledge over time [28].

We downloaded all DrugBank versions recorded as XML files over a decade, from version 3.0 (January 2011) to version 5.1.9 (January 2022). Access to download the database versions is free with a validated account; an account can be created with an institutional email. The DrugBank versions 5.1.9 to 4.5.0 can be downloaded from `https://go.drugbank.com/releases` and 4.3 to 3.0 from `https://go.drugbank.com/downloads/archived`.

For each medicine, DrugBank lists many parameters, properties, and extensive information, such as the generic name, brand names, indications, type, drug categories, ATC codes, chemical structure and formula, chemical identifiers, associated conditions, pharmacodynamics, mechanism of action, metabolism, toxicity, pathways, drug interactions, food interactions, clinical trials, patents, targets, enzymes, carriers, transporters, and so forth. Nonetheless, our present study analyzes the evolution of drug-drug and drug-target interaction networks (DDI and DTI), such that the only information we need from each drug in all DrugBank versions is *drug interactions* and *targets*. Also, we included information concerning *approved* human drugs only (consequently, we excluded the investigational, experimental, withdrawn, or vet-approved drugs).

For every drug in each database version, DrugBank lists the drugs with which it interacts; for example, in DrugBank 5.1.9, ibuprofen has a list of 1,236 approved interacting drugs. Each interaction has a text description; for the ibuprofen entry, the first interacting drug is abacavir, and the corresponding description reads, "Ibuprofen may decrease the excretion rate of Abacavir which could result in a higher serum level." At the same time, all DrugBank versions provide, for each drug entry, a list of interacting targets. For each interacting target, we find the following information: kind, organism (humans, in our case), pharmacological action, actions, general function, specific function, gene name, Uniprot ID, Uniprot name, molecular weight, and a list of references (i.e., scientific papers) to support the provided details experimentally. To continue with our example, DrugBank 5.1.9—the latest database version—lists 10 targets for ibuprofen (all proteins), from Prostaglandin G/H synthase 2 to Protein S100-A7.

Problem formulation

Complex networks

A *complex network* is a graph G consisting of a node (or vertex) set V and a link (or edge) set E, $G = (V, E)$, where any edge $e_{ij} \in E$ connects nodes $v_i, v_j \in V$. Additionally, compared to normal graphs, real-world complex networks have a large number of nodes $|V|$ (i.e., hundreds to millions) and intricate, nontrivial topologies (i.e., links do not form regular structures but are not completely random either). [45, 46, 41, 47]. If the nodes $v_i \in V$ are of the same type, G is a *monopartite* network; if any node v_i belongs to one of the m disjoint node subsets ($V_1 \cup V_2 \ldots \cup V_m = V$), then the network is *multipartite* or m-partite.

The network is *unweighted* if the weights of its edges/links $e_{ij} \in E$ are $w_{ij} = 1$. (In other words, if $w_{ij} = 1$ we have a link between nodes v_i and v_j, and if $w_{ij} = 0$ there is no link between v_i and v_j.) The network is *weighted* if w_{ij} are not binary values (i.e., $w_{ij} \in \mathbb{R}$); values $w_{ij} = 0$ also correspond to non-edges between v_i and v_j. We say that the network is *directed* if $\exists i, j\ (i \neq j)$ such that $w_{ij} \neq w_{ji}$. Unweighted networks are directed if $\exists e_{ij} \in E$ with $w_{ij} = 0$ and $w_{ji} = 1$.

Such networks G can also be expressed as adjacency matrices W containing elements w_{ij}, with $w_{ij} = 0$ when there is no link/edge

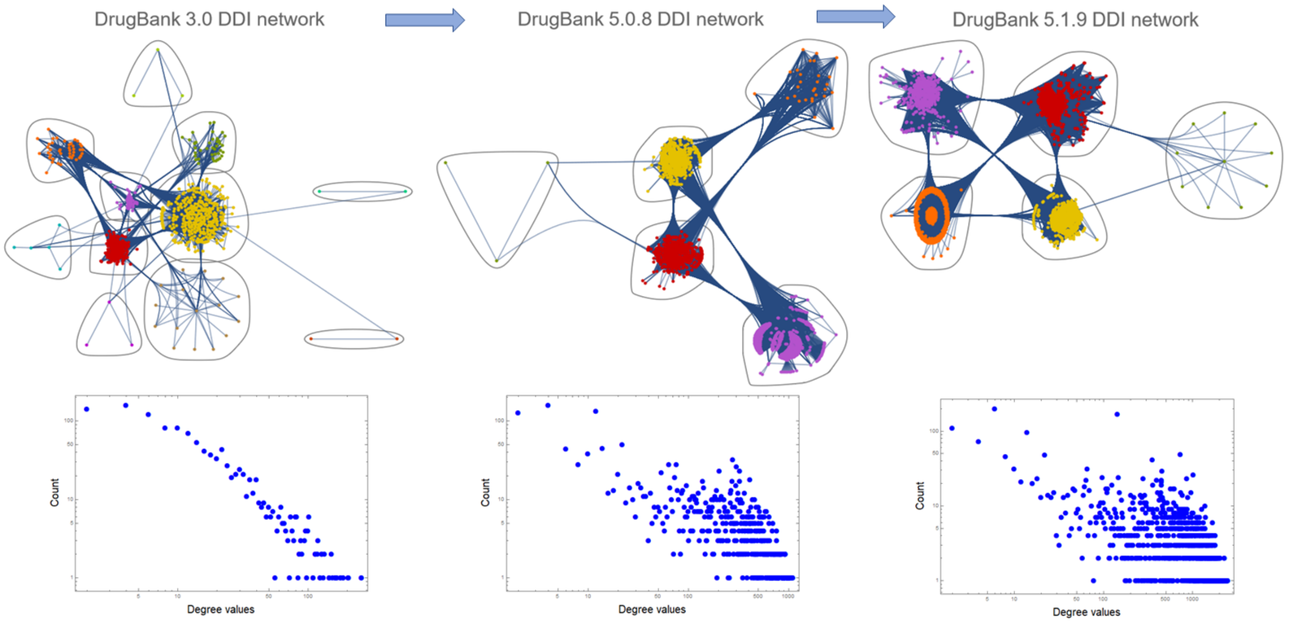

Figure 1. Drug-drug interaction (DDI) network evolution with the DrugBank data versions, showing the massive increase in density from version 3.0 to 5.1.9. Consequently, the number of network clusters/communities generated with hierarchical clustering in Mathematica 13.0 substantially decreases (nodes represent drugs, links represent drug-drug interactions, and node colors represent the distinct clusters/communities of network nodes). Also, the panels below show how the increase in density alters the power-law degree distribution in the DDI networks corresponding to the latest DrugBank versions.

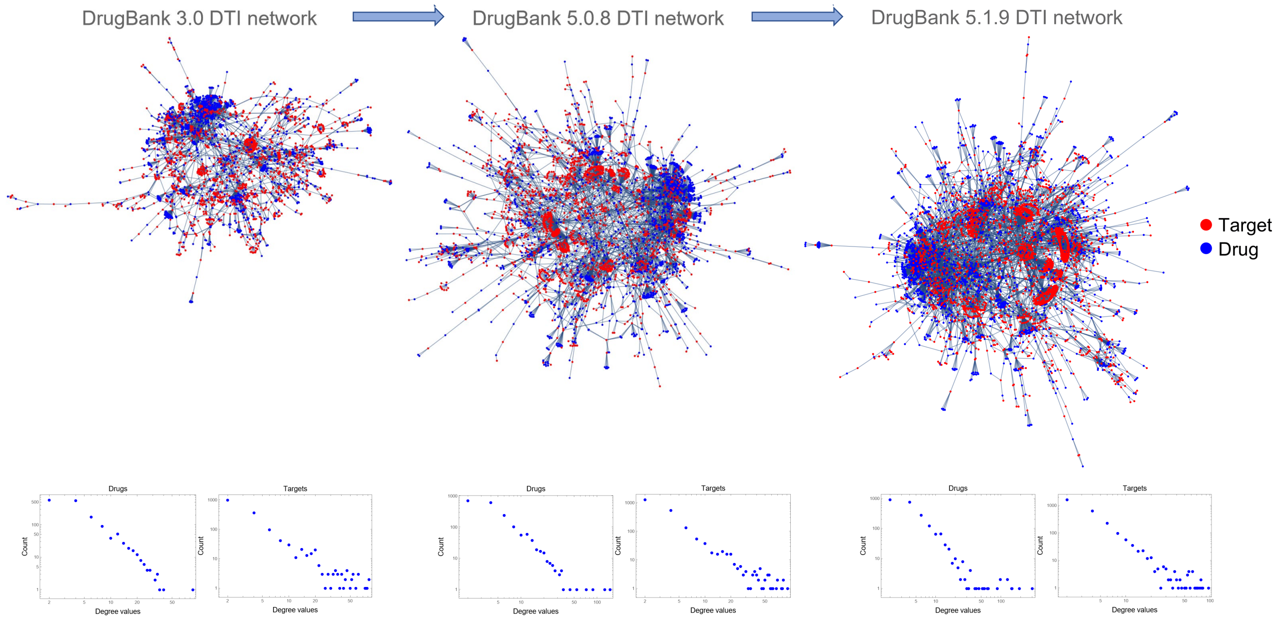

Figure 2. The evolution of drug-target interaction (DTI) bipartite networks with the successive DrugBank data versions, where the red nodes represent biological targets, the blue nodes represent drugs, and the links represent drug-target interactions. Although the DTI network sizes increase with the successive DrugBank data, the network structure and the degree distributions in both drugs and targets (see the panels below) are not altered substantially.

between nodes v_i and v_j. In unweighted networks, $w_{ij} \in \{0, 1\}$, and in weighted networks $w_{ij} \in \mathbb{R}$; furthermore, in undirected networks, elements in the adjacency matrix are symmetric relative to the first diagonal, i.e., $w_{ij} = w_{ji}$.

The *multi-layered* networks generalize the network concept by considering multiple types of edges/links so that the network can be described by several adjacency matrices W^q $(q \in \{1, 2, \ldots Q\})$, with elements w_{ij}^q for each of the Q types of links. Moreover, when acknowledging that the link structure (i.e., the network topology) changes over time t, the network is expressed as a function of time, $w_{ij}^q(t)$.

Drug networks

To mine the complexity of drug interactions in the biological environment, researchers use database information to build drug networks such as drug-drug, drug-target, and drug-disease interaction networks, as well as drug-disease and drug–adverse effects association networks. This paper considers drug-drug interaction networks (complex drug networks where nodes represent drugs) and drug-target interaction networks (bipartite networks where one type of node represents drugs, and the other represents biological targets). Links represent drug-drug and drug-target interactions in the DDI and DTI networks. We do not consider drug-drug

interaction type and strength. Consequently, the DDI networks are undirected, unweighted, and monopartite. Also, the drug-target interactions have no information regarding their strength, making DTI networks directed (from drug to target), unweighted, and bipartite.

Using the complex network formalism, in DDI networks, $\forall i, j, i \neq j$, $v_i \in V = \mathcal{D}$ and $e_{ij} \in E = \mathcal{I}\,(e_{ij} \neq 0)$, where $\mathcal{D}$ is the set of drugs and $\mathcal{I}$ the set of drug-drug interactions. In DTI networks, we have $v_i \in V = V_{\mathcal{D}} \cup V_{\mathcal{T}}$ (where $V_{\mathcal{D}}$ is the set of nodes representing drugs and $V_{\mathcal{T}}$ is the set of nodes representing targets) and $e_{ij} \in E = \mathcal{A}$, where $\mathcal{A} = \left\{e_{ij} | v_i \in V_{\mathcal{D}}, v_j \in V_{\mathcal{T}}, i \neq j, e_{ij} \neq 0\right\}$ represents the set of drug-target interactions). Additionally, the set of non-edges in the DDI networks is $\bar{\mathcal{I}} = \left\{e_{ij} | v_i, v_j \in \mathcal{D}, i \neq j, e_{ij} = 0\right\}$, and in the DTI is $\bar{\mathcal{A}} = \left\{e_{ij} | v_i \in \mathcal{D}, v_j \in \mathcal{T}, i \neq j, e_{ij} = 0\right\}$.

A non-edge in a DDI network ($\bar{e}_{ij} \in \bar{\mathcal{I}}$) or a DTI network ($\in \bar{\mathcal{A}}$) represents a drug-drug or a drug-target interaction that has a probability $p \in (0, 1)$ of being discovered in the future (i.e., missing information generating uncertainty). If $p = 0$, we are sure that interaction between v_i and v_j is impossible (i.e., the negative information on drug interactions).

The DDI and DTI networks are analyzed and processed with computational tools to acquire new insight and knowledge in areas such as drug repurposing, drug-drug interaction prediction, drug-target interaction prediction, designing synergistic drug combinations, and optimizing drug efficacy.

As this paper considers the main types of drug networks, namely drug-drug and drug-target interaction networks (or interactomes), we acknowledge two main issues:

i. The networks are built according to what we know (in terms of drug-drug or drug-target interactions) at the time when the database is recorded. Over time, some new information may be added, and some may be filtered according to the experimental biological findings.

ii. The uncertainty in building the drug networks, mainly represented by missing links between nodes—drug-drug or drug-target interactions that exist but are still uncovered—may affect the conclusions of drug network analysis methods. Previous research also expresses this important concern [27].

Accordingly, our investigation is set to answer two fundamental questions.

1. We already know the main topological metrics statistics in complex networks (biological, social, or technological) where the degree of uncertainty is smaller than in drug networks [46]. Thus, how do the drug network parameters evolve with the drug database versions compared to the typical complex network topological characteristics, such that they foster an effective and efficient computational complex network analysis?

2. Newer drug databases contain more information on both drug-drug and drug-target interactions. How did the robustness of the drug networks evolve after adding more knowledge?

To address question 1 we analyze the evolution over time of drug-drug and drug-target networks $w_{ij}(t)$ (where t are discrete-time moments when the drug database version was released) for parameters such as average degree, diameter, average path length, and average clustering coefficient. We analyze the same type of evolution for the distributions of centrality metrics such as degree, betweenness, closeness, eccentricity, and page rank.

To answer question 2, we implement a robustness test algorithm that adds/injects links representing potential unknown interactions to the known network, $w_{ij}(t) + u_{ij}$ (where u_{ij} represents the unknown links), and then represent the evolution of the ordinal correlation (Kendall τ) between the node rankings in $w_{ij}(t)$ and $w_{ij}(t) + u_{ij}$ with the rate of unknown links (expressed as the number of nodes in u_{ij} divided by the number of nodes in $w_{ij}(t)$). The rationale for our approach is that the analysis of node centrality rankings underpins most network-based bioinformatics methods and applications.

Methods

In this section, we describe the statistical and computational methods employed in this paper: subsection Network analysis introduces the main metrics and centralities required for the computational and statistical analysis of complex drug networks; subsection Database quality presents the statistical methods we use to test the drug database integrity; subsection Network analysis robustness explains the computer simulation method we propose to assess the robustness of the centrality-based drug network analysis strategies; subsection Computational methods describes the tools we use in this paper to implement the required computational and statistical techniques.

Network analysis

Network parameters and metrics

The *degree* of a node $v_i \in V$ in an undirected network G is defined as $d(v_i) = \sum w_{ij}$. In directed networks, we can compute the in-degree and out-degree of v_i as $d^I(v_i) = \sum w_{ij}$ and $d^O(v_i) = \sum w_{ji}$, respectively. Then, the average degree of network $G = (V, E)$ is

$$\langle d \rangle = \frac{1}{|V|} \sum_{v_i \in V} d(v_i) \tag{1}$$

where $|V|$ is the number of elements in V, namely the number of nodes in network G. If G is directed, we can compute the average in-degree and out-degree as $\langle d^I \rangle = \frac{1}{|V|} \sum_{v_i \in V} d^I(v_i)$ and $\langle d^O \rangle = \frac{1}{|V|} \sum_{v_i \in V} d^O(v_i)$. When the network G is multipartite, we can also compute the average degree for each node type V_j ($V_j \subset V$) as $\langle d_{V_j} \rangle = \frac{1}{|V_j|} \sum_{v_i \in V_j} d(v_i)$.

The *clustering coefficient* of a node v_i is the number of existing links between nodes directly connected to v_i divided by the total number of possible links,

$$c(v_i) = \frac{2\left|\left\{e_{jk} | j, k \in L_i\right\}\right|}{|L_i|\,(|L_i| - 1)}, \tag{2}$$

with L_i representing the set of nodes directly linked to v_i. The average clustering coefficient of network G is

$$\langle c \rangle = \frac{1}{|V|} \sum_{v_i \in V} c(v_i). \tag{3}$$

The *network density* is the ratio between the number of links/edges in G (i.e., $|E|$) and the maximum number of possible links,

$$r = \frac{2|E|}{|V|\,(|V| - 1)}. \tag{4}$$

The network G is a *connected graph* if there is a path between any two nodes $v_i, v_j \in V$; otherwise, G has multiple components (a component is a connected subgraph). In general, there can be many paths between two nodes v_i and v_j in a connected network or component; we denote the length of the shortest one $s(v_i, v_j)$. Then,

the average path length in G is

$$\langle s \rangle = \frac{2}{|V|\,(|V|-1)} \sum_{v_i, v_j \in V} s(v_i, v_j). \tag{5}$$

The *diameter* of a network G is the biggest shortest path between any two nodes $v_i, v_j \in V$,

$$\phi = \max_{v_i, v_j \in V} \left\{ s(v_i, v_j) \right\}. \tag{6}$$

Network centralities

A *node centrality* $\mathcal{C}$ is an attribute or metric that characterizes the importance of a node in the network; many studies in biological networks use centralities to rank nodes. The simplest centrality of node v_i is the *degree* $d\,(v_i)$, as the number of links (or the weight) associated with the node indicates its importance [48, 49, 50].

The *betweenness* centrality characterizes the node's role in connecting communities or clusters of nodes. (Such node clusters are often associated with specific functionality in biological networks, particularly drug networks [51, 52, 35, 25].) The betweenness of v_i is the number of paths (shortest or random walks) between all node pairs in G that cross v_i (normalized by the total number of node pairs in G),

$$b\,(v_i) = \sum_{v_j, v_k \in V;\, i,j \neq k} \frac{2\sigma_{j,k}\,(v_i)}{|V|\,(|V|-1)} \tag{7}$$

where

$$\sigma_{j,k}\,(v_i) = \begin{cases} 1 & \text{if } \exists\, s(v_j, v_k) \text{ that crosses } v_i \\ 0 & \text{otherwise}. \end{cases} \tag{8}$$

The *closeness* centrality measures how close the node v_i is to the other nodes; it is the inverse of the sum of shortest paths to all other nodes in V,

$$\gamma\,(v_i) = \left(\sum_{v_j \in V \setminus \{v_i\}} s(v_i, v_j) \right)^{-1}. \tag{9}$$

The *eigenvector* centrality assumes that the degree of a node v_i does not particularly determine its importance; instead, the importance of nodes directly connected to v_i is key. For node v_i, the eigenvector centrality value $\eta\,(v_i)$ is defined as

$$\eta\,(v_i) = \frac{1}{\lambda} \sum_{v_j \in L_i} \eta(v_j) = \frac{1}{\lambda} \sum_{v_j \in V} \eta(v_j) w_{ij} \tag{10}$$

where λ is a constant, w_{ij} is an element in the adjacency matrix W, and $\eta\,(v_i)$ is the eigenvector centrality value of nodes v_j directly connected to v_i. Because the definition in equation 10 is recursive, finding the eigenvector centrality values for all nodes in V requires solving the equation $W\eta = \lambda\eta$ using linear algebra [53]. (W is the adjacency matrix, and η is the vector of eigenvector centrality values for all nodes in V.)

Typical complex network characteristics

Generally, in real-world complex networks, the density of links is low (i.e., $\sim \frac{1}{n}$ because the number of links is of the order n, where n is the number of nodes, and far from the order n^2 corresponding to a completely connected network); the total number of links also determines the average degree, and the average degree is significantly lower than n; the degree follows either a power-law (exponent is between 1 and 3) or normal distribution; the average path length is between 3 and 10; the clustering coefficient is larger than $\frac{1}{n}$ but considerably lower than 1 [46, 54].

Database quality

The drug-drug and drug-target data quality in databases such as DrugBank are degraded by uncertainty, as they contain curated information from published studies, papers, and clinical trials. The ever-growing volume of empirical results that underpin drug databases can be affected by errors generated by improper data handling, academic misconduct, or imbalanced research focus (e.g., one may expect an abundance of new data on SARS-CoV-2 target data, but less so for rare diseases). These problems related to data quality in drug databases are adequately acknowledged in the literature, along with the systematic counter-measures [27, 55]. One such analytical approach to validate data quality is checking various parameter distributions against Benford's law [55]. The law of the first digit—or Benford's law—states that the natural distribution of the first digit f of a real-world variable (covering a wide range of values) is

$$P\,(f) = \log_{10}\left(1 + \frac{1}{f}\right), \tag{11}$$

where $f \in \{1, 2, \ldots 9\}$. This means that the probability of first digit being '1' is the highest, with the probabilities of the folowing digits probabilities decreasing logarithmically.

In this paper, we check Benford's law for the network centralities to verify data quality, meaning that we consider f the first digit of network centralities $\mathcal{C}$. For complex network centralities, a similar approach is described in [56], in the case of social networks. To the best of our knowledge, no similar study was performed in biological networks, although we find proven applications of Benford's law in systems biology [57, 58].

Network analysis robustness

The drug-drug and drug-target interactions we take from DrugBank are well documented and proven by experiment, but we can assume some unknown interactions are yet to be investigated or tested. To analyze the robustness of network analysis tools, we draw inspiration from the paper [27], where the authors added to the drug-target network generated with DrugBank new drug-target interactions (i.e., unaccounted by DrugBank) reported in another database; this way, they analyze how the centrality distributions change with the added information. In this paper, we adopt a systematic approach to study the robustness of both the network structure and the specific centralities by randomly adding unknown links (i.e., drug-drug or drug-target interactions) with a rate q, and then analyze the structural changes that manifest through modifications in the node rankings. We measure the structural changes incurred by adding the previously unknown links in two ways. First, we target the centralities with a power-law distribution (e.g., degree, betweenness) to compute the difference between the log-log distribution slopes α. Second, we compute the Kendall τ correlation between the node rankings according to centrality $\mathcal{C}$ before and after adding the unknown links ($\tau = 1$ indicates a perfect monotonous correlation, $\tau = 0$ indicates no monotonous correlation).

By representing τ as a function of q (with q going from a very small value to 0.1), we can analyze the robustness of the network structure: rapid decay of τ as q increases indicates that the node ranking according to $\mathcal{C}$ is fragile to the uncertainty of new links being present; conversely, slow decay of τ suggests a robust node ranking. We present the algorithmic description of our robustness test in Algorithm 1, where E_t represents the set of all possible edges in G and E_u is the set of previously unknown edges (accordingly, $E_t \setminus E$ is the set of non-existing edges in G). As this is a random

Algorithm 1 Analyze the variation of Kendall τ between node rankings and power-law distribution slope α (for node centrality $\mathcal{C}$) in network G and network with unknown edges G'.

Input: Network $G = (V, E)$.
Output: Representation of Kendall τ and power-law distribution slope α against unknown edge rate q.

$E_t \Leftarrow \left\{e_{ij} = (v_i, v_j) | v_i, v_j \in V; i < j\right\}$
for r in 0 to R **do**
  for q in range (0.001 to 0.1) **do**
    $E_u \Leftarrow \emptyset$
    for all $e_{ij} \in E_t \setminus E$ **do**
      $E_u \Leftarrow E_u \cup \left\{e_{ij}\right\}$ with probability q
    end for
    $E' \Leftarrow E \cup E_u$
    $G' \Leftarrow (V, E')$
    $l_V \Leftarrow$ list of nodes in V, descending order after $\mathcal{C}$
    $l'_V \Leftarrow$ list of nodes in V', descending order after $\mathcal{C}$
    $\tau_q \Leftarrow$ Kendall $\tau\ (l_V, l'_V)$
    $\alpha_q \Leftarrow$ Power-law distribution slope of $\mathcal{C}$ in G'
  end for
end for=0

simulation, we repeat it for each G and q (R times in Algorithm 1) to compute the average and variance for α and Kendall τ. (For the simulations presented in this manuscript, we used $R = 100$.)

Computational methods

To foster the reproducibility of our analysis, we provide all the necessary tools—Jupyter Notebook, Python, and WolframScript—as buildable Docker containers in the `https://github.com/research-hyperion/Drug_database_statistics` repository. We automatically install all the tools needed for reproducing our methods in a Linux container and provide a shell script for automatically running the analysis. The activation of WolframScript for running the Mathematica notebooks requires a Wolfram account. DrugBank versions recorded as XML files must be downloaded, as mentioned in Section Data description, in the root of the cloned repository in the DrugBank directory. Each XML file must be saved following a naming convention to avoid file overwriting; therefore, each DrugBank XML file is saved as *drugbank_version.xml* in the DrugBank directory.

To create the Docker image run the command **docker build –build-arg wolframId=<wolfram account email> –build-arg wolframPass=<wolfram account password> -t hyperion**. The image will take several GB of storage. The *wolframId* and the the *wolframPass* are the Wolfram account credentials needed to activate the WolframScript. After creating the container, run the command **docker run -t -i hyperion /bin/bash** in the terminal window to start an interactive shell in the Docker container.

We created a shell script that allows the execution of the Jupyter notebooks that build the DDI and the DTI networks and the network robustness analysis for each DrugBank version. As presented in Section DTI network robustness, due to the high complexity of the robustness analysis and huge computational burden, the script must provide the options to build and analyze the DDI and DTI networks or to perform the network robustness analysis. Invoking the script with the "-i" parameter will build and analyze DDI and DTI networks for each DrugBank version; invoking with the "-s" parameter will build the networks and run the robustness analysis.

The DDI and DTI network analyses are performed by 3 Jupyter Notebooks, namely *parse_DrugBank*, *parse_DrugBank-DDI*, and *parse_DrugBank-DTI*; if run in the mentioned order, we parse the DrugBank XML files, build and analyze the DDI and DTI networks. The robustness analysis depends on the files created by the mentioned notebooks to perform the algorithmic analysis presented in Section Network analysis robustness.

Results

This section analyzes two types of networks built from DrugBank data: drug-drug interaction networks DDI (where nodes v_i represent drugs and links represent drug-drug interactions) and drug-target networks DTI (nodes represent both drugs and targets and links represent drug-target interactions).

Many papers that employ complex network science for drug repurposing, drug interaction prediction, or adverse effect prognosis use other, more sophisticated drug networks [12, 13, 1, 33, 59](multi-partite networks, drug similarity networks, etc.) However, the straightforward DDI and DTI topologies are representative of the complexity issues at hand, and most of the more elaborated networks (such as the weighted similarity networks) can be derived from DDI and DTI structures through multi-partite network projection [60].

Network metrics and centrality analysis

Our analysis follows the evolution of metrics and centralities in DDI and DTI networks built with DrugBank information—from version 3.0 to version 5.1.8 (January 2011–January 2021).

In Figure 3, panel a, we present the evolution of the number of nodes (i.e., drugs) in DDI networks; in Figure 3, panel b, we show the evolution of the number of nodes, drugs, and targets in DTI networks. As shown, from version 3.0 to 5.1.9, the number of medicines/drugs roughly quadrupled; in contrast, the number of targets increased at a much lower rate. Such an evolution suggests that, over the years, the complexity of processing and analyzing drug interaction networks grew considerably. In both DDI and DTI networks, the number of drugs and targets is not the same as the total number of drugs and targets in the respective DrugBank versions because some drugs and targets have no known interactions.

We also notice the significant discrepancy between the number of links in DDI and DTI networks (Figure 4 (panels a and b, respectively), which determine the evolution of DDI and DTI density evolution in Figure 4, panels c and d.

Comparing the average path length and diameter in the DDI and DTI networks further emphasizes the observed discrepancies (see Figure 5 a and b); the same remark holds for the average degree in Figure 6 (panels a and b for DDI and DTI, respectively).

The DTI networks are bipartite; therefore, any link/edge connects one drug with one target. Consequently, DTI networks have a clustering coefficient of 0. Figure 7 presents the evolution of the average clustering coefficient $\langle c \rangle$ in DDI networks.

Like many other natural complex networks [46, 61], the DDI and DTI networks are scale-free, meaning that their node degree distribution is a power-law $P(d) \propto d^{-\alpha}$. Figure 8 presents the evolution of node degree's power-law distribution exponent α in DDI and DTI networks. (DTI is a bipartite directed network, and we also present the in-degree and out-degree distributions for targets and drugs, respectively.)

The analytical results presented in this section indicate that the DDI networks have become highly dense ($r = 0.1238$ with a huge $\langle d \rangle = 530.8455$ for DrugBank 5.1.8), with small $\langle s \rangle$ and ϕ and an unusually large α. Although the clustering coefficient $\langle c \rangle$ is high, detecting node communities or ranking nodes with centralities—standard network analysis techniques in network pharmacology [12, 14, 25]—becomes irrelevant because of the high link density. Conversely, the metrics and centrality statistics of the DTI networks are typical for biological scale-free complex networks [46, 54]: small $\langle s \rangle$ (but not smaller than 6), and an exponent α around 3. Therefore, the DTI network topologies foster the employment of

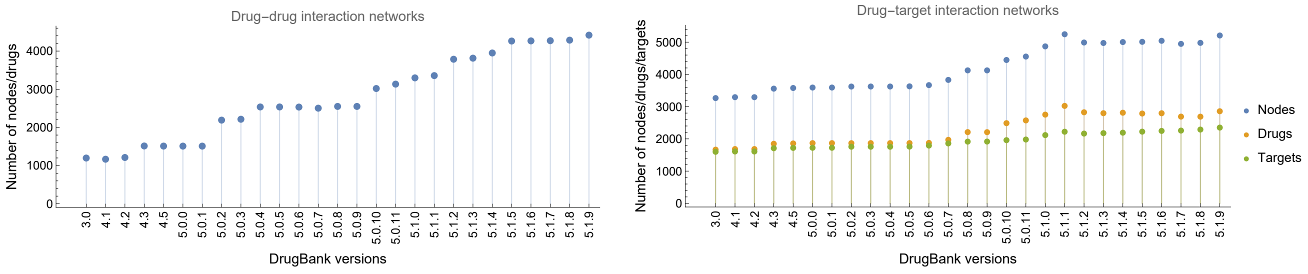

a) b)

Figure 3. The evolution of nodes $|V|$ in drug-drug (representing drugs $|V_{\mathcal{D}}|$, in panel a) and drug-target interaction networks (representing drugs $|V_{\mathcal{D}}|$ and targets $|V_{\mathcal{T}}|$, in panel b) built with information from DrugBank versions 3.0 to 5.1.9. The number of drugs in the DDI evolves from 1198 for version 3.0 to 4417 for version 5.1.9; the increase is steady, although some DrugBank versions abruptly put on more drugs so that several ensuing versions filter the added information. The number of drugs and targets in the DTI evolves respectively from 1166 and 1599 in version 3.0 to 2857 and 2350 in version 5.1.9; because the DTI network is bipartite, the number of DTI nodes is the sum of drugs and targets.

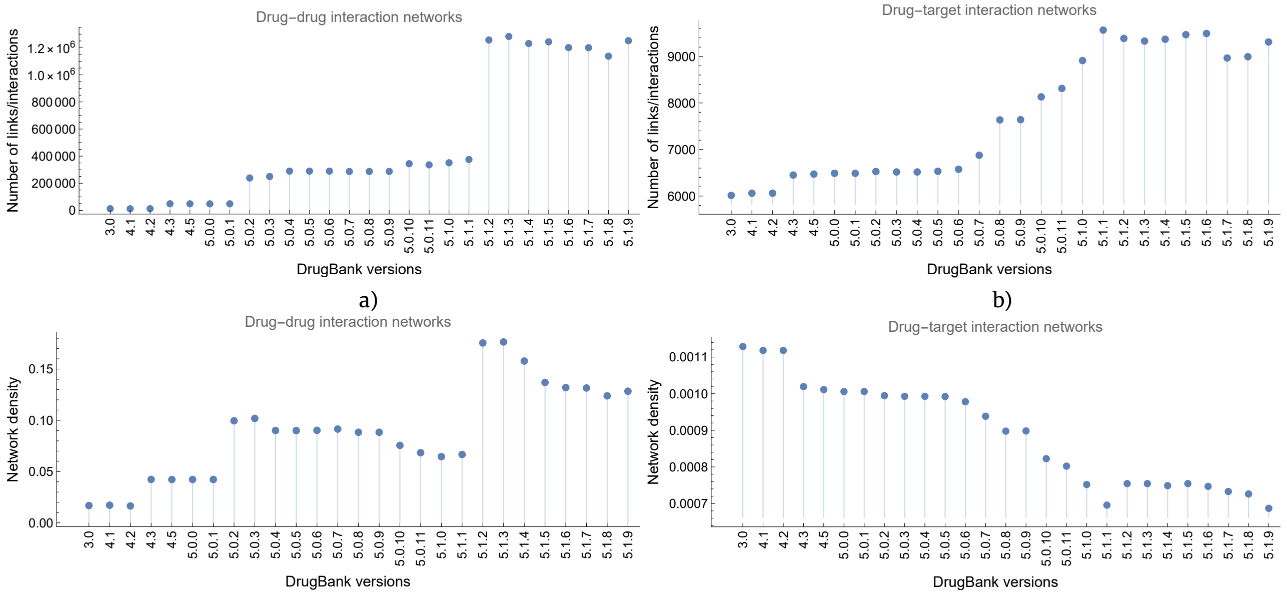

Figure 4. The number of links $|E|$ evolution in drug-drug and drug-target interaction networks, built with information from DrugBank versions 3.0 to 5.1.9 (panels a and b, respectively). The number of links in the DDI evolves from 12,089 in version 3.0 to 1,252,028 in version 5.1.9; in the DTI, it increases from 6,015 to 9,310. Panels c and d respectively present the evolution of density *r* in drug-drug and drug-target interaction networks, built with information from DrugBank versions 3.0 to 5.1.9. The density in the DDI networks evolves from 0.0186 in version 3.0 to 0.128377 in version 5.1.9; some versions abruptly increase the density (by adding many interactions), while the following versions filter the interactions and decrease the density (e.g., the density evolution from 5.1.1 to 5.1.9). In the DTI networks, the density evolves from 0.0011 in version 3.0 to 0.000687 in version 5.1.9; as shown, the density in DTI networks decreases with the newer database versions.

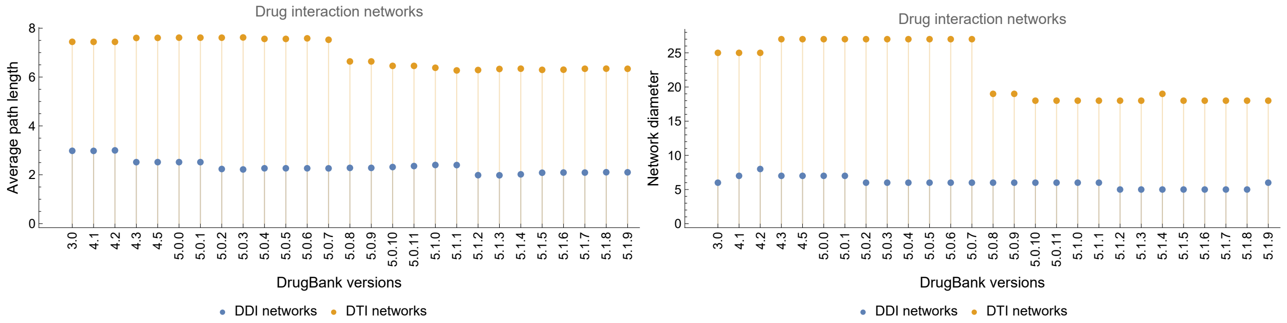

a) b)

Figure 5. The evolution of average path length $\langle s \rangle$ (panel a) and diameter ϕ (panel b) in drug-drug and drug-target interaction networks, built with information from DrugBank versions 3.0 to 5.1.9. The value of $\langle s \rangle$ evolves from 2.98 in version 3.0 to 2.1 in version 5.1.9. for DDI, and from 7.44 to 6.338 in DTI. The value of ϕ evolves from 6 in version 3.0 to 6 in version 5.1.9. for DDI, and from 25 to 18 in DTI.

community detection and other specific network analysis techniques.

Moreover, upon visual inspection, Figure 8 indicates that as the DDI networks become denser across the DrugBank versions, their degree exponent α becomes > 4, i.e., too big to correspond to real-world power-law distributions [46]. Indeed, the visual representa-

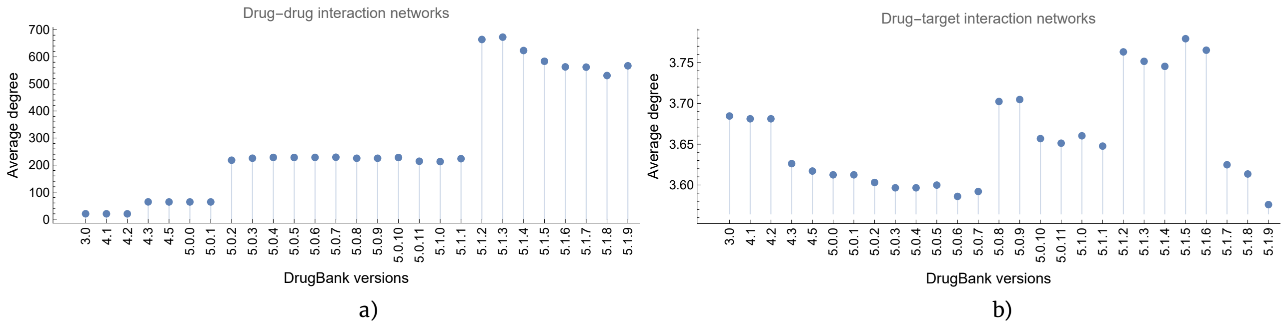

Figure 6. The evolution of average degree $\langle d \rangle$ in drug-drug and drug-target interaction networks (panels a and b, respectively), built with information from DrugBank versions 3.0 to 5.1.9. For DDI networks, the value of $\langle s \rangle$ increases from 20.181 in version 3.0 to 566.913 in version 5.1.9; for DTI networks, it oscillates from 3.684 to 3.576.

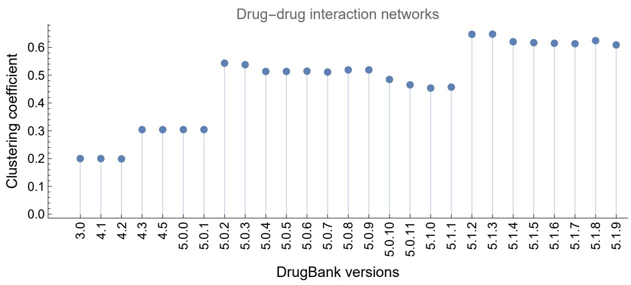

Figure 7. The evolution of average clustering coefficient $\langle c \rangle$ in drug-drug interaction networks, built with information from DrugBank versions 3.0 to 5.1.9. The value of $\langle c \rangle$ increases from 0.199 in version 3.0 to 0.609 in version 5.1.9.

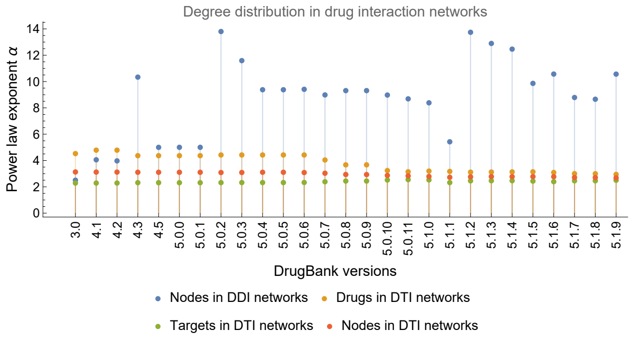

Figure 8. The evolution of the α exponent of the power-law degree distribution in drug-drug and drug-target interaction networks, built with information from DrugBank versions 3.0 to 5.1.9. In DDI networks, the α values are very high—specific for dense networks. In DTI networks, we present the overall degree distribution of the nodes and the in-degree and out-degree distributions, as the DTI network is bipartite and directed. (The connected nodes representing drugs have non-zero out-degrees and zero in-degrees, whereas nodes representing targets have zero out-degrees and non-zero in-degrees.)

tion of the degree and betweenness distributions in the DrugBank 3.0 DDI network show typical power-laws (Figure 9, panels a and c), whereas the DrugBank 5.1.9 counterparts indicate that only the betweenness distribution is not substantially altered (Figure 9, panels b and d).

We observe the same degradation for the other centrality distributions in DDI networks from the earlier versions of the drug database to the latest ones. In Figure 10 panels a, b, and c, we present the eigenvector, PageRank, and closeness distributions in the DrugBank 3.0 DDI network; in Figure 10 panels d, e, f, we visualize these distributions in the DrugBank 5.1.9 DDI network.

Conversely, in the DTI networks, we do not notice the same degradation of power-law degree and betweenness distributions from DrugBank 3.0 to 5.1.9 (see Figure 11). We see the same tendency not to alter the distributions in DTI networks across all DrugBank versions for eigenvector, PageRank, and closeness centralities (as presented in Figure 12).

Network centralities and Benford's law

We check if the distributions of centralities abide by Benford's law in drug-drug and drug-target networks. When data distribution in natural occurring datasets closely resembles the Benford distribution, we assume a high drug interaction data quality, generating robust analysis results.

We use Pearson's chi-squared (χ^2) test to measure the distance between degree and betweenness centrality distributions and the theoretical Benford distribution in DDI networks across all DrugBank versions, as indicated in [56]. However, as argued in [63], Pearson's χ^2 is often misused in such analysis cases; therefore, as suggested in this reference, we also use the Wasserstein distance and the sum of squared deviations between distributions. For these three metrics, a smaller distance to Benford's distribution means that the empirical centrality distribution in the drug network is more compliant with Benford's law of the first digit. Apart from the numerical analysis, we also used graphical representations of the distributions, including Q-Q plots, to check if the degree and betweenness distributions in DDI networks abide by Benford's law.

DDI networks

We notice by the visual inspection of Figures 13 and 14, that the compliance with Benford's law has degraded over the years (i.e., with the evolution of DrugBank versions) in DDI networks, especially for the degree distribution. Using the Wasserstein distance, the sum of squared deviations, and Pearson's χ^2, we show the evolution of distance between the theoretical Benford distribution and the empirical distribution of degree (Figure 15, panels a and b) and betweenness centrality (Figure 15, panels c and d) distributions in DDI networks across all DrugBank versions. (We represented Pearson's χ^2 separately because its range of values is larger than the other distance metrics.)

DTI networks

For the degree, Figures 16 and 17 offer a visual comparison between the first and the latest DrugBank versions; it shows that the empirical degree is far from Benford's theoretical distribution in all database versions. For the betweenness, Figure 18 presents the comparison between the theoretical Benford first digit distribution and the betweenness in DrugBank 3.0 and 5.1.9 DTI networks.

The overarching conclusion in DTI networks is that the degree is not compliant with Benford's law, but the betweenness is relatively compliant; these properties do not change across the DrugBank versions.

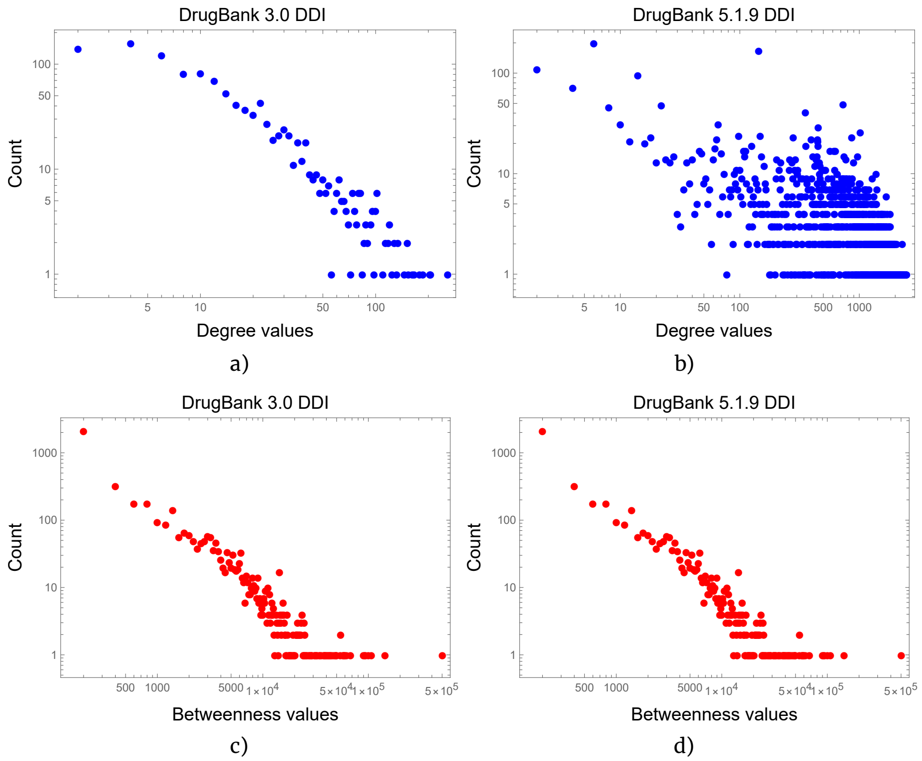

Figure 9. The degree and betweenness distributions in the drug-drug interaction (DDI) networks, built with information from the first and latest DrugBank versions. In panels a and c, we present the degree and betweenness distributions in the DrugBank 3.0 DDI network, whereas panels b and d show the DrugBank 5.1.9 counterparts. (We analyzed and calculated these distributions with the Powerlaw Python package [62].)

DTI network robustness

The algorithmic evaluation of network robustness, described in Section Network analysis robustness, entails running $R = 100$ times the following steps sequentially: adding random edges to the drug network according to a rate of unknown interactions q, determining the degree and betweenness centralities of all nodes in networks G and G' from Algorithm 1, sorting vertices/nodes in G and G' after their centralities, calculating the Kendall τ between the node hierarchies in G and G' after sorting the nodes in the descending order of their centrality values. The algorithmic complexity of adding random edges is $\mathcal{O}(n^2)$, where n represents the number of nodes of the network (the number of added edges is proportional to n^2, and the complexity of generating random numbers is $\mathcal{O}(1)$.) Determining the degree centrality for all network nodes has a complexity of $\mathcal{O}(n^2)$; calculating the betweenness of a node with the Brandes algorithm has a complexity of $\mathcal{O}(nN + n^2 \log n)$ (where N is the number of edges $|E|$ in $G = (V, E)$) [64]; therefore, as many drug networks are dense and $N \sim n^2$, computing the betweenness of all nodes in G entails between $\mathcal{O}(n^4)$ and $\mathcal{O}(n^5)$ complexity. The complexity of sorting the nodes according to their centralities as well as calculating the Kendall τ are between $\mathcal{O}(n \log n)$ and $\mathcal{O}(n^2)$ [65]. In conclusion, performing the algorithmic evaluation of network robustness from Section Network analysis robustness entails a huge computational burden, especially when processing the high-density DDI networks and considering the betweenness centrality.

Assuming the conclusion of the complexity considerations for Algorithm 1, we will focus our robustness study on DTI networks and perform it for the betweenness centrality in the less dense DrugBank 3.0 DTI network. As presented in the simulation results (see Figure 19, panels a and b) of node hierarchy robustness according to the degree centrality, we notice in both DrugBank 3.0 and 5.1.8 DTI networks that the Kendall τ decreases linearly with the unknown edge rate q, and only a slight increase for DrugBank 5.1.8 DTI in comparison with DrugBank 3.0 (Figure 19, panel c). After repeating all simulations 100 times for each q, we noticed a low variability in Figure 19, panels a and b.

As explained, due to algorithmic complexity reasons, we perform the betweenness centrality robustness test in Algorithm 1 for the DrugBank 3.0 DTI; the corresponding simulation results in Figure 19, panel d, reveal a logarithmic decrease with q, but a high variability of τ.

Because the analysis of the degree distribution under different rates of unknown interactions q is not computationally prohibitive in terms of complexity, according to Algorithm 1, we analyze the power-law distribution parameters in drug-target interaction networks (DTI) for DrugBank 3.0 and 5.1.8. We choose to focus our analysis on DTI—instead of DDI—because the DDI networks are very dense already, and adding unknown interactions does not have a significant impact. Figure 20 comparatively presents the evolution of the power-law degree distribution exponent α with the rate of unknown interactions q in DrugBank 3.0 (panel a) and DrugBank 5.1.8 (panel b) DTI networks.

Discussion

Our discussions mainly stress the drug datasets' vulnerable aspects, as revealed by the analysis results, specifically those related to the DDI and DTI networks.

Discussions on DDI networks

The first finding of our analysis is that the DDI networks built with data from the latest DrugBank versions deviate significantly from the typical complex network parameters and centrality distributions. Such a situation is generated by the high network density, owing to the colossal number of reported interactions. (The latest DDI network has a massive average degree of 650 and a very small average path length of around 2). The problematic situation of the very dense DDI networks calls for addressing the following issues.

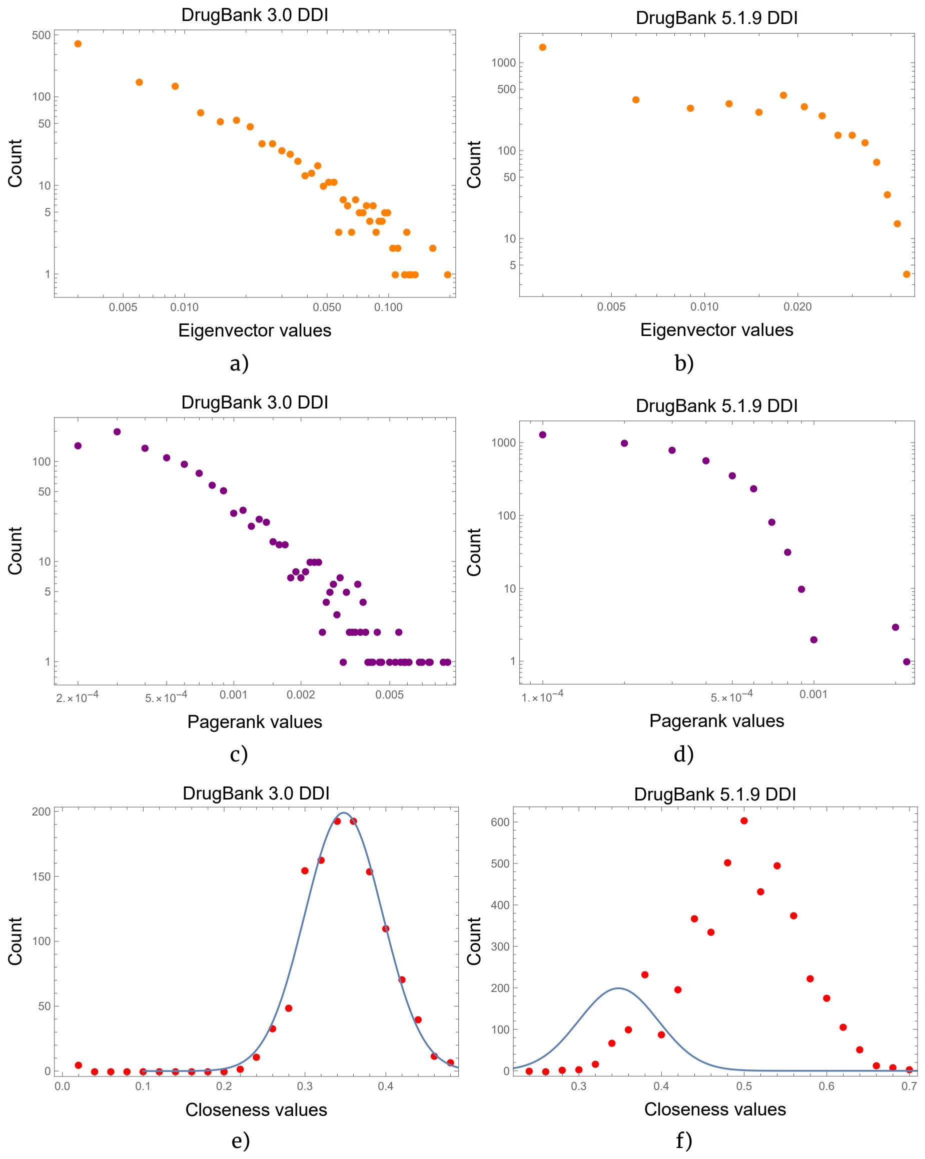

Figure 10. The comparison between eigenvector, PageRank, and closeness distributions in DDI networks built with information from DrugBank 3.0 and 5.1.9. In panels a and b, we show that the power-law distribution of eigenvector centrality from the DrugBank 3.0 DDI degrades in the latest versions; the same can be observed for the PageRank distributions in panels c and d. We also notice that the normal distribution of closeness (a common feature of real-world complex networks, particularly drug interaction networks [35]) in the DrugBank 3.0 DDI network degrades for the DrugBank 5.1.9 DDI network, as shown with the distribution fitting blue line in panels e and f. (We performed the distribution fitting in Mathematica 13.)

Some reliable methods for filtering out the irrelevant DDIs

H. Tilson et al. highlighted that a continuous process is required to select DDIs for the relevant clinical alert and support the clinical decision; physicians and pharmacists need to filter the DDIs and find a way to develop and agree on a standard set of evidence-based DDIs [66]. Indeed, medicines have a proven propensity toward drug-drug interactions in biological environments; therefore, there is a significant probability of interaction between any two drugs. However, only some of these interactions are relevant in specific circumstances.

Specifying rules/norms for recording the DDI severity

Experts promote the development of guidelines for drug-disease and drug-drug interactions in patients with multimorbidity. They suggest populational (i.e., big data) evidence to detect circumstances for relevant interactions [67]. Indeed, the different drug datasets often disagree on the severity level for the same DDI [68].

Establishing the clinical context and interactions' relevance for each drug.

The DDI severity depends on various clinical factors (e.g., a DDI may be major for some specific comorbidity or patient age and moderate/minor otherwise). Circumstances are paramount to analyzing the complexity of clinical practice and identifying potential DDIs and their attributes (i.e., type, severity, and frequency).

Clinicians' perspectives on the potential DDI clinical relevance are essential to avoid alert fatigue—a severe problem affecting the electronic DDI alert systems that support modern healthcare procedures [69]. Consequently, Pirnejad et al. recommend flexibility integrated into DDI clinical decision support systems to personalize potential DDI alerts [70].

Another approach proposes criteria for evaluating/scoring high-priority DDIs that provide clinical decision support alerts in electronic health records (EHR) [71]. E. Kontsioti and collaborators started from the assumption that the literature could be better in creating DDI reference sets or open resources that simultane-

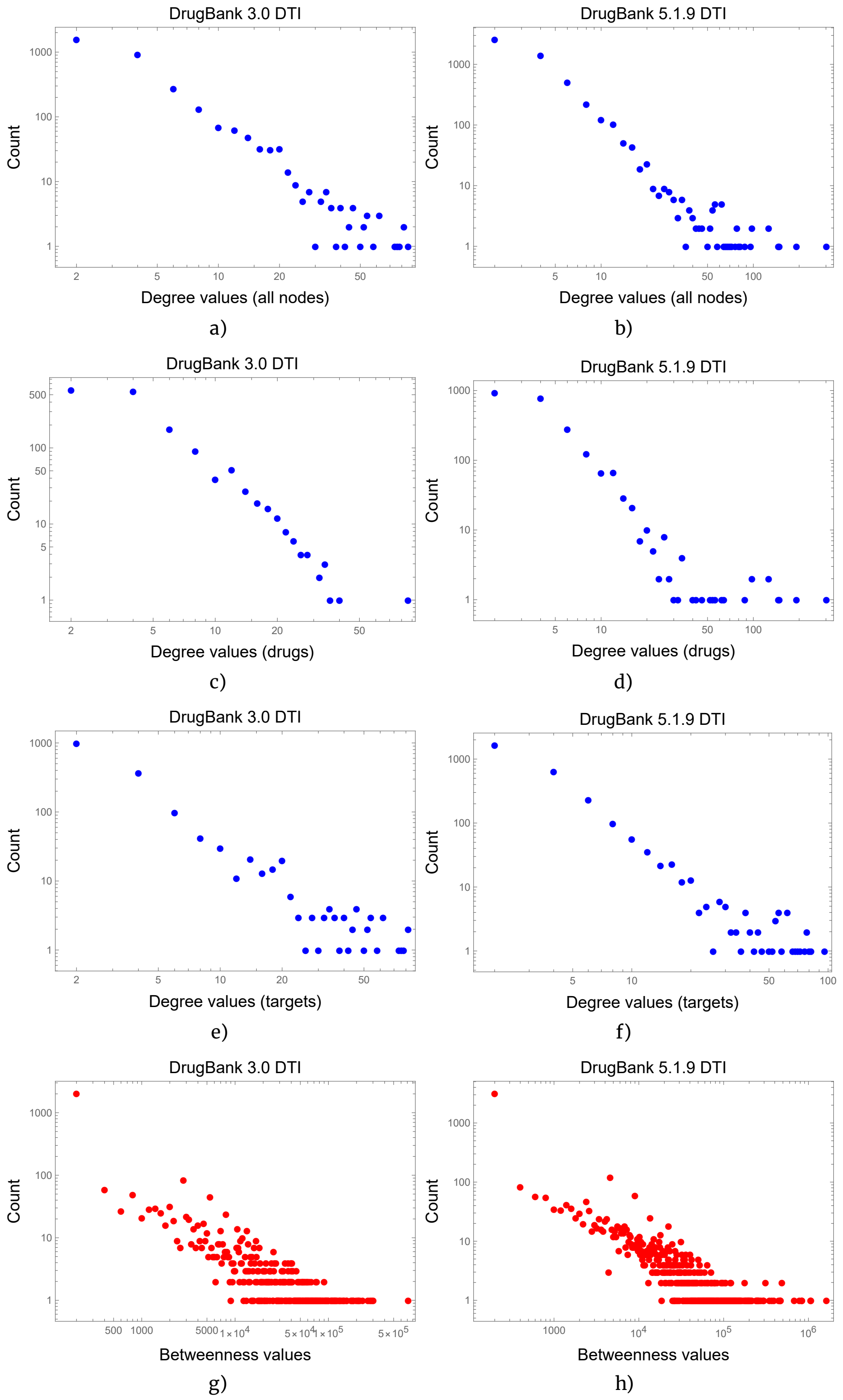

Figure 11. The degree and betweenness distributions in the drug-target interaction (DTI) networks, built with information from the first and latest DrugBank versions. (We analyzed and calculated these distributions with the Powerlaw Python package [62]). In panels a and c, e, and g, we present the degree and betweenness distributions in the DrugBank 3.0 DTI network (we separately display the drug and target, as well as all nodes' degree distributions), while panels b, d, f, and h show the DrugBank 5.1.9 counterparts.)

ously aim at DDIs' clinical relevance and interacting drugs' behavior. They automatically extracted and ensembled data from multiple resources and provided a pipeline for generating a reference set for DDIs that help postmarketing drug surveillance [72].

Nonetheless, the problem is that the drug datasets do not record the clinical context associated with the DDI severity; this may explain why different drug databases report different severity levels for the same DDI—they may assume dissimilar clinical situations.

Building a negative dataset of DDIs with drug pairs known as non-interacting in specific clinical circumstances

To simplify clinical work, A. Assiri and colleagues developed an anti-DDI resource for 200 drugs—a set of drug combinations with

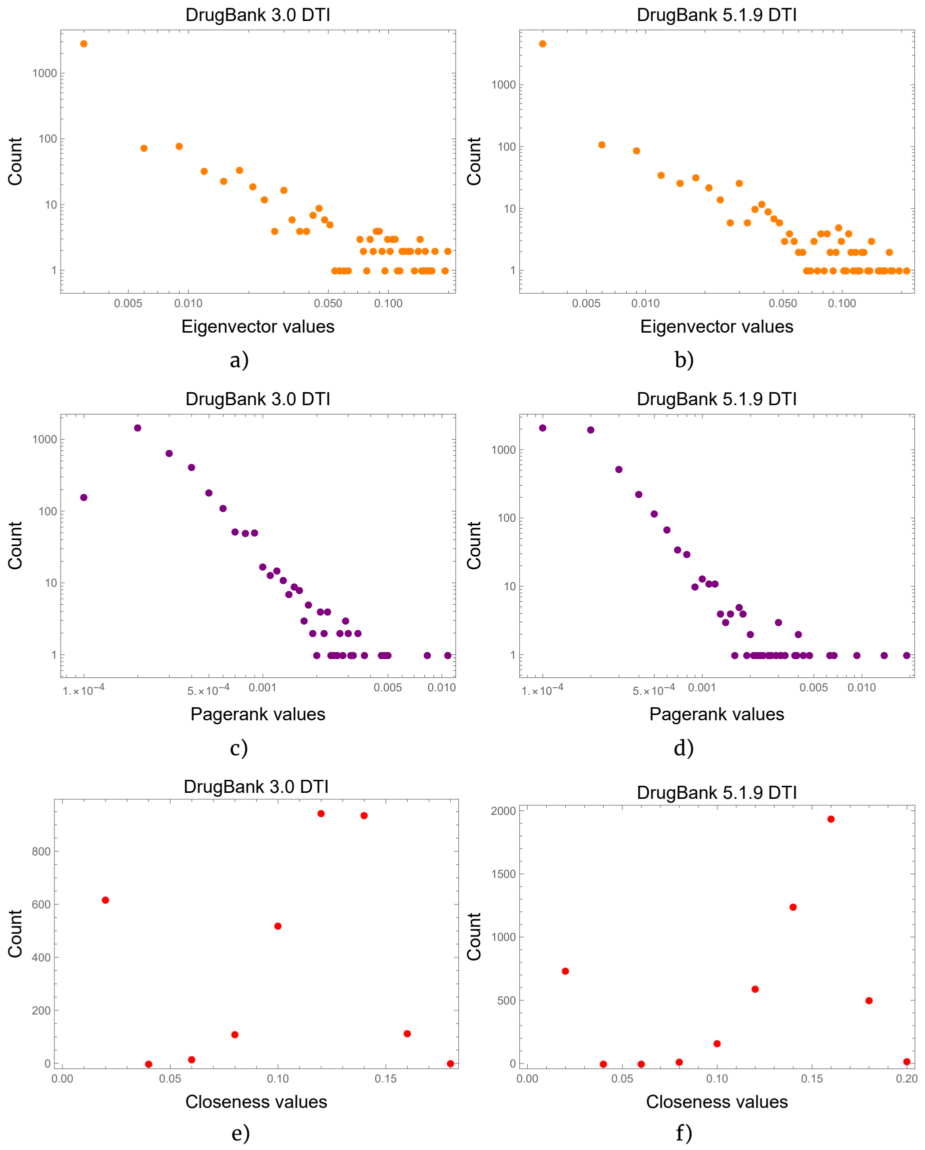

Figure 12. The comparison between eigenvector, PageRank, and closeness distributions in DTI networks with information from DrugBank 3.0 and 5.1.9. In panels a and b, we show that the eigenvector distribution from the DrugBank 3.0 DTI does not degrade in the latest versions; the same can be observed for the PageRank distributions in panels c and d. We also notice the non-regular distribution of closeness in both DrugBank 3.0 DTI and DrugBank 5.1.9 DTI networks in panels e and f. (We performed the distribution fitting in Mathematica 13.)

negative reported interactions (i.e., no risk of DDI) [73].

However, negative DDI information is scarce, and looking for such data means navigating a vast search space; therefore, developing computer-based tools for predicting non-interacting drug pairs would be highly beneficial [74].

Discussions on DTI networks

Our analysis results for DTI networks suggest that the data available across all drug database versions miss much information on drug-target interactions. Also, our simulations indicate that the robustness of centrality-based network analysis methods improves (slightly) with the newer database versions (see Figure 19); however, these latest versions' degree distribution is much more stable (Figure 20). The DTIs do not have the high-density problem of DDI networks and the entailed consequences. The DTI network results indicate the need to address the following issues.

Accurate methods for DTI predictions

We need more accurate drug-target interaction prediction tools, particularly for the DTIs involving new targets (e.g., introduced each new year by the FDA [42]). Such computational methods will help biologists prune the enormous drug-target interaction search space and focus on the most promising and potentially impactful experiments.

Computational DTI prediction methods evolved as a convenient alternative (or complement) to the conventional methods for discovering new drugs, repositioning drugs, or uncovering potential drug side effects. The DTI prediction methods are diverse: multi-molecular networks based on deep walk embedding model [75], knowledge graph embedding (KGE) model-neural factorization machine (NFM) unified framework [76], convolutional neural networks using only data on drug structure and protein sequence [77], heterogeneous network-based methods that integrate various drug data [8], combined computational techniques such as graph embeddings, graph mining, and machine learning [10], or a convolutional neural network method extracting local residue patterns of proteins participating in DTIs [78]. All these computational methods rely on existing (i.e., positive) drug-target interactions data; in many cases, they also use additional relevant data such as drug-drug or

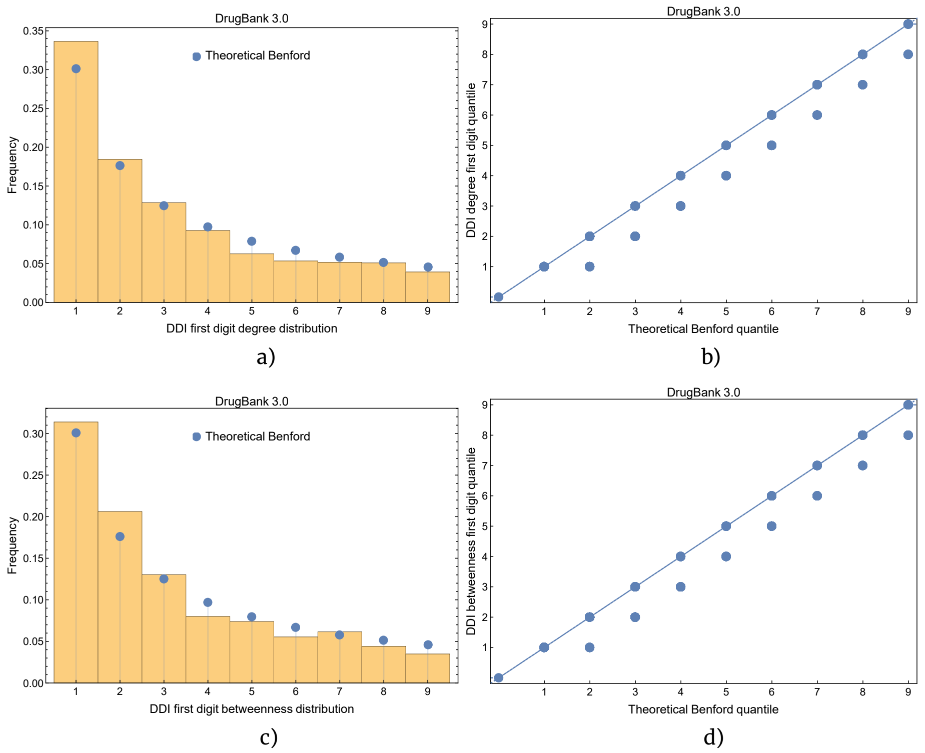

Figure 13. The compliance of degree and betweenness distributions in DDI networks built with DrugBank 3.0 data: a) the comparison between the empirical distribution represented in the histogram and the theoretical Benford distribution represented with blue disks, b) the Q-Q plot where the dashed trendline following the diagonal indicates a small distance to theoretical Benford distribution, c) the comparison between the empirical distribution and the theoretical Benford distribution, d) the Q-Q plot where the dashed also indicates a small distance to theoretical Benford distribution.

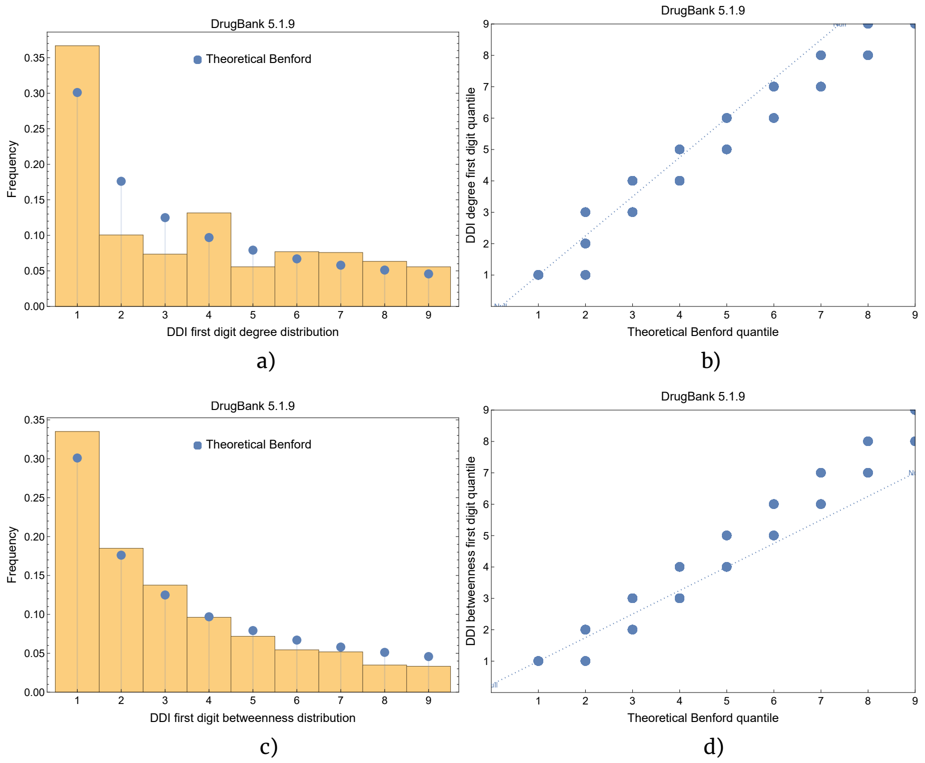

Figure 14. The compliance of degree distribution in DDI networks built with DrugBank 5.1.9 data: a) the comparison between the empirical distribution represented in the histogram and the theoretical Benford distribution represented with blue disks, b) the Q-Q plot where the dashed trendline does not follow the diagonal indicates a significant distance to theoretical Benford distribution, c) the comparison between the empirical distribution and the theoretical Benford distribution, d) the Q-Q plot indicating a close distance to theoretical Benford distribution.

target-target structural similarity [79].

Nevertheless, measuring the accuracy in drug-target interaction prediction (i.e., comparing and ranking the prediction methods correctly) is problematic in the absence of some robust ground truth. Accordingly, recent research proposes a comprehensive benchmark for assessing drug-target interaction prediction methods

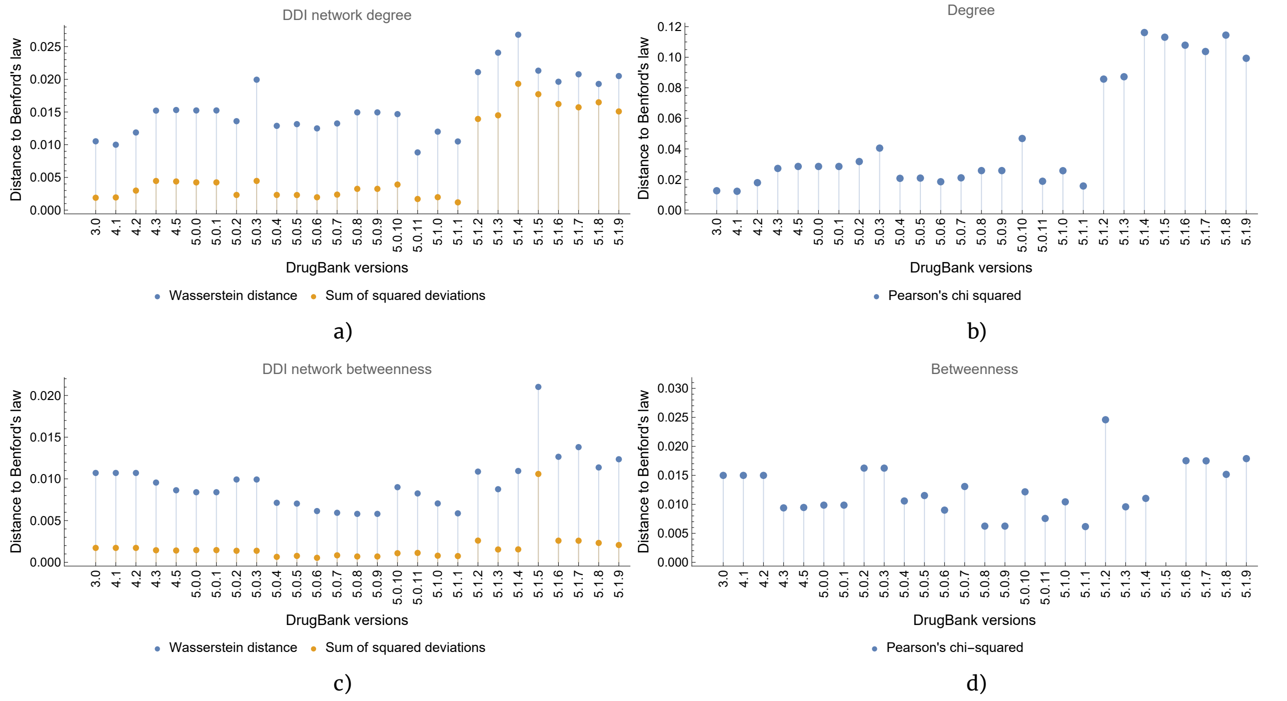

Figure 15. The evolution of distance between the Benford distribution and the empirical degree and betweenness distributions in DDI networks across DrugBank versions: a) Wasserstein distance and sum of squared deviations for the degree centrality, b) Pearson's χ^2 for the degree centrality, c) Wasserstein distance and sum of squared deviations for the betweenness centrality, b) Pearson's χ^2 for the betweenness centrality. Smaller values indicate a stronger compliance with Benford's law of the first digit.

[80], which will allow for their standardized and fair comparison.

Building negative drug-target interaction datasets

Robust DTI prediction requires a better ground truth, including the collections of interactions proven as nonexistent. However, it is impossible to test experimentally all potential DTIs because the search space is too extended.

The efficient training of DTI prediction machine learning models requires positive and negative examples—drug-target pairs experimentally demonstrated as interacting and drug-target couples proven as non-interacting. The DTIs in most drug datasets are robust positive examples because researchers demonstrated them experimentally; however, recording negative drug-target interaction examples is hard to approach [81, 82]. Most drug databases—including DrugBank—contain data supported by experimental research results reported in scientific papers. Such experiments are too expensive to aim at confirming the absence of drug-target interactions. Indeed, the economically reasonable approach is to spend valuable resources to demonstrate existing drug-target interactions because they will lead to new therapies. In this context, a viable approach is prioritizing the potential drug-target interactions by developing computational tools for predicting non-existing DTIs.

Many approaches interpret non-confirmed drug-target interactions as mere non-interactions because the negative DTI information is still scarce. Although frequent, such a practice is misleading since the lack of evidence that something exists does not necessarily mean it does not exist [26].

Potential implications

This paper investigates whether the evolution of drug databases over the last decade has brought—besides the increasing abundance of data—a more accurate and robust analysis of drug interaction networks. We found that the data abundance in the latest DrugBank versions has rendered DDI networks almost impossible to analyze because of their huge density; we also concluded that the DTI networks built with data from the latest database versions only slightly improve the analysis robustness. Fortunately, our investigation also uncovered some database issues that need adjustments (see Section Discussion). Fixing the reported problems will have far-reaching research implications.

First, in the case of drug-drug interaction data (and DDI networks), in our opinion, the field requires a research effort to define standardized labels for interaction severity. The drug-drug interaction severity labels currently used by drug databases—such as, for instance, Drugs.com and DrugBank—are character strings: "major", "moderate", "minor", and "no interactions found". While such labels convey a valuable message to pharmacologists, they are not helpful for statistical analysis or machine learning approaches. Clearly, any statistical or machine learning method must properly quantify the differences between severity labels. By this logic, it is difficult to quantify the difference between character strings "major" and "moderate" in comparison with, say, the difference between "major" and "no interactions found"; yet, from a pharmacological standpoint, the difference is substantial. Consequently, we consider that the field needs rigorous research to define numerical labels for the drug-drug interaction severity labels. Such an undertaking would not be trivial because merely respectively encoding "major", "moderate", "minor", and "no interactions were found" as "3", "2", "1", and "0" does not solve all issues. For instance, Drugs.com considers "major" interactions that are either "contraindicated" in any circumstance or tolerated under medical supervision; there is an evident difference between the two types of "major" drug-drug interactions.

Second, owing to the lack of negative information (i.e., drug-drug and drug-target interactions proven experimentally as nonexistent, see Section Discussion), there is still much uncertainty even in the latest drug database versions. Consequently, it remains hard to assess the performance of drug-drug and drug-target interaction prediction methods (as well as the effectiveness of computational drug repositioning pipelines) in the absence of standardized ground truth. In our opinion, to have a fair and reliable comparison of drug-drug and drug-target prediction methods, we need comprehensive benchmark datasets. In fairness, recent research efforts acknowledge this need for comprehensive benchmarking

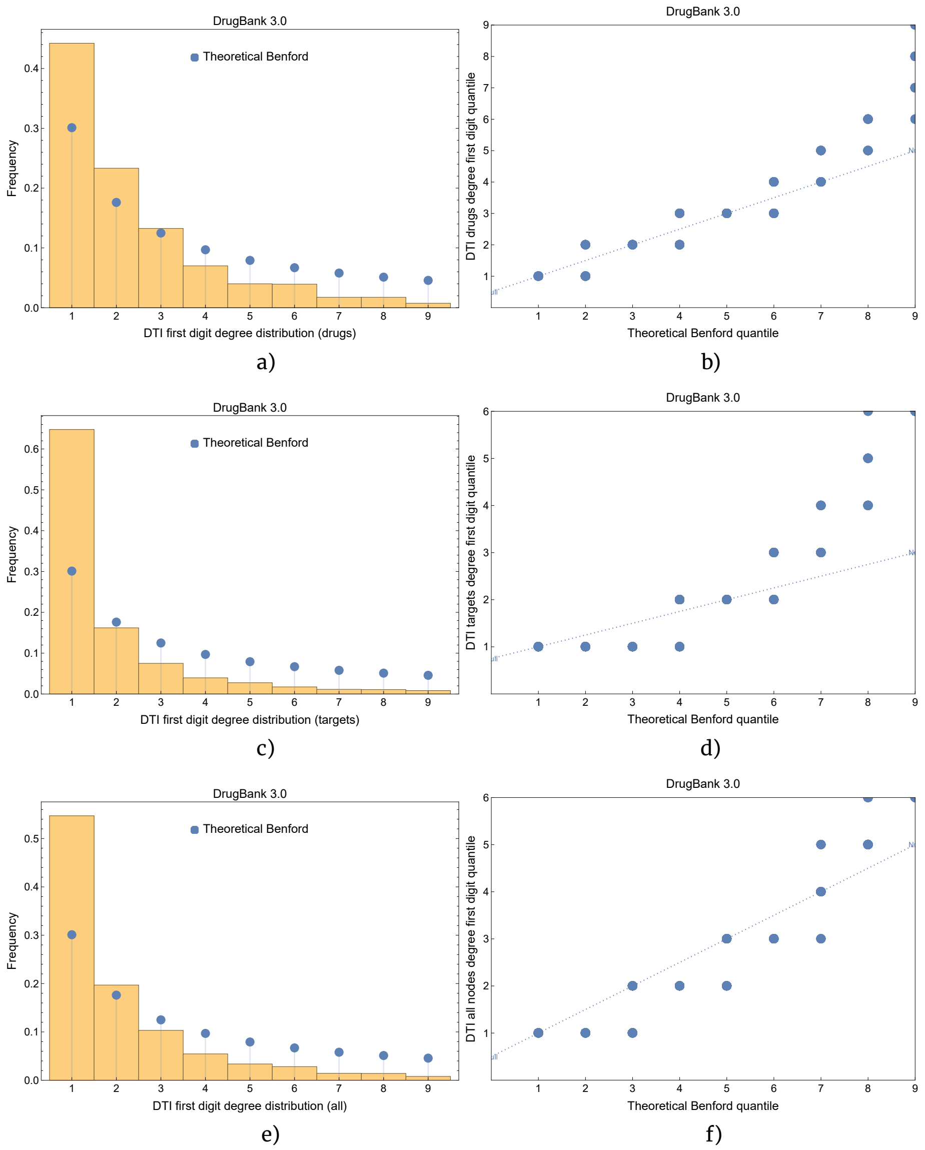

Figure 16. The compliance of degree distribution in DTI networks built with DrugBank 3.0 data. In panels a, c, and e, we compare the theoretical Benford distribution with blue disks and the degree distribution in DTI network nodes representing drugs, targets, and all DTI network nodes. In panels b, d, and f, we present the Q-Q plots corresponding to panels a, c, and e. All the results in this figure indicate non-compliance with the theoretical Benford distribution.

[80]; however, we think that a lot more research should be spent to standardize such benchmarks and benchmark suites, which the bioinformatics community should adopt. To this end, we believe we can draw inspiration from the way the computer architecture community managed to standardize the measurement of computer performance with the SPEC benchmark suites [83].

Third, any comprehensive benchmark dataset must contain negative information, namely drug-drug and drug-target interactions proven impossible (or inexistent). The effort of collecting a dataset of negative drug-target interaction examples is not necessarily daunting. To this end, we suggest that computational methods—such as molecular docking or molecular fingerprints [84, 40]—can be employed to precisely identify the most likely non-interactions. Thus, the resources entailed by the confirming experiments will be mitigated substantially.

Availability of source code and requirements

The page `https://github.com/research-hyperion/Drug_database_statistics` hosts the software implementing the data analysis methods described in this paper—entitled *Drug Database Statistics*. Our implementation uses Python and Wolfram Language; it is platform-independent and requires Docker Desktop on Microsoft Windows or Docker Engine on Linux distros. The software can be used under the GNU GPL v3.0 license.

Addtitional files

We provide all the analyses and simulations numerical results in file `https://github.com/research-hyperion/Drug_database_statistics/blob/master/Drug-networks-results-synthesis.xlsx`.

Declarations

List of abbreviations

ATC: anatomical therapeutic chemical classification system; COVID-19: coronavirus disease 2019; COX: cyclooxygenase; DDI: drug-drug interaction; DTI: drug-target interaction; EHR: electronic health record; FDA: United States food and drug administration; KGE: knowledge graph embedding; mRNA: messenger ribonucleic acid;

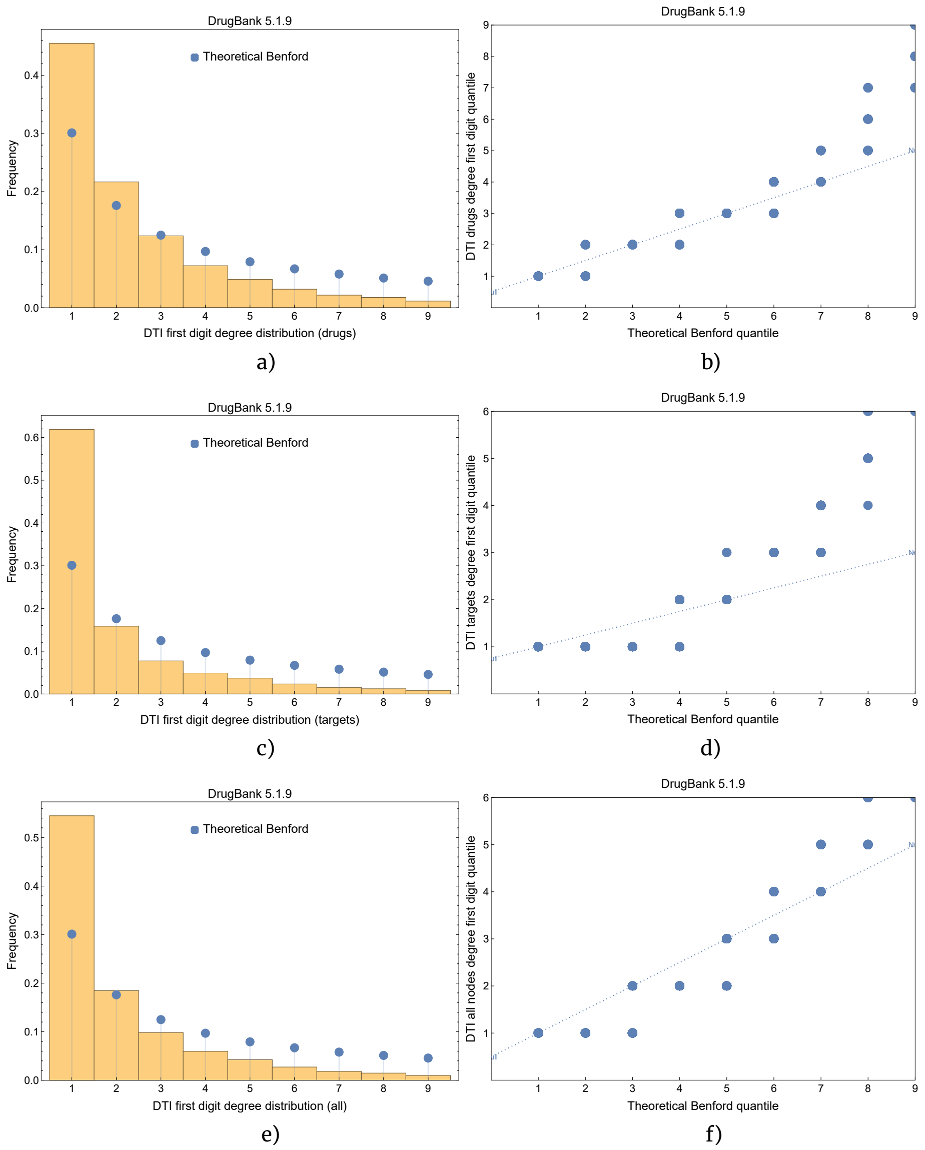

Figure 17. The compliance of degree distribution in DTI networks built with DrugBank 5.1.9 data (i.e., latest version). In panels a, c, and e, we compare the theoretical Benford distribution with blue disks and the degree distribution in DTI network nodes representing drugs, targets, and all DTI network nodes. In panels b, d, and f, we present the Q-Q plots corresponding to panels a, c, and e. All the results in this figure indicate non-compliance with the theoretical Benford distribution.

NFM: neural factorization machine; SPEC: standard performance evaluation corporation.

Ethical approval

Not applicable.

Consent for publication

Not applicable.

Competing interests

The authors declare that they have no competing interests.

Funding

This work was supported by a grant of the Romanian Ministry of Education and Research, CCCDI - UEFISCDI, project number PN-III-P2-2.1-PED-2019-2842, within PNCDI III.

Author's contributions

Design of study: M.U., L.U.; funding acquisition: M.U., L.U.; data analysis: M.U., S.M.A.; draft preparation: M.U., L.U.; review and editing: M.U., S.M.A., L.U.

Acknowledgements

Not applicable.

References

1. Recanatini M, Cabrelle C. Drug research meets network science: Where are we? Journal of medicinal chemistry 2020;63(16):8653–8666.
2. Azuaje F. Drug interaction networks: an introduction to translational and clinical applications. Cardiovascular research 2013;97(4):631–641.

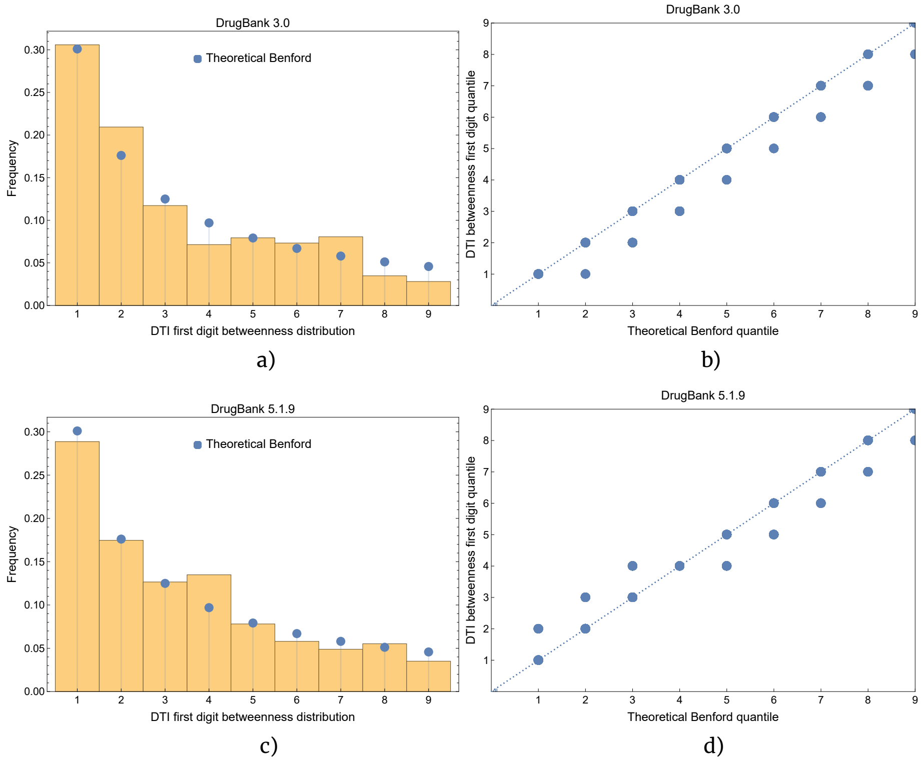

Figure 18. The compliance of betweenness distribution in DTI networks with Benford's law. In panels a and c, we compare the theoretical Benford distribution (blue disks) with the empirical betweenness distribution in DTI network nodes built with DrugBank 3.0 and 5.1.9 data, respectively. In panels b and d, we present the Q-Q plots corresponding to panels a and c. All the results in this figure indicate a relatively good compliance with the theoretical Benford distribution.

3. Zhang W, Chen Y, Liu F, Luo F, Tian G, Li X. Predicting potential drug-drug interactions by integrating chemical, biological, phenotypic and network data. BMC bioinformatics 2017;18(1):1–12.
4. Zhu J, Liu Y, Zhang Y, Chen Z, Wu X. Multi-Attribute Discriminative Representation Learning for Prediction of Adverse Drug-Drug Interaction. IEEE Transactions on Pattern Analysis and Machine Intelligence 2021;.
5. Qiu Y, Zhang Y, Deng Y, Liu S, Zhang W. A comprehensive review of computational methods for drug-drug interaction detection. IEEE/ACM Transactions on Computational Biology and Bioinformatics 2021;.
6. Su X, You ZH, Huang Ds, Wang L, Wong L, Ji B, et al. Biomedical Knowledge Graph Embedding with Capsule Network for Multi-label Drug-Drug Interaction Prediction. IEEE Transactions on Knowledge and Data Engineering 2022;.
7. Lu Y, Guo Y, Korhonen A. Link prediction in drug-target interactions network using similarity indices. BMC bioinformatics 2017;18(1):1–9.
8. Luo Y, Zhao X, Zhou J, Yang J, Zhang Y, Kuang W, et al. A network integration approach for drug-target interaction prediction and computational drug repositioning from heterogeneous information. Nature communications 2017;8(1):1–13.
9. Wu Z, Li W, Liu G, Tang Y. Network-based methods for prediction of drug-target interactions. Frontiers in pharmacology 2018;9:1134.
10. Thafar MA, Olayan RS, Ashoor H, Albaradei S, Bajic VB, Gao X, et al. DTiGEMS+: drug–target interaction prediction using graph embedding, graph mining, and similarity-based techniques. Journal of Cheminformatics 2020;12(1):1–17.
11. Thafar MA, Olayan RS, Albaradei S, Bajic VB, Gojobori T, Essack M, et al. DTi2Vec: Drug–target interaction prediction using network embedding and ensemble learning. Journal of cheminformatics 2021;13(1):1–18.
12. Lotfi Shahreza M, Ghadiri N, Mousavi SR, Varshosaz J, Green JR. A review of network-based approaches to drug repositioning. Briefings in bioinformatics 2018;19(5):878–892.
13. Sadeghi SS, Keyvanpour MR. An analytical review of computational drug repurposing. IEEE/ACM transactions on computational biology and bioinformatics 2019;18(2):472–488.
14. Badkas A, De Landtsheer S, Sauter T. Topological network measures for drug repositioning. Briefings in bioinformatics 2020;.
15. Jourdan JP, Bureau R, Rochais C, Dallemagne P. Drug repositioning: a brief overview. Journal of Pharmacy and Pharmacology 2020;72(9):1145–1151.
16. Bolgár B, Arany A, Temesi G, Balogh B, Antal P, Matyus P. Drug repositioning for treatment of movement disorders: from serendipity to rational discovery strategies. Current topics in medicinal chemistry 2013;13(18):2337–2363.
17. Sridhar D, Fakhraei S, Getoor L. A probabilistic approach for collective similarity-based drug–drug interaction prediction. Bioinformatics 2016;32(20):3175–3182.
18. Lin X, Quan Z, Wang ZJ, Ma T, Zeng X. KGNN: Knowledge Graph Neural Network for Drug-Drug Interaction Prediction. In: IJCAI, vol. 380; 2020. p. 2739–2745.
19. Feng YH, Zhang SW, Shi JY. DPDDI: a deep predictor for drug-drug interactions. BMC bioinformatics 2020;21(1):1–15.
20. Dickson M, Gagnon JP. The cost of new drug discovery and development. Discovery medicine 2009;4(22):172–179.
21. Chen XQ, Antman MD, Gesenberg C, Gudmundsson OS. Discovery pharmaceutics—challenges and opportunities. The AAPS journal 2006;8(2):E402–E408.
22. Food US, Administration D, Novel Drug Approvals for 2021; 2021. [Online; accessed 21-January-2022]. `https://www.fda.gov/drugs/new-drugs-fda-cders-new-molecular-entities-and-new-therapeutic-biological-products/novel-drug-approvals-2021`.
23. Sardana D, Zhu C, Zhang M, Gudivada RC, Yang L, Jegga AG. Drug repositioning for orphan diseases. Briefings in bioinfor-

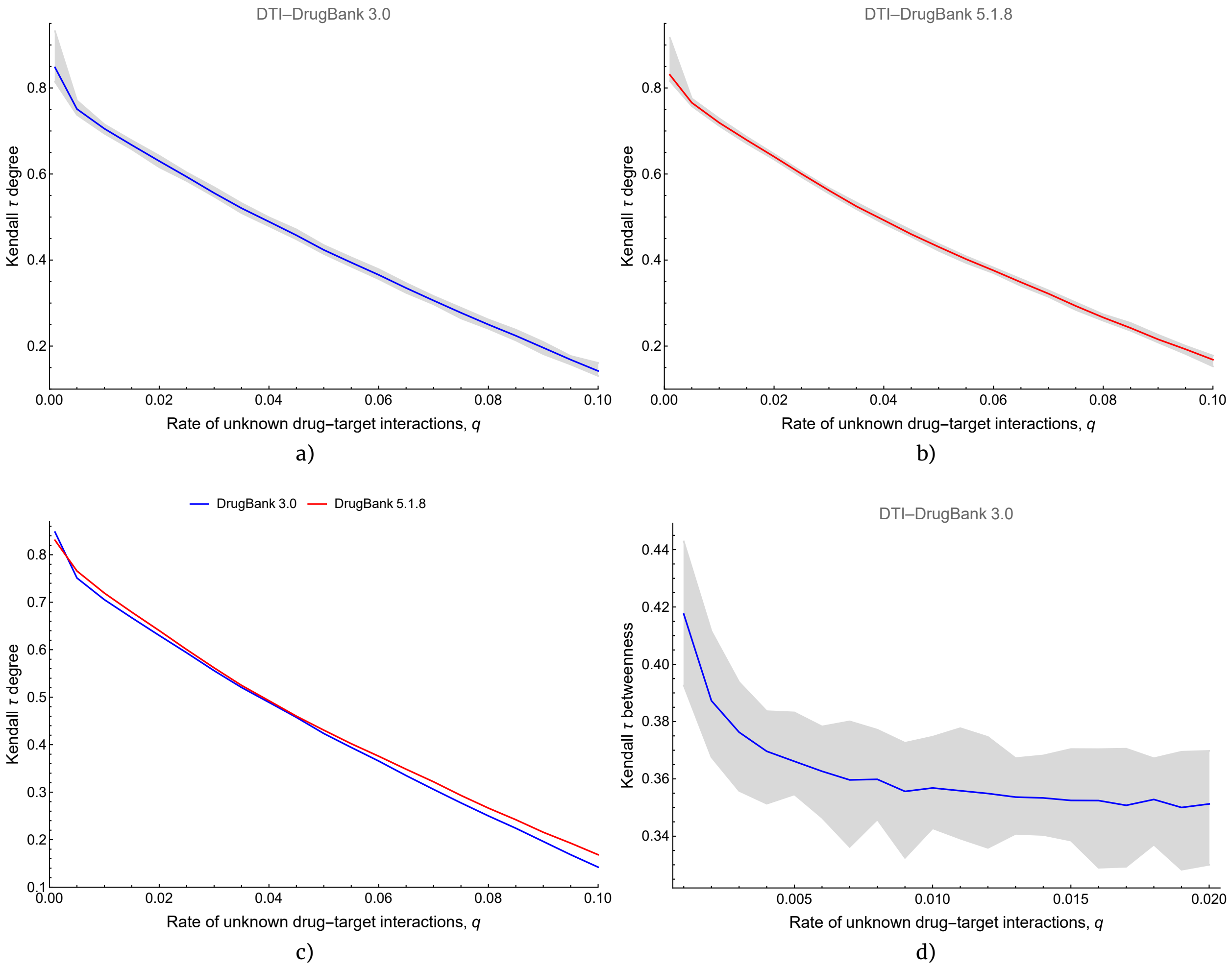

Figure 19. The evolution of Kendall τ (measuring the centrality-based ordinal correlation between the known DTI G and the DTI including unknown edges G') with the rate (i.e., fraction) of unknown edges q. Panel a shows the results for the DrugBank 3.0 DTI and the degree centrality, with the blue line following the average τ after 100 simulations and the grey area showing the variance. Panel b is the counterpart of panel a for DrugBank 5.1.8. Panel c presents the comparison between DrugBank 3.0 and 5.1.8 DTI networks. Panel d illustrates a robustness analysis similar to a, but for the betweenness centrality.

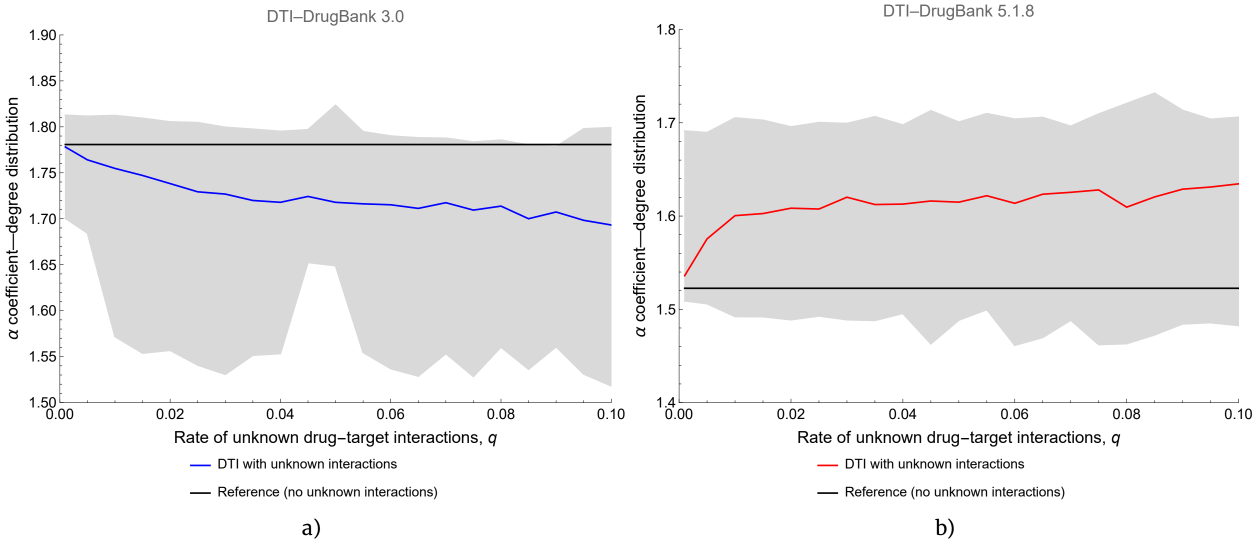

Figure 20. The evolution of the power-law distribution exponent α with the rate of unknown interactions q; panel a presents the case of the first DrugBank version 3.0 DTI network, whereas panel b shows the case of the DrugBank version 5.1.8 DTI network. In the figure, the black lines represent the reference α values (i.e., corresponding to DTI networks with no unknown interactions, $q = 0$). We performed simulations 100 times for each q value, showing the average values with blue and red lines, while the grey area represents the interval between minimum and maximum values.

matics 2011;12(4):346–356.
24. Serafin MB, Bottega A, Foletto VS, da Rosa TF, Hörner A, Hörner R. Drug repositioning is an alternative for the treatment of coronavirus COVID-19. International journal of antimicrobial

agents 2020;55(6):105969.
25. Gysi DM, Do Valle Í, Zitnik M, Ameli A, Gan X, Varol O, et al. Network medicine framework for identifying drug-repurposing opportunities for COVID-19. Proceedings of the National Academy of Sciences 2021;118(19).
26. Altman DG, Bland JM. Statistics notes: Absence of evidence is not evidence of absence. Bmj 1995;311(7003):485.
27. Mestres J, Gregori-Puigjane E, Valverde S, Sole RV. Data completeness—the Achilles heel of drug-target networks. Nature biotechnology 2008;26(9):983–984.
28. Wishart DS, Feunang YD, Guo AC, Lo EJ, Marcu A, Grant JR, et al. DrugBank 5.0: a major update to the DrugBank database for 2018. Nucleic acids research 2018;46(D1):D1074–D1082.
29. Avizienis A, Laprie JC, Randell B, Landwehr C. Basic concepts and taxonomy of dependable and secure computing. IEEE transactions on dependable and secure computing 2004;1(1):11–33.
30. Bleakley K, Yamanishi Y. Supervised prediction of drug–target interactions using bipartite local models. Bioinformatics 2009;25(18):2397–2403.
31. Cheng F, Liu C, Jiang J, Lu W, Li W, Liu G, et al. Prediction of drug-target interactions and drug repositioning via network-based inference. PLoS computational biology 2012;8(5):e1002503.
32. Lü L, Pan L, Zhou T, Zhang YC, Stanley HE. Toward link predictability of complex networks. Proceedings of the National Academy of Sciences 2015;112(8):2325–2330.
33. Xue H, Li J, Xie H, Wang Y. Review of drug repositioning approaches and resources. International journal of biological sciences 2018;14(10):1232.
34. Wu Z, Wang Y, Chen L. Network-based drug repositioning. Molecular BioSystems 2013;9(6):1268–1281.
35. Udrescu L, Sbârcea L, Topîrceanu A, Iovanovici A, Kurunczi L, Bogdan P, et al. Clustering drug-drug interaction networks with energy model layouts: community analysis and drug repurposing. Scientific reports 2016;6(1):1–10.
36. Yamanishi Y, Araki M, Gutteridge A, Honda W, Kanehisa M. Prediction of drug–target interaction networks from the integration of chemical and genomic spaces. Bioinformatics 2008;24(13):i232–i240.
37. Mestres J, Gregori-Puigjané E, Valverde S, Solé RV. The topology of drug–target interaction networks: implicit dependence on drug properties and target families. Molecular BioSystems 2009;5(9):1051–1057.
38. Tabei Y, Pauwels E, Stoven V, Takemoto K, Yamanishi Y. Identification of chemogenomic features from drug–target interaction networks using interpretable classifiers. Bioinformatics 2012;28(18):i487–i494.
39. Tanoli Z, Alam Z, Ianevski A, Wennerberg K, Vähä-Koskela M, Aittokallio T. Interactive visual analysis of drug–target interaction networks using drug target profiler, with applications to precision medicine and drug repurposing. Briefings in bioinformatics 2020;21(1):211–220.
40. Udrescu L, Bogdan P, Chiş A, Sîrbu IO, Topîrceanu A, Văruţ RM, et al. Uncovering New Drug Properties in Target-Based Drug–Drug Similarity Networks. Pharmaceutics 2020;12(9):879.
41. Newman ME, Barabási ALE, Watts DJ. The structure and dynamics of networks. Princeton university press; 2006.
42. Avram S, Halip L, Curpan R, Oprea TI. Novel drug targets in 2021. Nature reviews Drug Discovery 2022;.
43. Wishart DS, Knox C, Guo AC, Cheng D, Shrivastava S, Tzur D, et al. DrugBank: a knowledgebase for drugs, drug actions and drug targets. Nucleic acids research 2008;36(suppl_1):D901–D906.
44. Wishart DS, Knox C, Guo AC, Shrivastava S, Hassanali M, Stothard P, et al. DrugBank: a comprehensive resource for in silico drug discovery and exploration. Nucleic acids research 2006;34(suppl_1):D668–D672.
45. Newman ME. The structure and function of networks. Computer Physics Communications 2002;147(1-2):40–45.
46. Wang XF, Chen G. Complex networks: small-world, scale-free and beyond. IEEE circuits and systems magazine 2003;3(1):6–20.
47. Barabási AL. Network science. Philosophical Transactions of the Royal Society A: Mathematical, Physical and Engineering Sciences 2013;371(1987):20120375.
48. Jeong H, Mason SP, Barabási AL, Oltvai ZN. Lethality and centrality in protein networks. Nature 2001;411(6833):41–42.
49. Koschützki D, Schreiber F. Comparison of centralities for biological networks. In: German Conference on Bioinformatics 2004, GCB 2004 Society for Computer Science eV; 2004. .
50. Salavati C, Abdollahpouri A, Manbari Z. Ranking nodes in complex networks based on local structure and improving closeness centrality. Neurocomputing 2019;336:36–45.
51. Yıldırım MA, Goh KI, Cusick ME, Barabási AL, Vidal M. Drug—target network. Nature biotechnology 2007;25(10):1119–1126.
52. MacCuish JD, MacCuish NE. Clustering in bioinformatics and drug discovery. CRC Press; 2010.
53. Estrada E. The structure of complex networks: theory and applications. Oxford University Press; 2012.
54. Topirceanu A, Udrescu M, Vladutiu M. Genetically optimized realistic social network topology inspired by facebook. In: Online Social Media Analysis and Visualization Springer; 2014.p. 163–179.
55. Orita M, Hagiwara Y, Moritomo A, Tsunoyama K, Watanabe T, Ohno K. Agreement of drug discovery data with Benford's law. Expert opinion on drug discovery 2013;8(1):1–5.
56. Morzy M, Kajdanowicz T, Szymański BK. Benford's distribution in complex networks. Scientific reports 2016;6(1):1–8.
57. Grandison S, Morris RJ. Biological pathway kinetic rate constants are scale-invariant. Bioinformatics 2008;24(6):741–743.
58. Karthik D, Stelzer G, Gershanov S, Baranes D, Salmon-Divon M. Elucidating tissue specific genes using the Benford distribution. BMC genomics 2016;17(1):1–15.
59. Kastrin A, Ferk P, Leskošek B. Predicting potential drug-drug interactions on topological and semantic similarity features using statistical learning. PloS one 2018;13(5):e0196865.
60. Goh KI, Cusick ME, Valle D, Childs B, Vidal M, Barabási AL. The human disease network. Proceedings of the National Academy of Sciences 2007;104(21):8685–8690.
61. Barabási AL. Scale-free networks: a decade and beyond. science 2009;325(5939):412–413.
62. Alstott J, Bullmore E, Plenz D. powerlaw: a Python package for analysis of heavy-tailed distributions. PloS one 2014;9(1):e85777.
63. Kossovsky AE. On the Mistaken Use of the Chi-Square Test in Benford's Law. Stats 2021;4(2):419–453.
64. Bhardwaj S, Niyogi R, Milani A. Performance analysis of an algorithm for computation of betweenness centrality. In: International Conference on Computational Science and Its Applications Springer; 2011. p. 537–546.
65. Christensen D. Fast algorithms for the calculation of Kendall's τ. Computational Statistics 2005;20(1):51–62.
66. Tilson H, Hines LE, McEvoy G, Weinstein DM, Hansten PD, Matuszewski K, et al. Recommendations for selecting drug–drug interactions for clinical decision support. American Journal of health-system pharmacy 2016;73(8):576–585.
67. Dumbreck S, Flynn A, Nairn M, Wilson M, Treweek S, Mercer SW, et al. Drug-disease and drug-drug interactions: systematic examination of recommendations in 12 UK national clinical guidelines. bmj 2015;350.
68. Monteith S, Glenn T, Gitlin M, Bauer M. Potential drug interactions with drugs used for bipolar disorder: A comparison of 6 drug interaction database programs. Pharmacopsychiatry 2020;53(05):220–227.
69. Phansalkar S, Van der Sijs H, Tucker AD, Desai AA, Bell DS,

Teich JM, et al. Drug—drug interactions that should be non-interruptive in order to reduce alert fatigue in electronic health records. Journal of the American Medical Informatics Association 2013;20(3):489–493.
70. Pirnejad H, Amiri P, Niazkhani Z, Shiva A, Makhdoomi K, Abkhiz S, et al. Preventing potential drug-drug interactions through alerting decision support systems: a clinical context based methodology. International journal of medical informatics 2019;127:18–26.
71. Phansalkar S, Desai A, Choksi A, Yoshida E, Doole J, Czochanski M, et al. Criteria for assessing high-priority drug-drug interactions for clinical decision support in electronic health records. BMC medical informatics and decision making 2013;13(1):1–11.
72. Kontsioti E, Maskell S, Dutta B, Pirmohamed M. A reference set of clinically relevant adverse drug-drug interactions. Scientific Data 2022;9(1):1–9.
73. Assiri A, Noor A. Anti-DDI Resource: A Dataset for Potential Negative Reported Interaction Combinations to Improve Medical Research and Decision-Making. Journal of Healthcare Engineering 2022;2022.
74. Rohani N, Eslahchi C. Drug-drug interaction predicting by neural network using integrated similarity. Scientific reports 2019;9(1):1–11.
75. Chen ZH, You ZH, Guo ZH, Yi HC, Luo GX, Wang YB. Prediction of drug–target interactions from multi-molecular network based on deep walk embedding model. Frontiers in Bioengineering and Biotechnology 2020;8:338.
76. Ye Q, Hsieh CY, Yang Z, Kang Y, Chen J, Cao D, et al. A unified drug–target interaction prediction framework based on knowledge graph and recommendation system. Nature communications 2021;12(1):1–12.
77. Hu S, Zhang C, Chen P, Gu P, Zhang J, Wang B. Predicting drug-target interactions from drug structure and protein sequence using novel convolutional neural networks. BMC bioinformatics 2019;20(25):1–12.
78. Lee I, Keum J, Nam H. DeepConv-DTI: Prediction of drug-target interactions via deep learning with convolution on protein sequences. PLoS computational biology 2019;15(6):e1007129.
79. Hassanzadeh R, Shabani-Mashcool S. Does adding the drug-drug similarity to drug-target interaction prediction methods make a noticeable improvement in their efficiency? 2022;.
80. Zong N, Li N, Wen A, Ngo V, Yu Y, Huang M, et al. BETA: a comprehensive benchmark for computational drug–target prediction. Briefings in Bioinformatics 2022;.
81. Bagherian M, Sabeti E, Wang K, Sartor MA, Nikolovska-Coleska Z, Najarian K. Machine learning approaches and databases for prediction of drug–target interaction: a survey paper. Briefings in bioinformatics 2021;22(1):247–269.
82. Chen X, Yan CC, Zhang X, Zhang X, Dai F, Yin J, et al. Drug–target interaction prediction: databases, web servers and computational models. Briefings in bioinformatics 2016;17(4):696–712.
83. Panda R, Song S, Dean J, John LK. Wait of a decade: Did SPEC CPU 2017 broaden the performance horizon? In: 2018 IEEE International Symposium on High Performance Computer Architecture (HPCA) IEEE; 2018. p. 271–282.
84. Shi H, Liu S, Chen J, Li X, Ma Q, Yu B. Predicting drug-target interactions using Lasso with random forest based on evolutionary information and chemical structure. Genomics 2019;111(6):1839–1852.

Supplementary Material
Click here to access/download;Personal Cover;Response to reviewers and marked changes.pdf

Response to Reviewers

November 18, 2022

We thank the Academic Editor for allowing us to upgrade our manuscript and to address all comments. We would also like to thank the reviewers for their constructive feedback, which we believe has improved the quality of our paper. The revised version of the manuscript highlights our changes in blue.

Since all three reviewers raise multiple points, we have taken the liberty of responding point by point. All text written in ***bolded italics*** corresponds to Reviewers' comments. Our responses are written with regular fonts, while the references we made to changes incorporated in the revised manuscript are underlined.

We hope to have answered all of the reviewers' comments satisfactorily.

EDITOR COMMENTS

In particular, both reviewers feel the problem addressed in the paper and related concepts need to be explained and defined more clearly.

We carefully addressed the concerns formulated by the reviewers—particularly those concerning the concepts we used—and hope they are explained more clearly in the revised version of the manuscript.

REVIEWER COMMENTS

Reviewer #1 (Remarks to the Author):

In recent years, more and more comprehensive drug datasets have been proposed, however, there is much uncertainty in these drug datasets. Researchers cannot know if the not reported interactions are absent or yet to be discovered, which hampers the accuracy of bioinformatics applications on these datasets. The authors used complex network statistics tools and simulations of randomly-inserted previously unaccounted interactions in drug-drug and drug-target interaction networks, and found that the drug-drug interaction networks built with the latest dataset versions become very dense and, therefore, almost impossible to analyze with conventional complex network methods. On the other hand, for the latest drug database versions, drug-target networks still include much uncertainty; however, the robustness of complex network analysis methods slightly improves. The authors pinpoint future research

directions to improve the quality and practicality of drug databases for bioinformatics applications: benchmarking for drug-target interaction prediction and drug-drug interaction severity standardization.

We very much appreciate the thorough, insightful, and fair assessment of our work. The observations and comments will undoubtedly contribute to a more robust, readable, and wide-reaching paper.

Comments and suggestions:

1. ***The author should explain more clearly the relationship among 'uncertain issue of datasets', 'the evolution of various complex network parameters and centralities', 'the integrity of the processed data' and 'the robustness of centrality-hierarchization analysis methods'.***

We thank the reviewer for showing the need for clarity in the Background section—the part dedicated to analyzing the current issues in drug dataset analysis and the summary of our contributions. We used the terms cited by the reviewer in the enumeration (with bullets) of "our DDI and DTI network analysis across the DrugBank database versions."

In the initially submitted manuscript, the paragraph that tried to explain the connections between the "uncertainty issue of datasets," "the evolution of various complex network parameters and centralities," "the integrity of the processed data," and "the robustness of centrality-hierarchization analysis methods" read:
"Over the years, the comprehensiveness of drug datasets (such as DrugBank [28]) has constantly grown, and the data accuracy improved by manual curation, according to the latest literature results. This constant evolution of the most comprehensive drug database may have mitigated the concerns formulated by Mestres et al. Our paper investigates how the evolution of knowledge on drug interactions, mirrored by the DrugBank database, impacts the robustness of drug interaction network analysis; future research can exploit the insight we get to advance drug repositioning and drug-drug interaction prediction methods. "

Nonetheless, the reviewer is right. In the above paragraph, we used different terms than those used in the enumeration of the contributions, causing a lack of clarity and confusion. Following the reviewer's suggestion, we revised the paragraph that was intended to explain the mentioned terms. We hope that this part is much clearer in the revised form, which reads:

"As long as we do not have comprehensive negative information, the issue of **uncertainty in drug datasets** remains. However, the fact that—over the years—the comprehensiveness of drug datasets (such as DrugBank [28]) has constantly grown, and experts curated the data according to the latest literature results, may suggest that the **integrity of the data** improved. (The integrity characterizes

the "absence of improper data alterations" [29].) Indeed, the constant growth of the most comprehensive drug database (reflected in the **evolution of the various complex drug network parameters and centralities**) may have mitigated the concerns formulated by Mestres et al. Our paper investigates how the evolution of knowledge on drug interactions mirrored by the DrugBank database, impacts the **robustness of drug interaction network analysis based on centrality hierarchization methods**; future research can exploit the insight we get to advance drug repositioning and drug-drug interaction prediction methods." (Section *Background*, paragraph 7 on page 2 in the revised manuscript.)

We introduced a new reference that acknowledges the rigorous definition of system integrity:
[29] Avizienis, A., Laprie, J.C., Randell, B. and Landwehr, C., 2004. Basic concepts and taxonomy of dependable and secure computing. IEEE Transactions on Dependable and Secure Computing, 1(1), pp.11-33.

2. ***The author should clearly define the 'typical complex network', for example, the characteristics of a typical complex network and the differences between a typical complex network and a normal network. In addition, the first sentence of the first paragraph in the section 'Complex networks' seems to be a general definition of the 'network', not the 'complex network'.***

We thank the reviewer for the observation. First, we added the necessary details to the definition of complex networks, which now reads, "A complex network is a graph *G* consisting of a node (or vertex) set *V* and a link (or edge) set *E*, *G* = (*V*, *E*), where any edge $e_{ij} \in E$ connects nodes $v_i, v_j \in V$. Additionally, compared to normal graphs, real-world complex networks have a large number of nodes $|V|$ (i.e., hundreds to millions) and intricate, nontrivial topologies (i.e., links do not form regular structures but are not completely random either)." We highlighted the addition in blue text, in section *Problem formulation*, first paragraph in subsection *Complex networks*, page 3.

Second, we defined the typical characteristics of real-world complex networks in the manuscript in a new subsection titled *Typical complex network characteristics* (page 6 in the revised manuscript). We also point to this new subsection when using the term "typical complex networks" for the first time: "We explain the typical characteristics of real-world complex networks in subsection *Typical complex network characteristics*" (see the blue text on page 3, 4th paragraph). In the revised form of our manuscript, this new subsection reads, "Generally, in real-world complex networks, the density of links is low (i.e., $\sim \frac{1}{n}$ because the number of links is of the order *n*, where *n* is the number of nodes, and far from the order n^2 corresponding to a completely connected network); the total number of links also determines the average degree, and the average degree is significantly lower than *n*; the degree follows either a power-law (exponent is between 1 and 3) or normal distribution; the average path length is between 3 and 10; the clustering

coefficient is larger than $\frac{1}{n}$ but considerably lower than 1 [46, 54]." We highlighted these clarifications in blue text, section *Methods*, subsection *Typical complex network characteristics*, on page 6.

[46] Wang, X.F. and Chen, G., 2003. Complex networks: small-world, scale-free and beyond. IEEE circuits and systems magazine, 3(1), pp.6-20.
[54] Topirceanu, A., Udrescu, M. and Vladutiu, M., 2014. Genetically optimized realistic social network topology inspired by facebook. In Online Social Media Analysis and Visualization (pp. 163-179). Springer, Cham.

3. ***In the last sentence of the third paragraph in the section 'Complex networks', 'wij≠ wji' should be 'wij=wji'.***

The observation is correct, and we thank the reviewer for pointing out this typo; we corrected it in the revision (see the first paragraph on page 4).

4. ***In Equation (10), the function η() doesn't seem to be defined.***

The reviewer is right; we did not explain the eigenvector with clarity (while maintaining conciseness). We denoted the eccentricity of a node v_i as $\eta(v_i)$. Hence, equation (10) shows that eigenvector centrality for v_i is defined recursively: it adds the centralities of the nodes directly connected to v_i. To find the eigenvector centrality values for all nodes in the node set V of the network $G = (V, E)$, we solve the equation $W\boldsymbol{\eta} = \lambda\boldsymbol{\eta}$; W is the adjacency matrix, λ is a constant, and $\boldsymbol{\eta}$ is the vector of eigenvector centrality values for all nodes in G. We added these clarifying details for equation 10 in the revised manuscript (please see the blue text on page 6, subsubsection *Network centralities*).

5. ***In the section 'Discussion', the authors should include more analysis of the results. In addition, I suggest that the authors should elaborate analysis and conclusions with relevant findings from the experimental results, instead of talking about them separately.***

The reviewer is correct: our *Discussion* section needs to be organized better. According to the suggestions, we performed the following steps:

1. We renamed the subsections of the *Discussion* as *Discussions on DDI networks* and *Discussions on DTI networks*.
2. We extended the discussions to new issues, such as:
    a. The benefits of building prediction tools for identifying negative information (i.e., drug-drug and drug-target interactions that do not exist);

    b. The need for recording the relevant clinical context for drug-drug interaction severity;
    c. The fact that different drug datasets often disagree on the drug-drug interaction severity.
3. We reorganized the subsections such that we directly corroborate our analysis conclusions with the experimental results; these two parts are no longer separated.
4. We rewrite the entire *Discussion* section to eliminate redundant wording, thus bringing more conciseness and clarity to the manuscript.

Please find the revised form of the *Discussion* section in blue, starting on page 10.

6. ***Many important literature about the drug interactions have not been reviewed in the paper, such as DOI:10.1109/TPAMI.2021.3135841, DOI:10.1109/TKDE.2022.3154792, doi: 10.1186/s12859-016-1415-9, DOI:10.1109/TCBB.2021.3081268, etc.***

We thank the reviewer for pointing out that we did not include proper citations for papers on drug-drug interaction predictions reached from drug-drug and drug-target interactions. Indeed, drug-drug interaction prediction is highly relevant to the central theme of our paper. We added a paragraph in the Background section that explains the application of drug-drug and drug-target interaction prediction (besides drug repositioning, which we already presented in detail), where we correctly cited the outstanding work on drug-drug interaction prediction suggested by the reviewer. (Please, see the blue-text in section *Background*, first two paragraphs on page 2.) The new paragraphs read, "As such, harnessing big amounts of data describing the intricate drug interactions (or relationships) with other entities can lead to uncovering new drug properties: previously unknown drug-drug [3, 4, 5, 6] or drug-target interactions [7, 8, 9, 10, 11], drug repositioning [12, 13, 14]. // Computational drug-drug interaction (DDI) prediction is critical in designing effective and safe drug combinations and developing drugs [5, 6]. Drug-target interaction (DTI) prediction is a key-step strategy in drug discovery that leads to uncovering new drugs for biological targets and new targets for known, approved drugs—representing a crucial step towards drug repositioning and identifying possible side effects [8, 11]."

[3] Zhang, W., Chen, Y., Liu, F., Luo, F., Tian, G. and Li, X., 2017. Predicting potential drug-drug interactions by integrating chemical, biological, phenotypic and network data. BMC bioinformatics, 18(1), pp.1-12.

[4] Zhu, J., Liu, Y., Zhang, Y., Chen, Z. and Wu, X., 2021. Multi-Attribute Discriminative Representation Learning for Prediction of Adverse Drug-Drug Interaction. IEEE Transactions on Pattern Analysis and Machine Intelligence.

[5] Qiu, Y., Zhang, Y., Deng, Y., Liu, S. and Zhang, W., 2021. A comprehensive review of computational methods for drug-drug interaction detection. IEEE/ACM Transactions on Computational Biology and Bioinformatics.

[6] Su, X., You, Z.H., Huang, D.S., Wang, L., Wong, L., Ji, B. and Zhao, B., 2022. Biomedical Knowledge Graph Embedding with Capsule Network for Multi-label Drug-Drug Interaction Prediction. IEEE Transactions on Knowledge and Data Engineering.
[7] Lu, Y., Guo, Y. and Korhonen, A., 2017. Link prediction in drug-target interactions network using similarity indices. BMC bioinformatics, 18(1), pp.1-9.
[8] Luo, Y., Zhao, X., Zhou, J., Yang, J., Zhang, Y., Kuang, W., Peng, J., Chen, L. and Zeng, J., 2017. A network integration approach for drug-target interaction prediction and computational drug repositioning from heterogeneous information. Nature communications, 8(1), pp.1-13.
[9] Wu, Z., Li, W., Liu, G. and Tang, Y., 2018. Network-based methods for prediction of drug-target interactions. Frontiers in pharmacology, 9, p.1134.
[10] Thafar, M.A., Olayan, R.S., Ashoor, H., Albaradei, S., Bajic, V.B., Gao, X., Gojobori, T. and Essack, M., 2020. DTiGEMS+: drug–target interaction prediction using graph embedding, graph mining, and similarity-based techniques. Journal of Cheminformatics, 12(1), pp.1-17.
[11] Thafar, M.A., Olayan, R.S., Albaradei, S., Bajic, V.B., Gojobori, T., Essack, M. and Gao, X., 2021. DTi2Vec: Drug–target interaction prediction using network embedding and ensemble learning. Journal of cheminformatics, 13(1), pp.1-18.
[12] Lotfi Shahreza, M., Ghadiri, N., Mousavi, S.R., Varshosaz, J. and Green, J.R., 2018. A review of network-based approaches to drug repositioning. Briefings in bioinformatics, 19(5), pp.878-892.
[13] Sadeghi, S.S. and Keyvanpour, M.R., 2019. An analytical review of computational drug repurposing. IEEE/ACM transactions on computational biology and bioinformatics, 18(2), pp.472-488.
[14] Badkas, A., De Landtsheer, S. and Sauter, T., 2021. Topological network measures for drug repositioning. Briefings in bioinformatics, 22(4), p.bbaa357.

Reviewer #2 (Remarks to the Author):

The authors proposed an interesting analysis study for several drug-related networks using different versions of the DrugBank database. Therefore, the work is network analysis and machine learning-based methods to analyze the future of drug-drug interactions and drug-target interactions. Hence, this study highlights the future research that can improve the drug databases quality and for bioinformatics applications.

This work tackles a genuine topic to improve bioinformatics applications using complex networks of drug-related data. The abstract and the introduction were well written and presented good motivation, description. However, I have some concerns that have to be addressed. Therefore, I do recommend this work for publications in the GigaScience journal after addressing the following comments.

We thank the reviewer for delivering a sharp, fair, and comprehensive summary of our work. The provided comments will substantially contribute to a much more robust, readable, and wide-reaching manuscript.

Comments

Comment 1. The problem formulation is not very clear. More details are needed for the negative or missing information but focused on the problem itself.

We thank the reviewer for pointing out this issue. Indeed, we misplaced the explanation of the drug-drug and drug-target interaction networks using the formal framework from the *Problem formulation* section in the *Results*. We corrected this mistake by moving this part in the *Problem formulation*. Also, following the reviewer's suggestion, we used network formalism to explain better what missing and negative information means in the context of our problem. <u>These added details and corrections are on page 4, section *Problem formulation*, subsection *Drug networks* (first three paragraphs)</u>. In the revised manuscript, these paragraphs read:

“To mine the complexity of drug interactions in the biological environment, researchers use database information to build drug networks such as drug-drug, drug-target, and drug-disease interaction networks, as well as drug-disease and drug--adverse effects association networks. This paper considers drug-drug interaction networks (complex drug networks where nodes represent drugs) and drug-target interaction networks (bipartite networks where one type of node represents drugs, and the other represents biological targets). Links represent drug-drug and drug-target interactions in the DDI and DTI networks. We do not consider drug-drug interaction type and strength. Consequently, the DDI networks are undirected, unweighted, and monopartite. Also, the drug-target interactions have no information regarding their strength, making DTI networks directed (from drug to target), unweighted, and bipartite.

Using the complex network formalism, in DDI networks, $\forall i, j, i \neq j, v_i \in V = \mathcal{D}$ and $e_{ij} \in E = \mathcal{I}$ ($e_{ij} \neq 0$), where $\mathcal{D}$ is the set of drugs and $\mathcal{I}$ the set of drug-drug interactions. In DTI networks, we have $v_i \in V = V_{\mathcal{D}} \cup V_{\mathcal{T}}$ (where $V_{\mathcal{D}}$ is the set of nodes representing drugs and $V_{\mathcal{T}}$ is the set of nodes representing targets) and $e_{ij} \in E = \mathcal{A}$, where $\mathcal{A} = \{e_{ij} | v_i \in V_{\mathcal{D}}, v_j \in V_{\mathcal{T}}, i \neq j, e_{ij} \neq 0\}$ represents the set of drug-target interactions). Additionally, the set of non-edges in the DDI networks is $\bar{\mathcal{I}} = \{e_{ij} | v_i, v_j \in \mathcal{D}, i \neq j, e_{ij} = 0\}$ and in the DTI is $\bar{\mathcal{A}} = \{e_{ij} | v_i \in \mathcal{D}, v_j \in \mathcal{T}, i \neq j, e_{ij} = 0\}$.

A non-edge in a DDI network ($\bar{e}_{ij} \in \bar{\mathcal{I}}$) or a DTI network ($\in \bar{\mathcal{A}}$) represents a drug-drug or a drug-target interaction that has a probability $p \in (0,1)$ of being discovered in the future (i.e., missing information generating uncertainty). If $p = 0$, we are sure that interaction between v_i and v_j is impossible (i.e., the negative information on drug interactions)."

Comment 2. The manuscript covered a literature in terms of the methods using the DrugBank database. However, this literature is not enough and more discussion about drug repositioning applications using drug-drug and drug-target network-based methods or other types of applications using DrugBank datasets can improve the manuscript, specifically the literature review. Thus, citing more relevant and recent literature on DTI and DDI prediction is recommended, which will enrich the paper.

The reviewer is correct in pointing out that the Background section only provides details and citations on the drug repositioning applications. Accordingly, we corrected this downside by explaining the drug-drug and drug-target interaction prediction benefits; we cited the relevant recent literature (please, see the blue-text in section *Background*, first 2 paragraphs on page 2.)

The new paragraph reads, "As such, harnessing big amounts of data describing the intricate drug interactions (or relationships) with other entities can lead to uncovering new drug properties: previously unknown drug-drug [3, 4, 5, 6] or drug-target interactions [7, 8, 9, 10, 11], drug repositioning [12, 13, 14]. Computational drug-drug interaction (DDI) prediction is critical in designing effective and safe drug combinations and developing drugs [5, 6]. Drug-target interaction (DTI) prediction is a key-step strategy in drug discovery that leads to uncovering new drugs for biological targets and new targets for known, approved drugs—representing a crucial step towards drug repositioning and identifying possible side effects [8, 11]."

[3] Zhang, W., Chen, Y., Liu, F., Luo, F., Tian, G. and Li, X., 2017. Predicting potential drug-drug interactions by integrating chemical, biological, phenotypic and network data. BMC bioinformatics, 18(1), pp.1-12.

[4] Zhu, J., Liu, Y., Zhang, Y., Chen, Z. and Wu, X., 2021. Multi-Attribute Discriminative Representation Learning for Prediction of Adverse Drug-Drug Interaction. IEEE Transactions on Pattern Analysis and Machine Intelligence.
[5] Qiu, Y., Zhang, Y., Deng, Y., Liu, S. and Zhang, W., 2021. A comprehensive review of computational methods for drug-drug interaction detection. IEEE/ACM Transactions on Computational Biology and Bioinformatics.
[6] Su, X., You, Z.H., Huang, D.S., Wang, L., Wong, L., Ji, B. and Zhao, B., 2022. Biomedical Knowledge Graph Embedding with Capsule Network for Multi-label Drug-Drug Interaction Prediction. IEEE Transactions on Knowledge and Data Engineering.
[7] Lu, Y., Guo, Y. and Korhonen, A., 2017. Link prediction in drug-target interactions network using similarity indices. BMC bioinformatics, 18(1), pp.1-9.
[8] Luo, Y., Zhao, X., Zhou, J., Yang, J., Zhang, Y., Kuang, W., Peng, J., Chen, L. and Zeng, J., 2017. A network integration approach for drug-target interaction prediction and computational drug repositioning from heterogeneous information. Nature communications, 8(1), pp.1-13.
[9] Wu, Z., Li, W., Liu, G. and Tang, Y., 2018. Network-based methods for prediction of drug-target interactions. Frontiers in pharmacology, 9, p.1134.
[10] Thafar, M.A., Olayan, R.S., Ashoor, H., Albaradei, S., Bajic, V.B., Gao, X., Gojobori, T. and Essack, M., 2020. DTiGEMS+: drug–target interaction prediction using graph embedding, graph mining, and similarity-based techniques. Journal of Cheminformatics, 12(1), pp.1-17.
[11] Thafar, M.A., Olayan, R.S., Albaradei, S., Bajic, V.B., Gojobori, T., Essack, M. and Gao, X., 2021. DTi2Vec: Drug–target interaction prediction using network embedding and ensemble learning. Journal of cheminformatics, 13(1), pp.1-18.
[12] Lotfi Shahreza, M., Ghadiri, N., Mousavi, S.R., Varshosaz, J. and Green, J.R., 2018. A review of network-based approaches to drug repositioning. Briefings in bioinformatics, 19(5), pp.878-892.
[13] Sadeghi, S.S. and Keyvanpour, M.R., 2019. An analytical review of computational drug repurposing. IEEE/ACM transactions on computational biology and bioinformatics, 18(2), pp.472-488.
[14] Badkas, A., De Landtsheer, S. and Sauter, T., 2021. Topological network measures for drug repositioning. Briefings in bioinformatics, 22(4), p.bbaa357.

Comment 3. The methodology section is a bit distracting. A summary paragraph can be included as well as the structure of this section can be improved.

We appreciate this observation. The reviewer is correct; in the initially submitted manuscript, the *Methodology* section assembles all the subsections containing formal definitions, methods, and algorithms. Indeed, the *Problem formulation* is not a method, so we moved this part out of the *Methods* as a standalone/independent section (starting on page 3). We also added a summary

paragraph to explain the structure of the *Methods* section, as suggested by the reviewer. The new paragraph, highlighted in blue on page 5, at the beginning of the section *Methods*, reads:
"In this section, we describe the statistical and computational methods employed in this paper: subsection *Network analysis* introduces the main metrics and centralities required for the computational and statistical analysis of complex drug networks; subsection *Database quality* presents the statistical methods we use to test the drug database integrity; subsection *Network analysis robustness* explains the computer simulation method we propose to assess the robustness of the centrality-based drug network analysis strategies; subsection *Computational methods* describes the tools we use in this paper to implement the required computational and statistical techniques."

Comment 4. The benefit of Figure 3 is not clear. Better description with the purpose of the figure will add more value.
We thank the reviewer for raising this issue. We introduced Figure 3 to show the evolution of size in drug-drug and drug-target interaction networks (i.e., as the number of drugs and targets) with the DrugBank versions. As shown, compared with the DDI and DTI sizes in the first available DrugBank version, the number of medicines/drugs almost quadrupled; in contrast, the number of targets increased at a much lower rate. This observation is in line with the other results reported in our paper, thus strengthening the overarching conclusion.

In the revised form of our manuscript, according to the reviewer's suggestion, we introduced Figure 3 with a clarifying statement. The statement now reads, "In Figure 3, panel a, we present the evolution of the number of nodes (i.e., drugs) in DDI networks; in Figure 3, panel b, we show the evolution of the number of nodes, drugs, and targets in DTI networks. As shown, from version 3.0 to 5.1.9, the number of medicines/drugs roughly quadrupled; in contrast, the number of targets increased at a much lower rate. Such an evolution suggests that, over the years, the complexity of processing and analyzing drug interaction networks grew considerably." In our revised manuscript, we highlighted this change with blue text, on page 7, section *Results*, subsection *Network metrics and centrality analysis*, paragraph 2.

Comment 5. The text can be more concise to improve the text quality especially in the discussion.

The reviewer is correct: our *Discussion* section needs better structure and wording. According to the suggestion, we performed the following steps:

1. We renamed the subsections of the *Discussion* as *Discussions on DDI networks* and *Discussions on DTI networks*.
2. We rewrite the entire *Discussion* section to eliminate redundant wording, thus bringing more conciseness and clarity to the manuscript.

Please find the revised form of the Discussion section in blue, starting on page 10.

GigaScience, 2022, 1–21

doi: xx.xxxx/xxxx
Manuscript in Preparation
Paper

PAPER

The curse and blessing of abundance—the evolution of drug interaction databases and their impact on drug network analysis

Mihai Udrescu[1,*], Sebastian Mihai Ardelean[1] and Lucreţia Udrescu[2]

[1]Department of Computer and Information Technology, Politehnica University of Timișoara, Vasile Pârvan Blvd.,300223, Timișoara, Romania and [2]Department I—Drug Analysis, "Victor Babeș" University of Medicine and Pharmacy Timișoara, Eftimie Murgu Sq., 300041, Timișoara, Romania

*mudrescu@cs.upt.ro

Abstract

Background
Widespread bioinformatics applications such as drug repositioning or drug-drug interaction prediction rely on the recent advances in machine learning, complex network science, and comprehensive drug datasets comprising the latest research results in molecular biology, biochemistry, or pharmacology. The problem is that there is much uncertainty in these drug datasets—we know the drug-drug or drug-target interactions reported in the research papers, but we cannot know if the not reported interactions are absent or yet to be discovered. This uncertainty hampers the accuracy of such bioinformatics applications.
Results
We use complex network statistics tools and simulations of randomly-inserted previously unaccounted interactions in drug-drug and drug-target interaction networks—built with data from DrugBank versions released over the last decade—to investigate whether the abundance of new research data (included in the latest dataset versions) mitigate the uncertainty issue. Our results show that the drug-drug interaction networks built with the latest dataset versions become very dense and, therefore, almost impossible to analyze with conventional complex network methods. On the other hand, for the latest drug database versions, drug-target networks still include much uncertainty; however, the robustness of complex network analysis methods slightly improves.
Conclusions
Our big data analysis results pinpoint future research directions to improve the quality and practicality of drug databases for bioinformatics applications: benchmarking for drug-target interaction prediction and drug-drug interaction severity standardization.

Key words: drug-drug interaction networks; drug-target interaction networks; drug database integrity; analysis robustness of drug networks

Background

Spurred by the fast development of efficient complex network analysis tools based on machine learning and the evergrowing drug/medicine databases we witnessed over the last decade, processing drug interaction networks became an appealing drug design method [1, 2]. Such drug interaction networks can represent various relationships or interactions involving active substances, e.g., drug-drug interactions, drug-target interactions, drug-gene interactions, drug-disease relationships, drug–adverse reaction relationships. As such, harnessing big amounts of data describing the intricate drug interactions (or relationships) with other entities

Compiled on: November 18, 2022.
Draft manuscript prepared by the author.

Key Points

- Many notable and helpful bioinformatics applications, such as drug repositioning and drug-drug interaction prediction, employ machine learning methods and statistical analysis on drug-drug and drug-target interaction network features (i.e., parameters/metrics and centralities).
- The main problem with the approach based on the drug interaction network analysis is that there is much uncertainty in drug databases: the reported drug-drug and drug-target interactions are certain, but it is uncertain if the non-reported interactions do not exist or are yet to be uncovered.
- Versioned drug databases—including DrugBank—record research results accumulated over the last decade, thus enabling network analysis that exposes the weak points and provides hints to improve the drug databases' practicality and the drug network analysis's accuracy.
- Our complex network analysis on the evolution of drug-drug and drug-target interaction networks shows that the drug-drug interaction networks made with the latest dataset versions have become too dense to analyze with established complex network methods.
- We do not notice the same density increase in drug-target networks; despite containing much uncertainty, the robustness of drug-target network analysis methods scarcely improves with the evolution of drug database versions.
- Our investigation provides guidance for future studies to improve the usefulness of drug databases and the accuracy of bioinformatics applications: standardization of drug-drug interaction labeling and delivering a comprehensive benchmark dataset for drug-target interaction prediction.

can lead to uncovering new drug properties: previously unknown drug-drug [3, 4, 5, 6] or drug-target interactions [7, 8, 9, 10, 11], drug repositioning [12, 13, 14].

Computational drug-drug interaction (DDI) prediction is critical in designing effective and safe drug combinations and developing drugs [5, 6]. Drug-target interaction (DTI) prediction is a key-step strategy in drug discovery that leads to uncovering new drugs for biological targets and new targets for known, approved drugs—representing a crucial step towards drug repositioning and identifying possible side effects [8, 11].

Drug repositioning (or repurposing) means finding new applications for drugs already in use [15]; the drug propensity for multiple functions underpins this undertaking. Before the upswing of big data and machine learning, pharmacologists and medical doctors mainly relied on serendipity to uncover drug repositionings [16]. The most illustrative example is aspirin, introduced as an antipyretic but—over time—revealed as having painkiller and antiplatelet effects. The prediction of drug-drug interactions and drug-drug interaction severity is another drug-interaction network analysis application with substantial benefits in therapeutic practice [17, 18, 19]. It is clear that predicting either adverse or synergistic interactions in drug-drug interaction networks help to tailor effective therapies for patients with multiple comorbidities.

In comparison with traditional drug design, drug repositioning entails simpler testing and validation procedures (because many adverse events and effects, as well as interactions with food and other drugs, are already known and tested), which translates into reduced costs and approval times [20, 21]. (In the FDA New Drug Therapy Approvals 2021, 50 drugs are are *new molecular entities* or *new therapeutic biologics*, and 17 are *drug repositionings* [22].) All these arguments make drug repositioning tempting for exceptional therapeutical cases, such as orphan diseases—for which there is insufficient research funding [23]—and new-pathogen epidemic diseases [24]—for which timing is essential. Particularly relevant is the case of the COVID-19 pandemic, where drug repositioning proved to be a valuable therapeutical method for quick public healthcare response, given that the conventional drug design requires a substantial amount of time. Indeed, many COVID-19 repositioning predictions uncovered with big data exploration and complex drug networks were confirmed by in vitro and in vivo experiments [25].

Despite the advances in machine learning, big data mining, complex network analysis, and the undeniable benefits of computational drug repositioning, the field still has significant problems. The most critical issue that affects the robustness of computer-based drug network analysis is that the drug interactions/relationships (e.g., with other drugs, targets, adverse reactions, or diseases) in drug databases primarily reflect what we know as positive information from in vitro and in vivo experiments. We have little negative information, i.e., interactions we know for sure that do not exist. If we do not have information about a specific drug interacting with a particular target, this does not mean that there is no way they interact; after all, "absence of evidence is not evidence of absence" [26].

Consequently, in computational drug repositioning, we do not have a robust ground truth to operate with; this can seriously affect the analysis accuracy of complex drug interaction networks. Mestres et al. have articulated this idea very eloquently [27] by showing how the network analysis based on degree centrality hierarchization of drugs in a network built with information from one database is affected by adding the information from another database. (In complex networks, a centrality expresses the importance of a network node/vertex; most network-based analysis approaches use either centrality-hierarchization or community detection methods.) Nonetheless, the enormous benefits of confirming (with in vitro and in vivo methods) even a few drug repositionings—uncovered by computational big data approaches—offset such accuracy problems.

As long as we do not have comprehensive negative information, the issue of uncertainty in drug datasets remains. However, the fact that—over the years—the comprehensiveness of drug datasets (such as DrugBank [28]) has constantly grown, and experts curated the data according to the latest literature results, may suggest that the integrity of the data improved. (The integrity characterizes the "absence of improper data alterations" [29].) Indeed, the constant growth of the most comprehensive drug database (reflected in the evolution of the various complex drug network parameters and centralities) may have mitigated the concerns formulated by Mestres et al. [27]. Our paper investigates how the evolution of knowledge on drug interactions mirrored by the DrugBank database, impacts the robustness of drug interaction network analysis based on centrality hierarchization methods; future research can exploit the insight we get to advance drug repositioning and drug-drug interaction prediction methods.

We build the drug-drug interaction (DDI) and drug-target interaction (DTI) networks with the data from DrugBank versions 3.0 to 5.1.9. Over the years, many research papers used DDI [30, 31, 32, 33, 34, 35] and DTI networks [36, 37, 38, 39, 40] for computational drug repositioning and drug-drug interaction pre-

diction. A drug-drug interaction occurs when one drug influences the action/effect of another drug in a biological environment. Such a drug-drug relationship signifies that one drug augments or, conversely, that mitigates the effect of the other one; either way, this generally translates into a clinically potential harmful situation. A drug-target interaction exists if the drug exerts a specific action upon a biological target (generally, a protein or enzyme), thus producing a pharmacological effect [40].

We found the motivation of our research in the visual inspection of the evolution of DDI and DTI networks over the years and across the successive DrugBank versions. Figure 1 shows how the DDI network density increased from version 3.0 to 5.1.9, via 5.0.8, such that the number of network clusters/communities substantially decreased; moreover, as shown in the panels below, the initial power-law degree distribution in DrugBank 3.0 DDI is altered in the subsequent versions. In contrast with the DDI network evolution presented in Figure 1, the equivalent evolution for DTI networks in Figure 2 does not increase the density substantially, and the structure does not change too much despite the increasing size of the networks—from version 3.0 to 5.0.8 and 5.1.9. Also, the panels below the DTI networks in Figure 2 show that the DTI degree distributions in both drugs and targets are power-law across all DrugBank versions. Indeed, this discrepancy between DDI and DTI network evolution over time (as reflected by the successive DrugBank data versions) inspires the study we present in this paper; the results obtained provide valuable insight for researchers developing big data techniques in systems and networks pharmacology, aiming at applications such as computational drug repositioning or drug-drug interaction prediction.

The contributions of our DDI and DTI network analysis across the DrugBank database versions are:

- We present for the first time the evolution of various complex network parameters and centralities [41], in drug-drug and drug-target interaction networks as the knowledge on drug-drug and drug-target interactions grew over more than a decade; we comment on the far-reaching consequences of data evolution on the computational analysis tools used in drug repositioning.
- We estimate the integrity of the processed data using Benford's law on the most prominent network centralities (i.e., degree and betweenness).
- We test the robustness of centrality-hierarchization analysis methods in drug interaction networks using our algorithm that automatically adds unknown interactions in ascending ratios to notice how this process affects the drugs' order of importance.

Accordingly, one main finding is that the DDI network parameters and centralities distributions were close to those of the typical complex networks in the earlier DrugBank versions but deviated markedly in the latest versions. Instead, the DTI parameters and network centralities distributions oscillate but remain close to the typical complex network ranges across all DrugBank versions. (We explain the typical characteristics of real-world complex networks in subsection Typical complex network characteristics.)

Such an evolution of the DDI networks owes to their enormous increase in density (i.e., many recent experimental results report new drug-drug interactions); however, typical complex networks are patently sparse. The overarching conclusion is that complex network analysis became irrelevant in the DDI networks built with the latest database versions data. Also, the integrity analysis of the DTI data finds that even the latest DrugBank versions still miss many unaccounted drug-target interactions; therefore, the robustness of the centrality hierarchization analysis (i.e., degree, as suggested in [27]) in DTI networks improved only marginally with the new data in the latest versions. Such a situation calls for an intensified effort to uncover new drug-target interactions; indeed, in 2021, only 17 new drug-target interactions were approved [42].

Data description

We chose the DrugBank dataset [28, 43, 44] because it is the most comprehensive and robust drug database, being curated manually by experts and scientists, according to the latest scientific discoveries reported in the literature; it is also versioned, which allows for analyzing the evolution of knowledge over time [28].

We downloaded all DrugBank versions recorded as XML files over a decade, from version 3.0 (January 2011) to version 5.1.9 (January 2022). Access to download the database versions is free with a validated account; an account can be created with an institutional email. The DrugBank versions 5.1.9 to 4.5.0 can be downloaded from `https://go.drugbank.com/releases` and 4.3 to 3.0 from `https://go.drugbank.com/downloads/archived`.

For each medicine, DrugBank lists many parameters, properties, and extensive information, such as the generic name, brand names, indications, type, drug categories, ATC codes, chemical structure and formula, chemical identifiers, associated conditions, pharmacodynamics, mechanism of action, metabolism, toxicity, pathways, drug interactions, food interactions, clinical trials, patents, targets, enzymes, carriers, transporters, and so forth. Nonetheless, our present study analyzes the evolution of drug-drug and drug-target interaction networks (DDI and DTI), such that the only information we need from each drug in all DrugBank versions is *drug interactions* and *targets*. Also, we included information concerning *approved* human drugs only (consequently, we excluded the investigational, experimental, withdrawn, or vet-approved drugs).

For every drug in each database version, DrugBank lists the drugs with which it interacts; for example, in DrugBank 5.1.9, ibuprofen has a list of 1,236 approved interacting drugs. Each interaction has a text description; for the ibuprofen entry, the first interacting drug is abacavir, and the corresponding description reads, "Ibuprofen may decrease the excretion rate of Abacavir which could result in a higher serum level." At the same time, all DrugBank versions provide, for each drug entry, a list of interacting targets. For each interacting target, we find the following information: kind, organism (humans, in our case), pharmacological action, actions, general function, specific function, gene name, Uniprot ID, Uniprot name, molecular weight, and a list of references (i.e., scientific papers) to support the provided details experimentally. To continue with our example, DrugBank 5.1.9—the latest database version—lists 10 targets for ibuprofen (all proteins), from Prostaglandin G/H synthase 2 to Protein S100-A7.

Problem formulation

Complex networks

A *complex network* is a graph G consisting of a node (or vertex) set V and a link (or edge) set E, $G = (V, E)$, where any edge $e_{ij} \in E$ connects nodes $v_i, v_j \in V$. Additionally, compared to normal graphs, real-world complex networks have a large number of nodes $|V|$ (i.e., hundreds to millions) and intricate, nontrivial topologies (i.e., links do not form regular structures but are not completely random either). [45, 46, 41, 47]. If the nodes $v_i \in V$ are of the same type, G is a *monopartite* network; if any node v_i belongs to one of the m disjoint node subsets ($V_1 \cup V_2 \ldots \cup V_m = V$), then the network is *multipartite* or m-partite.

The network is *unweighted* if the weights of its edges/links $e_{ij} \in E$ are $w_{ij} = 1$. (In other words, if $w_{ij} = 1$ we have a link between nodes v_i and v_j, and if $w_{ij} = 0$ there is no link between v_i and v_j.) The network is *weighted* if w_{ij} are not binary values (i.e., $w_{ij} \in \mathbb{R}$); values $w_{ij} = 0$ also correspond to non-edges between v_i and v_j. We say that the network is *directed* if $\exists i, j\ (i \neq j)$ such that $w_{ij} \neq w_{ji}$. Unweighted networks are directed if $\exists e_{ij} \in E$ with $w_{ij} = 0$ and $w_{ji} = 1$.

Such networks G can also be expressed as adjacency matrices W containing elements w_{ij}, with $w_{ij} = 0$ when there is no link/edge

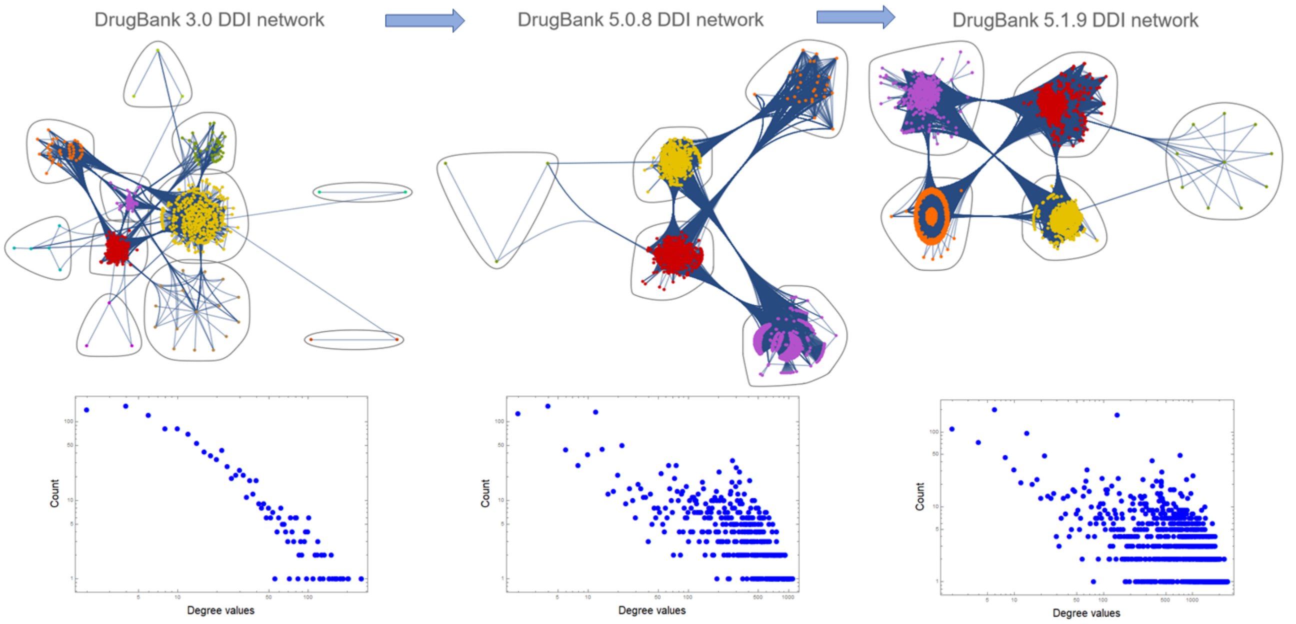

Figure 1. Drug-drug interaction (DDI) network evolution with the DrugBank data versions, showing the massive increase in density from version 3.0 to 5.1.9. Consequently, the number of network clusters/communities generated with hierarchical clustering in Mathematica 13.0 substantially decreases (nodes represent drugs, links represent drug-drug interactions, and node colors represent the distinct clusters/communities of network nodes). Also, the panels below show how the increase in density alters the power-law degree distribution in the DDI networks corresponding to the latest DrugBank versions.

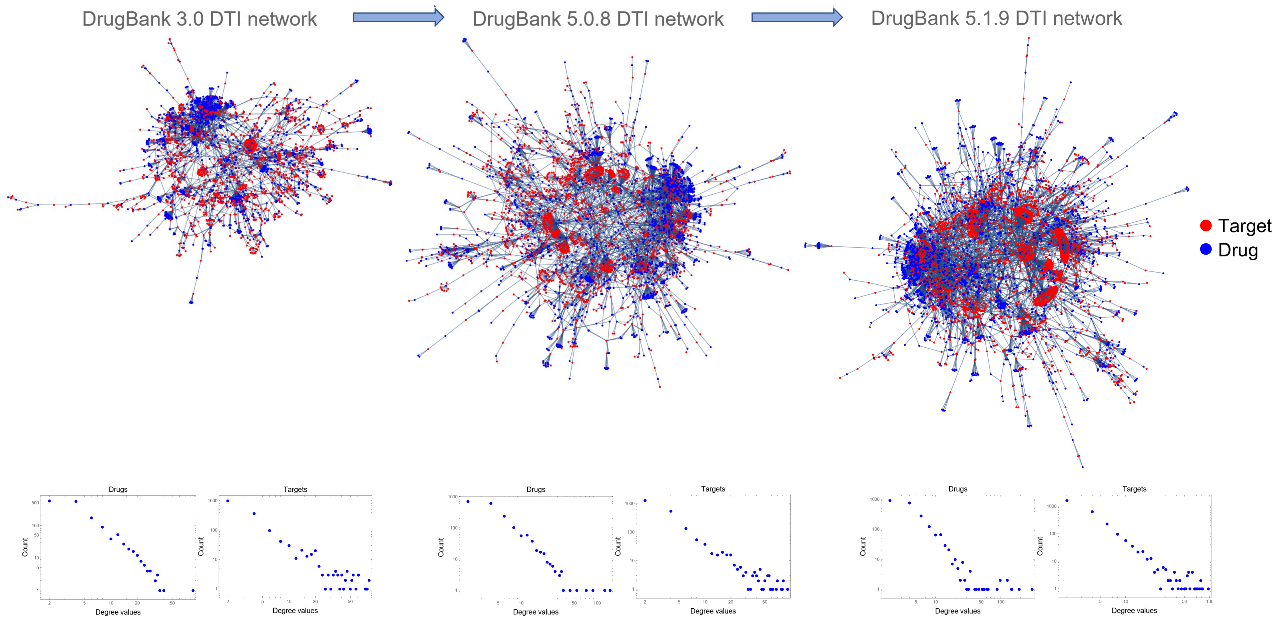

Figure 2. The evolution of drug-target interaction (DTI) bipartite networks with the successive DrugBank data versions, where the red nodes represent biological targets, the blue nodes represent drugs, and the links represent drug-target interactions. Although the DTI network sizes increase with the successive DrugBank data, the network structure and the degree distributions in both drugs and targets (see the panels below) are not altered substantially.

between nodes v_i and v_j. In unweighted networks, $w_{ij} \in \{0, 1\}$, and in weighted networks $w_{ij} \in \mathbb{R}$; furthermore, in undirected networks, elements in the adjacency matrix are symmetric relative to the first diagonal, i.e., $w_{ij} = w_{ji}$.

The *multi-layered* networks generalize the network concept by considering multiple types of edges/links so that the network can be described by several adjacency matrices W^q ($q \in \{1, 2, \ldots Q\}$), with elements w^q_{ij} for each of the Q types of links. Moreover, when acknowledging that the link structure (i.e., the network topology) changes over time t, the network is expressed as a function of time, $w^q_{ij}(t)$.

Drug networks

To mine the complexity of drug interactions in the biological environment, researchers use database information to build drug networks such as drug-drug, drug-target, and drug-disease interaction networks, as well as drug-disease and drug–adverse effects association networks. This paper considers drug-drug interaction networks (complex drug networks where nodes represent drugs) and drug-target interaction networks (bipartite networks where one type of node represents drugs, and the other represents biological targets). Links represent drug-drug and drug-target interactions in the DDI and DTI networks. We do not consider drug-drug

interaction type and strength. Consequently, the DDI networks are undirected, unweighted, and monopartite. Also, the drug-target interactions have no information regarding their strength, making DTI networks directed (from drug to target), unweighted, and bipartite.

Using the complex network formalism, in DDI networks, $\forall i, j, i \neq j, v_i \in V = \mathcal{D}$ and $e_{ij} \in E = \mathcal{I}\left(e_{ij} \neq 0\right)$, where $\mathcal{D}$ is the set of drugs and $\mathcal{I}$ the set of drug-drug interactions. In DTI networks, we have $v_i \in V = V_{\mathcal{D}} \cup V_{\mathcal{T}}$ (where $V_{\mathcal{D}}$ is the set of nodes representing drugs and $V_{\mathcal{T}}$ is the set of nodes representing targets) and $e_{ij} \in E = \mathcal{A}$, where $\mathcal{A} = \left\{e_{ij} | v_i \in V_{\mathcal{D}}, v_j \in V_{\mathcal{T}}, i \neq j, e_{ij} \neq 0\right\}$ represents the set of drug-target interactions). Additionally, the set of non-edges in the DDI networks is $\bar{\mathcal{I}} = \left\{e_{ij} | v_i, v_j \in \mathcal{D}, i \neq j, e_{ij} = 0\right\}$, and in the DTI is $\bar{\mathcal{A}} = \left\{e_{ij} | v_i \in \mathcal{D}, v_j \in \mathcal{T}, i \neq j, e_{ij} = 0\right\}$.

A non-edge in a DDI network ($\bar{e}_{ij} \in \bar{\mathcal{I}}$) or a DTI network ($\in \bar{\mathcal{A}}$) represents a drug-drug or a drug-target interaction that has a probability $p \in (0, 1)$ of being discovered in the future (i.e., missing information generating uncertainty). If $p = 0$, we are sure that interaction between v_i and v_j is impossible (i.e., the negative information on drug interactions).

The DDI and DTI networks are analyzed and processed with computational tools to acquire new insight and knowledge in areas such as drug repurposing, drug-drug interaction prediction, drug-target interaction prediction, designing synergistic drug combinations, and optimizing drug efficacy.

As this paper considers the main types of drug networks, namely drug-drug and drug-target interaction networks (or interactomes), we acknowledge two main issues:

i. The networks are built according to what we know (in terms of drug-drug or drug-target interactions) at the time when the database is recorded. Over time, some new information may be added, and some may be filtered according to the experimental biological findings.

ii. The uncertainty in building the drug networks, mainly represented by missing links between nodes—drug-drug or drug-target interactions that exist but are still uncovered—may affect the conclusions of drug network analysis methods. Previous research also expresses this important concern [27].

Accordingly, our investigation is set to answer two fundamental questions.

1. We already know the main topological metrics statistics in complex networks (biological, social, or technological) where the degree of uncertainty is smaller than in drug networks [46]. Thus, how do the drug network parameters evolve with the drug database versions compared to the typical complex network topological characteristics, such that they foster an effective and efficient computational complex network analysis?

2. Newer drug databases contain more information on both drug-drug and drug-target interactions. How did the robustness of the drug networks evolve after adding more knowledge?

To address question 1 we analyze the evolution over time of drug-drug and drug-target networks $w_{ij}(t)$ (where t are discrete-time moments when the drug database version was released) for parameters such as average degree, diameter, average path length, and average clustering coefficient. We analyze the same type of evolution for the distributions of centrality metrics such as degree, betweenness, closeness, eccentricity, and page rank.

To answer question 2, we implement a robustness test algorithm that adds/injects links representing potential unknown interactions to the known network, $w_{ij}(t) + u_{ij}$ (where u_{ij} represents the unknown links), and then represent the evolution of the ordinal correlation (Kendall τ) between the node rankings in $w_{ij}(t)$ and $w_{ij}(t) + u_{ij}$ with the rate of unknown links (expressed as the number of nodes in u_{ij} divided by the number of nodes in $w_{ij}(t)$). The rationale for our approach is that the analysis of node centrality rankings underpins most network-based bioinformatics methods and applications.

Methods

In this section, we describe the statistical and computational methods employed in this paper: subsection Network analysis introduces the main metrics and centralities required for the computational and statistical analysis of complex drug networks; subsection Database quality presents the statistical methods we use to test the drug database integrity; subsection Network analysis robustness explains the computer simulation method we propose to assess the robustness of the centrality-based drug network analysis strategies; subsection Computational methods describes the tools we use in this paper to implement the required computational and statistical techniques.

Network analysis

Network parameters and metrics

The *degree* of a node $v_i \in V$ in an undirected network G is defined as $d(v_i) = \sum w_{ij}$. In directed networks, we can compute the in-degree and out-degree of v_i as $d^I(v_i) = \sum w_{ij}$ and $d^O(v_i) = \sum w_{ji}$, respectively. Then, the average degree of network $G = (V, E)$ is

$$\langle d \rangle = \frac{1}{|V|} \sum_{v_i \in V} d(v_i) \quad (1)$$

where $|V|$ is the number of elements in V, namely the number of nodes in network G. If G is directed, we can compute the average in-degree and out-degree as $\langle d^I \rangle = \frac{1}{|V|} \sum_{v_i \in V} d^I(v_i)$ and $\langle d^O \rangle = \frac{1}{|V|} \sum_{v_i \in V} d^O(v_i)$. When the network G is multipartite, we can also compute the average degree for each node type V_j ($V_j \subset V$) as $\langle d_{V_j} \rangle = \frac{1}{|V_j|} \sum_{v_i \in V_j} d(v_i)$.

The *clustering coefficient* of a node v_i is the number of existing links between nodes directly connected to v_i divided by the total number of possible links,

$$c(v_i) = \frac{2\left|\left\{e_{jk} | j, k \in L_i\right\}\right|}{|L_i|\left(|L_i| - 1\right)}, \quad (2)$$

with L_i representing the set of nodes directly linked to v_i. The average clustering coefficient of network G is

$$\langle c \rangle = \frac{1}{|V|} \sum_{v_i \in V} c(v_i). \quad (3)$$

The *network density* is the ratio between the number of links/edges in G (i.e., $|E|$) and the maximum number of possible links,

$$r = \frac{2|E|}{|V|\left(|V| - 1\right)}. \quad (4)$$

The network G is a *connected graph* if there is a path between any two nodes $v_i, v_j \in V$; otherwise, G has multiple components (a component is a connected subgraph). In general, there can be many paths between two nodes v_i and v_j in a connected network or component; we denote the length of the shortest one $s(v_i, v_j)$. Then,

the average path length in G is

$$\langle s \rangle = \frac{2}{|V|\,(|V|-1)} \sum_{v_i, v_j \in V} s(v_i, v_j). \tag{5}$$

The *diameter* of a network G is the biggest shortest path between any two nodes $v_i, v_j \in V$,

$$\phi = \max_{v_i, v_j \in V} \left\{ s(v_i, v_j) \right\}. \tag{6}$$

Network centralities

A *node centrality* $\mathcal{C}$ is an attribute or metric that characterizes the importance of a node in the network; many studies in biological networks use centralities to rank nodes. The simplest centrality of node v_i is the *degree* $d\,(v_i)$, as the number of links (or the weight) associated with the node indicates its importance [48, 49, 50].

The *betweenness* centrality characterizes the node's role in connecting communities or clusters of nodes. (Such node clusters are often associated with specific functionality in biological networks, particularly drug networks [51, 52, 35, 25].) The betweenness of v_i is the number of paths (shortest or random walks) between all node pairs in G that cross v_i (normalized by the total number of node pairs in G),

$$b\,(v_i) = \sum_{v_j, v_k \in V;\, i,j \neq k} \frac{2\sigma_{j,k}\,(v_i)}{|V|\,(|V|-1)} \tag{7}$$

where

$$\sigma_{j,k}\,(v_i) = \begin{cases} 1 & \text{if } \exists\, s(v_j, v_k) \text{ that crosses } v_i \\ 0 & \text{otherwise}. \end{cases} \tag{8}$$

The *closeness* centrality measures how close the node v_i is to the other nodes; it is the inverse of the sum of shortest paths to all other nodes in V,

$$\gamma\,(v_i) = \left(\sum_{v_j \in V \setminus \{v_i\}} s(v_i, v_j) \right)^{-1}. \tag{9}$$

The *eigenvector* centrality assumes that the degree of a node v_i does not particularly determine its importance; instead, the importance of nodes directly connected to v_i is key. For node v_i, the eigenvector centrality value $\eta\,(v_i)$ is defined as

$$\eta\,(v_i) = \frac{1}{\lambda} \sum_{v_j \in L_i} \eta(v_j) = \frac{1}{\lambda} \sum_{v_j \in V} \eta(v_j) w_{ij} \tag{10}$$

where λ is a constant, w_{ij} is an element in the adjacency matrix W, and $\eta\,(v_j)$ is the eigenvector centrality value of nodes v_j directly connected to v_i. Because the definition in equation 10 is recursive, finding the eigenvector centrality values for all nodes in V requires solving the equation $W\eta = \lambda\eta$ using linear algebra [53]. (W is the adjacency matrix, and η is the vector of eigenvector centrality values for all nodes in V.)

Typical complex network characteristics

Generally, in real-world complex networks, the density of links is low (i.e., $\sim \frac{1}{n}$ because the number of links is of the order n, where n is the number of nodes, and far from the order n^2 corresponding to a completely connected network); the total number of links also determines the average degree, and the average degree is significantly lower than n; the degree follows either a power-law (exponent is between 1 and 3) or normal distribution; the average path length is between 3 and 10; the clustering coefficient is larger than $\frac{1}{n}$ but considerably lower than 1 [46, 54].

Database quality

The drug-drug and drug-target data quality in databases such as DrugBank are degraded by uncertainty, as they contain curated information from published studies, papers, and clinical trials. The ever-growing volume of empirical results that underpin drug databases can be affected by errors generated by improper data handling, academic misconduct, or imbalanced research focus (e.g., one may expect an abundance of new data on SARS-CoV-2 target data, but less so for rare diseases). These problems related to data quality in drug databases are adequately acknowledged in the literature, along with the systematic counter-measures [27, 55]. One such analytical approach to validate data quality is checking various parameter distributions against Benford's law [55]. The law of the first digit—or Benford's law—states that the natural distribution of the first digit f of a real-world variable (covering a wide range of values) is

$$P\,(f) = \log_{10}\left(1 + \frac{1}{f}\right), \tag{11}$$

where $f \in \{1, 2, \ldots 9\}$. This means that the probability of first digit being '1' is the highest, with the probabilities of the folowing digits probabilities decreasing logarithmically.

In this paper, we check Benford's law for the network centralities to verify data quality, meaning that we consider f the first digit of network centralities $\mathcal{C}$. For complex network centralities, a similar approach is described in [56], in the case of social networks. To the best of our knowledge, no similar study was performed in biological networks, although we find proven applications of Benford's law in systems biology [57, 58].

Network analysis robustness

The drug-drug and drug-target interactions we take from DrugBank are well documented and proven by experiment, but we can assume some unknown interactions are yet to be investigated or tested. To analyze the robustness of network analysis tools, we draw inspiration from the paper [27], where the authors added to the drug-target network generated with DrugBank new drug-target interactions (i.e., unaccounted by DrugBank) reported in another database; this way, they analyze how the centrality distributions change with the added information. In this paper, we adopt a systematic approach to study the robustness of both the network structure and the specific centralities by randomly adding unknown links (i.e., drug-drug or drug-target interactions) with a rate q, and then analyze the structural changes that manifest through modifications in the node rankings. We measure the structural changes incurred by adding the previously unknown links in two ways. First, we target the centralities with a power-law distribution (e.g., degree, betweenness) to compute the difference between the log-log distribution slopes α. Second, we compute the Kendall τ correlation between the node rankings according to centrality $\mathcal{C}$ before and after adding the unknown links ($\tau = 1$ indicates a perfect monotonous correlation, $\tau = 0$ indicates no monotonous correlation).

By representing τ as a function of q (with q going from a very small value to 0.1), we can analyze the robustness of the network structure: rapid decay of τ as q increases indicates that the node ranking according to $\mathcal{C}$ is fragile to the uncertainty of new links being present; conversely, slow decay of τ suggests a robust node ranking. We present the algorithmic description of our robustness test in Algorithm 1, where E_t represents the set of all possible edges in G and E_u is the set of previously unknown edges (accordingly, $E_t \setminus E$ is the set of non-existing edges in G). As this is a random

Algorithm 1 Analyze the variation of Kendall τ between node rankings and power-law distribution slope α (for node centrality $\mathcal{C}$) in network G and network with unknown edges G'.

Input: Network $G = (V, E)$.
Output: Representation of Kendall τ and power-law distribution slope α against unknown edge rate q.

$E_t \Leftarrow \left\{ e_{ij} = (v_i, v_j) | v_i, v_j \in V; i < j \right\}$
for r in 0 to R **do**
  for q in range (0.001 to 0.1) **do**
    $E_u \Leftarrow \emptyset$
    for all $e_{ij} \in E_t \setminus E$ **do**
      $E_u \Leftarrow E_u \cup \left\{ e_{ij} \right\}$ with probability q
    end for
    $E' \Leftarrow E \cup E_u$
    $G' \Leftarrow (V, E')$
    $l_V \Leftarrow$ list of nodes in V, descending order after $\mathcal{C}$
    $l'_V \Leftarrow$ list of nodes in V', descending order after $\mathcal{C}$
    $\tau_q \Leftarrow$ Kendall $\tau \left(l_V, l'_V \right)$
    $\alpha_q \Leftarrow$ Power-law distribution slope of $\mathcal{C}$ in G'
  end for
end for=0

simulation, we repeat it for each G and q (R times in Algorithm 1) to compute the average and variance for α and Kendall τ. (For the simulations presented in this manuscript, we used $R = 100$.)

Computational methods

To foster the reproducibility of our analysis, we provide all the necessary tools—Jupyter Notebook, Python, and WolframScript—as buildable Docker containers in the `https://github.com/research-hyperion/Drug_database_statistics` repository. We automatically install all the tools needed for reproducing our methods in a Linux container and provide a shell script for automatically running the analysis. The activation of WolframScript for running the Mathematica notebooks requires a Wolfram account. DrugBank versions recorded as XML files must be downloaded, as mentioned in Section Data description, in the root of the cloned repository in the DrugBank directory. Each XML file must be saved following a naming convention to avoid file overwriting; therefore, each DrugBank XML file is saved as *drugbank_version.xml* in the DrugBank directory.

To create the Docker image run the command **docker build –build-arg wolframId=<wolfram account email> –build-arg wolframPass=<wolfram account password> -t hyperion**. The image will take several GB of storage. The *wolframId* and the the *wolframPass* are the Wolfram account credentials needed to activate the WolframScript. After creating the container, run the command **docker run -t -i hyperion /bin/bash** in the terminal window to start an interactive shell in the Docker container.

We created a shell script that allows the execution of the Jupyter notebooks that build the DDI and the DTI networks and the network robustness analysis for each DrugBank version. As presented in Section DTI network robustness, due to the high complexity of the robustness analysis and huge computational burden, the script must provide the options to build and analyze the DDI and DTI networks or to perform the network robustness analysis. Invoking the script with the "-i" parameter will build and analyze DDI and DTI networks for each DrugBank version; invoking with the "-s" parameter will build the networks and run the robustness analysis.

The DDI and DTI network analyses are performed by 3 Jupyter Notebooks, namely *parse_DrugBank*, *parse_DrugBank-DDI*, and *parse_DrugBank-DTI*; if run in the mentioned order, we parse the DrugBank XML files, build and analyze the DDI and DTI networks. The robustness analysis depends on the files created by the mentioned notebooks to perform the algorithmic analysis presented in Section Network analysis robustness.

Results

This section analyzes two types of networks built from DrugBank data: drug-drug interaction networks DDI (where nodes v_i represent drugs and links represent drug-drug interactions) and drug-target networks DTI (nodes represent both drugs and targets and links represent drug-target interactions).

Many papers that employ complex network science for drug repurposing, drug interaction prediction, or adverse effect prognosis use other, more sophisticated drug networks [12, 13, 1, 33, 59](multi-partite networks, drug similarity networks, etc.) However, the straightforward DDI and DTI topologies are representative of the complexity issues at hand, and most of the more elaborated networks (such as the weighted similarity networks) can be derived from DDI and DTI structures through multi-partite network projection [60].

Network metrics and centrality analysis

Our analysis follows the evolution of metrics and centralities in DDI and DTI networks built with DrugBank information—from version 3.0 to version 5.1.8 (January 2011–January 2021).

In Figure 3, panel a, we present the evolution of the number of nodes (i.e., drugs) in DDI networks; in Figure 3, panel b, we show the evolution of the number of nodes, drugs, and targets in DTI networks. As shown, from version 3.0 to 5.1.9, the number of medicines/drugs roughly quadrupled; in contrast, the number of targets increased at a much lower rate. Such an evolution suggests that, over the years, the complexity of processing and analyzing drug interaction networks grew considerably. In both DDI and DTI networks, the number of drugs and targets is not the same as the total number of drugs and targets in the respective DrugBank versions because some drugs and targets have no known interactions.

We also notice the significant discrepancy between the number of links in DDI and DTI networks (Figure 4 (panels a and b, respectively), which determine the evolution of DDI and DTI density evolution in Figure 4, panels c and d.

Comparing the average path length and diameter in the DDI and DTI networks further emphasizes the observed discrepancies (see Figure 5 a and b); the same remark holds for the average degree in Figure 6 (panels a and b for DDI and DTI, respectively).

The DTI networks are bipartite; therefore, any link/edge connects one drug with one target. Consequently, DTI networks have a clustering coefficient of 0. Figure 7 presents the evolution of the average clustering coefficient $\langle c \rangle$ in DDI networks.

Like many other natural complex networks [46, 61], the DDI and DTI networks are scale-free, meaning that their node degree distribution is a power-law $P(d) \propto d^{-\alpha}$. Figure 8 presents the evolution of node degree's power-law distribution exponent α in DDI and DTI networks. (DTI is a bipartite directed network, and we also present the in-degree and out-degree distributions for targets and drugs, respectively.)

The analytical results presented in this section indicate that the DDI networks have become highly dense ($r = 0.1238$ with a huge $\langle d \rangle = 530.8455$ for DrugBank 5.1.8), with small $\langle s \rangle$ and ϕ and an unusually large α. Although the clustering coefficient $\langle c \rangle$ is high, detecting node communities or ranking nodes with centralities—standard network analysis techniques in network pharmacology [12, 14, 25]—becomes irrelevant because of the high link density. Conversely, the metrics and centrality statistics of the DTI networks are typical for biological scale-free complex networks [46, 54]: small $\langle s \rangle$ (but not smaller than 6), and an exponent α around 3. Therefore, the DTI network topologies foster the employment of

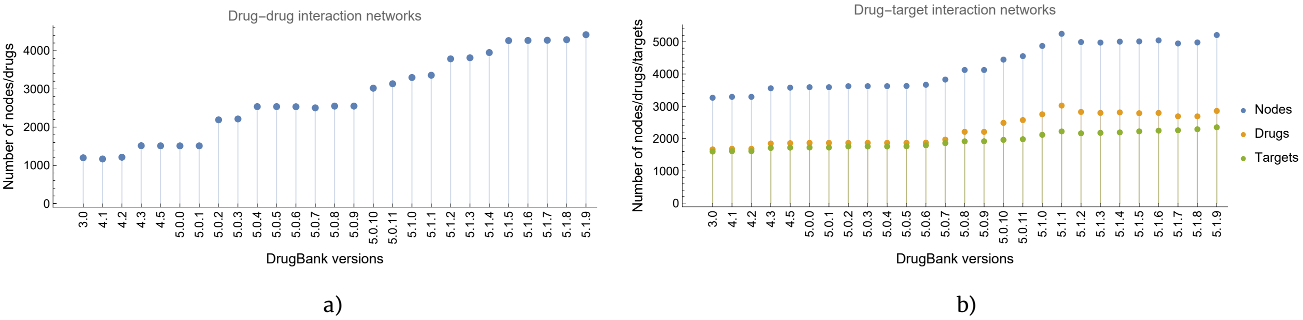

Figure 3. The evolution of nodes $|V|$ in drug-drug (representing drugs $|V_{\mathcal{D}}|$, in panel a) and drug-target interaction networks (representing drugs $|V_{\mathcal{D}}|$ and targets $|V_{\mathcal{T}}|$, in panel b) built with information from DrugBank versions 3.0 to 5.1.9. The number of drugs in the DDI evolves from 1198 for version 3.0 to 4417 for version 5.1.9; the increase is steady, although some DrugBank versions abruptly put on more drugs so that several ensuing versions filter the added information. The number of drugs and targets in the DTI evolves respectively from 1166 and 1599 in version 3.0 to 2857 and 2350 in version 5.1.9; because the DTI network is bipartite, the number of DTI nodes is the sum of drugs and targets.

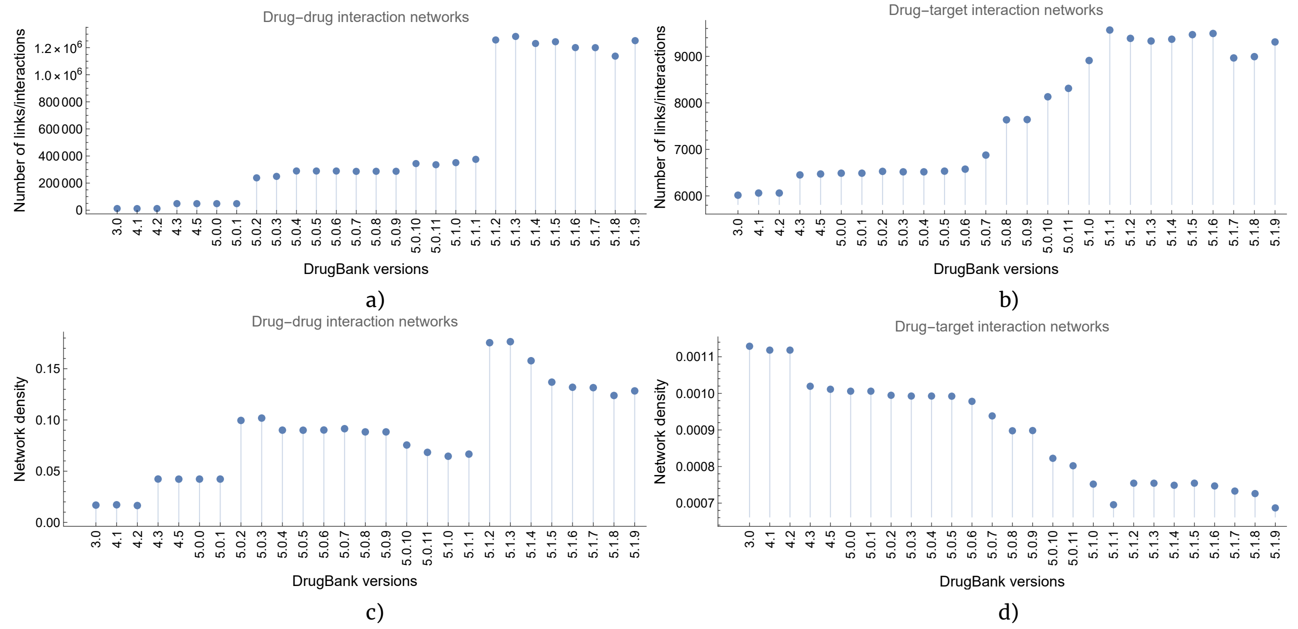

Figure 4. The number of links $|E|$ evolution in drug-drug and drug-target interaction networks, built with information from DrugBank versions 3.0 to 5.1.9 (panels a and b, respectively). The number of links in the DDI evolves from 12,089 in version 3.0 to 1,252,028 in version 5.1.9; in the DTI, it increases from 6,015 to 9,310. Panels c and d respectively present the evolution of density r in drug-drug and drug-target interaction networks, built with information from DrugBank versions 3.0 to 5.1.9. The density in the DDI networks evolves from 0.0186 in version 3.0 to 0.128377 in version 5.1.9; some versions abruptly increase the density (by adding many interactions), while the following versions filter the interactions and decrease the density (e.g., the density evolution from 5.1.1 to 5.1.9). In the DTI networks, the density evolves from 0.0011 in version 3.0 to 0.000687 in version 5.1.9; as shown, the density in DTI networks decreases with the newer database versions.

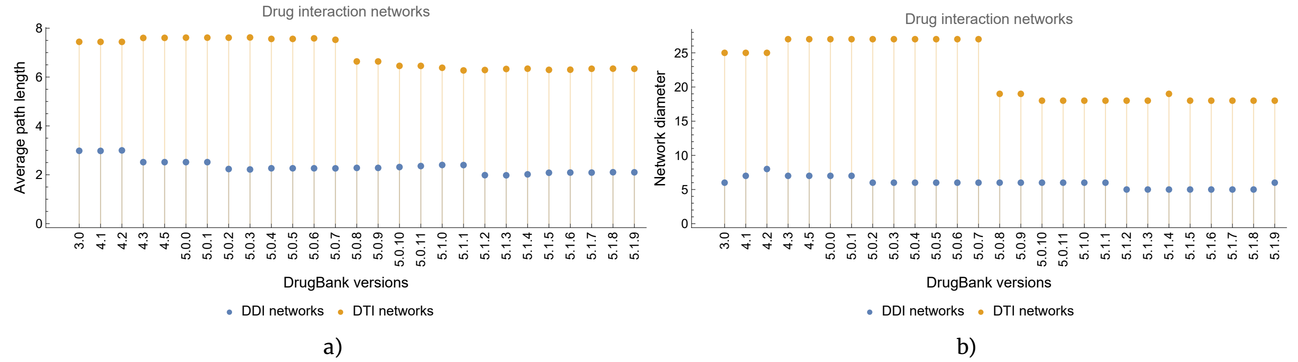

Figure 5. The evolution of average path length $\langle s \rangle$ (panel a) and diameter ϕ (panel b) in drug-drug and drug-target interaction networks, built with information from DrugBank versions 3.0 to 5.1.9. The value of $\langle s \rangle$ evolves from 2.98 in version 3.0 to 2.1 in version 5.1.9. for DDI, and from 7.44 to 6.338 in DTI. The value of ϕ evolves from 6 in version 3.0 to 6 in version 5.1.9. for DDI, and from 25 to 18 in DTI.

community detection and other specific network analysis techniques.

Moreover, upon visual inspection, Figure 8 indicates that as the DDI networks become denser across the DrugBank versions, their degree exponent α becomes > 4, i.e., too big to correspond to real-world power-law distributions [46]. Indeed, the visual representa-

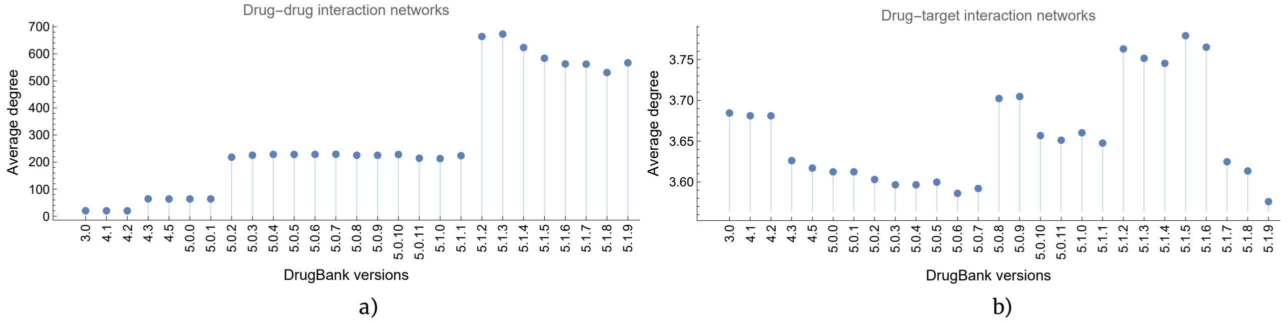

Figure 6. The evolution of average degree $\langle d \rangle$ in drug-drug and drug-target interaction networks (panels a and b, respectively), built with information from DrugBank versions 3.0 to 5.1.9. For DDI networks, the value of $\langle s \rangle$ increases from 20.181 in version 3.0 to 566.913 in version 5.1.9; for DTI networks, it oscillates from 3.684 to 3.576.

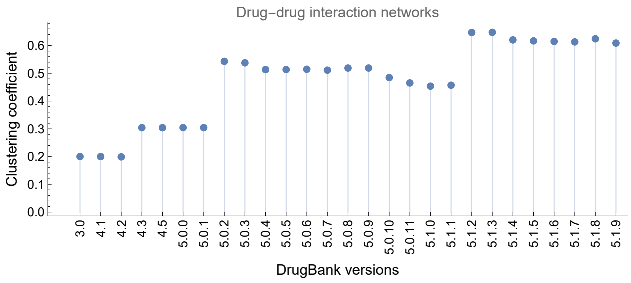

Figure 7. The evolution of average clustering coefficient $\langle c \rangle$ in drug-drug interaction networks, built with information from DrugBank versions 3.0 to 5.1.9. The value of $\langle c \rangle$ increases from 0.199 in version 3.0 to 0.609 in version 5.1.9.

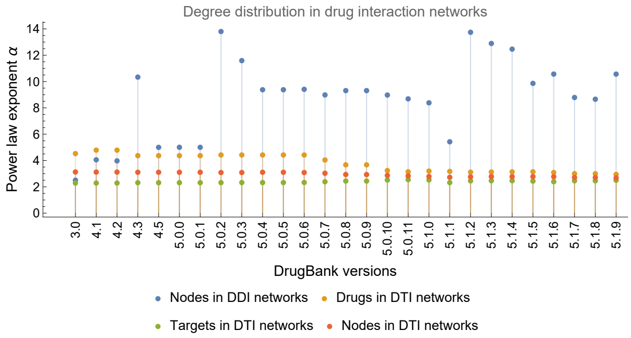

Figure 8. The evolution of the α exponent of the power-law degree distribution in drug-drug and drug-target interaction networks, built with information from DrugBank versions 3.0 to 5.1.9. In DDI networks, the α values are very high—specific for dense networks. In DTI networks, we present the overall degree distribution of the nodes and the in-degree and out-degree distributions, as the DTI network is bipartite and directed. (The connected nodes representing drugs have non-zero out-degrees and zero in-degrees, whereas nodes representing targets have zero out-degrees and non-zero in-degrees.)

tion of the degree and betweenness distributions in the DrugBank 3.0 DDI network show typical power-laws (Figure 9, panels a and c), whereas the DrugBank 5.1.9 counterparts indicate that only the betweenness distribution is not substantially altered (Figure 9, panels b and d).

We observe the same degradation for the other centrality distributions in DDI networks from the earlier versions of the drug database to the latest ones. In Figure 10 panels a, b, and c, we present the eigenvector, PageRank, and closeness distributions in the DrugBank 3.0 DDI network; in Figure 10 panels d, e, f, we visualize these distributions in the DrugBank 5.1.9 DDI network.

Conversely, in the DTI networks, we do not notice the same degradation of power-law degree and betweenness distributions from DrugBank 3.0 to 5.1.9 (see Figure 11). We see the same tendency not to alter the distributions in DTI networks across all DrugBank versions for eigenvector, PageRank, and closeness centralities (as presented in Figure 12).

Network centralities and Benford's law

We check if the distributions of centralities abide by Benford's law in drug-drug and drug-target networks. When data distribution in natural occurring datasets closely resembles the Benford distribution, we assume a high drug interaction data quality, generating robust analysis results.

We use Pearson's chi-squared (χ^2) test to measure the distance between degree and betweenness centrality distributions and the theoretical Benford distribution in DDI networks across all DrugBank versions, as indicated in [56]. However, as argued in [63], Pearson's χ^2 is often misused in such analysis cases; therefore, as suggested in this reference, we also use the Wasserstein distance and the sum of squared deviations between distributions. For these three metrics, a smaller distance to Benford's distribution means that the empirical centrality distribution in the drug network is more compliant with Benford's law of the first digit. Apart from the numerical analysis, we also used graphical representations of the distributions, including Q-Q plots, to check if the degree and betweenness distributions in DDI networks abide by Benford's law.

DDI networks

We notice by the visual inspection of Figures 13 and 14, that the compliance with Benford's law has degraded over the years (i.e., with the evolution of DrugBank versions) in DDI networks, especially for the degree distribution. Using the Wasserstein distance, the sum of squared deviations, and Pearson's χ^2, we show the evolution of distance between the theoretical Benford distribution and the empirical distribution of degree (Figure 15, panels a and b) and betweenness centrality (Figure 15, panels c and d) distributions in DDI networks across all DrugBank versions. (We represented Pearson's χ^2 separately because its range of values is larger than the other distance metrics.)

DTI networks

For the degree, Figures 16 and 17 offer a visual comparison between the first and the latest DrugBank versions; it shows that the empirical degree is far from Benford's theoretical distribution in all database versions. For the betweenness, Figure 18 presents the comparison between the theoretical Benford first digit distribution and the betweenness in DrugBank 3.0 and 5.1.9 DTI networks.

The overarching conclusion in DTI networks is that the degree is not compliant with Benford's law, but the betweenness is relatively compliant; these properties do not change across the DrugBank versions.

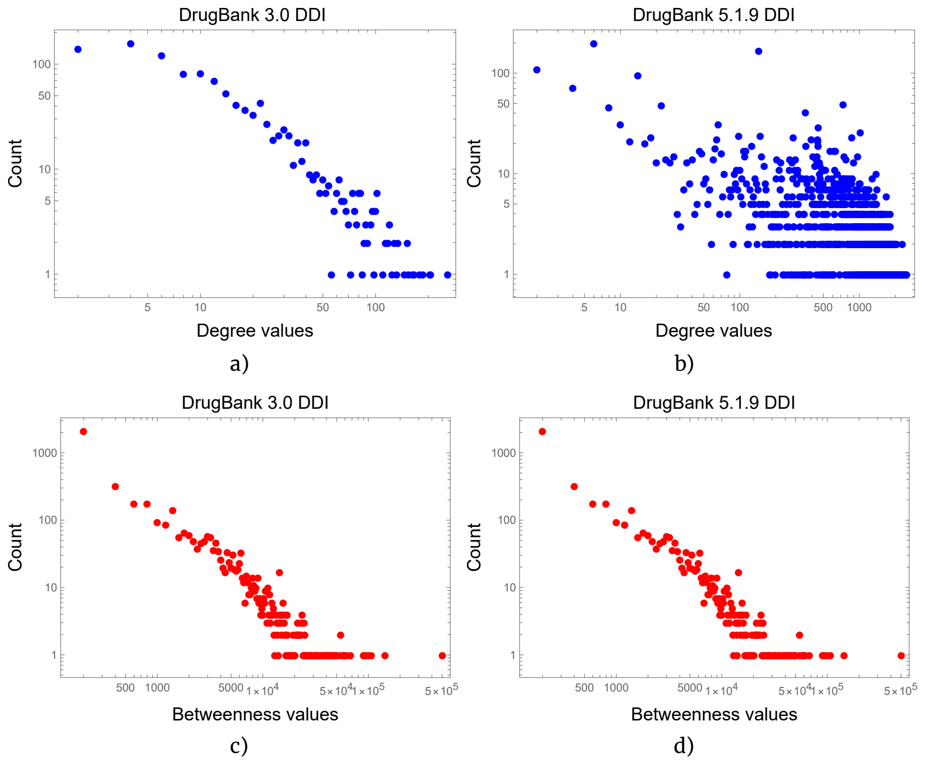

Figure 9. The degree and betweenness distributions in the drug-drug interaction (DDI) networks, built with information from the first and latest DrugBank versions. In panels a and c, we present the degree and betweenness distributions in the DrugBank 3.0 DDI network, whereas panels b and d show the DrugBank 5.1.9 counterparts. (We analyzed and calculated these distributions with the Powerlaw Python package [62].)

DTI network robustness

The algorithmic evaluation of network robustness, described in Section Network analysis robustness, entails running $R = 100$ times the following steps sequentially: adding random edges to the drug network according to a rate of unknown interactions q, determining the degree and betweenness centralities of all nodes in networks G and G' from Algorithm 1, sorting vertices/nodes in G and G' after their centralities, calculating the Kendall τ between the node hierarchies in G and G' after sorting the nodes in the descending order of their centrality values. The algorithmic complexity of adding random edges is $\mathcal{O}(n^2)$, where n represents the number of nodes of the network (the number of added edges is proportional to n^2, and the complexity of generating random numbers is $\mathcal{O}(1)$.) Determining the degree centrality for all network nodes has a complexity of $\mathcal{O}(n^2)$; calculating the betweenness of a node with the Brandes algorithm has a complexity of $\mathcal{O}(nN + n^2 \log n)$ (where N is the number of edges $|E|$ in $G = (V, E)$) [64]; therefore, as many drug networks are dense and $N \sim n^2$, computing the betweenness of all nodes in G entails between $\mathcal{O}(n^4)$ and $\mathcal{O}(n^5)$ complexity. The complexity of sorting the nodes according to their centralities as well as calculating the Kendall τ are between $\mathcal{O}(n \log n)$ and $\mathcal{O}(n^2)$ [65]. In conclusion, performing the algorithmic evaluation of network robustness from Section Network analysis robustness entails a huge computational burden, especially when processing the high-density DDI networks and considering the betweenness centrality.

Assuming the conclusion of the complexity considerations for Algorithm 1, we will focus our robustness study on DTI networks and perform it for the betweenness centrality in the less dense DrugBank 3.0 DTI network. As presented in the simulation results (see Figure 19, panels a and b) of node hierarchy robustness according to the degree centrality, we notice in both DrugBank 3.0 and 5.1.8 DTI networks that the Kendall τ decreases linearly with the unknown edge rate q, and only a slight increase for DrugBank 5.1.8 DTI in comparison with DrugBank 3.0 (Figure 19, panel c). After repeating all simulations 100 times for each q, we noticed a low variability in Figure 19, panels a and b.

As explained, due to algorithmic complexity reasons, we perform the betweenness centrality robustness test in Algorithm 1 for the DrugBank 3.0 DTI; the corresponding simulation results in Figure 19, panel d, reveal a logarithmic decrease with q, but a high variability of τ.

Because the analysis of the degree distribution under different rates of unknown interactions q is not computationally prohibitive in terms of complexity, according to Algorithm 1, we analyze the power-law distribution parameters in drug-target interaction networks (DTI) for DrugBank 3.0 and 5.1.8. We choose to focus our analysis on DTI—instead of DDI—because the DDI networks are very dense already, and adding unknown interactions does not have a significant impact. Figure 20 comparatively presents the evolution of the power-law degree distribution exponent α with the rate of unknown interactions q in DrugBank 3.0 (panel a) and DrugBank 5.1.8 (panel b) DTI networks.

Discussion

Our discussions mainly stress the drug datasets' vulnerable aspects, as revealed by the analysis results, specifically those related to the DDI and DTI networks.

Discussions on DDI networks

The first finding of our analysis is that the DDI networks built with data from the latest DrugBank versions deviate significantly from the typical complex network parameters and centrality distributions. Such a situation is generated by the high network density, owing to the colossal number of reported interactions. (The latest DDI network has a massive average degree of 650 and a very small average path length of around 2). The problematic situation of the very dense DDI networks calls for addressing the following issues.

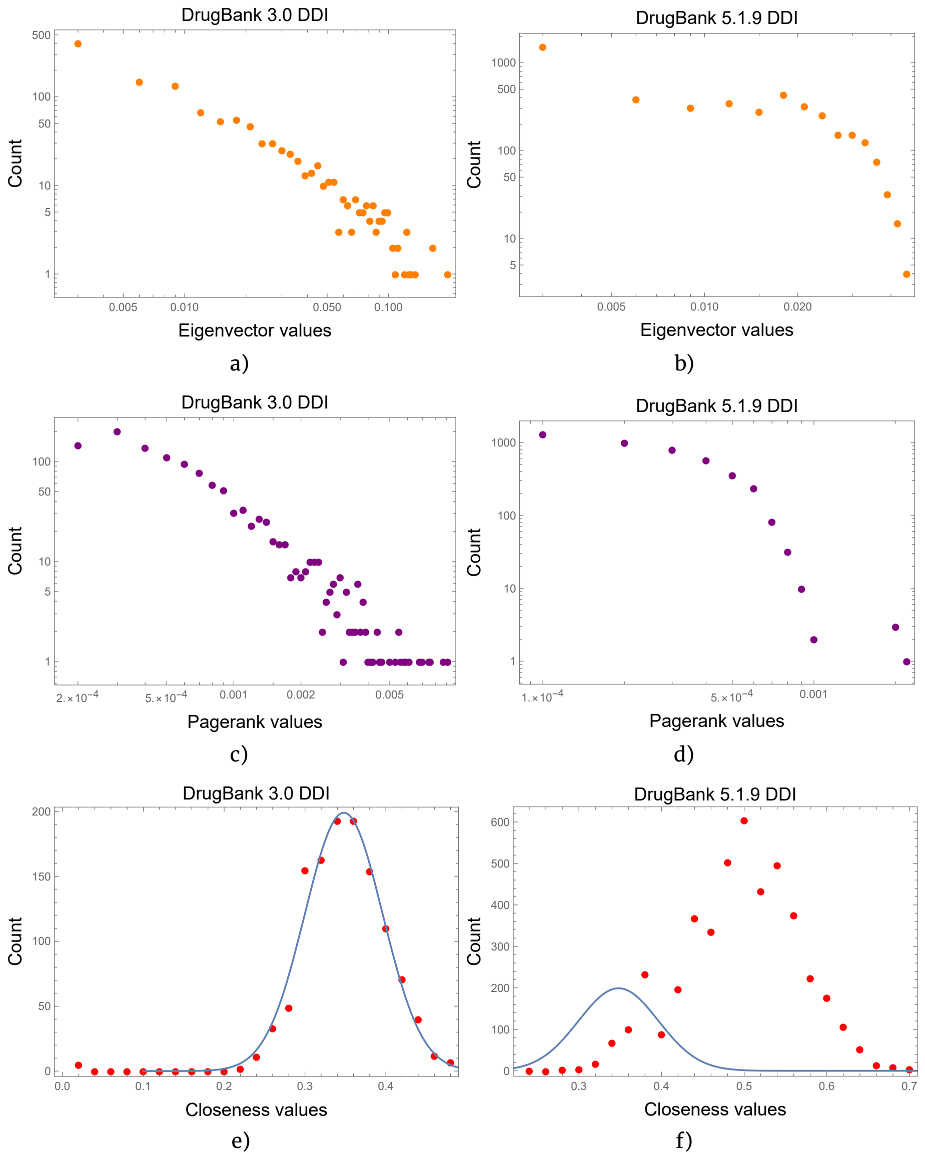

Figure 10. The comparison between eigenvector, PageRank, and closeness distributions in DDI networks built with information from DrugBank 3.0 and 5.1.9. In panels a and b, we show that the power-law distribution of eigenvector centrality from the DrugBank 3.0 DDI degrades in the latest versions; the same can be observed for the PageRank distributions in panels c and d. We also notice that the normal distribution of closeness (a common feature of real-world complex networks, particularly drug interaction networks [35]) in the DrugBank 3.0 DDI network degrades for the DrugBank 5.1.9 DDI network, as shown with the distribution fitting blue line in panels e and f. (We performed the distribution fitting in Mathematica 13.)

Some reliable methods for filtering out the irrelevant DDIs
H. Tilson et al. highlighted that a continuous process is required to select DDIs for the relevant clinical alert and support the clinical decision; physicians and pharmacists need to filter the DDIs and find a way to develop and agree on a standard set of evidence-based DDIs [66]. Indeed, medicines have a proven propensity toward drug-drug interactions in biological environments; therefore, there is a significant probability of interaction between any two drugs. However, only some of these interactions are relevant in specific circumstances.

Specifying rules/norms for recording the DDI severity
Experts promote the development of guidelines for drug-disease and drug-drug interactions in patients with multimorbidity. They suggest populational (i.e., big data) evidence to detect circumstances for relevant interactions [67]. Indeed, the different drug datasets often disagree on the severity level for the same DDI [68].

Establishing the clinical context and interactions' relevance for each drug.
The DDI severity depends on various clinical factors (e.g., a DDI may be major for some specific comorbidity or patient age and moderate/minor otherwise). Circumstances are paramount to analyzing the complexity of clinical practice and identifying potential DDIs and their attributes (i.e., type, severity, and frequency).

Clinicians' perspectives on the potential DDI clinical relevance are essential to avoid alert fatigue—a severe problem affecting the electronic DDI alert systems that support modern healthcare procedures [69]. Consequently, Pirnejad et al. recommend flexibility integrated into DDI clinical decision support systems to personalize potential DDI alerts [70].

Another approach proposes criteria for evaluating/scoring high-priority DDIs that provide clinical decision support alerts in electronic health records (EHR) [71]. E. Kontsioti and collaborators started from the assumption that the literature could be better in creating DDI reference sets or open resources that simultane-

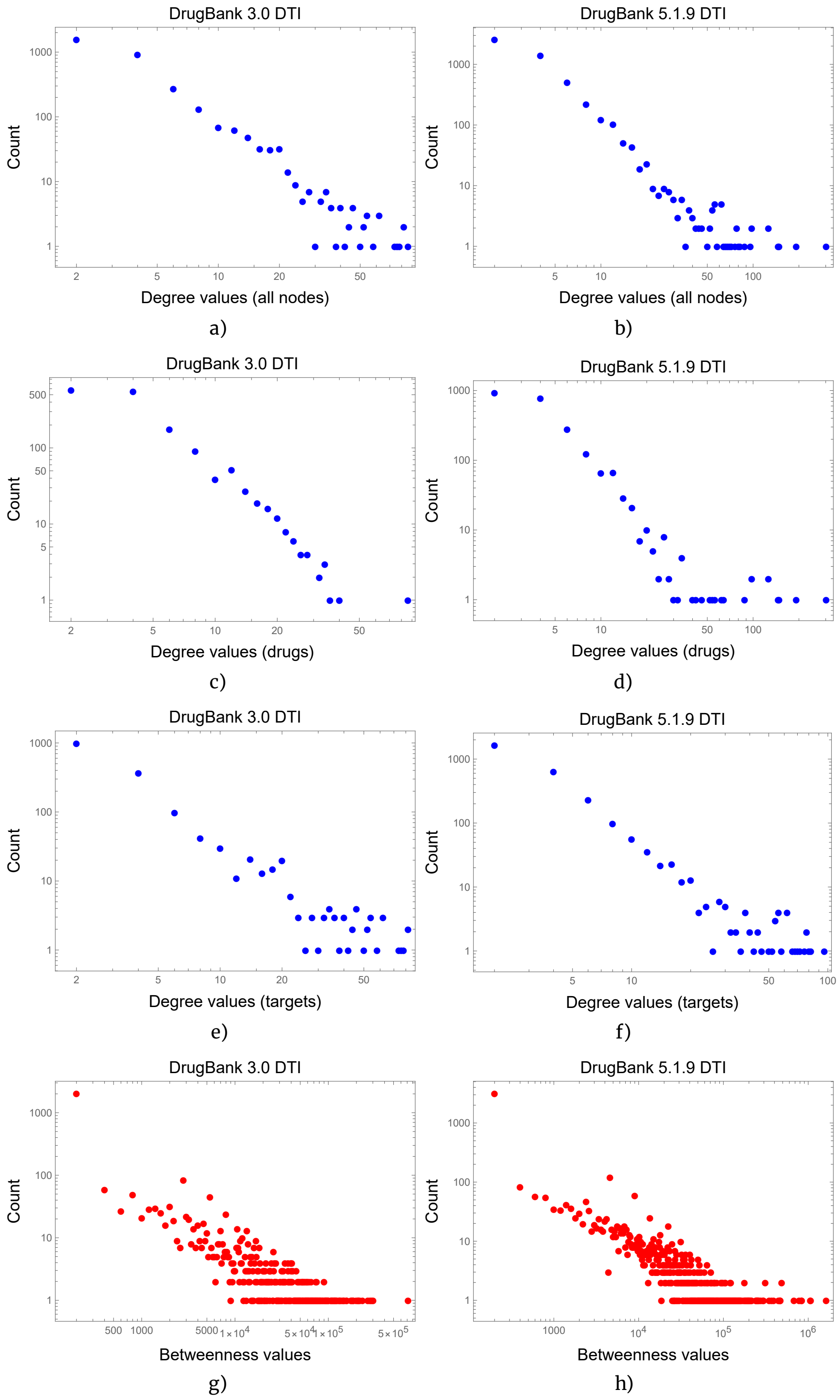

Figure 11. The degree and betweenness distributions in the drug-target interaction (DTI) networks, built with information from the first and latest DrugBank versions. (We analyzed and calculated these distributions with the Powerlaw Python package [62]). In panels a and c, e, and g, we present the degree and betweenness distributions in the DrugBank 3.0 DTI network (we separately display the drug and target, as well as all nodes' degree distributions), while panels b, d, f, and h show the DrugBank 5.1.9 counterparts.)

ously aim at DDIs' clinical relevance and interacting drugs' behavior. They automatically extracted and ensembled data from multiple resources and provided a pipeline for generating a reference set for DDIs that help postmarketing drug surveillance [72].

Nonetheless, the problem is that the drug datasets do not record the clinical context associated with the DDI severity; this may explain why different drug databases report different severity levels for the same DDI—they may assume dissimilar clinical situations.

Building a negative dataset of DDIs with drug pairs known as non-interacting in specific clinical circumstances

To simplify clinical work, A. Assiri and colleagues developed an anti-DDI resource for 200 drugs—a set of drug combinations with

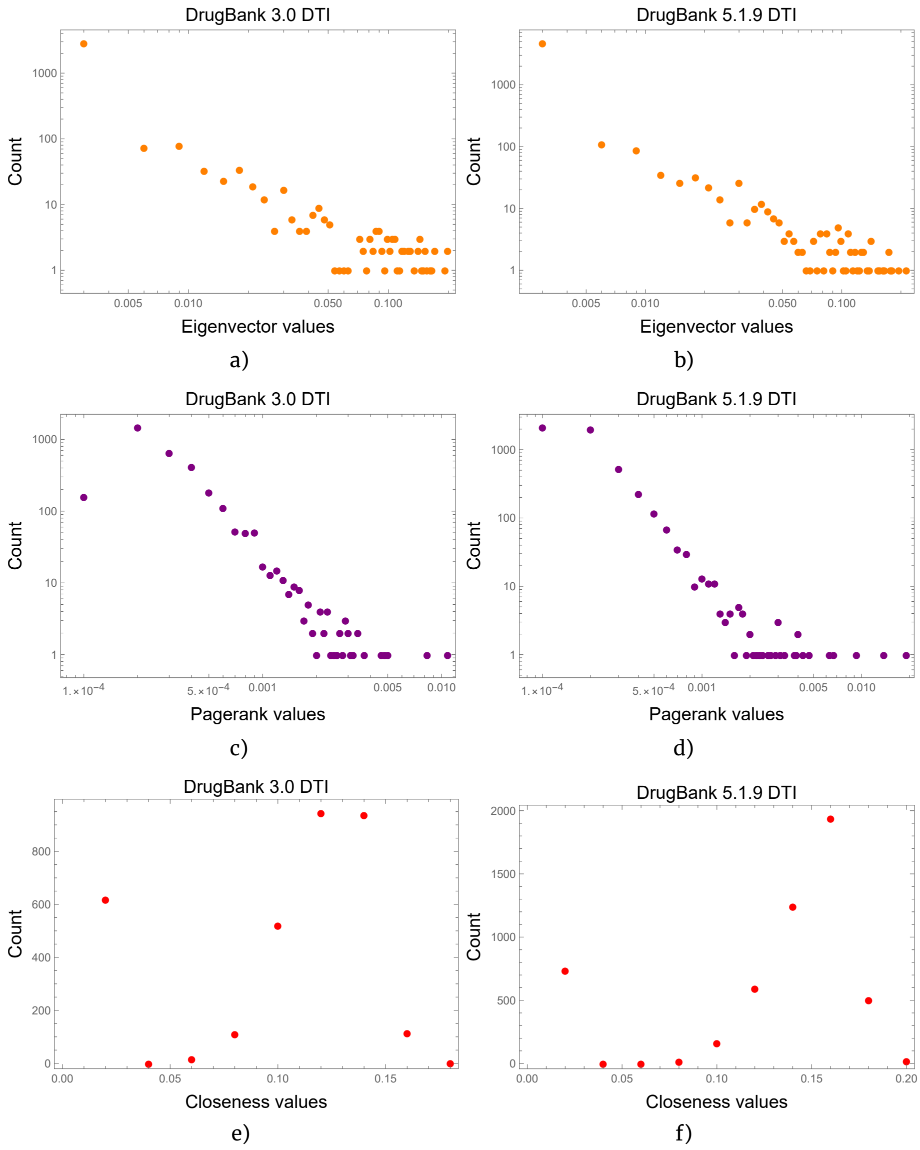

Figure 12. The comparison between eigenvector, PageRank, and closeness distributions in DTI networks with information from DrugBank 3.0 and 5.1.9. In panels a and b, we show that the eigenvector distribution from the DrugBank 3.0 DTI does not degrade in the latest versions; the same can be observed for the PageRank distributions in panels c and d. We also notice the non-regular distribution of closeness in both DrugBank 3.0 DTI and DrugBank 5.1.9 DTI networks in panels e and f. (We performed the distribution fitting in Mathematica 13.)

negative reported interactions (i.e., no risk of DDI) [73].

However, negative DDI information is scarce, and looking for such data means navigating a vast search space; therefore, developing computer-based tools for predicting non-interacting drug pairs would be highly beneficial [74].

Discussions on DTI networks

Our analysis results for DTI networks suggest that the data available across all drug database versions miss much information on drug-target interactions. Also, our simulations indicate that the robustness of centrality-based network analysis methods improves (slightly) with the newer database versions (see Figure 19); however, these latest versions' degree distribution is much more stable (Figure 20). The DTIs do not have the high-density problem of DDI networks and the entailed consequences. The DTI network results indicate the need to address the following issues.

Accurate methods for DTI predictions

We need more accurate drug-target interaction prediction tools, particularly for the DTIs involving new targets (e.g., introduced each new year by the FDA [42]). Such computational methods will help biologists prune the enormous drug-target interaction search space and focus on the most promising and potentially impactful experiments.

Computational DTI prediction methods evolved as a convenient alternative (or complement) to the conventional methods for discovering new drugs, repositioning drugs, or uncovering potential drug side effects. The DTI prediction methods are diverse: multi-molecular networks based on deep walk embedding model [75], knowledge graph embedding (KGE) model-neural factorization machine (NFM) unified framework [76], convolutional neural networks using only data on drug structure and protein sequence [77], heterogeneous network-based methods that integrate various drug data [8], combined computational techniques such as graph embeddings, graph mining, and machine learning [10], or a convolutional neural network method extracting local residue patterns of proteins participating in DTIs [78]. All these computational methods rely on existing (i.e., positive) drug-target interactions data; in many cases, they also use additional relevant data such as drug-drug or

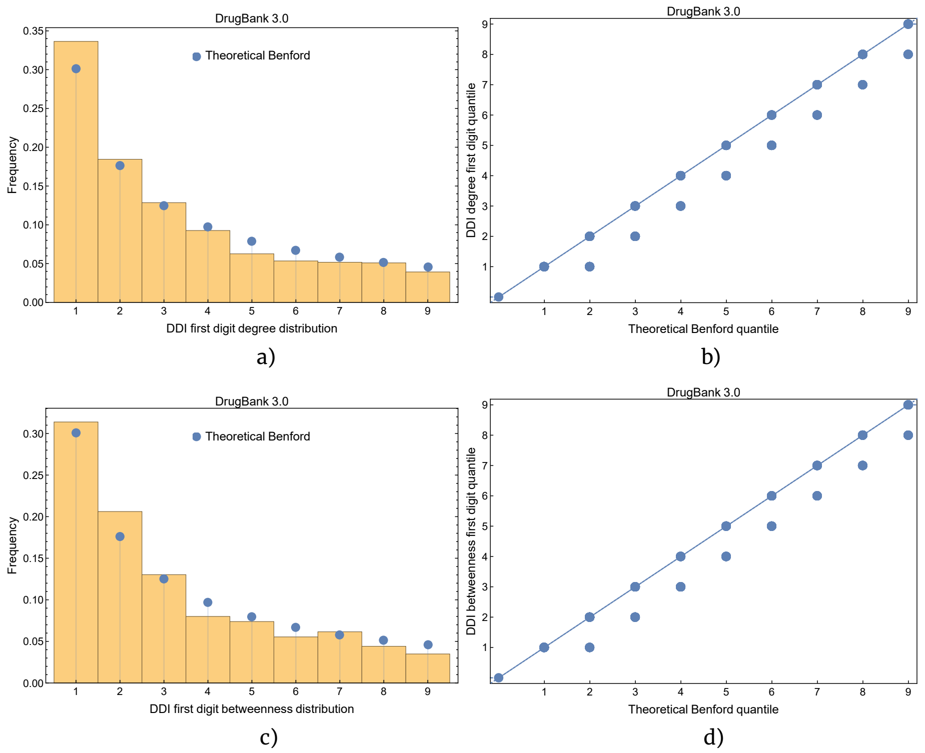

Figure 13. The compliance of degree and betweenness distributions in DDI networks built with DrugBank 3.0 data: a) the comparison between the empirical distribution represented in the histogram and the theoretical Benford distribution represented with blue disks, b) the Q-Q plot where the dashed trendline following the diagonal indicates a small distance to theoretical Benford distribution, c) the comparison between the empirical distribution and the theoretical Benford distribution, d) the Q-Q plot where the dashed also indicates a small distance to theoretical Benford distribution.

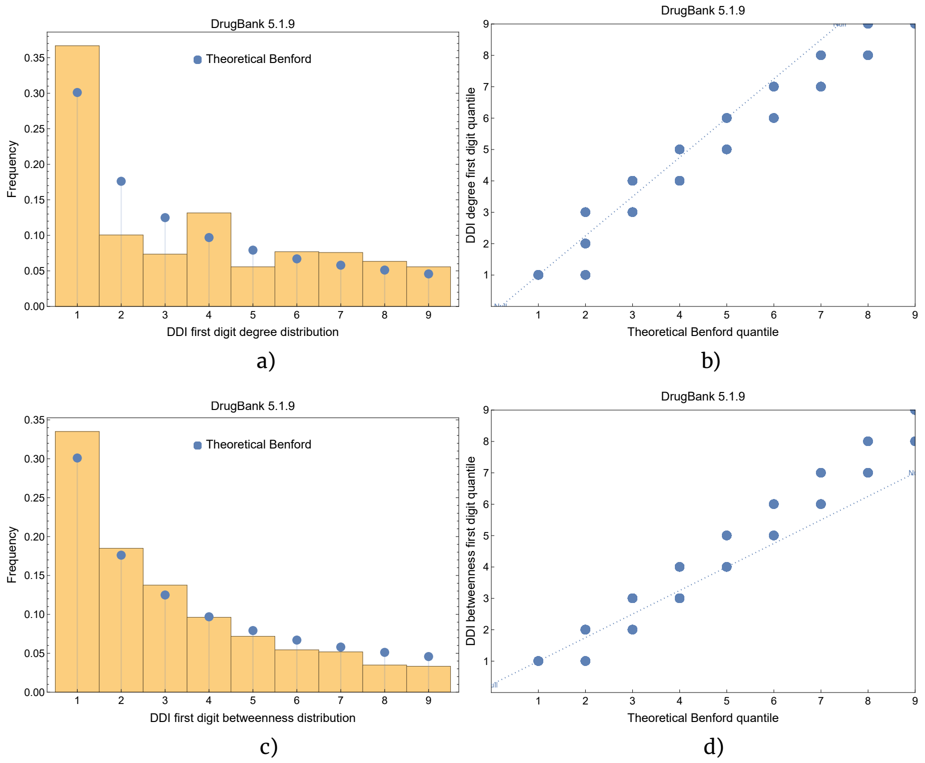

Figure 14. The compliance of degree distribution in DDI networks built with DrugBank 5.1.9 data: a) the comparison between the empirical distribution represented in the histogram and the theoretical Benford distribution represented with blue disks, b) the Q-Q plot where the dashed trendline does not follow the diagonal indicates a significant distance to theoretical Benford distribution, c) the comparison between the empirical distribution and the theoretical Benford distribution, d) the Q-Q plot indicating a close distance to theoretical Benford distribution.

target-target structural similarity [79].

Nevertheless, measuring the accuracy in drug-target interaction prediction (i.e., comparing and ranking the prediction methods correctly) is problematic in the absence of some robust ground truth. Accordingly, recent research proposes a comprehensive benchmark for assessing drug-target interaction prediction methods

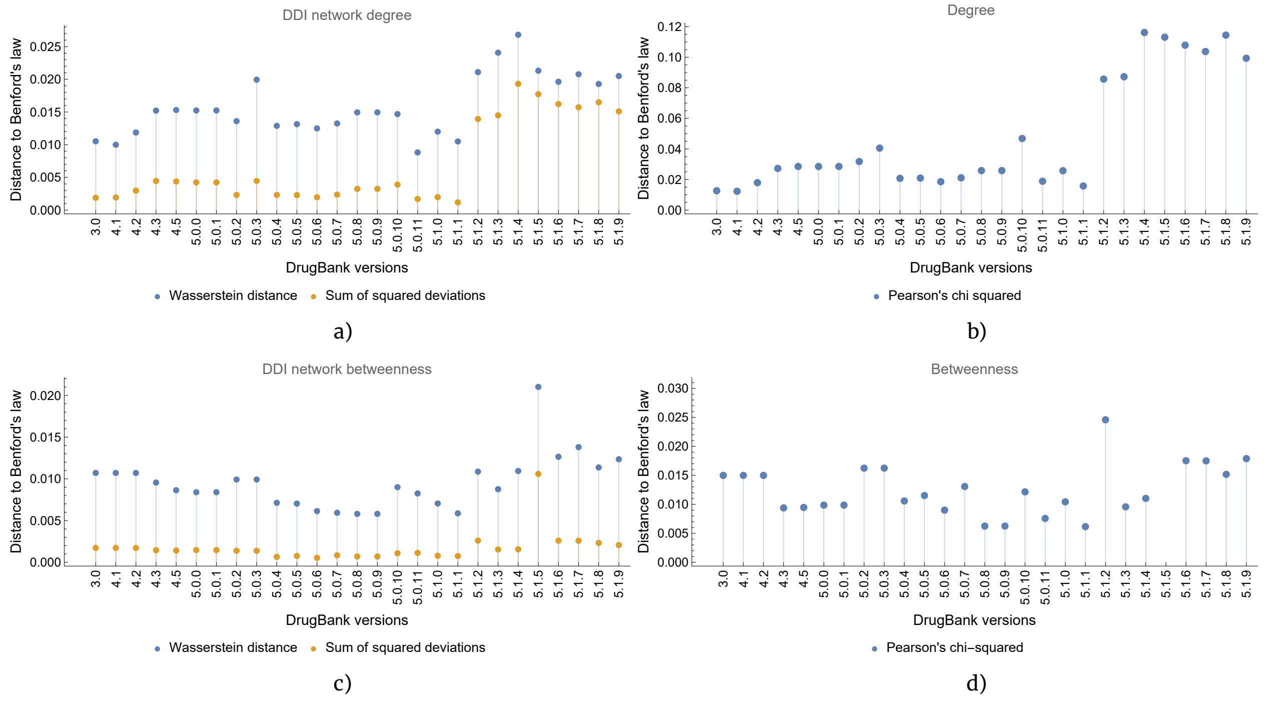

Figure 15. The evolution of distance between the Benford distribution and the empirical degree and betweenness distributions in DDI networks across DrugBank versions: a) Wasserstein distance and sum of squared deviations for the degree centrality, b) Pearson's χ^2 for the degree centrality, c) Wasserstein distance and sum of squared deviations for the betweenness centrality, b) Pearson's χ^2 for the betweenness centrality. Smaller values indicate a stronger compliance with Benford's law of the first digit.

[80], which will allow for their standardized and fair comparison.

Building negative drug-target interaction datasets

Robust DTI prediction requires a better ground truth, including the collections of interactions proven as nonexistent. However, it is impossible to test experimentally all potential DTIs because the search space is too extended.

The efficient training of DTI prediction machine learning models requires positive and negative examples—drug-target pairs experimentally demonstrated as interacting and drug-target couples proven as non-interacting. The DTIs in most drug datasets are robust positive examples because researchers demonstrated them experimentally; however, recording negative drug-target interaction examples is hard to approach [81, 82]. Most drug databases—including DrugBank—contain data supported by experimental research results reported in scientific papers. Such experiments are too expensive to aim at confirming the absence of drug-target interactions. Indeed, the economically reasonable approach is to spend valuable resources to demonstrate existing drug-target interactions because they will lead to new therapies. In this context, a viable approach is prioritizing the potential drug-target interactions by developing computational tools for predicting non-existing DTIs.

Many approaches interpret non-confirmed drug-target interactions as mere non-interactions because the negative DTI information is still scarce. Although frequent, such a practice is misleading since the lack of evidence that something exists does not necessarily mean it does not exist [26].

Potential implications

This paper investigates whether the evolution of drug databases over the last decade has brought—besides the increasing abundance of data—a more accurate and robust analysis of drug interaction networks. We found that the data abundance in the latest DrugBank versions has rendered DDI networks almost impossible to analyze because of their huge density; we also concluded that the DTI networks built with data from the latest database versions only slightly improve the analysis robustness. Fortunately, our investigation also uncovered some database issues that need adjustments (see Section Discussion). Fixing the reported problems will have far-reaching research implications.

First, in the case of drug-drug interaction data (and DDI networks), in our opinion, the field requires a research effort to define standardized labels for interaction severity. The drug-drug interaction severity labels currently used by drug databases—such as, for instance, Drugs.com and DrugBank—are character strings: "major", "moderate", "minor", and "no interactions found". While such labels convey a valuable message to pharmacologists, they are not helpful for statistical analysis or machine learning approaches. Clearly, any statistical or machine learning method must properly quantify the differences between severity labels. By this logic, it is difficult to quantify the difference between character strings "major" and "moderate" in comparison with, say, the difference between "major" and "no interactions found"; yet, from a pharmacological standpoint, the difference is substantial. Consequently, we consider that the field needs rigorous research to define numerical labels for the drug-drug interaction severity labels. Such an undertaking would not be trivial because merely respectively encoding "major", "moderate", "minor", and "no interactions were found" as "3", "2", "1", and "0" does not solve all issues. For instance, Drugs.com considers "major" interactions that are either "contraindicated" in any circumstance or tolerated under medical supervision; there is an evident difference between the two types of "major" drug-drug interactions.

Second, owing to the lack of negative information (i.e., drug-drug and drug-target interactions proven experimentally as nonexistent, see Section Discussion), there is still much uncertainty even in the latest drug database versions. Consequently, it remains hard to assess the performance of drug-drug and drug-target interaction prediction methods (as well as the effectiveness of computational drug repositioning pipelines) in the absence of standardized ground truth. In our opinion, to have a fair and reliable comparison of drug-drug and drug-target prediction methods, we need comprehensive benchmark datasets. In fairness, recent research efforts acknowledge this need for comprehensive benchmarking

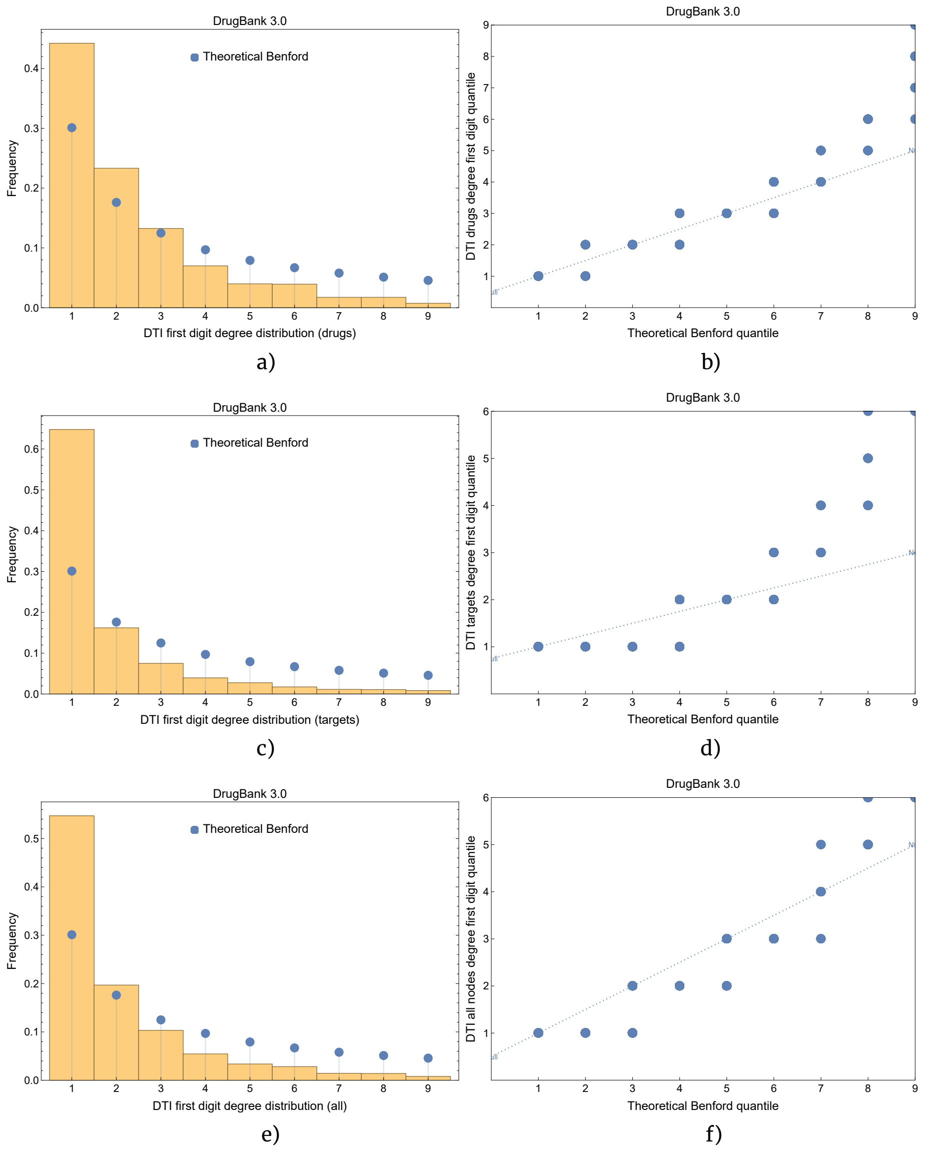

Figure 16. The compliance of degree distribution in DTI networks built with DrugBank 3.0 data. In panels a, c, and e, we compare the theoretical Benford distribution with blue disks and the degree distribution in DTI network nodes representing drugs, targets, and all DTI network nodes. In panels b, d, and f, we present the Q-Q plots corresponding to panels a, c, and e. All the results in this figure indicate non-compliance with the theoretical Benford distribution.

[80]; however, we think that a lot more research should be spent to standardize such benchmarks and benchmark suites, which the bioinformatics community should adopt. To this end, we believe we can draw inspiration from the way the computer architecture community managed to standardize the measurement of computer performance with the SPEC benchmark suites [83].

Third, any comprehensive benchmark dataset must contain negative information, namely drug-drug and drug-target interactions proven impossible (or inexistent). The effort of collecting a dataset of negative drug-target interaction examples is not necessarily daunting. To this end, we suggest that computational methods—such as molecular docking or molecular fingerprints [84, 40]—can be employed to precisely identify the most likely non-interactions. Thus, the resources entailed by the confirming experiments will be mitigated substantially.

Availability of source code and requirements

The page https://github.com/research-hyperion/Drug_database_statistics hosts the software implementing the data analysis methods described in this paper—entitled *Drug Database Statistics*. Our implementation uses Python and Wolfram Language; it is platform-independent and requires Docker Desktop on Microsoft Windows or Docker Engine on Linux distros. The software can be used under the GNU GPL v3.0 license.

Addtitional files

We provide all the analyses and simulations numerical results in file https://github.com/research-hyperion/Drug_database_statistics/blob/master/Drug-networks-results-synthesis.xlsx.

Declarations

List of abbreviations

ATC: anatomical therapeutic chemical classification system; COVID-19: coronavirus disease 2019; COX: cyclooxygenase; DDI: drug-drug interaction; DTI: drug-target interaction; EHR: electronic health record; FDA: United States food and drug administration; KGE: knowledge graph embedding; mRNA: messenger ribonucleic acid;

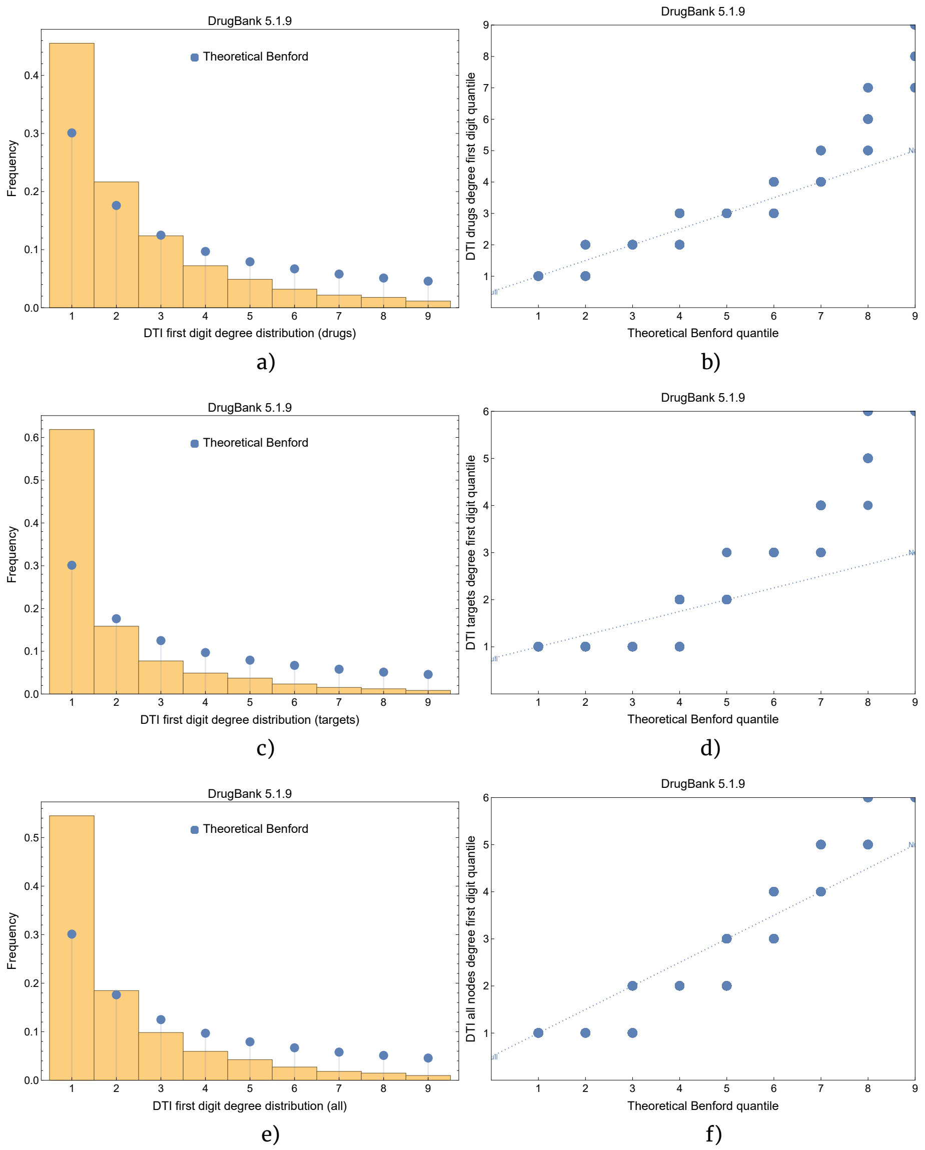

Figure 17. The compliance of degree distribution in DTI networks built with DrugBank 5.1.9 data (i.e., latest version). In panels a, c, and e, we compare the theoretical Benford distribution with blue disks and the degree distribution in DTI network nodes representing drugs, targets, and all DTI network nodes. In panels b, d, and f, we present the Q-Q plots corresponding to panels a, c, and e. All the results in this figure indicate non-compliance with the theoretical Benford distribution.

NFM: neural factorization machine; SPEC: standard performance evaluation corporation.

Ethical approval

Not applicable.

Consent for publication

Not applicable.

Competing interests

The authors declare that they have no competing interests.

Funding

This work was supported by a grant of the Romanian Ministry of Education and Research, CCCDI - UEFISCDI, project number PN-III-P2-2.1-PED-2019-2842, within PNCDI III.

Author's contributions

Design of study: M.U., L.U.; funding acquisition: M.U., L.U.; data analysis: M.U., S.M.A.; draft preparation: M.U., L.U.; review and editing: M.U., S.M.A., L.U.

Acknowledgements

Not applicable.

References

1. Recanatini M, Cabrelle C. Drug research meets network science: Where are we? Journal of medicinal chemistry 2020;63(16):8653–8666.
2. Azuaje F. Drug interaction networks: an introduction to translational and clinical applications. Cardiovascular research 2013;97(4):631–641.

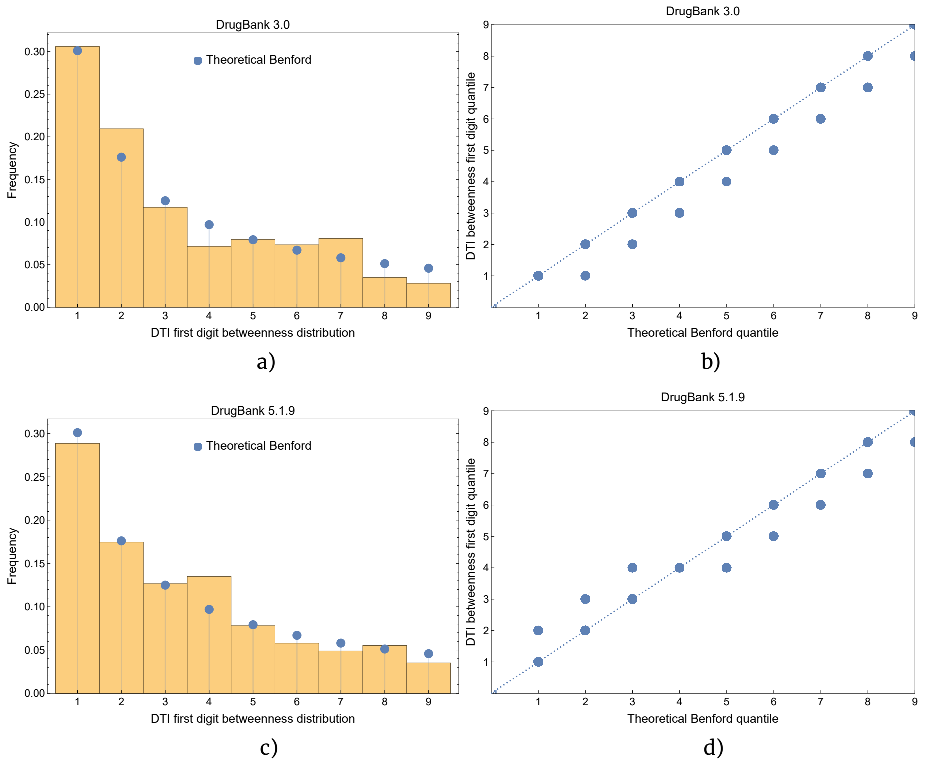

Figure 18. The compliance of betweenness distribution in DTI networks with Benford's law. In panels a and c, we compare the theoretical Benford distribution (blue disks) with the empirical betweenness distribution in DTI network nodes built with DrugBank 3.0 and 5.1.9 data, respectively. In panels b and d, we present the Q-Q plots corresponding to panels a and c. All the results in this figure indicate a relatively good compliance with the theoretical Benford distribution.

3. Zhang W, Chen Y, Liu F, Luo F, Tian G, Li X. Predicting potential drug-drug interactions by integrating chemical, biological, phenotypic and network data. BMC bioinformatics 2017;18(1):1–12.
4. Zhu J, Liu Y, Zhang Y, Chen Z, Wu X. Multi-Attribute Discriminative Representation Learning for Prediction of Adverse Drug-Drug Interaction. IEEE Transactions on Pattern Analysis and Machine Intelligence 2021;.
5. Qiu Y, Zhang Y, Deng Y, Liu S, Zhang W. A comprehensive review of computational methods for drug-drug interaction detection. IEEE/ACM Transactions on Computational Biology and Bioinformatics 2021;.
6. Su X, You ZH, Huang Ds, Wang L, Wong L, Ji B, et al. Biomedical Knowledge Graph Embedding with Capsule Network for Multi-label Drug-Drug Interaction Prediction. IEEE Transactions on Knowledge and Data Engineering 2022;.
7. Lu Y, Guo Y, Korhonen A. Link prediction in drug-target interactions network using similarity indices. BMC bioinformatics 2017;18(1):1–9.
8. Luo Y, Zhao X, Zhou J, Yang J, Zhang Y, Kuang W, et al. A network integration approach for drug-target interaction prediction and computational drug repositioning from heterogeneous information. Nature communications 2017;8(1):1–13.
9. Wu Z, Li W, Liu G, Tang Y. Network-based methods for prediction of drug-target interactions. Frontiers in pharmacology 2018;9:1134.
10. Thafar MA, Olayan RS, Ashoor H, Albaradei S, Bajic VB, Gao X, et al. DTiGEMS+: drug–target interaction prediction using graph embedding, graph mining, and similarity-based techniques. Journal of Cheminformatics 2020;12(1):1–17.
11. Thafar MA, Olayan RS, Albaradei S, Bajic VB, Gojobori T, Essack M, et al. DTi2Vec: Drug–target interaction prediction using network embedding and ensemble learning. Journal of cheminformatics 2021;13(1):1–18.
12. Lotfi Shahreza M, Ghadiri N, Mousavi SR, Varshosaz J, Green JR. A review of network-based approaches to drug repositioning. Briefings in bioinformatics 2018;19(5):878–892.
13. Sadeghi SS, Keyvanpour MR. An analytical review of computational drug repurposing. IEEE/ACM transactions on computational biology and bioinformatics 2019;18(2):472–488.
14. Badkas A, De Landtsheer S, Sauter T. Topological network measures for drug repositioning. Briefings in bioinformatics 2020;.
15. Jourdan JP, Bureau R, Rochais C, Dallemagne P. Drug repositioning: a brief overview. Journal of Pharmacy and Pharmacology 2020;72(9):1145–1151.
16. Bolgár B, Arany A, Temesi G, Balogh B, Antal P, Matyus P. Drug repositioning for treatment of movement disorders: from serendipity to rational discovery strategies. Current topics in medicinal chemistry 2013;13(18):2337–2363.
17. Sridhar D, Fakhraei S, Getoor L. A probabilistic approach for collective similarity-based drug–drug interaction prediction. Bioinformatics 2016;32(20):3175–3182.
18. Lin X, Quan Z, Wang ZJ, Ma T, Zeng X. KGNN: Knowledge Graph Neural Network for Drug-Drug Interaction Prediction. In: IJCAI, vol. 380; 2020. p. 2739–2745.
19. Feng YH, Zhang SW, Shi JY. DPDDI: a deep predictor for drug-drug interactions. BMC bioinformatics 2020;21(1):1–15.
20. Dickson M, Gagnon JP. The cost of new drug discovery and development. Discovery medicine 2009;4(22):172–179.
21. Chen XQ, Antman MD, Gesenberg C, Gudmundsson OS. Discovery pharmaceutics—challenges and opportunities. The AAPS journal 2006;8(2):E402–E408.
22. Food US, Administration D, Novel Drug Approvals for 2021; 2021. [Online; accessed 21-January-2022]. https://www.fda.gov/drugs/new-drugs-fda-cders-new-molecular-entities-and-new-therapeutic-biological-products/novel-drug-approvals-2021.
23. Sardana D, Zhu C, Zhang M, Gudivada RC, Yang L, Jegga AG. Drug repositioning for orphan diseases. Briefings in bioinfor-

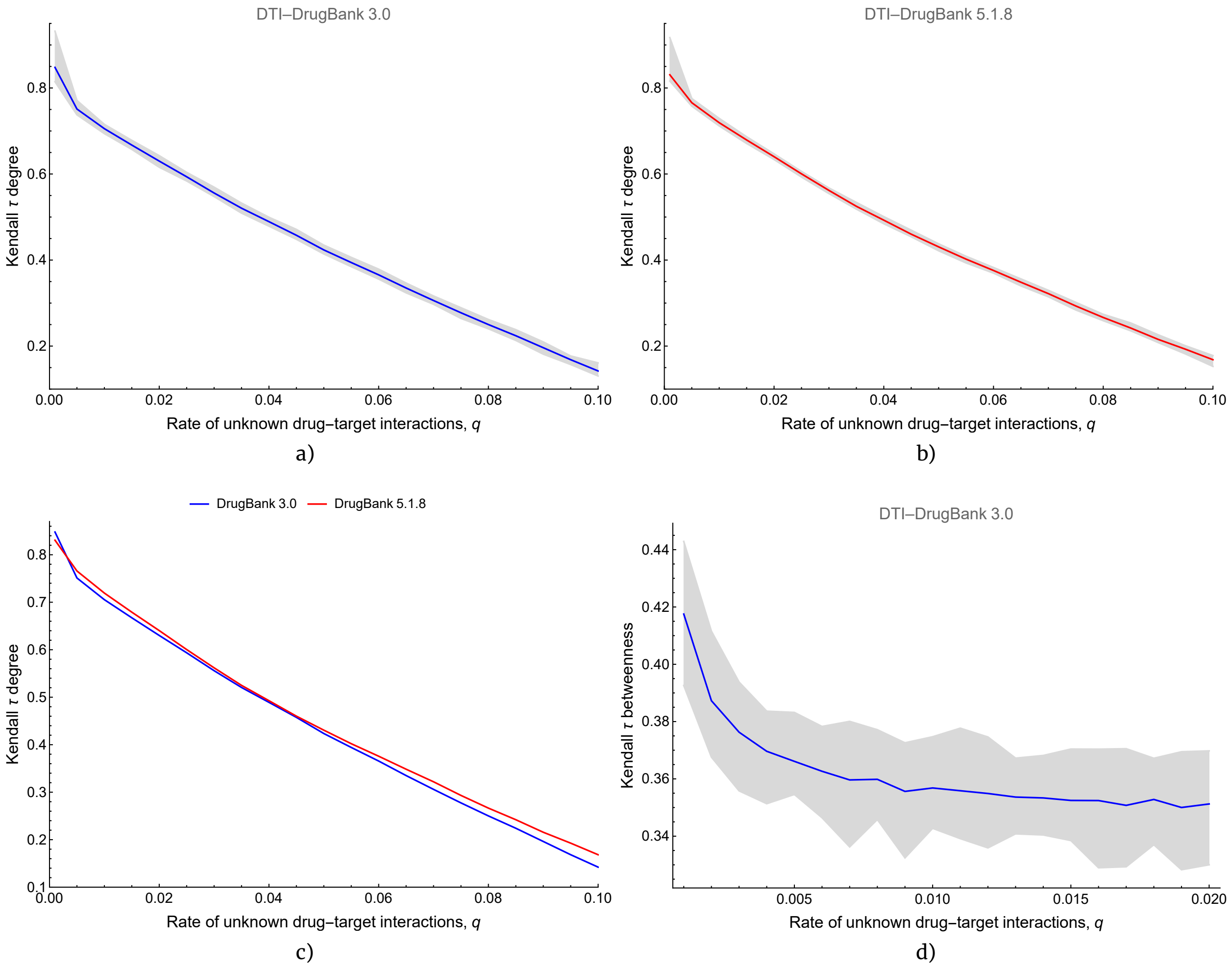

Figure 19. The evolution of Kendall τ (measuring the centrality-based ordinal correlation between the known DTI G and the DTI including unknown edges G') with the rate (i.e., fraction) of unknown edges q. Panel a shows the results for the DrugBank 3.0 DTI and the degree centrality, with the blue line following the average τ after 100 simulations and the grey area showing the variance. Panel b is the counterpart of panel a for DrugBank 5.1.8. Panel c presents the comparison between DrugBank 3.0 and 5.1.8 DTI networks. Panel d illustrates a robustness analysis similar to a, but for the betweenness centrality.

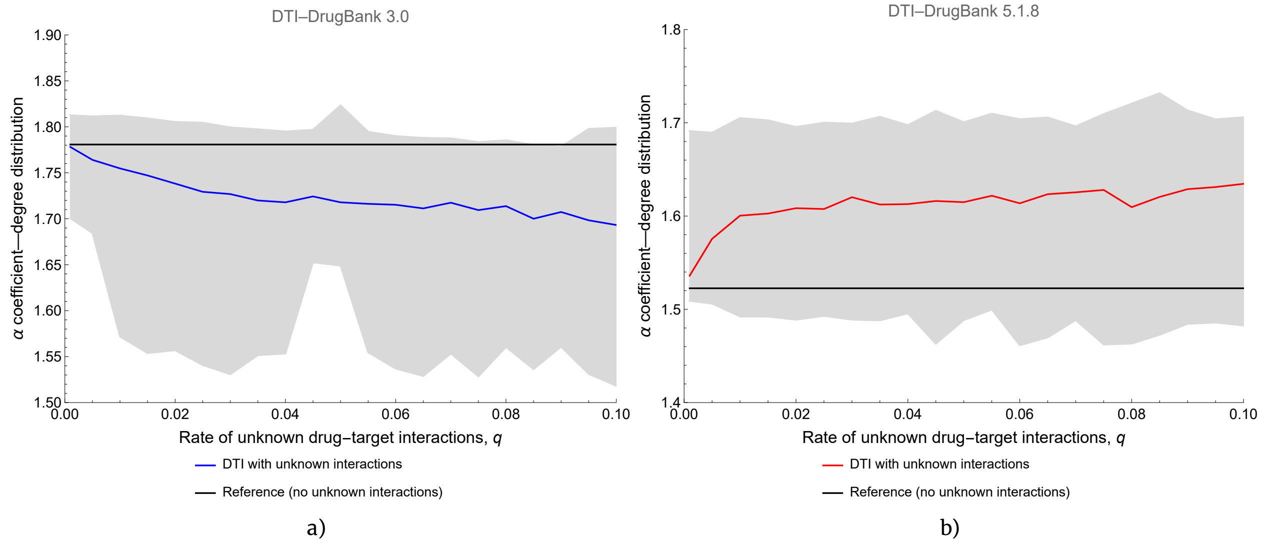

Figure 20. The evolution of the power-law distribution exponent α with the rate of unknown interactions q; panel a presents the case of the first DrugBank version 3.0 DTI network, whereas panel b shows the case of the DrugBank version 5.1.8 DTI network. In the figure, the black lines represent the reference α values (i.e., corresponding to DTI networks with no unknown interactions, $q = 0$). We performed simulations 100 times for each q value, showing the average values with blue and red lines, while the grey area represents the interval between minimum and maximum values.

matics 2011;12(4):346–356.

24. Serafin MB, Bottega A, Foletto VS, da Rosa TF, Hörner A, Hörner R. Drug repositioning is an alternative for the treatment of coronavirus COVID-19. International journal of antimicrobial

agents 2020;55(6):105969.
25. Gysi DM, Do Valle Í, Zitnik M, Ameli A, Gan X, Varol O, et al. Network medicine framework for identifying drug-repurposing opportunities for COVID-19. Proceedings of the National Academy of Sciences 2021;118(19).
26. Altman DG, Bland JM. Statistics notes: Absence of evidence is not evidence of absence. Bmj 1995;311(7003):485.
27. Mestres J, Gregori-Puigjane E, Valverde S, Sole RV. Data completeness—the Achilles heel of drug-target networks. Nature biotechnology 2008;26(9):983–984.
28. Wishart DS, Feunang YD, Guo AC, Lo EJ, Marcu A, Grant JR, et al. DrugBank 5.0: a major update to the DrugBank database for 2018. Nucleic acids research 2018;46(D1):D1074–D1082.
29. Avizienis A, Laprie JC, Randell B, Landwehr C. Basic concepts and taxonomy of dependable and secure computing. IEEE transactions on dependable and secure computing 2004;1(1):11–33.
30. Bleakley K, Yamanishi Y. Supervised prediction of drug–target interactions using bipartite local models. Bioinformatics 2009;25(18):2397–2403.
31. Cheng F, Liu C, Jiang J, Lu W, Li W, Liu G, et al. Prediction of drug-target interactions and drug repositioning via network-based inference. PLoS computational biology 2012;8(5):e1002503.
32. Lü L, Pan L, Zhou T, Zhang YC, Stanley HE. Toward link predictability of complex networks. Proceedings of the National Academy of Sciences 2015;112(8):2325–2330.
33. Xue H, Li J, Xie H, Wang Y. Review of drug repositioning approaches and resources. International journal of biological sciences 2018;14(10):1232.
34. Wu Z, Wang Y, Chen L. Network-based drug repositioning. Molecular BioSystems 2013;9(6):1268–1281.
35. Udrescu L, Sbârcea L, Topîrceanu A, Iovanovici A, Kurunczi L, Bogdan P, et al. Clustering drug-drug interaction networks with energy model layouts: community analysis and drug repurposing. Scientific reports 2016;6(1):1–10.
36. Yamanishi Y, Araki M, Gutteridge A, Honda W, Kanehisa M. Prediction of drug–target interaction networks from the integration of chemical and genomic spaces. Bioinformatics 2008;24(13):i232–i240.
37. Mestres J, Gregori-Puigjané E, Valverde S, Solé RV. The topology of drug–target interaction networks: implicit dependence on drug properties and target families. Molecular BioSystems 2009;5(9):1051–1057.
38. Tabei Y, Pauwels E, Stoven V, Takemoto K, Yamanishi Y. Identification of chemogenomic features from drug–target interaction networks using interpretable classifiers. Bioinformatics 2012;28(18):i487–i494.
39. Tanoli Z, Alam Z, Ianevski A, Wennerberg K, Vähä-Koskela M, Aittokallio T. Interactive visual analysis of drug–target interaction networks using drug target profiler, with applications to precision medicine and drug repurposing. Briefings in bioinformatics 2020;21(1):211–220.
40. Udrescu L, Bogdan P, Chiş A, Sîrbu IO, Topîrceanu A, Văruţ RM, et al. Uncovering New Drug Properties in Target-Based Drug–Drug Similarity Networks. Pharmaceutics 2020;12(9):879.
41. Newman ME, Barabási ALE, Watts DJ. The structure and dynamics of networks. Princeton university press; 2006.
42. Avram S, Halip L, Curpan R, Oprea TI. Novel drug targets in 2021. Nature reviews Drug Discovery 2022;.
43. Wishart DS, Knox C, Guo AC, Cheng D, Shrivastava S, Tzur D, et al. DrugBank: a knowledgebase for drugs, drug actions and drug targets. Nucleic acids research 2008;36(suppl_1):D901–D906.
44. Wishart DS, Knox C, Guo AC, Shrivastava S, Hassanali M, Stothard P, et al. DrugBank: a comprehensive resource for in silico drug discovery and exploration. Nucleic acids research 2006;34(suppl_1):D668–D672.
45. Newman ME. The structure and function of networks. Computer Physics Communications 2002;147(1-2):40–45.
46. Wang XF, Chen G. Complex networks: small-world, scale-free and beyond. IEEE circuits and systems magazine 2003;3(1):6–20.
47. Barabási AL. Network science. Philosophical Transactions of the Royal Society A: Mathematical, Physical and Engineering Sciences 2013;371(1987):20120375.
48. Jeong H, Mason SP, Barabási AL, Oltvai ZN. Lethality and centrality in protein networks. Nature 2001;411(6833):41–42.
49. Koschützki D, Schreiber F. Comparison of centralities for biological networks. In: German Conference on Bioinformatics 2004, GCB 2004 Society for Computer Science eV; 2004. .
50. Salavati C, Abdollahpouri A, Manbari Z. Ranking nodes in complex networks based on local structure and improving closeness centrality. Neurocomputing 2019;336:36–45.
51. Yıldırım MA, Goh KI, Cusick ME, Barabási AL, Vidal M. Drug—target network. Nature biotechnology 2007;25(10):1119–1126.
52. MacCuish JD, MacCuish NE. Clustering in bioinformatics and drug discovery. CRC Press; 2010.
53. Estrada E. The structure of complex networks: theory and applications. Oxford University Press; 2012.
54. Topirceanu A, Udrescu M, Vladutiu M. Genetically optimized realistic social network topology inspired by facebook. In: Online Social Media Analysis and Visualization Springer; 2014.p. 163–179.
55. Orita M, Hagiwara Y, Moritomo A, Tsunoyama K, Watanabe T, Ohno K. Agreement of drug discovery data with Benford's law. Expert opinion on drug discovery 2013;8(1):1–5.
56. Morzy M, Kajdanowicz T, Szymański BK. Benford's distribution in complex networks. Scientific reports 2016;6(1):1–8.
57. Grandison S, Morris RJ. Biological pathway kinetic rate constants are scale-invariant. Bioinformatics 2008;24(6):741–743.
58. Karthik D, Stelzer G, Gershanov S, Baranes D, Salmon-Divon M. Elucidating tissue specific genes using the Benford distribution. BMC genomics 2016;17(1):1–15.
59. Kastrin A, Ferk P, Leskošek B. Predicting potential drug-drug interactions on topological and semantic similarity features using statistical learning. PloS one 2018;13(5):e0196865.
60. Goh KI, Cusick ME, Valle D, Childs B, Vidal M, Barabási AL. The human disease network. Proceedings of the National Academy of Sciences 2007;104(21):8685–8690.
61. Barabási AL. Scale-free networks: a decade and beyond. science 2009;325(5939):412–413.
62. Alstott J, Bullmore E, Plenz D. powerlaw: a Python package for analysis of heavy-tailed distributions. PloS one 2014;9(1):e85777.
63. Kossovsky AE. On the Mistaken Use of the Chi-Square Test in Benford's Law. Stats 2021;4(2):419–453.
64. Bhardwaj S, Niyogi R, Milani A. Performance analysis of an algorithm for computation of betweenness centrality. In: International Conference on Computational Science and Its Applications Springer; 2011. p. 537–546.
65. Christensen D. Fast algorithms for the calculation of Kendall's τ. Computational Statistics 2005;20(1):51–62.
66. Tilson H, Hines LE, McEvoy G, Weinstein DM, Hansten PD, Matuszewski K, et al. Recommendations for selecting drug–drug interactions for clinical decision support. American Journal of health-system pharmacy 2016;73(8):576–585.
67. Dumbreck S, Flynn A, Nairn M, Wilson M, Treweek S, Mercer SW, et al. Drug-disease and drug-drug interactions: systematic examination of recommendations in 12 UK national clinical guidelines. bmj 2015;350.
68. Monteith S, Glenn T, Gitlin M, Bauer M. Potential drug interactions with drugs used for bipolar disorder: A comparison of 6 drug interaction database programs. Pharmacopsychiatry 2020;53(05):220–227.
69. Phansalkar S, Van der Sijs H, Tucker AD, Desai AA, Bell DS,

Teich JM, et al. Drug—drug interactions that should be non-interruptive in order to reduce alert fatigue in electronic health records. Journal of the American Medical Informatics Association 2013;20(3):489–493.
70. Pirnejad H, Amiri P, Niazkhani Z, Shiva A, Makhdoomi K, Abkhiz S, et al. Preventing potential drug-drug interactions through alerting decision support systems: a clinical context based methodology. International journal of medical informatics 2019;127:18–26.
71. Phansalkar S, Desai A, Choksi A, Yoshida E, Doole J, Czochanski M, et al. Criteria for assessing high-priority drug-drug interactions for clinical decision support in electronic health records. BMC medical informatics and decision making 2013;13(1):1–11.
72. Kontsioti E, Maskell S, Dutta B, Pirmohamed M. A reference set of clinically relevant adverse drug-drug interactions. Scientific Data 2022;9(1):1–9.
73. Assiri A, Noor A. Anti-DDI Resource: A Dataset for Potential Negative Reported Interaction Combinations to Improve Medical Research and Decision-Making. Journal of Healthcare Engineering 2022;2022.
74. Rohani N, Eslahchi C. Drug-drug interaction predicting by neural network using integrated similarity. Scientific reports 2019;9(1):1–11.
75. Chen ZH, You ZH, Guo ZH, Yi HC, Luo GX, Wang YB. Prediction of drug–target interactions from multi-molecular network based on deep walk embedding model. Frontiers in Bioengineering and Biotechnology 2020;8:338.
76. Ye Q, Hsieh CY, Yang Z, Kang Y, Chen J, Cao D, et al. A unified drug–target interaction prediction framework based on knowledge graph and recommendation system. Nature communications 2021;12(1):1–12.
77. Hu S, Zhang C, Chen P, Gu P, Zhang J, Wang B. Predicting drug-target interactions from drug structure and protein sequence using novel convolutional neural networks. BMC bioinformatics 2019;20(25):1–12.
78. Lee I, Keum J, Nam H. DeepConv-DTI: Prediction of drug-target interactions via deep learning with convolution on protein sequences. PLoS computational biology 2019;15(6):e1007129.
79. Hassanzadeh R, Shabani-Mashcool S. Does adding the drug-drug similarity to drug-target interaction prediction methods make a noticeable improvement in their efficiency? 2022;.
80. Zong N, Li N, Wen A, Ngo V, Yu Y, Huang M, et al. BETA: a comprehensive benchmark for computational drug–target prediction. Briefings in Bioinformatics 2022;.
81. Bagherian M, Sabeti E, Wang K, Sartor MA, Nikolovska-Coleska Z, Najarian K. Machine learning approaches and databases for prediction of drug–target interaction: a survey paper. Briefings in bioinformatics 2021;22(1):247–269.
82. Chen X, Yan CC, Zhang X, Zhang X, Dai F, Yin J, et al. Drug–target interaction prediction: databases, web servers and computational models. Briefings in bioinformatics 2016;17(4):696–712.
83. Panda R, Song S, Dean J, John LK. Wait of a decade: Did SPEC CPU 2017 broaden the performance horizon? In: 2018 IEEE International Symposium on High Performance Computer Architecture (HPCA) IEEE; 2018. p. 271–282.
84. Shi H, Liu S, Chen J, Li X, Ma Q, Yu B. Predicting drug-target interactions using Lasso with random forest based on evolutionary information and chemical structure. Genomics 2019;111(6):1839–1852.
