# Supplementary material for: The curse and blessing of abundance—the evolution of drug interaction databases and their impact on drug network analysis
Source: Gigascience. 2023 Mar 9;12:giad011. doi: 10.1093/gigascience/giad011 (PMC10023830; doi:10.1093/gigascience/giad011)
Supplement: giad011_Response_to_Reviewer_Comments_Original_Submission [file giad011_response_to_reviewer_comments_original_submission.pdf]

Author's Response To Reviewer Comments

Close

---Please also see the attached file "Response to reviewers and marked changes.pdf". the response contains mathematical formulas and special characters, which we cannot include in this box. ---

Response to Reviewers

November 18, 2022

We thank the Academic Editor for allowing us to upgrade our manuscript and to address all comments. We would also like to thank the reviewers for their constructive feedback, which we believe has improved the quality of our paper. The revised version of the manuscript highlights our changes in blue.
Since all three reviewers raise multiple points, we have taken the liberty of responding point by point. All text written in bolded italics corresponds to Reviewers' comments. Our responses are written with regular fonts, while the references we made to changes incorporated in the revised manuscript are underlined.
We hope to have answered all of the reviewers' comments satisfactorily.

EDITOR COMMENTS

In particular, both reviewers feel the problem addressed in the paper and related concepts need to be explained and defined more clearly.

Response: We carefully addressed the concerns formulated by the reviewers—particularly those concerning the concepts we used—and hope they are explained more clearly in the revised version of the manuscript.

REVIEWER COMMENTS

Reviewer #1 (Remarks to the Author):

In recent years, more and more comprehensive drug datasets have been proposed, however, there is much uncertainty in these drug datasets. Researchers cannot know if the not reported interactions are absent or yet to be discovered, which hampers the accuracy of bioinformatics applications on these datasets. The authors used complex network statistics tools and simulations of randomly-inserted previously unaccounted interactions in drug-drug and drug-target interaction networks, and found that the drug-drug interaction networks built with the latest dataset versions become very dense and, therefore, almost impossible to analyze with conventional complex network methods. On the other hand, for the latest drug database versions, drug-target networks still include much uncertainty; however, the robustness of complex network analysis methods slightly improves. The authors pinpoint future research directions to improve the quality and practicality of drug databases for bioinformatics applications: benchmarking for drug-target interaction prediction and drug-drug interaction severity standardization.

Response: We very much appreciate the thorough, insightful, and fair assessment of our work. The observations and comments will undoubtedly contribute to a more robust, readable, and wide-reaching paper.

Comments and suggestions:
The author should explain more clearly the relationship among 'uncertain issue of datasets', 'the evolution of various complex network parameters and centralities', 'the integrity of the processed data' and 'the robustness of centrality-hierarchization analysis methods'.

Response:
We thank the reviewer for showing the need for clarity in the Background section—the part dedicated to analyzing the current issues in drug dataset analysis and the summary of our contributions. We used the terms cited by the reviewer in the enumeration (with bullets) of "our DDI and DTI network analysis across the DrugBank database versions."

In the initially submitted manuscript, the paragraph that tried to explain the connections between the "uncertainty issue of datasets,'' "the evolution of various complex network parameters and centralities," "the integrity of the processed data," and "the robustness of centrality-hierarchization analysis methods" read:
"Over the years, the comprehensiveness of drug datasets (such as DrugBank [28]) has constantly grown, and the data accuracy improved by manual curation, according to the latest literature results. This constant evolution of the most comprehensive drug database may have mitigated the concerns formulated by Mestres et al. Our paper investigates how the evolution of knowledge on drug interactions, mirrored by the DrugBank database, impacts the robustness of drug interaction network analysis; future research can exploit the insight we get to advance drug repositioning and drug-drug interaction prediction methods. "

Nonetheless, the reviewer is right. In the above paragraph, we used different terms than those used in the enumeration of the contributions, causing a lack of clarity and confusion. Following the reviewer's suggestion, we revised the paragraph that was intended to explain the mentioned terms. We hope that this part is much clearer in the revised form, which reads:

"As long as we do not have comprehensive negative information, the issue of uncertainty in drug datasets remains. However, the fact that—over the years—the comprehensiveness of drug datasets (such as DrugBank [28]) has constantly grown, and experts curated the data according to the latest literature results, may suggest that the integrity of the data improved. (The integrity characterizes the "absence of improper data alterations" [29].) Indeed, the constant growth of the most comprehensive drug database (reflected in the evolution of the various complex drug network parameters and centralities) may have mitigated the concerns formulated by Mestres et al. Our paper investigates how the evolution of knowledge on drug interactions mirrored by the DrugBank database, impacts the robustness of drug interaction network analysis based on centrality hierarchization methods; future research can exploit the insight we get to advance drug repositioning and drug-drug interaction prediction methods." (Section Background, paragraph 7 on page 2 in the revised manuscript.)

We introduced a new reference that acknowledges the rigorous definition of system integrity:
[29] Avizienis, A., Laprie, J.C., Randell, B. and Landwehr, C., 2004. Basic concepts and taxonomy of dependable and secure computing. IEEE Transactions on Dependable and Secure Computing, 1(1), pp.11-33.

Comment: The author should clearly define the 'typical complex network', for example, the characteristics of a typical complex network and the differences between a typical complex network and a normal network. In addition, the first sentence of the first paragraph in the section 'Complex networks' seems to be a general definition of the 'network', not the 'complex network'.

Response: We thank the reviewer for the observation. First, we added the necessary details to the definition of complex networks, which now reads, "A complex network is a graph G consisting of a node (or vertex) set V and a link (or edge) set E, $G = (V, E)$, where any edge $e_{ij}\in E$ connects nodes $v_{i\ }{,v}_j\in V$. Additionally, compared to normal graphs, real-world complex networks have a large number of nodes $\left|V\right|$ (i.e., hundreds to millions) and intricate, nontrivial topologies (i.e., links do not form regular structures but are not completely random either)." We highlighted the addition in blue text, in section Problem formulation, first paragraph in subsection Complex networks, page 3.

Second, we defined the typical characteristics of real-world complex networks in the manuscript in a new subsection titled Typical complex network characteristics (page 6 in the revised manuscript). We also point to this new subsection when using the term "typical complex networks" for the first time: "We explain the typical characteristics of real-world complex networks in subsection Typical complex network characteristics" (see the blue text on page 3, 4th paragraph). In the revised form of our manuscript, this

new subsection reads, "Generally, in real-world complex networks, the density of links is low (i.e., \sim\frac{1}{n} because the number of links is of the order n, where n is the number of nodes, and far from the order n^2 corresponding to a completely connected network); the total number of links also determines the average degree, and the average degree is significantly lower than n; the degree follows either a power-law (exponent is between 1 and 3) or normal distribution; the average path length is between 3 and 10; the clustering coefficient is larger than \frac{1}{n} but considerably lower than 1 [46, 54]." We highlighted these clarifications in blue text, section Methods, subsection Typical complex network characteristics, on page 6.

[46] Wang, X.F. and Chen, G., 2003. Complex networks: small-world, scale-free and beyond. IEEE circuits and systems magazine, 3(1), pp.6-20.
[54] Topirceanu, A., Udrescu, M. and Vladutiu, M., 2014. Genetically optimized realistic social network topology inspired by facebook. In Online Social Media Analysis and Visualization (pp. 163-179). Springer, Cham.

Comment: In the last sentence of the third paragraph in the section 'Complex networks', 'wij ≠ wji' should be 'wij=wji'.

Response: The observation is correct, and we thank the reviewer for pointing out this typo; we corrected it in the revision (see the first paragraph on page 4).

Comment: In Equation (10), the function η() doesn't seem to be defined.

Response: The reviewer is right; we did not explain the eigenvector with clarity (while maintaining conciseness). We denoted the eccentricity of a node v_i as \eta(v_i). Hence, equation (10) shows that eigenvector centrality for v_i is defined recursively: it adds the centralities of the nodes directly connected to v_i. To find the eigenvector centrality values for all nodes in the node set V of the network G = (V, E), we solve the equation Wη = λη; W is the adjacency matrix, λ is a constant, and η is the vector of eigenvector centrality values for all nodes in G. We added these clarifying details for equation 10 in the revised manuscript (please see the blue text on page 6, subsubsection Network centralities).

Comment: In the section 'Discussion', the authors should include more analysis of the results. In addition, I suggest that the authors should elaborate analysis and conclusions with relevant findings from the experimental results, instead of talking about them separately.

Response: The reviewer is correct: our Discussion section needs to be organized better. According to the suggestions, we performed the following steps:
We renamed the subsections of the Discussion as Discussions on DDI networks and Discussions on DTI networks.
We extended the discussions to new issues, such as:
The benefits of building prediction tools for identifying negative information (i.e., drug-drug and drug-target interactions that do not exist);
The need for recording the relevant clinical context for drug-drug interaction severity;
The fact that different drug datasets often disagree on the drug-drug interaction severity.
We reorganized the subsections such that we directly corroborate our analysis conclusions with the experimental results; these two parts are no longer separated.
We rewrite the entire Discussion section to eliminate redundant wording, thus bringing more conciseness and clarity to the manuscript.
Please find the revised form of the Discussion section in blue, starting on page 10.

Comment: Many important literature about the drug interactions have not been reviewed in the paper, such as DOI:10.1109/TPAMI.2021.3135841, DOI:10.1109/TKDE.2022.3154792, doi: 10.1186/s12859-016-1415-9, DOI:10.1109/TCBB.2021.3081268, etc.

Response: We thank the reviewer for pointing out that we did not include proper citations for papers on drug-drug interaction predictions reached from drug-drug and drug-target interactions. Indeed, drug-drug

interaction prediction is highly relevant to the central theme of our paper. We added a paragraph in the Background section that explains the application of drug-drug and drug-target interaction prediction (besides drug repositioning, which we already presented in detail), where we correctly cited the outstanding work on drug-drug interaction prediction suggested by the reviewer. (Please, see the blue-text in section Background, first two paragraphs on page 2.) The new paragraphs read, "As such, harnessing big amounts of data describing the intricate drug interactions (or relationships) with other entities can lead to uncovering new drug properties: previously unknown drug-drug [3, 4, 5, 6] or drug-target interactions [7, 8, 9, 10, 11], drug repositioning [12, 13, 14]. // Computational drug-drug interaction (DDI) prediction is critical in designing effective and safe drug combinations and developing drugs [5, 6]. Drug-target interaction (DTI) prediction is a key-step strategy in drug discovery that leads to uncovering new drugs for biological targets and new targets for known, approved drugs—representing a crucial step towards drug repositioning and identifying possible side effects [8, 11]."

[3] Zhang, W., Chen, Y., Liu, F., Luo, F., Tian, G. and Li, X., 2017. Predicting potential drug-drug interactions by integrating chemical, biological, phenotypic and network data. BMC bioinformatics, 18(1), pp.1-12.
[4] Zhu, J., Liu, Y., Zhang, Y., Chen, Z. and Wu, X., 2021. Multi-Attribute Discriminative Representation Learning for Prediction of Adverse Drug-Drug Interaction. IEEE Transactions on Pattern Analysis and Machine Intelligence.
[5] Qiu, Y., Zhang, Y., Deng, Y., Liu, S. and Zhang, W., 2021. A comprehensive review of computational methods for drug-drug interaction detection. IEEE/ACM Transactions on Computational Biology and Bioinformatics.
[6] Su, X., You, Z.H., Huang, D.S., Wang, L., Wong, L., Ji, B. and Zhao, B., 2022. Biomedical Knowledge Graph Embedding with Capsule Network for Multi-label Drug-Drug Interaction Prediction. IEEE Transactions on Knowledge and Data Engineering.
[7] Lu, Y., Guo, Y. and Korhonen, A., 2017. Link prediction in drug-target interactions network using similarity indices. BMC bioinformatics, 18(1), pp.1-9.
[8] Luo, Y., Zhao, X., Zhou, J., Yang, J., Zhang, Y., Kuang, W., Peng, J., Chen, L. and Zeng, J., 2017. A network integration approach for drug-target interaction prediction and computational drug repositioning from heterogeneous information. Nature communications, 8(1), pp.1-13.
[9] Wu, Z., Li, W., Liu, G. and Tang, Y., 2018. Network-based methods for prediction of drug-target interactions. Frontiers in pharmacology, 9, p.1134.
[10] Thafar, M.A., Olayan, R.S., Ashoor, H., Albaradei, S., Bajic, V.B., Gao, X., Gojobori, T. and Essack, M., 2020. DTiGEMS+: drug–target interaction prediction using graph embedding, graph mining, and similarity-based techniques. Journal of Cheminformatics, 12(1), pp.1-17.
[11] Thafar, M.A., Olayan, R.S., Albaradei, S., Bajic, V.B., Gojobori, T., Essack, M. and Gao, X., 2021. DTi2Vec: Drug–target interaction prediction using network embedding and ensemble learning. Journal of cheminformatics, 13(1), pp.1-18.
[12] Lotfi Shahreza, M., Ghadiri, N., Mousavi, S.R., Varshosaz, J. and Green, J.R., 2018. A review of network-based approaches to drug repositioning. Briefings in bioinformatics, 19(5), pp.878-892.
[13] Sadeghi, S.S. and Keyvanpour, M.R., 2019. An analytical review of computational drug repurposing. IEEE/ACM transactions on computational biology and bioinformatics, 18(2), pp.472-488.
[14] Badkas, A., De Landtsheer, S. and Sauter, T., 2021. Topological network measures for drug repositioning. Briefings in bioinformatics, 22(4), p.bbaa357.

Reviewer #2 (Remarks to the Author):

The authors proposed an interesting analysis study for several drug-related networks using different versions of the DrugBank database. Therefore, the work is network analysis and machine learning-based methods to analyze the future of drug-drug interactions and drug-target interactions. Hence, this study highlights the future research that can improve the drug databases quality and for bioinformatics applications.

This work tackles a genuine topic to improve bioinformatics applications using complex networks of drug-

related data. The abstract and the introduction were well written and presented good motivation, description. However, I have some concerns that have to be addressed. Therefore, I do recommend this work for publications in the GigaScience journal after addressing the following comments.

Response: We thank the reviewer for delivering a sharp, fair, and comprehensive summary of our work. The provided comments will substantially contribute to a much more robust, readable, and wide-reaching manuscript.

Comments
Comment 1. The problem formulation is not very clear. More details are needed for the negative or missing information but focused on the problem itself.

Response: We thank the reviewer for pointing out this issue. Indeed, we misplaced the explanation of the drug-drug and drug-target interaction networks using the formal framework from the Problem formulation section in the Results. We corrected this mistake by moving this part in the Problem formulation. Also, following the reviewer's suggestion, we used network formalism to explain better what missing and negative information means in the context of our problem. These added details and corrections are on page 4, section Problem formulation, subsection Drug networks (first three paragraphs). In the revised manuscript, these paragraphs read:

“To mine the complexity of drug interactions in the biological environment, researchers use database information to build drug networks such as drug-drug, drug-target, and drug-disease interaction networks, as well as drug-disease and drug--adverse effects association networks. This paper considers drug-drug interaction networks (complex drug networks where nodes represent drugs) and drug-target interaction networks (bipartite networks where one type of node represents drugs, and the other represents biological targets). Links represent drug-drug and drug-target interactions in the DDI and DTI networks. We do not consider drug-drug interaction type and strength. Consequently, the DDI networks are undirected, unweighted, and monopartite. Also, the drug-target interactions have no information regarding their strength, making DTI networks directed (from drug to target), unweighted, and bipartite.

Using the complex network formalism, in DDI networks, $\forall i,j,\ i\neq j,v_i\in V=\mathcal{D}\$ and $e_{ij}\in E=\mathcal{I}$ $(e_{ij}\neq0)$, where $\mathcal{D}$ is the set of drugs and$\ \mathcal{I}$ the set of drug-drug interactions. In DTI networks, we have $v_i\in V=V_\mathcal{D}\cup V_\mathcal{T}$ (where $V_\mathcal{D}$ is the set of nodes representing drugs and $V_\mathcal{T}$ is the set of nodes representing targets) and $e_{ij}\in E=\mathcal{A}$, where $\mathcal{A}=\left\{e_{ij}\middle| v_i\in V_\mathcal{D},v_j\in V_\mathcal{T},i\neq j,e_{ij}\neq0\right\}$ represents the set of drug-target interactions). Additionally, the set of non-edges in the DDI networks is $\bar{\mathcal{I}}=\left\{e_{ij}\middle| v_i,v_j\in\mathcal{D},i\neq j,e_{ij}=0\right\}\$ and in the DTI is $\bar{\mathcal{A}}=\left\{e_{ij}\middle| v_i\in\mathcal{D},v_j\in\mathcal{T},i\neq j,e_{ij}=0\right\}$.

A non-edge in a DDI network $({\bar{e}}_{ij}\in\bar{\mathcal{I}})$ or a DTI network $(\in\bar{\mathcal{A}})$ represents a drug-drug or a drug-target interaction that has a probability $p\in\left(0,1\right)$ of being discovered in the future (i.e., missing information generating uncertainty). If $p=0$, we are sure that interaction between v_i and v_j is impossible (i.e., the negative information on drug interactions)."

Comment 2. The manuscript covered a literature in terms of the methods using the DrugBank database. However, this literature is not enough and more discussion about drug repositioning applications using drug-drug and drug-target network-based methods or other types of applications using DrugBank datasets can improve the manuscript, specifically the literature review. Thus, citing more relevant and recent literature on DTI and DDI prediction is recommended, which will enrich the paper.

Response: The reviewer is correct in pointing out that the Background section only provides details and citations on the drug repositioning applications. Accordingly, we corrected this downside by explaining the drug-drug and drug-target interaction prediction benefits; we cited the relevant recent literature (please, see the blue-text in section Background, first 2 paragraphs on page 2.)

The new paragraph reads, "As such, harnessing big amounts of data describing the intricate drug interactions (or relationships) with other entities can lead to uncovering new drug properties: previously unknown drug-drug [3, 4, 5, 6] or drug-target interactions [7, 8, 9, 10, 11], drug repositioning [12, 13, 14]. Computational drug-drug interaction (DDI) prediction is critical in designing effective and safe drug combinations and developing drugs [5, 6]. Drug-target interaction (DTI) prediction is a key-step strategy in drug discovery that leads to uncovering new drugs for biological targets and new targets for known, approved drugs—representing a crucial step towards drug repositioning and identifying possible side effects [8, 11]."

[3] Zhang, W., Chen, Y., Liu, F., Luo, F., Tian, G. and Li, X., 2017. Predicting potential drug-drug interactions by integrating chemical, biological, phenotypic and network data. BMC bioinformatics, 18(1), pp.1-12.
[4] Zhu, J., Liu, Y., Zhang, Y., Chen, Z. and Wu, X., 2021. Multi-Attribute Discriminative Representation Learning for Prediction of Adverse Drug-Drug Interaction. IEEE Transactions on Pattern Analysis and Machine Intelligence.
[5] Qiu, Y., Zhang, Y., Deng, Y., Liu, S. and Zhang, W., 2021. A comprehensive review of computational methods for drug-drug interaction detection. IEEE/ACM Transactions on Computational Biology and Bioinformatics.
[6] Su, X., You, Z.H., Huang, D.S., Wang, L., Wong, L., Ji, B. and Zhao, B., 2022. Biomedical Knowledge Graph Embedding with Capsule Network for Multi-label Drug-Drug Interaction Prediction. IEEE Transactions on Knowledge and Data Engineering.
[7] Lu, Y., Guo, Y. and Korhonen, A., 2017. Link prediction in drug-target interactions network using similarity indices. BMC bioinformatics, 18(1), pp.1-9.
[8] Luo, Y., Zhao, X., Zhou, J., Yang, J., Zhang, Y., Kuang, W., Peng, J., Chen, L. and Zeng, J., 2017. A network integration approach for drug-target interaction prediction and computational drug repositioning from heterogeneous information. Nature communications, 8(1), pp.1-13.
[9] Wu, Z., Li, W., Liu, G. and Tang, Y., 2018. Network-based methods for prediction of drug-target interactions. Frontiers in pharmacology, 9, p.1134.
[10] Thafar, M.A., Olayan, R.S., Ashoor, H., Albaradei, S., Bajic, V.B., Gao, X., Gojobori, T. and Essack, M., 2020. DTiGEMS+: drug–target interaction prediction using graph embedding, graph mining, and similarity-based techniques. Journal of Cheminformatics, 12(1), pp.1-17.
[11] Thafar, M.A., Olayan, R.S., Albaradei, S., Bajic, V.B., Gojobori, T., Essack, M. and Gao, X., 2021. DTi2Vec: Drug–target interaction prediction using network embedding and ensemble learning. Journal of cheminformatics, 13(1), pp.1-18.
[12] Lotfi Shahreza, M., Ghadiri, N., Mousavi, S.R., Varshosaz, J. and Green, J.R., 2018. A review of network-based approaches to drug repositioning. Briefings in bioinformatics, 19(5), pp.878-892.
[13] Sadeghi, S.S. and Keyvanpour, M.R., 2019. An analytical review of computational drug repurposing. IEEE/ACM transactions on computational biology and bioinformatics, 18(2), pp.472-488.
[14] Badkas, A., De Landtsheer, S. and Sauter, T., 2021. Topological network measures for drug repositioning. Briefings in bioinformatics, 22(4), p.bbaa357.

Comment 3. The methodology section is a bit distracting. A summary paragraph can be included as well as the structure of this section can be improved.

Response: We appreciate this observation. The reviewer is correct; in the initially submitted manuscript, the Methodology section assembles all the subsections containing formal definitions, methods, and algorithms. Indeed, the Problem formulation is not a method, so we moved this part out of the Methods as a standalone/independent section (starting on page 3). We also added a summary paragraph to explain the structure of the Methods section, as suggested by the reviewer. The new paragraph, highlighted in blue on page 5, at the beginning of the section Methods, reads:
"In this section, we describe the statistical and computational methods employed in this paper: subsection Network analysis introduces the main metrics and centralities required for the computational and statistical analysis of complex drug networks; subsection Database quality presents the statistical methods we use to test the drug database integrity; subsection Network analysis robustness explains the computer simulation method we propose to assess the robustness of the centrality-based drug network analysis strategies;

subsection Computational methods describes the tools we use in this paper to implement the required computational and statistical techniques."

Comment 4. The benefit of Figure 3 is not clear. Better description with the purpose of the figure will add more value.

Response: We thank the reviewer for raising this issue. We introduced Figure 3 to show the evolution of size in drug-drug and drug-target interaction networks (i.e., as the number of drugs and targets) with the DrugBank versions. As shown, compared with the DDI and DTI sizes in the first available DrugBank version, the number of medicines/drugs almost quadrupled; in contrast, the number of targets increased at a much lower rate. This observation is in line with the other results reported in our paper, thus strengthening the overarching conclusion.

In the revised form of our manuscript, according to the reviewer's suggestion, we introduced Figure 3 with a clarifying statement. The statement now reads, "In Figure 3, panel a, we present the evolution of the number of nodes (i.e., drugs) in DDI networks; in Figure 3, panel b, we show the evolution of the number of nodes, drugs, and targets in DTI networks. As shown, from version 3.0 to 5.1.9, the number of medicines/drugs roughly quadrupled; in contrast, the number of targets increased at a much lower rate. Such an evolution suggests that, over the years, the complexity of processing and analyzing drug interaction networks grew considerably." In our revised manuscript, we highlighted this change with blue text, on page 7, section Results, subsection Network metrics and centrality analysis, paragraph 2.

Comment 5. The text can be more concise to improve the text quality especially in the discussion.

Response: The reviewer is correct: our Discussion section needs better structure and wording. According to the suggestion, we performed the following steps:
We renamed the subsections of the Discussion as Discussions on DDI networks and Discussions on DTI networks.
We rewrite the entire Discussion section to eliminate redundant wording, thus bringing more conciseness and clarity to the manuscript.
Please find the revised form of the Discussion section in blue, starting on page 10.

Close
