# Supplementary material for: The curse and blessing of abundance—the evolution of drug interaction databases and their impact on drug network analysis
Source: Gigascience. 2023 Mar 9;12:giad011. doi: 10.1093/gigascience/giad011 (PMC10023830; doi:10.1093/gigascience/giad011)
Supplement: giad011_Response_to_Reviewer_Comments_Revision_1 [file giad011_response_to_reviewer_comments_revision_1.pdf]

Author's Response To Reviewer Comments

Close

Editor: 1) Please include a citation to your new GigaDB dataset (including the DOI link) to your reference list, and cite this by number in the data availability section.
The reference is:
[xx] Udrescu M; Ardelean SM; Udrescu L Supporting data for "The curse and blessing of abundance-the evolution of drug interaction databases and their impact on drug network analysis." GigaScience Database. 2023 http://dx.doi.org/10.5524/102351
For example, write: "An archival copy of the github repository is available via the Gigascience database GigaDB [xx]."

Response 1: We cite our new GigaDB dataset as reference [85] in the revised manuscript (section Code Availability).

2) Also in the "availability" section, please move the URL of your github repository to the bibliography and cite it by number, analogous to the example above. (At Gigascience, we treat all data sources, including internet URLs, as citable items that should be part of the bibliography).

Response 2: We cite the supporting GitHub as reference [86] in the revised manuscript (section Code Availability).

3) Please add ORCID IDs to the title page. We have the following in our files: ORCID iDs: Mihai Udrescu [0000-0002-7607-9240]; Sebastian Mihai Ardelean [0000-0003-0968-1191]; Lucretia Udrescu [0000-0002-3084-6301];

Response 3: We added the ORCID IDs for all authors in the authors' list.

4) The conversion of the LaTex source in Editorial Manager didn't work for some reason. If this persists, that's not a problem at this stage, you can upload the converted PDF for now and add all LaTex source files as supplemental material. Please make sure the LaTex source files are the latest revised version, including the minor edits above.

Response 4: We "sanitized" the main LaTex file, as well as the bib file, and now we have no error in Overleaf (only warnings). Please find all the files (including the updated ones) in our new submission. We hope the conversion will work now.

Close
