# Supplementary material for: The curse and blessing of abundance—the evolution of drug interaction databases and their impact on drug network analysis
Source: Gigascience. 2023 Mar 9;12:giad011. doi: 10.1093/gigascience/giad011 (PMC10023830; doi:10.1093/gigascience/giad011)
Supplement: giad011_Reviewer_1_Report_Original_Submission — Wen Zhang -- 10/7/2022 [file giad011_reviewer_1_report_original_submission.pdf]

Reviewer Report

Title: The curse and blessing of abundance---the evolution of drug interaction databases and their impact on drug network analysis

Version: Original Submission **Date:** 10/7/2022

Reviewer name: Wen Zhang

Reviewer Comments to Author:

In recent years, more and more comprehensive drug datasets have been proposed, however, there is much uncertainty in these drug datasets. Researchers cannot know if the not reported interactions are absent or yet to be discovered, which hampers the accuracy of bioinformatics applications on these datasets. The authors used complex network statistics tools and simulations of randomly-inserted previously unaccounted interactions in drug-drug and drug-target interaction networks, and found that the drug-drug interaction networks built with the latest dataset versions become very dense and, therefore, almost impossible to analyze with conventional complex network methods. On the other hand, for the latest drug database versions, drug-target networks still include much uncertainty; however, the robustness of complex network analysis methods slightly improves. The authors pinpoint future research directions to improve the quality and practicality of drug databases for bioinformatics applications: benchmarking for drug-target interaction prediction and drug-drug interaction severity standardization.
Comments and suggestions:
1. The author should explain more clearly the relationship among 'uncertain issue of datasets', 'the evolution of various complex network parameters and centralities', 'the integrity of the processed data' and 'the robustness of centrality-hierarchization analysis methods'.
2. The author should clearly define the 'typical complex network', for example, the characteristics of a typical complex network and the differences between a typical complex network and a normal network. In addition, the first sentence of the first paragraph in the section 'Complex networks' seems to be a general definition of the 'network', not the 'complex network'.
3. In the last sentence of the third paragraph in the section 'Complex networks', 'wij Ì¸= wji' should be 'wij=wji'.
4. In Equation (10), the function Î·() doesn't seem to be defined.
5. In the section 'Discussion', the authors should include more analysis of the results. In addition, I suggest that the authors should elaborate analysis and conclusions with relevant findings from the experimental results, instead of talking about them separately.
6. Many important literature about the drug interactions have not been reviewed in the paper, such as DOI:10.1109/TPAMI.2021.3135841, DOI:10.1109/TKDE.2022.3154792, doi: 10.1186/s12859-016-1415-9, DOI:10.1109/TCBB.2021.3081268, etc.

Methods

Are the methods appropriate to the aims of the study, are they well described, and are necessary controls included? Choose an item.

Conclusions

Are the conclusions adequately supported by the data shown? Choose an item.

Reporting Standards

Does the manuscript adhere to the journal's guidelines on minimum standards of reporting? Choose an item.

Choose an item.

Statistics

Are you able to assess all statistics in the manuscript, including the appropriateness of statistical tests used? Choose an item.

Quality of Written English

Please indicate the quality of language in the manuscript: Choose an item.

Declaration of Competing Interests

Please complete a declaration of competing interests, considering the following questions:

- Have you in the past five years received reimbursements, fees, funding, or salary from an organisation that may in any way gain or lose financially from the publication of this manuscript, either now or in the future?
- Do you hold any stocks or shares in an organisation that may in any way gain or lose financially from the publication of this manuscript, either now or in the future?
- Do you hold or are you currently applying for any patents relating to the content of the manuscript?
- Have you received reimbursements, fees, funding, or salary from an organization that holds or has applied for patents relating to the content of the manuscript?
- Do you have any other financial competing interests?
- Do you have any non-financial competing interests in relation to this paper?

If you can answer no to all of the above, write 'I declare that I have no competing interests' below. If your reply is yes to any, please give details below.

I declare that I have no competing interests

I agree to the open peer review policy of the journal. I understand that my name will be included on my report to the authors and, if the manuscript is accepted for publication, my named report including any attachments I upload will be posted on the website along with the authors' responses. I agree for my report to be made available under an Open Access Creative Commons CC-BY license (http://creativecommons.org/licenses/by/4.0/). I understand that any comments which I do not wish to be included in my named report can be included as confidential comments to the editors, which will not be published.

Choose an item.

To further support our reviewers, we have joined with Publons, where you can gain additional credit to further highlight your hard work (see: https://publons.com/journal/530/gigascience). On publication of this paper, your review will be automatically added to Publons, you can then choose whether or not to claim your Publons credit. I understand this statement.

Yes Choose an item.
