# Supplementary material for: The curse and blessing of abundance—the evolution of drug interaction databases and their impact on drug network analysis
Source: Gigascience. 2023 Mar 9;12:giad011. doi: 10.1093/gigascience/giad011 (PMC10023830; doi:10.1093/gigascience/giad011)
Supplement: giad011_Reviewer_2_Report_Original_Submission — Maha A. Thafar, Ph.D. -- 10/22/2022 [file giad011_reviewer_2_report_original_submission.pdf]

Reviewer Report

Title: The curse and blessing of abundance---the evolution of drug interaction databases and their impact on drug network analysis

Version: Original Submission **Date:** 10/22/2022

Reviewer name: Maha A. Thafar, Ph.D.

Reviewer Comments to Author:

Journal:
GigaScience
Date: 2022, 1-20.
Title: The curse and blessing of abundanceâ€"the evolution of drug interaction databases and their impact on drug network analysis
Overview Summary:
The authors proposed an interesting analysis study for several drug-related networks using different versions of the DrugBank database. Therefore, the work is network analysis and machine learning-based methods to analyze the future of drug-drug interactions and drug-target interactions. Hence, this study highlights the future research that can improve the drug databases quality and for bioinformatics applications.
This work tackles a genuine topic to improve bioinformatics applications using complex networks of drug-related data. The abstract and the introduction were well written and presented good motivation, description. However, I have some concerns that have to be addressed. Therefore, I do recommend this work for publications in the GigaScience journal after addressing the following comments.
Comments
Comment 1. The problem formulation is not very clear. More details are needed for the negative or missing information but focused on the problem itself.
Comment 2. The manuscript covered a literature in terms of the methods using the DrugBank database. However, this literature is not enough and more discussion about drug repositioning applications using drug-drug and drug-target network-based methods or other types of applications using DrugBank datasets can improve the manuscript, specifically the literature review. Thus, citing more relevant and recent literature on DTI and DDI prediction is recommended, which will enrich the paper.
Comment 3. The methodology section is a bit distracting. A summary paragraph can be included as well as the structure of this section can be improved.
Comment 4. The benefit of Figure 3 is not clear. Better description with the purpose of the figure will add more value.
Comment 5. The text can be more concise to improve the text quality especially in the discussion.

Methods

Are the methods appropriate to the aims of the study, are they well described, and are necessary controls included? Choose an item.

Conclusions

Are the conclusions adequately supported by the data shown? Choose an item.

Reporting Standards

Does the manuscript adhere to the journal's guidelines on minimum standards of reporting? Choose an item.

Choose an item.

Statistics

Are you able to assess all statistics in the manuscript, including the appropriateness of statistical tests used? Choose an item.

Quality of Written English

Please indicate the quality of language in the manuscript: Choose an item.

Declaration of Competing Interests

Please complete a declaration of competing interests, considering the following questions:

- Have you in the past five years received reimbursements, fees, funding, or salary from an organisation that may in any way gain or lose financially from the publication of this manuscript, either now or in the future?
- Do you hold any stocks or shares in an organisation that may in any way gain or lose financially from the publication of this manuscript, either now or in the future?
- Do you hold or are you currently applying for any patents relating to the content of the manuscript?
- Have you received reimbursements, fees, funding, or salary from an organization that holds or has applied for patents relating to the content of the manuscript?
- Do you have any other financial competing interests?
- Do you have any non-financial competing interests in relation to this paper?

If you can answer no to all of the above, write 'I declare that I have no competing interests' below. If your reply is yes to any, please give details below.

NO I declare that I have no competing interests' below.

I agree to the open peer review policy of the journal. I understand that my name will be included on my report to the authors and, if the manuscript is accepted for publication, my named report including any attachments I upload will be posted on the website along with the authors' responses. I agree for my

report to be made available under an Open Access Creative Commons CC-BY license (http://creativecommons.org/licenses/by/4.0/). I understand that any comments which I do not wish to be included in my named report can be included as confidential comments to the editors, which will not be published.

Choose an item.

To further support our reviewers, we have joined with Publons, where you can gain additional credit to further highlight your hard work (see: https://publons.com/journal/530/gigascience). On publication of this paper, your review will be automatically added to Publons, you can then choose whether or not to claim your Publons credit. I understand this statement.

Yes Choose an item.
